# Non-classical ferroptosis inhibition by a small molecule targeting PHB2

**DOI:** 10.1038/s41467-022-35294-2

**Published:** 2022-12-03

**Authors:** Wei Yang, Bo Mu, Jing You, Chenyu Tian, Huachao Bin, Zhiqiang Xu, Liting Zhang, Ronggang Ma, Ming Wu, Guo Zhang, Chong Huang, Linli Li, Zhenhua Shao, Lunzhi Dai, Laurent Désaubry, Shengyong Yang

**Affiliations:** 1 State Key Laboratory of Biotherapy and Cancer Center, West China Hospital, Sichuan University, Chengdu, Sichuan 610041 China; 2 North Sichuan Medical College, Nanchong, Sichuan 637000 China; 3 Key Laboratory of Drug Targeting and Drug Delivery System of the Education Ministry, West China School of Pharmacy, Sichuan University, Chengdu, Sichuan 610041 China; 4 Regenerative Nanomedicine Laboratory (UMR1260), INSERM-University of Strasbourg, Center of Research in Biomedicine of Strasbourg (CRBS), Strasbourg, France

**Keywords:** Cell death, Target identification, Target identification

## Abstract

Ferroptosis is a new type of programmed cell death characterized by iron-dependent lipid peroxidation. Ferroptosis inhibition is thought as a promising therapeutic strategy for a variety of diseases. Currently, a majority of known ferroptosis inhibitors belong to either antioxidants or iron-chelators. Here we report a new ferroptosis inhibitor, termed YL-939, which is neither an antioxidant nor an iron-chelator. Chemical proteomics revealed the biological target of YL-939 to be prohibitin 2 (PHB2). Mechanistically, YL-939 binding to PHB2 promotes the expression of the iron storage protein ferritin, hence reduces the iron content, thereby decreasing the susceptibility to ferroptosis. We further showed that YL-939 could substantially ameliorate liver damage in a ferroptosis-related acute liver injury model by targeting the PHB2/ferritin/iron axis. Overall, we identified a non-classical ferroptosis inhibitor and revealed a new regulation mechanism of ferroptosis. These findings may present an attractive intervention strategy for ferroptosis-related diseases.

## Abstract (web summary)

Ferroptosis is a promising therapeutic target for a variety of diseases, but a majority of known ferroptosis inhibitors belong to either antioxidants or iron chelators. Here, the authors discover a new non-classical small molecule inhibitor that is a PHB2 binder and show it ameliorates liver damage in an acute liver injury model.

## Introduction

Ferroptosis is an iron-dependent regulated necrotic cell death caused by lipid peroxidation^1^. It is strictly modulated by cellular antioxidant systems and iron metabolism. Currently, at least four signal pathways have been identified to be involved in the antioxidant systems and ferroptosis regulation: system x_c_^−^—glutathione (GSH)–glutathione peroxidase 4 (GPX4)^2^, NADPH—ferroptosis suppressor protein 1 (FSP1)–CoQ10^3,4^, GTP cyclohydrolase 1 (GCH1)—tetrahydrobiopterin (BH_4_)–dihydrofolate reductase (DHFR)^5^, and dihydroorotate dehydrogenase (DHODH)–ubiquinol^6^. Dysregulation of the cellular antioxidant systems may cause lipid peroxidation, which then triggers a radical chain reaction within lipid bilayers, and eventually results in cell death^7^. Iron is an essential driving factor of intracellular lipid peroxidation and ferroptosis. A number of proteins regulating iron metabolism have been discovered, such as transferrin receptor 1 (TFR1) and divalent metal transporter 1 (DMT1) for iron transport and uptake^8^, ferroportin (FPN) for intracellular iron export^9^, and ferritin for the iron storage^10^. Abnormality of iron metabolism may lead to disturbed iron-homeostasis. Iron overload may trigger and catalyze lipid peroxidation by Fenton reaction and cause ferroptosis^11^. Antioxidants and iron chelators can efficiently suppress ferroptosis.

Ferroptosis has been linked to a variety of diseases and ferroptosis inhibition is thought as a promising strategy to the treatment of related diseases, such as ischemia-reperfusion injury^12,13^, acute organ injury^14–16^, and neurodegenerative diseases^17,18^. Currently, a number of ferroptosis inhibitors have been reported^17,19,20^. Nonetheless, a majority of them belong to the classical antioxidants or iron chelators. It has been established that redox balance in the body’s internal environment is critical for health^21^. Breaking the redox balance by reducing the oxidation state non-selectively may cause diseases, for example, cancer^22^ and senescence^23^. In addition, iron is a crucial trace element that is essential for fundamental physiological processes, thus mammals have developed elegant mechanisms for keeping both cellular and whole-body iron concentrations within the optimal physiologic range^8,24^. The use of iron chelators to eliminate the iron inevitably leads to side effects, such as anemia^25^ and many metabolic diseases^26^. Therefore, it is of great clinical significance to develop ferroptosis inhibitors that are neither antioxidants nor iron chelators. Here we report such a small molecule ferroptosis inhibitor, termed YL-939. Chemical proteomics revealed that the biological target of YL-939 is prohibitin 2 (PHB2), which was further confirmed by various molecular biology experiments. The underlying mechanism of PHB2 regulating ferroptosis was also explored. We finally investigated the in vivo effect of YL-939 in a ferroptosis-related acute liver injury model.

## Results

### Discovery of the non-classical ferroptosis inhibitor YL-939

**Fig. 1:**
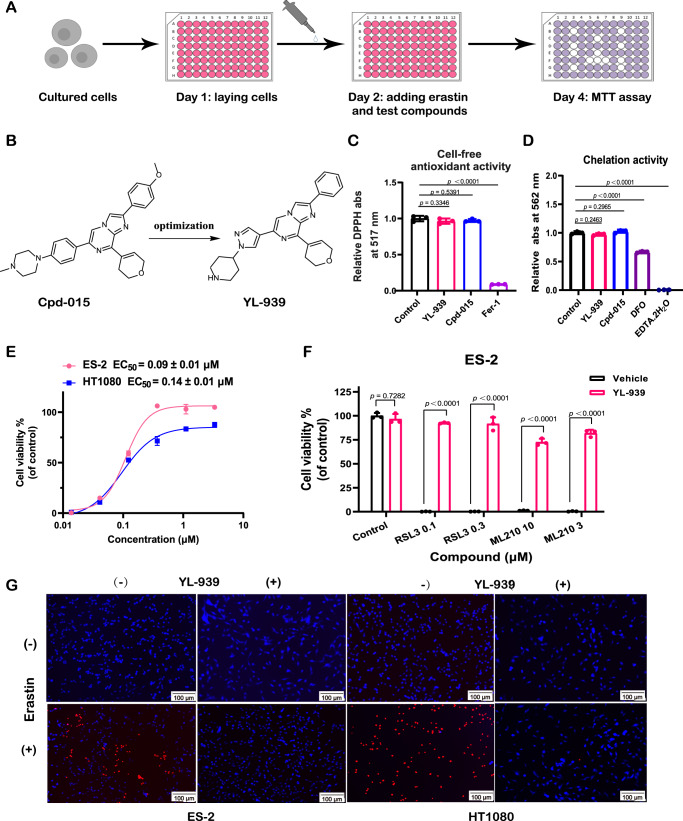
Discovery of the non-classical ferroptosis inhibitor YL-939. **A** Screening scheme of ferroptosis inhibitors. Erastin (10 μM) and test compounds (3 μM) were added to the cultured ES-2 cells. After 48 h treatment, MTT assay was used to detect cell viability. **B** Chemical structure of Cpd-015 and YL-939. **C** The antioxidant activities of Cpd-015 (50 μM) and YL-939 (50 μM) analyzed by DPPH (2,2-Diphenyl-1-Picrylhydrazyl) assay. Data represent the mean ± SD of three biological replicates. Statistical analyses were performed by one-way ANOVA with Dunnett’s multiple comparisons test. Specific *p*-values are indicated in the figure. **D** Iron chelating ability of Cpd-015 (50 μM) and YL-939 (50 μM). Data represent the mean ± SD of three biological replicates. Statistical analyses were performed by one-way ANOVA with Dunnett’s multiple comparisons test. Specific *p*-values are indicated in the figure. **E** Cell protective effect of YL-939 on erastin-induced ES-2 and HT1080 cell ferroptosis models. Data represent mean of two independent biological replicates. **F** Cell protective effect of YL-939 on different inducers of ferroptosis in ES-2 cells. Data represent the mean ± SD of three biological replicates. Statistical analyses were performed by two-way ANOVA with Sidak’s multiple comparisons test. Specific *p*-values are indicated in the figure. **G** Dual staining with CytoCalcein™ violet 450 (blue, living cell) and 7-AAD (red, ferroptotic cells) of ES-2 and HT1080 cells treated with or without erastin for 10 h. Representative images of two biological replicates are shown. Scale bars: 100 μm. Source data are provided as a Source data file.

To discover ferroptosis inhibitors, an erastin-induced ES-2 cell ferroptosis model was used to screen an in-house chemical library containing about 4000 compounds (Fig. 1A); erastin is a potent inhibitor of system x_c_^−^ and a widely used inducer of ferroptosis^1,27,28^. In this screening campaign, 16 compounds were found to be able to protect cells from ferroptosis at a concentration of 3 μM (Supplementary Fig. 1). We then examined their antioxidation activity by a free radical scavenging assay (DPPH) and iron-chelation ability by a ferrozine-based colorimetric assay (Supplementary Fig. 2A and B). The results showed that only one compound, Cpd-015 (Fig. 1B), had no activity for both antioxidation and iron-chelation (Fig. 1C and D). The half-effective concentration (EC_50_) of Cpd-015 in the erastin-induced ES-2 cell ferroptosis model is 2.82 μM (Supplementary Fig. 2C), indicating a moderate potency.

**Fig. 2:**
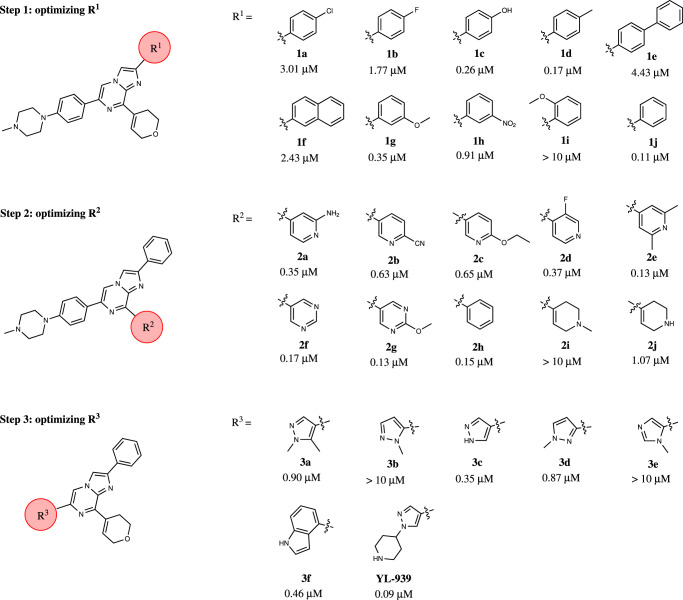
A stepwise structural optimization process toward Cpd-015. Step 1: we fixed R^2^ and R^3^ and optimized R^1^. Step 2: we optimized R^2^ with R^1^ fixed as the optimal phenyl group and R^3^ as its original subgroup. Step 3: we fixed R^1^ and R^2^ as their optimal subgroups and optimized R^3^.

To improve the potency of Cpd-015, a stepwise optimization process was carried out, which focused on three subgroups: 4-methoxybenzene (R^1^), 3,6-dihydro-2H-pyran (R^2^), and 1-methyl-4-phenylpiperazine (R^3^) (Fig. 2). First, we fixed R^2^ and R^3^ and optimized R^1^. A total of 10 new compounds (**1a-1j**) with varied R^1^ were synthesized, and compound **1j** harboring a phenyl group at R^1^ showed the highest potency. Second, we optimized R^2^ with R^1^ fixed as the optimal phenyl group and R^3^ as its original subgroup. Another 10 new compounds (**2a-2j**) with different R^2^ were synthesized and compound **1j** was still the most potent one. Finally, we fixed R^1^ and R^2^ as their optimal subgroups and optimized R^3^. 7 new compounds were prepared and compound YL-939 was the most active one (Figs. 1B and 2), which showed an EC_50_ value of 0.09 μM in the erastin-induced ES-2 cell ferroptosis model (Fig. 1E). Similar as Cpd-015, YL-939 is neither an antioxidant nor an iron-chelator (Fig. 1C and D).

To verify the anti-ferroptosis effect of YL-939, we tested the activity of YL-939 on another five different cell lines, including HT-1080, Miapaca-2, Calu-1, HCT116 and SHSY5Y. YL-939 displayed very similar effect as that on ES-2 with EC_50_ values of 0.14 μM, 0.25 μM, 0.16 μM, 0.16 μM and 0.24 μM, respectively, indicating a cell-independent effect (Fig. 1E and Supplementary Fig. 3A). Moreover, we used other reported ferroptosis inducers to induce ferroptosis, including RSL3^29^ and ML210^30^. In all these assays, YL-939 efficiently protected cells from ferroptosis (Fig. 1F). The anti-ferroptosis effect of YL-939 was also visualized in cytocalcein violet 450 (staining living cells) and 7-AAD (staining dead cells) dyeing experiments (Fig. 1G). Moreover, the cytotoxicity of YL-939 was determined using the methyl thiazolyl tetrazolium (MTT) colorimetry assay. YL-939 did not show obvious cytotoxicity against six normal cell lines, including L02, LX-2, Beas-2b, HUVEC, Arpe and hTERT-HPNE at concentrations less than 3 μM, but displayed evident cytotoxicity when concentrations are larger than 3 μM (Supplementary Fig. 3B).

**Fig. 3:**
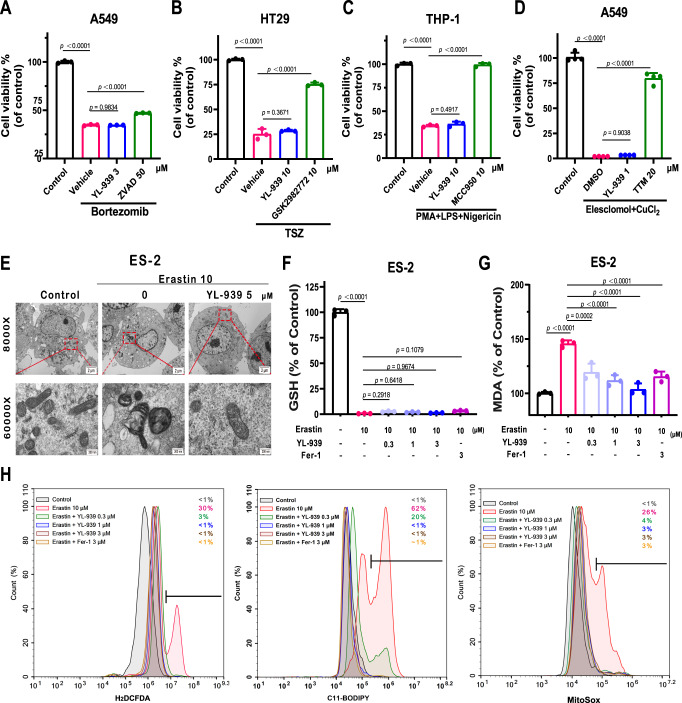
YL-939 specifically protected cells from ferroptosis. **A** Effect of YL-939 on the bortezomib-induced A549 cell apoptosis model. ZVAD was used as a positive compound. Data represent the mean ± SD of three biological replicates. **B** Effect of YL-939 on the HT29 cell necroptosis model induced by TNFα (10 ng mL^−1^), Smac-mimetic (100 nM), and Z-VAD-FMK (20 mM). GSK2982772 was used as a positive compound. Data represent the mean ± SD of three biological replicates. **C** Effect of YL-939 on the cell pyroptosis model induced by phorbol-12-myristate-13-acetate (PMA), lipopolysaccharide (LPS), and nigericin. MCC950 was used as a positive compound. Data represent the mean ± SD of three biological replicates. **D** Effect of YL-939 on the elesclomol/CuCl_2_-induced cuprotosis model. Tetrathiomolybdate (TTM) was used as a positive compound. Data represent the mean ± SD of four biological replicates. **E** Transmission electron microscopy of ES-2 cells treated with DMSO (10 h), erastin (10 μM) with or without YL-939 (5 μM, 10 h). Representative images of two biological replicates are shown. Scale bars: 2 μm or 200 nm. **F**, **G** Effects of YL-939 on the GSH level and lipid oxidation level (indicated by MDA) in ferroptosis. Data represent the mean ± SD of three biological replicates. **H** Cytosolic, lipid, and mitochondrial ROS production assessed by flow cytometry using H_2_DCFDA, C11-BODIPY, and MitoSox. Data were obtained from two independent biological replicates. For **A**–**D** and **F**, **G**, statistical analyses were performed by one-way ANOVA with Dunnett’s multiple comparisons test. Specific *p*-values are indicated in the figure. Source data are provided as a Source data file.

We further showed that YL-939 did not rescue cell death in bortezomib-induced cell apoptosis^31^ (Fig. 3A), TNFα/Smac-mimetic/z-VAD-FMK (TSZ)-induced necroptosis^32^ (Fig. 3B), nigericin-induced pyroptosis^33^ (Fig. 3C), and elesclomol/CuCl_2_-induced cuprotosis^34^ (Fig. 3D) models, indicating that YL-939 is not an inhibitor of apoptosis, necroptosis, pyroptosis, and cuprotosis. Transmission electron microscopy experiments revealed that YL-939 protected erastin-induced mitochondrial cristae disappearance and outer membrane rupture, a typical phenotype of ferroptosis^1^ (Fig. 3E). Further experiments showed that YL-939 treatment did not impact the level of GSH (Fig. 3F), while substantially reduced the level of malondialdehyde (MDA) (Fig. 3G), a lipid peroxidation product, compared with untreated cells. We next examined the impact of YL-939 on the reactive oxygen species (ROS), which play a critical role in the progression of ferroptosis. Treatment of ES-2 cells with erastin (10 μM) resulted in an increase of ROS in cytosolic, membrane lipids, and mitochondria at 10 h, as assayed by flow cytometry using the fluorescent dyes H_2_DCFDA, C11-BODIPY, and MitoSox, respectively (Fig. 3H). YL-939 treatment substantially diminished ROS in these cellular elements (Fig. 3H), further indicating that YL-939 is an effective ferroptosis inhibitor.

### PHB2 was identified as the biological target of YL-939

**Fig. 4:**
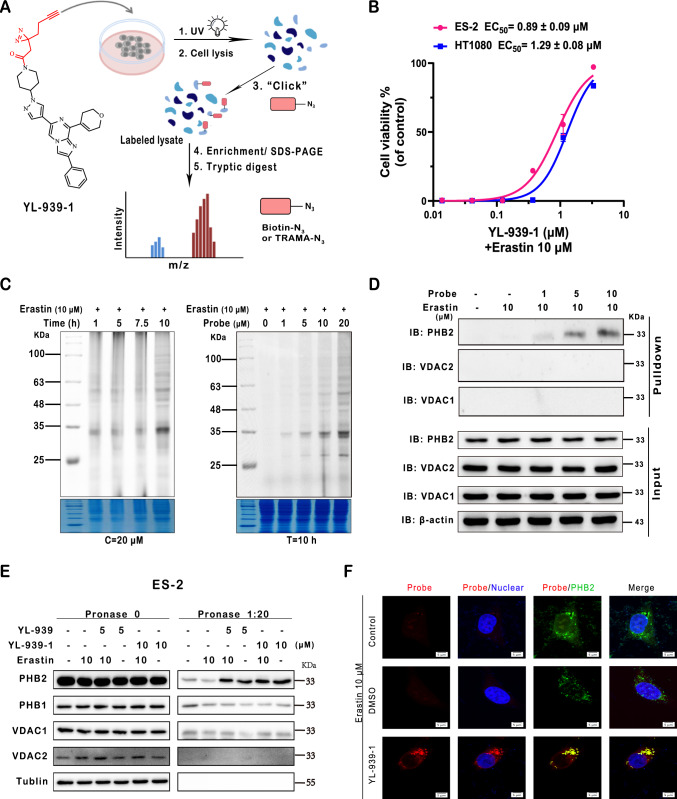
PHB2 was identified as the biological target of YL-939 in cells. **A** The chemical structure of YL-939-1 and the schematic process for the activity-based protein profiling (ABPP) experiments. **B** Cell protection effects of YL-939-1 in erastin-induced ferroptosis models (HT1080 and ES-2). Data represent mean of two independent biological replicates. **C** Time- and concentration-dependent labeling profiles of YL-939-1 with ES-2 cells. Blots shown are representative of two biological replicates. **D** Identification of YL-939-1 binding proteins using pull-down/western blot. Blots shown are representative of two biological replicates. **E** Identification of YL-939-1 and YL-939 binding proteins using DARTS. Blots shown are representative of two biological replicates. **F** Cellular imaging of the probes (in red) and immunofluorescence of the PHB2 protein (in green) in ES-2 cells. Representative images of two biological replicates are shown. Scale bars: 5 μm. Source data are provided as a Source data file.

To rapidly determine the biological target of YL-939, we first tested the activity of YL-939 against a panel of 401 recombinant human protein kinases by using the kinase profiling assay provided by Eurofins, since kinases are often the key components in various signal pathways^35,36^. YL-939 showed very weak or no activity in this assay (Supplementary Table 1), implying that YL-939 does not bind to these kinases. We then carried out affinity-based proteome profiling assay (ABPP) coupled with bioimaging to identify the biological target of YL-939 (Fig. 4A). To this end, we designed and synthesized a photoaffinity probe, YL-939-1 (Fig. 4A and Supplementary Fig. 5), which is YL-939 attached by a photo-crosslinker (L3) that contains a photo-reactive group diazirine and a clickable alkyne handle^37^. This probe showed EC_50_ values of 0.92 μM and 1.08 μM in erastin-induced ES-2 and HT1080 cell ferroptosis models, respectively (Fig. 4B), indicating being suitable for labeling profile.

Next, the probe was incubated with ES-2 cells for 1–10 h. After that, the mixture was exposed to UV light (365 nm) for 20 min and then conjugated with TAMRA-N_3_. The proteins labeled were separated by SDS-PAGE followed by in-gel fluorescence scanning. As shown in Fig. 4C, probe YL-939-1 labeled a 33 kDa protein in a time- and concentration-dependent manner. Subsequently, a biotin-streptavidin pull-down was performed with probe YL-939-1: YL-939-1 and cells were incubated, and the mixture was exposed to UV light (365 nm) for 20 min. The YL-939-1 binding proteins were conjugated to biotin-azide, using click chemistry, and captured with streptavidin beads, followed by digestion and analysis, using liquid chromatography-tandem mass spectrometry (LC-MS/MS). The results showed that there were about 400 proteins labeled by the probe molecule (Supplementary Table 2). Proteins with molecular weight of about 33 KDa are shown in Supplementary Table 3. Considering the intensity values in the mass spectrometry experiment, we selected three proteins, VDAC1, VDAC2, and PHB2, to perform pull-down/western blot analysis, which revealed that only PHB2 protein could be pulled down by the probe (Fig. 4D), indicating that PHB2 is most likely the binding protein of YL-939.

To confirm the binding of YL-939/YL-939-1 with PHB2 in situ, we performed a label-free small-molecule target identification assay called drug affinity-responsive target stability (DARTS)^38^ followed by western blot. It was observed that YL-939/YL-939-1 protected PHB2 from the degradation by pronase, but had no effect on the PHB2 isoform PHB1, as well as VDAC1 and VDAC2 (Fig. 4E). Subsequent bioimaging experiments further showed that the fluorescence signals produced from the probe well overlaped with that from PHB2 (Fig. 4F). Meanwhile, we conducted fixed cell imaging with YL-939-1 probe on PHB2-depleted and PHB2-expressed cells, respectively. The proteins that could be labeled by YL-939-1 was substantially reduced in the PHB2 knockdown cells, while in the cells that re-expressed PHB2, the labeling efficiency of YL-939-1 was significantly increased (Supplementary Fig. 6). All the results demonstrated that YL-939 binds to PHB2 in intact cells.

**Fig. 5:**
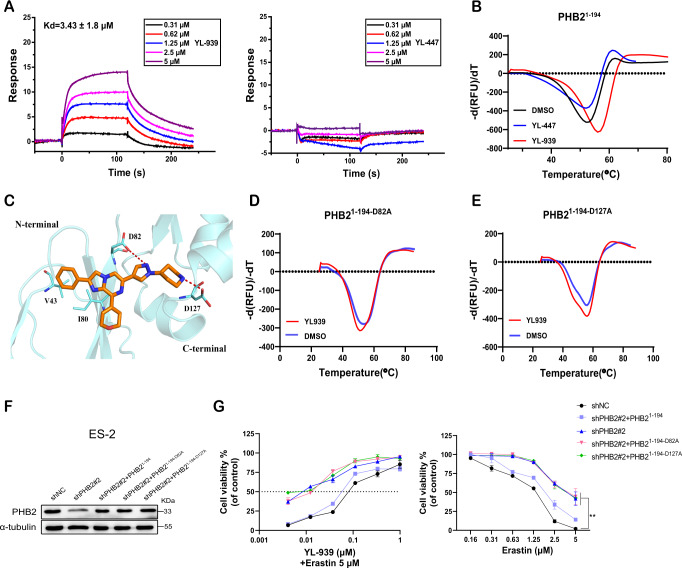
YL-939 binds to the PHB2 protein. **A** SPR analysis of YL-939 (left) or YL-447 (right) binding to the recombinant PHB2^1–194^ protein. Data were obtained from two biological replicates. **B** DSF analysis of YL-939 or YL-447 binding to the recombinant PHB2^1–194^ protein. Data were obtained from three biological replicates. **C** A predicted binding mode of YL-939 with PHB2^1–194^. The three-dimensional structure of PHB2 predicted by AlphaFold (https://alphafold.ebi.ac.uk/entry/Q99623) was used as the receptor structure. **D** DSF analysis of YL-939 binding to the recombinant mutated PHB2^1-194-D82A^ protein. Data were obtained from three biological replicates. **E** DSF analysis of YL-939 binding to the recombinant mutated PHB2^1-194-D127A^ protein. Data were obtained from three biological replicates. **F** Protein expression of each recombinant PHB2. Blots shown are representative of two biological replicates. **G** Effects of PHB2 mutants or wild type on ferroptosis protection of YL-939 treatment and sensitivity of cells to ferroptosis. Data represent the mean ± SD of three biological replicates. Statistical analyses were performed by two-way ANOVA with Sidak’s multiple comparisons test: **p < 0.01. Source data are provided as a Source data file.

We then expressed and purified the PHB2^1–194^ protein in an *E. coli* expression system. Differential scanning fluorimetry (DSF) and surface plasmon resonance (SPR) analysis were used to measure the binding ability of YL-939 and PHB2. Here YL-447, which is a structurally similar compound as YL-939 but has no activity in ferroptosis inhibition assay at a concentration of 10 μM (Supplementary Fig. 7), was used as a negative control. YL-939 showed a K_d_ value of 3.43 μM in the SPR assay and a thermal shift (∆T_m_) of 2.65 °C in the DSF assay, while the negative control did not show activity in the two assays (Fig. 5A, B).

We next explored the possible binding mode between YL-939 and PHB2. Because we failed to obtain the crystal structure of PHB2, molecular docking was thus used to predict the binding site of YL-939, in which the three-dimensional structure of PHB2 predicted by AlphaFold (https://alphafold.ebi.ac.uk/entry/Q99623) was taken as the receptor structure due to lack of PHB2 structural information. The docking result showed that YL-939 may locate at an area between the N-terminal lobe (composed of β-sheets) and C-terminal lobe (composed of β-sheets and α-helixes) (Fig. 5C). Two hydrogen bonds are formed: one is between the nitrogen atom of pyrazole ring and residue D82, and the other one is between the nitrogen atom of piperidine ring and residue D127. The distal phenyl group forms hydrophobic interaction with residues V43 and I80. To verify the predicted binding mode, we generated two mutants of PHB2: D82A and D127A, and re-tested the binding ability of YL-939 by DSF. The results showed that the two mutants almost abolished the binding of YL-939 to PHB2 (Fig. 5D, E). By the way, we examined the impact of the two mutants on the cell ferroptosis. To this end, the PHB2 mutant plasmids were transfected into PHB2-knockdown cells (ES-2-shPHB2#2) (Fig. 5F). As shown in Fig. 5G, compared with PHB2 wide-type, the D82A and D127A mutants reduced the ferroptosis sensitivity to erastin.

Because several small molecule compounds have recently been reported to be able to bind to PHB2, one may wonder whether these PHB2 inhibitors can protect cells from ferroptosis. To examine this issue, we tested two representative PHB2 binders, FL3^39,40^ and JI051^41^ (Supplementary Fig. 8A). Our results showed that the two compounds could indeed bind to PHB2 (Supplementary Fig. 8B), but displayed very weak activity in protecting ES-2 cells from erastin-induced ferroptosis (Supplementary Fig. 8C). Further cytotoxicity assays indicated that the two compounds displayed much larger cytotoxicity compared with YL-939, which offset the cell protection effect against ferroptosis (Supplementary Fig. 8D). To know whether FL3 and JI051 bind to the same binding site as YL-939, we used the PHB2 D82A and D127A mutation assays again. The results showed that the mutants had very weak or no impact on the binding of the two compounds (Supplementary Fig. 8B), implying that FL3 and JI051 may potentially bind to a site different from that of YL-939.

### Engagement of PHB2 in the ferroptosis regulation

**Fig. 6:**
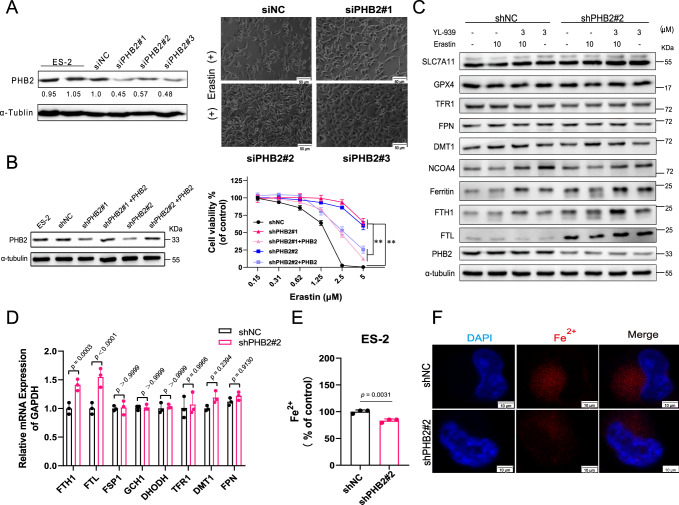
PHB2 participates in the regulation of ferroptosis and mechanism of action. **A** Knockdown of PHB2 using siRNA abolished erastin-induced cell death. Western Blot analysis of PHB2 protein expression is shown on the left. Blots shown are representative of two biological replicates. ES-2 cells transient transfected siRNA were treated with Erastin (10 μM) for 10 h. Representative images of cell state observed under the microscope are shown on the right. Scale bars: 5 μm. **B** PHB2 expression affects the sensitivity of cells to ferroptosis. Blots shown are representative of two biological replicates. Data represent the mean ± SD of three biological replicates. Statistical analyses were performed by two-way ANOVA with Sidak’s multiple comparisons test: **p < 0.01. **C** Inhibition of PHB2 by shRNA or YL-939 increased the expression level of ferritin protein. Blots shown are representative of three biological replicates. **D** Effects of PHB2 knockdown on ferroptosis key genes. Data represent the mean ± SD of three biological independent experiments. Statistical analyses were performed by two-way ANOVA with Sidak’s multiple comparisons test. Specific *p*-values are indicated in the figure. **E** PHB2 knockdown decreased the iron content determined by Iron assay kit. Data represent the mean ± SD of three biological replicates. Statistical analyses were performed by two-tailed unpaired Student’s *t*-test. Specific *p*-values are indicated in the figure. **F** PHB2 knockdown decreased the iron content examined with fluorescent probes. Cells were stained with Fe^2+^ probe (red) and Dapi (blue). Representative images of two biological replicates are shown. Scale bars: 10 μm. Source data are provided as a Source data file.

We then investigated the engagement of PHB2 in the ferroptosis regulation. As shown in Fig. 6A, *PHB2* knockdown by small interfering RNA (siRNA) substantially inhibited erastin-induced ferroptosis. Further, to explore the influence of PHB2 on the ferroptosis sensitivity, different concentrations of erastin were used to induce ferroptosis. *PHB2* knockdown led to a significant resistance to ferroptosis. While restoring PHB2 expression in *PHB2* knockdown cells evidently recovered the ferroptosis sensitivity (Fig. 6B).

To explore the mechanism underlying PHB2 regulating ferroptosis, key proteins in the four known signaling pathways involved in the cellular antioxidant system and ferroptosis regulation were detected upon PHB2 inhibition. *PHB2* knockdown or inhibition by YL-939 had no impact on the expression of key components of the GPX4 signal pathway, such as SLC7A11 and GPX4 (Fig. 6C). *PHB2* knockdown also did not impact the mRNA expression of *FSP1*, *GCH1* and *DHODH* (Fig. 6D), which are core components for the other three pathways, respectively. All these results indicate that PHB2 does not participate in the regulation of ferroptosis through the four known signaling pathways.

**Fig. 7:**
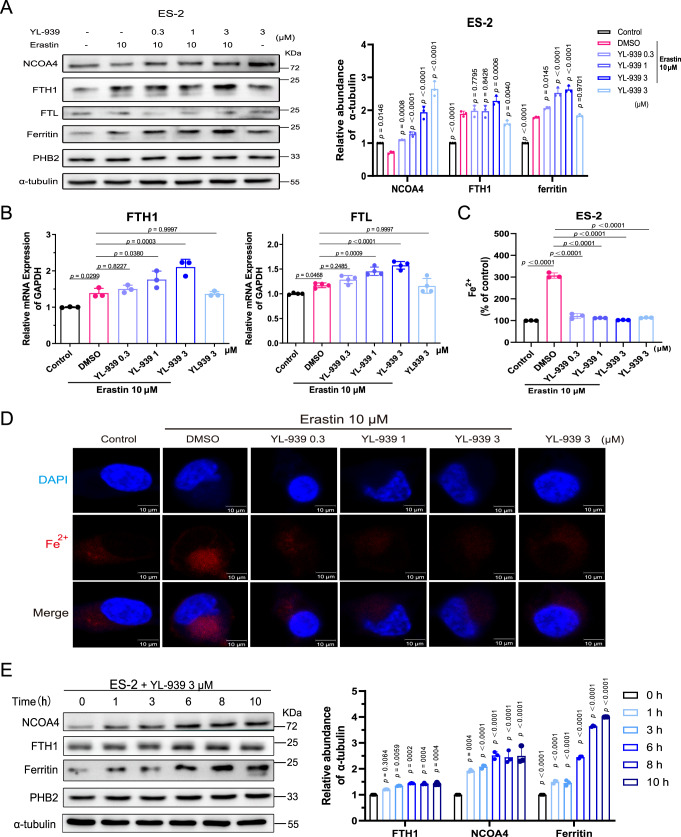
The effects of YL-939 treatment on ferritin expression and iron level. **A** YL-939 treatment improved ferritin protein expression in a concentration-dependent manner. Blots shown are representative of three biological replicates. The quantification of immunoblots was carried out. Data represent the mean ± SD of three biological replicates. Statistical analyses were performed by two-way ANOVA with Dunnett’s multiple comparisons test. Specific *p*-values are indicated in the figure. **B** YL-939 treatment improved the mRNA expression of *FTH1* and *FTL*. Data represent the mean ± SD of three biological independent experiments. Statistical analyses were performed by Owo-way ANOVA with Dunnett’s multiple comparisons test. Specific *p*-values are indicated in the figure. **C** YL-939 treatment decreased the iron content in a concentration-dependent manner. Data represent the mean ± SD of three biological replicates. Statistical analyses were performed by Owo-way ANOVA with Dunnett’s multiple comparisons test. Specific *p*-values are indicated in the figure. **D** YL-939 treatment decreased the iron content examined with fluorescent probes. Cells were stained with Fe^2+^ probe (red) and Dapi (blue). Representative images of two biological replicates are shown. Scale bars: 10 μm. **E** The effect of YL-939 treatment on NCOA4 protein level was determined by western blot. Blots shown are representative of three biological replicates. The quantification of immunoblots was carried out. Data are presented as mean ± SD. Statistical analyses were performed by two-way ANOVA with Sidak’s multiple comparisons test. Specific *p*-values are indicated in the figure. Source data are provided as a Source data file.

We next examined whether *PHB2* knockdown affects iron metabolism in ferroptosis. Compared with control cells (ES-2-shNC), PHB2 knockdown markedly up-regulated the expression of ferritin, but had no impact on TFR1, DMT1, and FPN ((Fig. 6C). Similar to the western blot experiments, *PHB2* knockdown evidently elevated the mRNA expression of ferritin heavy chain (*FTH1*) and ferritin light chain (*FTL*), which are two components of ferritin, but did not affect the mRNA expression of *TFR1* and *DMT1* (Fig. 6D). Consistent with the ferritin up-regulation, *PHB2* knockdown significantly reduced the content of iron (Fig. 6E, F). We then examined whether YL-939 treatment had the same effect as *PHB2* knockdown on ferritin and the intracellular iron level. Our results showed that, similar to the effect of *PHB2* knockdown, YL-939 treatment dose-dependently up-regulated ferritin protein (Fig. 7A) and the mRNA expression of *FTH1* and *FTL* (Fig. 7B). It also dose-dependently reduced the intracellular iron level that was elevated by erastin treatment (Fig. 7C, D).

Because ferritinophagy plays a critical role in regulating ferritin as well as the intracellular iron level, we further explored the effect of *PHB2* knockdown or YL-939 treatment on ferritinophagy. Previous studies have shown that nuclear receptor coactivator 4 (NCOA4) is the key molecule to cause ferritinophagy. Mechanically, NCOA4 is a specific cargo receptor for ferritin, and is responsible for delivering ferritin to lysosomes for degrading (ferritinophagy), and thus releasing iron and promoting ferroptosis^42^. Therefore, we examined the effect of YL-939 treatment or *PHB2* knockdown on the NCOA4 levels. Western blot assays showed that the level of NCOA4 in the erastin-treated group decreased (Figs. 6C and 7A), which was consistent with literature^43,44^. The decrease of NCOA4 level was demonstrated to be achieved through autophagy (ferritinophagy), specifically autophagosomes/lysosomes^42^. Notably, we observed an increase of ferritin level, in line with the increases of FTH1 levels, during ferroptosis (Fig. 7A, B). A reasonable explanation might be that the increase of cellular iron level during ferroptosis will induce the expression of endogenous ferritin^44^. YL-939 treatment substantially increased the NCOA4 and ferritin (or indicated by FTH1) levels in erastin-treated cells in a concentration-dependent manner (Fig. 7A). *PHB2* knockdown showed similar effects as YL-939 treatment. The same effects of *PHB2* knockdown or YL-939 treatment were also observed in cells without erastin treatment (Figs. 6C and 7E). All these results indicated that *PHB2* knockdown or YL-939 treatment blocked autophagosomes/lysosomes, and hence inhibited ferritinophagy.

### In vivo effect of YL-939

**Fig. 8:**
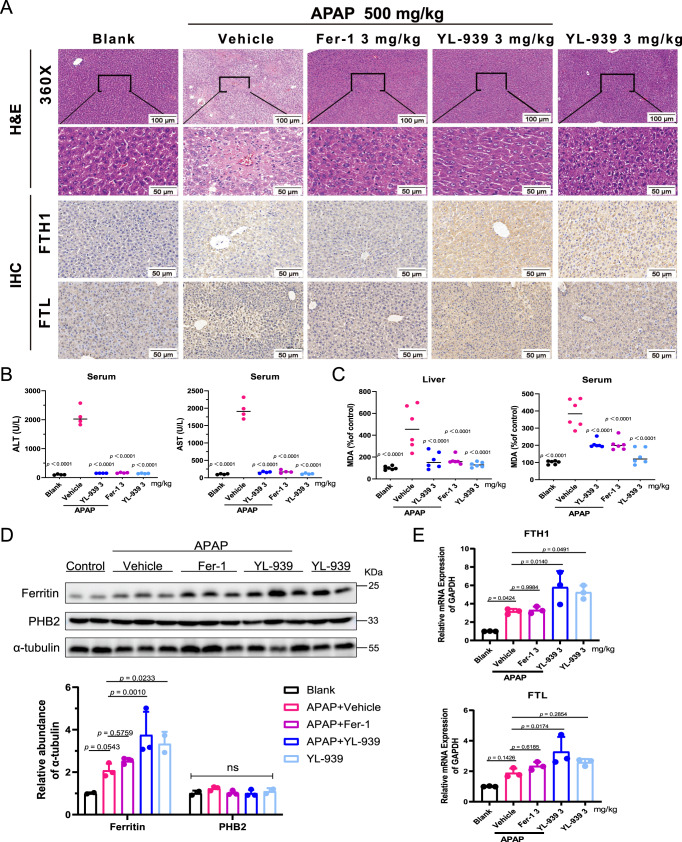
YL-939 ameliorated liver damage in an acetaminophen (APAP)-induced acute liver injury model. Liver and serum samples were obtained from mice injected with vehicle or APAP (500 mg kg^−1^) 6 h after injection. Male mice treated with YL-939 (3 mg kg^−1^), Fer-1 (3 mg kg^−1^) or vehicle 2 h prior to injection (*n* = 8 mice). **A** Representative images (*n* = 3) of H&E staining and immunohistochemistry (FTH1 and FTL) of liver in acetaminophen (APAP)-induced liver injury. Scale bars: 50 μm or 100 μm. **B** Serum AST and ALT levels were quantified. Data represent the mean ± SD from four mice/group. Statistical analyses were performed by one-way ANOVA with Sidak’s multiple comparisons test. Specific *p*-values are indicated in the figure. **C** Serum and hepatic MDA levels were assessed. Data represent the mean ± SD from six mice/group. Statistical analyses were performed by one-way ANOVA with Dunnett’s multiple comparisons test. Specific *p*-values are indicated in the figure. ns: no significance (*p* > 0.05). **D** Hepatic ferritin protein expression was determined by western blot and quantification of immunoblots was carried out (*n* = 2 mice in control group and YL-939 alone group, *n* = 3 mice in other groups). Data represent the mean ± SD. Statistical analyses were performed by two-way ANOVA with Dunnett’s multiple comparisons test. Specific *p*-values are indicated in the figure. **E** Hepatic *FTH1* and *FTL* mRNA expression was assessed. Data represent the mean ± SD from three mice/group. Statistical analyses were performed by one-way ANOVA with Dunnett’s multiple comparisons test. Specific *p*-values are indicated in the figure. Data were obtained from two independent experiments. Source data are provided as a Source data file.

Acetaminophen (APAP) induced acute liver injury model was used to evaluate the in vivo effect of YL-939. APAP is a widely used drug to treat colds and fever, arthralgia, neuralgia and migraine, cancer pain, and postoperative pain relief^45^. APAP overdose is a common cause of drug-induced liver injury (DILI)^46^. Ferroptosis has been recently implicated in APAP-induced liver injury^15,47^. As displayed in Fig. 8A, intraperitoneal (i.p.) administration of YL-939 remarkably inhibited the cell death and inflammatory infiltration in the liver tissues of male C57BL/J6 mice that received APAP. It also substantially lowered the serum levels of aspartate aminotransferase (AST) and alanine aminotransferase (ALT) (Fig. 8B), which were elevated due to APAP injection, implying that the hepatotoxicity was suppressed. Meanwhile, the lipid peroxidation products in liver tissues and serum indicated by MDA were effectively decreased following the administration of YL-939 (Fig. 8C). Fer-1, which is a radical-trapping antioxidant and was used a positive control, also showed similar protective effects. Moreover, western blot assays showed that YL-939 substantially increased the protein expression of hepatic ferritin in liver tissues (with or without APAP treatment) (Fig. 8D). This result was further confirmed by immunohistochemical staining (Fig. 8A) and qPCR of *FTH1* and *FTL* (Fig. 8E). In addition, protein and mRNA expression of hepatic ferritin were increased in a time-dependent manner after YL-939 injection without APAP injection (Supplementary Fig. 9).

## Discussion

Ferroptosis has been implicated in the pathological cell death associated with many diseases, including neurodegenerative diseases^17^, cancer cell death^48^, acute renal failure^16^, drug-induced hepatotoxicity^15,47^, and brain and heart ischemia/reperfusion injury^12,14^. Understanding the regulation mechanism and developing effective intervention agents of ferroptosis have emerged as a hot topic. In this account, we discovered a new potent non-classical small molecule ferroptosis inhibitor, YL-939. This compound did not show impact on the currently known signal pathways regulating ferroptosis. We then carried out affinity-based proteome profiling coupled with bioimaging to identify the biological target of YL-939, which revealed that PHB2 is the direct binding protein of YL-939.

PHB2 is a member of the prohibitin domain family and is widely distributed in different cellular compartments such as the mitochondria, nucleus, and cell membrane^49^. Previous studies have shown that PHB2 is involved in the regulation of many biological processes, such as metabolism^50^, mitochondrial function^51,52^, and survival^53^. PHB2 is also considered as the etiology of many diseases, including cancer^54^, inflammation^55^, cardiovascular^39^, and neurodegenerative diseases^56,57^. Up to now, there is only one report^58^ that linked PHB2 with ferroptosis, in which PHB2 was conjectured to participate the regulation of H_2_O_2_-induced cellular ferroptosis by reducing aquaporin expression. However, according to the Cancer Cell Line Encyclopedia (CCLE), the aquaporin proteins are barely expressed in ES-2 cells. Meanwhile the mRNAs of aquaporin genes were also not detected in our qRT-PCR experiment on ES-2 cells. We thus excluded the possibility that PHB2 regulated ferroptosis by affecting aquaporin. Our experimental results showed that PHB2 participated in the ferroptosis regulation not through the known signaling pathways, but by regulating the ferritin expression and ferritinophagy to affect the ferritin level and hence the iron content.

In this study, the APAP-induced liver injury model was adopted to evaluate the in vivo effect of our compound. This model was chosen because it is a classical animal model of ferroptosis and easy to operate. In addition, chronic liver diseases such as alcoholic liver diseases or non-alcoholic steatohepatitis (NASH) are known to be related to ferroptosis^59,60^. These chronic liver diseases are accompanied with iron overload, and patients often demonstrate high serum ferritin levels. In principle, small molecule compounds targeting PHB2 could be a potential therapeutic option for these chronic liver diseases, and other ferroptosis-related diseases as well. Even though, further in-depth investigations for the therapeutic effects on these disease models are still needed in later studies.

Conclusively, we discovered and characterized a new non-classical ferroptosis inhibitor, YL-939. The biological target of YL-939 was identified to be PHB2. We then for the first time revealed that PHB2 participates the regulation of ferroptosis by modulating both the ferritin expression and ferritinophagy, and hence the iron content. Nevertheless, the detailed regulation mechanism of the PHB2-ferritin-iron axis remains to be explored in the future. Overall, the pharmacological inhibition of PHB2 might represent an attractive approach for the treatment of ferroptosis-associated with disorders.

## Methods

### Cell culture

The ES-2 cell line was purchased from the National Collection of Authenticated Cell Cultures (No. TCHu111). The other cell lines used in this investigation were purchased from the American Type Culture Collection (ATCC). HT29, L02, LX-2, Beas-2b, HUVEC cells were cultured in DMEM (Gibco) medium supplemented with 10% fetal bovine serum, 100 U/mL penicillin, and 100 U/mL streptomycin. A549, THP-1, Arpe, hTERT-HPNE and ES-2 cells were cultured in RPMI-1640 (Gibco) medium supplemented with 10% fetal bovine serum, 100 U/mL penicillin, and 100 U/mL streptomycin. HT1080 were cultured in MEM (Gibco) medium supplemented with 10% fetal bovine serum, 100 U/mL penicillin, and 100 U/mL streptomycin. All cell incubations were performed at 37 °C under 5% CO_2_. All cells were negative for mycoplasma, and these cell lines are not among those commonly misidentified by International Cell Line Authentication Committee (ICLAC).

### Screening of ferroptosis inhibitors

ES-2 cells were selected to develop the screening model for ferroptosis inhibitors. In this model, 10 μM erastin and test compounds with specific concentrations were added at the same time. The initial concentration of test compounds was 3 μM. Forty-eight hours later, the cell viability was detected by MTT assay.

### Cell viability assays

MTT assay and CCK-8 assay were performed for the evaluation of cell viability. The cells were seeded in a 96-well plate at a density of 2000–5000 cells per well. Overnight, test compounds were added for 48 h. 20 μL of MTT (5 mg mL^−1^ in saline, Sigma) was then added per well. 2–4 h later, 50 μL SDS (10% in water with 0.1% HCl) was added per well or 10 μL of CCK-8 (MCE) was added per well for 2–4 h. Finally, the absorbance was obtained from a multiscan spectrum reader (BMG labtech) at 570 nm. The cell survival rate was calculated subsequently.

### Cytocalcein violet 450/7-AAD assay

ES-2 and HT1080 cells were separately inoculated into 12-well plates. After treatment with test compounds for 10 h, cells were washed with PBS and cytocalcein violet 450 were added to the cells at a final concentration of 5 μM. Incubated at 37 °C for 20–30 min, and cells were gently cleaned with PBS once. Then, 7-AAD dye was added to the cells and incubated at 37 °C for 20–30 min. Finally, the cells were observed and photographed under a fluorescence microscope (Olympus).

### Transmission electron microscope

The cells at logarithmic growth stage were inoculated into 60 mm petri dishes. After treatment with compounds for 10 h, cells were collected by trypsin digestion, 0.5% glutaraldehyde fixative was slowly added along the wall. The cells were suspended and placed at 4 °C for 10 min. Then transferred to a 1.5 mL tube, and after high-speed centrifugation, the supernatant was discarded and 3% glutaraldehyde solution was added to fix the cells. Then, the cells were fixed with 1% osmium tetroxide, and after infiltration with series proportions of dehydrating agent and epoxy resin (Epon812), embedding and making 50 mm sections, the samples were stained with uranium acetate for 10–15 min, and then stained with lead citrate for 1–2 min at room temperature. Finally, the cells were visualized with transmission electron microscopy (JEM-1400PLUS)^61^.

### Detection of intracellular ROS

The cells at logarithmic growth stage were inoculated into 6-well plates at a density of 2 × 10^5^ cells per well. After treatment with compounds for 10 h, the cells were harvested by EDTA-free trypsin, re-suspended in 0.5 mL hanks balanced salt solution (HBSS, Gibco). Then, lipid peroxidation in situ, cytoplasmic ROS, and mitochondrial ROS were, respectively, indicated using the fluorescent lipid peroxidation sensor BODIPY 581/591 C11 (2 μM, ThermoFisher Scientific), H_2_DCFDA (5 μM, ThermoFisher Scientific) and MitoSOX (5 μM, ThermoFisher Scientific); and incubated for 10 min at 37 °C. Cells were collected by centrifugation and then suspended in 0.5 mL HBSS buffer, and a minimum of 10,000 cells were analyzed using a flow cytometer (Agilent NovoSampler Pro).

### Determination of intracellular MDA levels

The level of lipid peroxidation was assessed using an MDA detection kit (Beyotime) according to the manufacturer’s instructions. In brief, the cells at logarithmic growth stage were inoculated into 6-well plates at a density of 2 × 10^5^ cells per well. After treatment with test compounds for 10 h, cells were washed with cold phosphate buffered saline (PBS) buffer, added cell lysis buffer for 10 min in ice, and then cell supernatant was collected by centrifugation. Finally, added 0.2 mL of MDA working solution (0.37% TBA plus antioxidants from the kit) to the supernatant and heat at 100 °C for 15 min. Then, the samples were cooled in a water bath to room temperature and centrifuged at room temperature at 1000 × *g* for 10 min. Finally, 0.2 mL of supernatant was added to the clear 96-well plate and the absorbance was measured at 532 nm using a CLARIOstar microplate reader (BMG Labtech, v5.61).

### Determination of intracellular GSH levels

The cells at logarithmic growth stage were inoculated into 6-well plates at a density of 2 × 10^5^ cells per well. After treatment with test compounds for 10 h, cells were washed with PBS buffer, added triploid protein removal reagent. Then, the samples were gathered by repeated freeze-thaw. Finally, the amount of glutathione was evaluated using a GSH detection kit (Beyotime) according to the manufacturer’s instructions^62^.

### Western blot analysis

Cells were treated with test compounds for 10 h, then washed twice with PBS buffer and lysed in NP-40 lysis buffer (1% NP-40, 150 mM NaCl, 0.25% deoxycholate, 1 mM EGTA, 1 mM NaF, 50 mM Tris-HCl, and 2 mM sodium orthovanadate) containing 1x protease inhibitor cocktail, and 1 mM phenylmethylsulfonyl fluoride (PMSF) on ice for 15–20 min. Cell supernatants were collected by ultrasound and centrifugation. Then, all samples were normalized after determined by a bicinchoninic acid (BCA) protein quantification kit (Beyotime). And after SDS-PAGE loading buffer was added to each sample, the samples were boiling for 8 min at 100 °C. Then, the total protein was separated by 12% SDS-PAGE gel electrophoresis and transferred onto a PVDF membrane (Millipore). The membrane was sealed with 5% milk in TBS/T (TBS buffer with 0.1% tween-20) for 2–5 h, followed by incubation of primary antibodies (1:1000) at 4 °C. Next, the membrane was washed three times with TBS/T, and incubated in the corresponding secondary antibody (1:5000) at 37 °C for 1 h, and washed three times with TBS/T subsequently. Finally, the immune-band was visualized with the SuperLumia ECL plus HRP Substrate Kit (Abbkine). Antibodies used: PHB2 (#14085), VDAC1 (ET1601-20), VDAC2 (#9412), PHB1 (#2426), α-Tublin (T9026), β-actin (66009-1-Ig), SLC7A11 (ab175186), GPX4 (ab125066), TFR1 (10084-2-AP), FPN (NBP1-21502), DMT1 (20507-1-AP), Ferritin (ab75973), NCOA4 (#66849), FTH1 (#4393), FTL (A11241), HRP-conjugated Affinipure Goat Anti-Rabbit IgG(H + L) (SA00001-2), HRP-conjugated Affinipure Goat Anti-Mouse IgG(H + L) (SA00001-1).

### Real-time quantitative polymerase chain reaction (RT-qPCR)

The cells at logarithmic growth stage were inoculated into 6-well plates at a density of 2 × 10^5^ cells per well. Cells were treated with test compounds for 10 h. And total RNA was extracted from cells according to the manufacturer’s instruction (Foregene). Then, total RNA was converted into cDNA using the HiScript II Q RT SuperMix for qPCR (+gDNA wiper) (Vazyme). Subsequently, all qRT-PCR experiments were conducted strictly according to the instructions of the ChamQ Universal SYBR qPCR Master Mix (Vazyme). Finally, the quantities of the targets were normalized to the housekeeping gene (*GAPDH*). All primers of the selected genes were listed as follows: for *PHB2(h)*, forward, 5′-AAGATGCTTGGAGAAGCACTGAGCAAGAA-3′; reverse, 5′-AGCACAAGGTTGTCAGCTGTGAGATAGATA-3′; for *FTH1(h)*, forward, 5′-AGTCTTACTACTTTGACCGC-3′; reverse, 5′-AGTCTGGTTTCTTGATATCCTG-3′;for *FTL(h)*, forward, 5′-GTCAATTTGTACCTGCAGGCC-3′; reverse, 5′-CTCGGCCAATTCGCGGAA-3′; for *DMT1(h)*, forward, 5′-AGCTCCACCATGACAGGAACCT-3′; reverse, 5′-TGGCAATAGAGCGAGTCAGAACC-3′; for *TFR1(h)*, forward, 5′-ATCGGTTGGTGCCACTGAATGG-3′; reverse, 5′-ACAACAGTGGGCTGGCAGAAAC-3′; for *FSP1(h)*, forward, 5′-CATCAAAGTGCAGACGGAC-3′; reverse, 5′-TAGTCTGCTCTCAAACGCT-3′; for *DHODH(h)*, forward, 5′-CTCAGGAAGGAAACCCTAGAC-3′; reverse, 5′-CGTGACTGTTAAATCCATACCTG-3′; for *GCH1(h)*, forward, 5′-GTGTATGGTAATGCGAGGTGTACAG-3′; reverse, 5′-CTTCCCGAGTCTTTGGATCCT-3′; for *GAPDH(*h), forward, 5′-ACTGCCAACGTGTCAGTGGT-3′; reverse, 5′-GTGTCGCTGTTGAAGTCAGA-3′; for *PHB2(m)*, forward, 5′-ATCCGTGTTCACCGTGGAAG-3′; reverse, 5′-CCCGAATGTCATAGATGATGGG-3′; for *FTH1(m)*, forward, 5′-CAAGTGCGCCAGAACTACCA-3′; reverse, 5′-GCCACATCATCTCGGTCAAAA-3′; *for FTL(m)*, forward, 5′-CCATCTGACCAACCTCCGC-3′; reverse, 5′-CGCTCAAAGAGATACTCGCC-3′; for *GAPDH(m)*, forward, 5′-CAGTGGCAAAGTGGAGATTGTTG-3′; reverse, 5′-TCGCTCCTGGAAGATGGTGAT-3′.

### Synthesis of YL-939 and probe YL-939-1

YL-939 was synthesized following the reaction route outlined in Supplementary Fig. 5A. 2-Amino-3,5-dibromopyrazine reacted with 2-bromoacetophenone in 120 °C condition to form intermediate **5j**, which underwent a Suzuki-Miyaura reaction with commercially available 3,6-dihydro-2*H*-pyran-4-boronic acid pinacol ester to afford **6j**. **6j** further underwent the Suzuki-Miyaura reaction and followed by Boc-deprotection to give compound YL-939.

YL-939-1 was prepared according to the reaction route shown in Supplementary Fig. 5B. Intermediate 2-(3-(but-3-yn-1-yl)-3*H*-diazirin-3-yl)ethan-1-ol (L2) was generated by commercially available L1 after a two-step reaction. Afterward, the hydroxyl group of the L2 was oxidized to the carboxyl group to yield the linker L3. Finally, a condensation reaction between YL-939 and L3 generated probe YL-939-1.

### In-gel fluorescence

ES-2 cells were placed into 100 mm petri dishes, after treatment with erastin and YL-939-1 or DMSO for 1–10 h, cells were washed twice with PBS buffer, irradiated under an ultraviolet at 365 nm for 20 min on ice. Then, the cells were lysed with NP-40 lysis buffer (Beyotime) containing 1× protease inhibitor cocktail and 1 mM phosphatase inhibitor PMSF for 30 min. The proteins were collected by centrifugation for 15 min (16,000 × *g*, 4 °C), and then all protein concentrations were detected by a BCA protein quantification kit (Beyotime) and diluted to 1 mg/mL with NP-40 lysis buffer. Subsequently, click chemistry reaction reagents were added in following order: 50 μM TRAMA-N_3_, 1 mM ascorbic acid, 100 μM tris(3-hydroxypropyltriazolylmethyl)amine (THPTA), and 1 mM CuSO_4_. The reaction was incubated for 1–2 h at room temperature in the dark with gentle shaking prior to addition of pre-chilled acetone (−20 °C). The proteins were collected by centrifugation (16,000 × *g*, 10 min, 4 °C), and washed twice with pre-chilled methanol. Eventually, the samples were dissolved in NP-40 lysis buffer, and added SDS-PAGE loading buffer before heated for 8 min at 100 °C, and loaded onto the SDS-PAGE gel. Then, protein lanes were visualized by in-gel fluorescence scanning (Typhoon FLA 9500) and coomassie blue staining.

### Target identification experiments

The cells were treated and protein supernatants were collected as described in In-gel Fluorescence section. Subsequently, click chemistry reaction reagents were added in following order: 200 μM Biotin-azide, 1 mM ascorbic acid, 100 μM THPTA, and 1 mM CuSO_4_. The reaction was incubated for 1–2 h at room temperature in the dark with gentle shaking prior to addition of pre-chilled acetone (−20 °C). All samples were centrifuged (16,000 × *g*, 10 min, 4 °C), and washed twice with pre-chilled methanol. Eventually, the samples were dissolved in 1% SDS (in PBS) with sonication, and incubated with 0.1 mL streptavidin beads with gently mixing at 4 °C overnight. Eventually, the beads were rinsed with the following solutions in sequence: 1% SDS (in PBS, 3 × 1 mL × 5 min), 0.1% SDS (in PBS, 3 × 1 mL × 5 min), and PBS (3 × 1 mL × 5 min). The samples were mixed with SDS-PAGE loading buffer before heating for 8 min at 100 °C. After enriching the protein on the streptavidin-coated magnetic beads, the beads were collected by centrifugation (1000 × *g*, 3 min at room temperature). Subsequently, the samples were re-suspended in 6 M urea (in PBS), added 100 mM DTT in 25 mM ammonium bicarbonate (ABB) in PBS, and incubated at 37 °C with shaking for 30 min, followed by washing once with PBS for 5 min. For alkylation, the samples were re-suspended in 6 M urea (in PBS), added 400 mM IAA in 25 mM ABB (in PBS), and incubated at 37 °C with shaking for 30 min in the dark environment, followed by washing once with PBS for 5 min. For the digestion, 2 M urea (in PBS), 1 mM CaCl_2_ in 50 mM ABB, and 1 μg trypsin were added to the beads, the mixture solutions were incubated at 37 °C overnight. Finally, the reaction was quenched by adding trifluoroacetic acid (TFA), and the supernatants were collected by centrifugation (1000 × *g*, 2 min at room temperature). After collecting the supernatants containing the digested peptides, samples were desalted with Waters C18 micro columns (ThermoFisher Scientific), and dried by vacuum centrifugation. The samples were reconstituted in 0.1% formic acid before analyzing on Easy-nLC 1000 system coupled to a Q Exactive HF (ThermoFisher Scientific). The raw data were processed and searched with MaxQuant 1.5.4.1 with MS tolerance of 4.5 ppm, and MS/MS tolerance of 20 ppm. The UniProt human protein database (release 2016_07, 70630 sequences) and database for proteomics contaminants from MaxQuant were used for database searches. Reversed database searches were used to evaluate false discovery rate (FDR) of peptide and protein identifications.

### Drug affinity responsive target stability (DARTS)

ES-2 cells were placed into 60 mm petri dishes, after treatment with test compounds for 8 h; cells were washed twice with PBS buffer, and cells were lysed in 200 μL NP-40 lysis buffer containing 1× protease inhibitor cocktail, and 1 mM PMSF on ice for 20 min. And each soluble protein was collected and normalized to 2 mg mL^−1^. Divided the protein into 50 μL per tube, added pronase (Roche) at a ratio of 20:1 and incubated at 25 °C for 5 min, and then added 20 times of proteasome inhibitor cocktail on ice to stop the enzymatic hydrolysis reaction for 10 min. Finally, the proteins were mixed with SDS-PAGE loading buffer and heated at 100 °C for 8 min. The results were performed by western blotting assay as described in Western blot analysis.

### Cellular imaging study

In order to explore whether the fluorescent label TRAMA-N_3_ could enter the cells and bind to the probe YL-939-1, we performed cellular imaging and immunofluorescence. ES-2 cells were seeded into a 24-well plate covered with cell slides. After treatment with probe YL-939-1 for 3 h, cells were washed twice with PBS buffer, and then irradiated under an ultraviolet at 365 nm for 20 min on ice. Added 0.5 mL 4% paraformaldehyde (PFA) to fix the cells, and incubated for 30 min at room temperature. The cells were washed twice with PBS buffer followed by adding 0.5 mL of 0.1% Trinton X-100 (in PBS). Subsequently, added the mixed click reaction agents to the cells, incubated for 2 h with gently mixing at room temperature. Washed the cells triple with PBS/T (PBS buffer with 0.1% tween-20), The cells were blocked with 3% bovine serum albumin (BSA) in PBS/T for 0.5 h, followed by incubation of primary antibodies (1:200) at 4 °C overnight. Next, the membrane was washed three times with PBS/T, and incubated in the corresponding fluorescent secondary antibody (1:200) at room temperature for 1 h, and washed three times with PBS/T subsequently. Finally, the nuclei were stained with Hoechest 33342 (5 mg mL^−1^, KeyGEN) for 10 min at 37 °C. The cell slides were transferred to a glass slide and observed under a confocal microscope (Zeiss LSM 880).

### siRNA transfection

ES-2 cells were transfected for 24 h with 50 nM siPHB2 and lipofectamine 2000 (ThermoFisher Scientific), positive control siGAPDH or fluorescent negative control siRNA, transfection efficiency was verified by western blot experiment. The cells were treated with compounds for 24 h, and cell viability were detected using CCK-8 assay, trypan blue staining or observed on a microscope. PHB2 siRNA#1 sequence used was 5′-CUACAGAUGGUGAAUAUCUTT-3′; PHB2 siRNA#2 sequence used was 5′-CUGUAGAAGCCAAACAAGUTT-3′; PHB2 siRNA#3 sequence used was 5′-CACAGAAUCGUAUCUAUCUTT-3′.

### Lentiviral transfection

The shRNA sequences for transfection were as follows: shPHB2#1, 5ʹ-CCAGAATATCTCCAAGACGAT-3ʹ; shPHB2#2, 5ʹ-AAGAACCCTGGCTACATCAAA-3′. The two sequences were respectively cloned into PTSB-SH-copGFP-2A-PURO vector. For overexpression of PHB2, the PHB2 sequence was inserted into the pLVX-IRES-Zsgreen1 vector between 5′ EcoRI and 3′ XhoI. Then, the plasmids were transfected using TransEasy transfection reagent (Foregene) following the manufacturer’s instructions.

### Fe^2+^ detection

Cells were seeded into a 6-well plate or on 24-well cell slides, treated with compounds for 10 h. The intracellular iron was detected with a Fe^2+^ detection kit (ThermoFisher Scientific) or a fluorescent imaging probe (BioTracker 575 Red Fe2+ Dye, Sigma-Aldrich) according to the manufacturer’s instructions.

### Protein preparation

The DNA sequence of encoding 1–194 amino acid human PHB2 with a 6× His tag was amplified and inserted into a pET-28a(+) vector through 5′NcoRI and 3’XhoI restriction sites. PHB2^1–194^ and its variants were expressed in *E.coli BL21 (DE3)* cells at 37 °C. Recombinant protein expression was induced by the addition of 0.5 mM isopropyl-β-D-1-thiogalactopyranoside (IPTG) and further incubated at 20 °C. The next day, cells were collected by high-speed centrifugation (4500 × *g*, 15 min), and the pellets were re-suspended in lysis buffer (50 mM Hepes, 500 mM NaCl, 0.5 mM tris(2-carboxyethyl)-phosphine hydrochloride (TCEP.HCl, ThermoFisher Scientific), 1 mM PMSF and 10% glycerol, pH 7.5). The bacteria were centrifuged at 18,000 × *g* for 45 min, and the supernatant was transferred to the Ni-NTA Column. The PHB2^1–194^ protein was eluted by elution buffer (20 mM Hepes, 0.25 M NaCl, and 300 mM imidazole, pH 7.5). Then the protein was loaded onto an S column (GE-Healthcare). After elution with a NaCl gradient, the homogeneity of the protein sample was evaluated using coomassie blue-stained SDS-polyacrylamide gels. The eluted protein was further purified by size exclusion chromatography using a Superdex 200 10/300 GL column (GE Healthcare) in buffer (20 mM Hepes, 150 mM NaCl, pH 7.5). Fractions corresponding to monomeric peaks were pooled together. The quality of the protein purification was validated by SDS-PAGE analysis. The variants were purified similarly as described above.

### Differential scanning fluorimetry assay (DSF)

DSF experiments were performed using Bio-Rad CFX ConnectTM Real-Time System. First, 9.8 μL protein and SYPRO Orange mixture were added per well, and then mixed 0.2 μL compound. The final assay volume is 10 μL. The final assay system contained 20 mM Hepes, pH 7.5 150 mm NaCl, 10 μM protein, and 200 μM compound. Thermal denaturation was achieved by a temperature ramp from 25 °C to 95 °C (1 °C per minute).

### Surface plasmon resonance (SPR)

PHB2^1–194^ was immobilized to 6000–9000 response units on flow cells 3 and 4 of Biacore series S CM5 sensor chip (GE Healthcare) on a Biacore S200 instrument (GE Healthcare) after predilution to HBS buffer (10 mM Hepes, 150 mM NaCl, 3 mM EDTA, 0.05% w/v Surfactant P20, pH7.4). After changing to assay buffer (137 mM NaCl, 2.7 mM KCl, 10 mM Na_2_HPO_4_, 2 mM KH_2_PO_4_, 0.05% w/v Surfactant P20, pH 7.2–7.4), the sensor chip was predilution before used for K_d_ measurements. The flow rate was 10 μL min^−1^, and the contact time and dissociation time were 120 s and 100 s, respectively. Data reported in this study are means of at least two independent experiments ± standard deviations.

### Acute liver injury

All procedures related to animal handling, care, and treatment in efficacy studies were performed according to the guidelines approved by the Institute Animal Care and Use Committee (IACUC) of West China Hospital, Sichuan University (20211063 A). It has been reported that female mice were resistant to the hepatotoxic effects of APAP than male mice^63,64^. Considering this, C57BL/6 male mice (age: 4–5 weeks) were used in the APAP-induced hepatic injury model in this study. The mice were kept in cages with individual ventilation under 65% humidity and an ambient temperature of 21–23 °C and a 12 h–12 h day-night cycle for housing and husbandry.

Male C57BL/6J mice, 4–5 weeks of age were given a single intraperitoneal injection of vehicle or compound YL-939 dissolved in 5% DMSO plus 3% HS-15 Solutol plus 92% saline, followed by intraperitoneal injection of 500 mg kg^−1^ acetaminophen dissolved in saline 2 h later. After 6 h, the mouse serum was isolated for detection of AST and ALT with an automatic biochemical analyzer (Roche) according to the manufacturer’s protocols. The livers were harvested, a part of which was quick frozen using liquid nitrogen for the detection of protein and mRNA expression of hepatic ferritin, and the other part of which was fixed in 4% paraformaldehyde for hematoxylin and eosin (H&E) staining and immunohistochemistry (IHC).

### Histology and immunohistochemistry

Histology and immunohistochemistry were performed as described^65^. The isolated liver tissues were fixed in 4% paraformaldehyde at room temperature for 24 h. After embedding in paraffin, sections of 4-μm-thick sections were stained with hematoxylin and eosin according to the instructions of manufacturer. Immunohistochemical analysis was performed using the following primary antibody: anti-FTH1 (CST) and anti-FTL (ABclonal). The stained sections were scanned using a Pannoramic MIDI II scanner (3DHISTECH).

### Data analysis

All results were expressed as mean ± standard deviation (SD), and statistical analysis were made by means of one-way ANOVA test, two-way ANOVA test and t test with GraphPad Prism 8 software and Microsoft Excel 2016, *p* < 0.05 was considered as significant.

### Reporting summary

Further information on research design is available in the Nature Portfolio Reporting Summary linked to this article.

## Supplementary information

Supplementary Information

Reporting Summary

## Data Availability

The data that supports the findings of this study are available within the article and its Supplementary. The protein information used in this study is available in the UniProt human protein database (release 2016_07, 70630 sequences) under https://www.uniprot.org/. The Source data of all figures within the article and the Supplementary are provided as a Source data file. Source data are provided with this paper.

## Source data

Source Data

## References

[1] Dixon SJ (2012). Ferroptosis: an iron-dependent form of nonapoptotic cell death. Cell.

[2] Yang WS (2014). Regulation of ferroptotic cancer cell death by GPX4. Cell.

[3] Bersuker K (2019). The CoQ oxidoreductase FSP1 acts parallel to GPX4 to inhibit ferroptosis. Nature.

[4] Doll S (2019). FSP1 is a glutathione-independent ferroptosis suppressor. Nature.

[5] Kraft VAN (2020). GTP cyclohydrolase 1/tetrahydrobiopterin counteract ferroptosis through lipid remodeling. ACS Cent. Sci..

[6] Mao C (2021). DHODH-mediated ferroptosis defence is a targetable vulnerability in cancer. Nature.

[7] Kagan VE (2017). Oxidized arachidonic and adrenic PEs navigate cells to ferroptosis. Nat. Chem. Biol..

[8] Vogt AS (2021). On iron metabolism and its regulation. Int. J. Mol. Sci..

[9] Donovan A (2005). The iron exporter ferroportin/Slc40a1 is essential for iron homeostasis. Cell Metab..

[10] Worwood M (1990). Ferritin. Blood Rev..

[11] Hassannia B, Vandenabeele P, Vanden Berghe T (2019). Targeting ferroptosis to iron out cancer. Cancer Cell.

[12] Tuo QZ (2017). Tau-mediated iron export prevents ferroptotic damage after ischemic stroke. Mol. Psychiatry.

[13] Linkermann A (2014). Synchronized renal tubular cell death involves ferroptosis. Proc. Natl Acad. Sci. USA.

[14] Fang X (2019). Ferroptosis as a target for protection against cardiomyopathy. Proc. Natl Acad. Sci. USA.

[15] Yamada N (2020). Ferroptosis driven by radical oxidation of n-6 polyunsaturated fatty acids mediates acetaminophen-induced acute liver failure. Cell Death Dis..

[16] Friedmann Angeli JP (2014). Inactivation of the ferroptosis regulator Gpx4 triggers acute renal failure in mice. Nat. Cell Biol..

[17] Skouta R (2014). Ferrostatins inhibit oxidative lipid damage and cell death in diverse disease models. J. Am. Chem. Soc..

[18] Hambright WS, Fonseca RS, Chen L, Na R, Ran Q (2017). Ablation of ferroptosis regulator glutathione peroxidase 4 in forebrain neurons promotes cognitive impairment and neurodegeneration. Redox Biol..

[19] Feng Y, Madungwe NB, Imam Aliagan AD, Tombo N, Bopassa JC (2019). Liproxstatin-1 protects the mouse myocardium against ischemia/reperfusion injury by decreasing VDAC1 levels and restoring GPX4 levels. Biochem. Bioph. Res. Co..

[20] Zhang Y (2019). Ferroptosis inhibitor SRS 16-86 attenuates ferroptosis and promotes functional recovery in contusion spinal cord injury. Brain Res..

[21] Xu Y, Liang M, Ugbolue UC, Fekete G, Gu Y (2022). Effect of physical exercise under different intensity and antioxidative supplementation for plasma superoxide dismutase in healthy adults: systematic review and network meta-analysis. Front. Physiol..

[22] Xian D, Lai R, Song J, Xiong X, Zhong J (2019). Emerging perspective: role of increased ROS and redox imbalance in skin carcinogenesis. Oxid. Med. Cell Longev..

[23] Davalli P, Mitic T, Caporali A, Lauriola A, D’Arca D (2016). ROS, cell senescence, and novel molecular mechanisms in aging and age-related diseases. Oxid. Med. Cell Longev..

[24] Hentze MW, Muckenthaler MU, Galy B, Camaschella C (2010). Two to tango: regulation of mammalian iron metabolism. Cell.

[25] Benoit SL, Maier RJ (2021). The nickel-chelator dimethylglyoxime inhibits human amyloid beta peptide in vitro aggregation. Sci. Rep..

[26] Kontoghiorghes, G. J. & Kontoghiorghe, C. N. Iron and chelation in biochemistry and medicine: new approaches to controlling iron metabolism and treating related diseases. *Cells* **9**, 1456 (2020). 10.3390/cells9061456 PMC7349684 32545424

[27] Yu Y (2015). The ferroptosis inducer erastin enhances sensitivity of acute myeloid leukemia cells to chemotherapeutic agents. Mol. Cell Oncol..

[28] Hao S (2017). Cysteine dioxygenase 1 mediates erastin-induced ferroptosis in human gastric cancer cells. Neoplasia.

[29] Sui X (2018). RSL3 drives ferroptosis through GPX4 inactivation and ROS production in colorectal cancer. Front. Pharm..

[30] Eaton JK (2020). Selective covalent targeting of GPX4 using masked nitrile-oxide electrophiles. Nat. Chem. Biol..

[31] Voortman J, Checinska A, Giaccone G (2007). The proteasomal and apoptotic phenotype determine bortezomib sensitivity of non-small cell lung cancer cells. Mol. Cancer.

[32] Xiong Y (2019). The bromodomain protein BRD4 positively regulates necroptosis via modulating MLKL expression. Cell Death Differ..

[33] Zhou Z (2020). Heat shock protein 90 inhibitors suppress pyroptosis in THP-1 cells. Biochem. J..

[34] Tsvetkov P (2022). Copper induces cell death by targeting lipoylated TCA cycle proteins. Science.

[35] Yi J (2020). Oncogenic activation of PI3K-AKT-mTOR signaling suppresses ferroptosis via SREBP-mediated lipogenesis. Proc. Natl Acad. Sci. USA.

[36] Li C (2020). LKB1-AMPK axis negatively regulates ferroptosis by inhibiting fatty acid synthesis. Signal Transduct. Target. Ther..

[37] Li Z (2013). Design and synthesis of minimalist terminal alkyne-containing diazirine photo-crosslinkers and their incorporation into kinase inhibitors for cell- and tissue-based proteome profiling. Angew. Chem. Int. Ed..

[38] Ren Y-S (2021). Drug affinity responsive target stability (DARTS) accelerated small molecules target discovery: Principles and application. Biochem. Pharmacol..

[39] Bernard Y (2011). Flavaglines alleviate doxorubicin cardiotoxicity: implication of Hsp27. PLoS ONE.

[40] Polier G (2012). The natural anticancer compounds rocaglamides inhibit the Raf-MEK-ERK pathway by targeting prohibitin 1 and 2. Chem. Biol..

[41] Perron A (2018). Small-molecule screening yields a compound that inhibits the cancer-associated transcription factor Hes1 via the PHB2 chaperone. J. Biol. Chem..

[42] Mancias JD (2014). Quantitative proteomics identifies NCOA4 as the cargo receptor mediating ferritinophagy. Nature.

[43] Chen G-Q (2020). Artemisinin compounds sensitize cancer cells to ferroptosis by regulating iron homeostasis. Cell Death Differ..

[44] Gao M (2016). Ferroptosis is an autophagic cell death process. Cell Res..

[45] Brune K, Renner B, Tiegs G (2015). Acetaminophen/paracetamol: a history of errors, failures and false decisions. Eur. J. Pain..

[46] Mossanen JC, Tacke F (2015). Acetaminophen-induced acute liver injury in mice. Lab Anim..

[47] Lorincz T, Jemnitz K, Kardon T, Mandl J, Szarka A (2015). Ferroptosis is involved in acetaminophen induced cell death. Pathol. Oncol. Res..

[48] Llabani, E. et al. Diverse compounds from pleuromutilin lead to a thioredoxin inhibitor and inducer of ferroptosis. *Nat. Chem.* **11**, 521–532 (2019). 10.1038/s41557-019-0261-6 PMC6639018 31086302

[49] Merkwirth C, Langer T (2009). Prohibitin function within mitochondria: essential roles for cell proliferation and cristae morphogenesis. BBA-Mol. Cell Res..

[50] Kasashima K, Ohta E, Kagawa Y, Endo H (2006). Mitochondrial functions and estrogen receptor-dependent nuclear translocation of pleiotropic human prohibitin 2. J. Biol. Chem..

[51] Wei Y, Chiang W-C, Sumpter R, Mishra P, Levine B (2017). Prohibitin 2 is an inner mitochondrial membrane mitophagy receptor. Cell.

[52] Yan C (2019). PHB2 (prohibitin 2) promotes PINK1-PRKN/Parkin-dependent mitophagy by the PARL-PGAM5-PINK1 axis. Autophagy.

[53] Thuaud F, Ribeiro N, Nebigil CG, Desaubry L (2013). Prohibitin ligands in cell death and survival: mode of action and therapeutic potential. Chem. Biol..

[54] Yurugi H (2017). Targeting prohibitins with chemical ligands inhibits KRAS-mediated lung tumours. Oncogene.

[55] Xu Y, Wang J, Xu W, Ding F, Ding W (2019). Prohibitin 2-mediated mitophagy attenuates renal tubular epithelial cells injury by regulating mitochondrial dysfunction and NLRP3 inflammasome activation. Am. J. Physiol. Ren. Physiol..

[56] Guyot AC (2020). A small compound targeting prohibitin with potential interest for cognitive deficit rescue in aging mice and Tau pathology treatment. Sci. Rep..

[57] Merkwirth C (2012). Loss of prohibitin membrane scaffolds impairs mitochondrial architecture and leads to tau hyperphosphorylation and neurodegeneration. PLoS Genet..

[58] Takashi Y (2020). Mitochondrial dysfunction promotes aquaporin expression that controls hydrogen peroxide permeability and ferroptosis. Free Radic. Bio. Med..

[59] Tsurusaki S (2019). Hepatic ferroptosis plays an important role as the trigger for initiating inflammation in nonalcoholic steatohepatitis. Cell Death Dis..

[60] Li X (2020). Targeting ferroptosis alleviates methionine-choline deficient (MCD)-diet induced NASH by suppressing liver lipotoxicity. Liver Int..

[61] Jung, M. K. & Mun, J. Y. Sample preparation and imaging of exosomes by transmission electron microscopy. *J. Vis. Exp.* **131**, e56482 (2018). 10.3791/56482 PMC5908436 29364263

[62] Saghy T (2019). Loss of transglutaminase 2 sensitizes for diet-induced obesity-related inflammation and insulin resistance due to enhanced macrophage c-Src signaling. Cell Death Dis..

[63] Dai G (2006). Acetaminophen metabolism does not contribute to gender difference in its hepatotoxicity in mouse. Toxicological Sci..

[64] Du K (2014). Lower susceptibility of female mice to acetaminophen hepatotoxicity: role of mitochondrial glutathione, oxidant stress and c-jun N-terminal kinase. Toxicol. Appl. Pharmacol..

[65] Tietze F (1969). Enzymic method for quantitative determination of nanogram amounts of total and oxidized glutathione: Applications to mammalian blood and other tissues. Anal. Biochem.

