# Supplementary material for: Non-classical ferroptosis inhibition by a small molecule targeting PHB2
Source: Nat Commun. 2022 Dec 3;13:7473. doi: 10.1038/s41467-022-35294-2 (PMC9719519; doi:10.1038/s41467-022-35294-2)

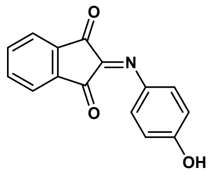

1-d-4
72.4%

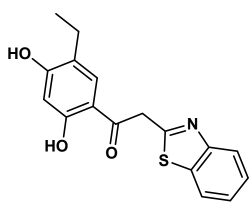

4-c-2
73.2%

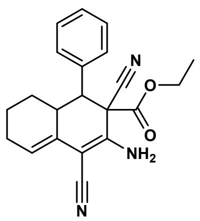

4-d-2
60.5%

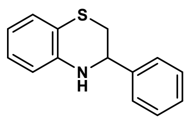

4-h-4
40.9%

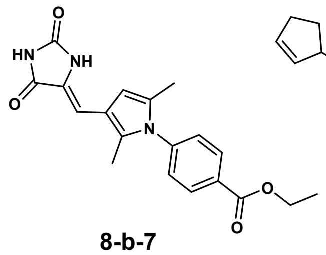

8-b-7
29.6%

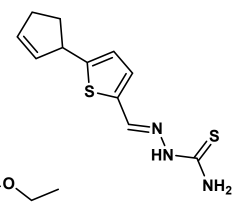

8-g-11
50.7%

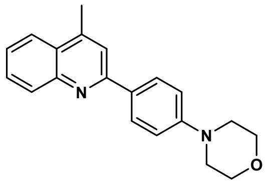

9-a-8
61.3%

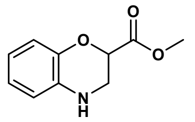

9-g-6
81.0%

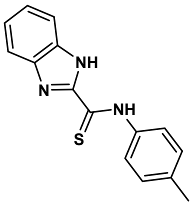

11-d-5
49.8%

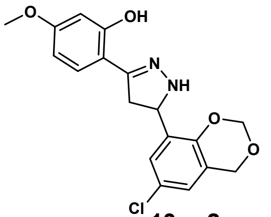

13-g-2
58.7%

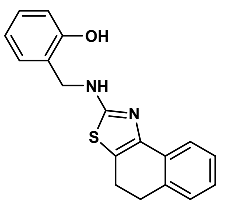

17-e-11
92.6%

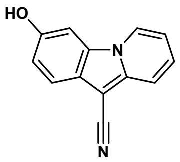

22-d-7
54.6%

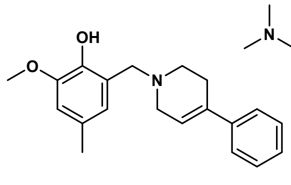

28-c-9
50.4%

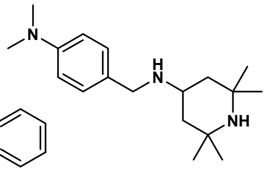

30-c-3
49.9%

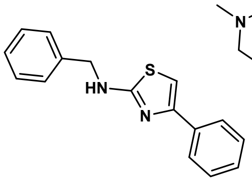

37-e-8
92.2%

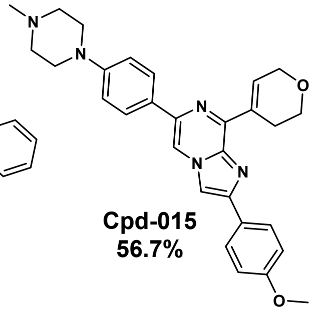

Cpd-015
56.7%

Supplementary Fig. 1: **Chemical structures and ferroptosis inhibitory rates of 16 compounds obtained in the ferroptosis inhibitor screening against 4,000 compounds.** The erastin (10 μM)-induced ES-2 cell ferroptosis model was adopted and the used concentration of compounds was 3 μM. %Ctrl values represent the survivability of ES-2 cells.

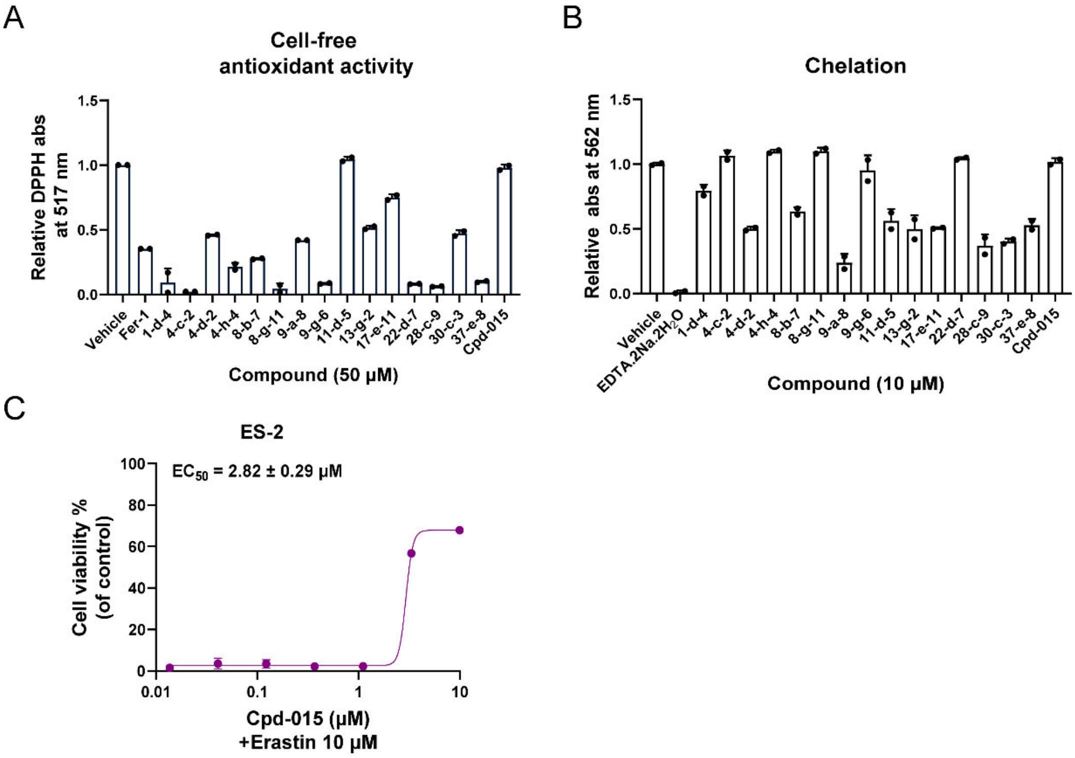

Supplementary Fig. 2: **The antioxidation activity and iron-chelation ability of the obtained ferroptosis inhibitors and the dose-dependent activity of Cpd-015.** (A) The antioxidation activity was tested by a free radical scavenging assay (DPPH). Data represent mean of two independent biological replicates. (B) The iron-chelation ability was measured by a ferrozine-based colorimetric assay. Data represent mean of two independent biological replicates. (C) The erastin-induced ES-2 cell ferroptosis model was used. MTT was adopted to measure the cell viability. Data represent mean of two independent biological replicates. Source data are provided as a Source Data file.

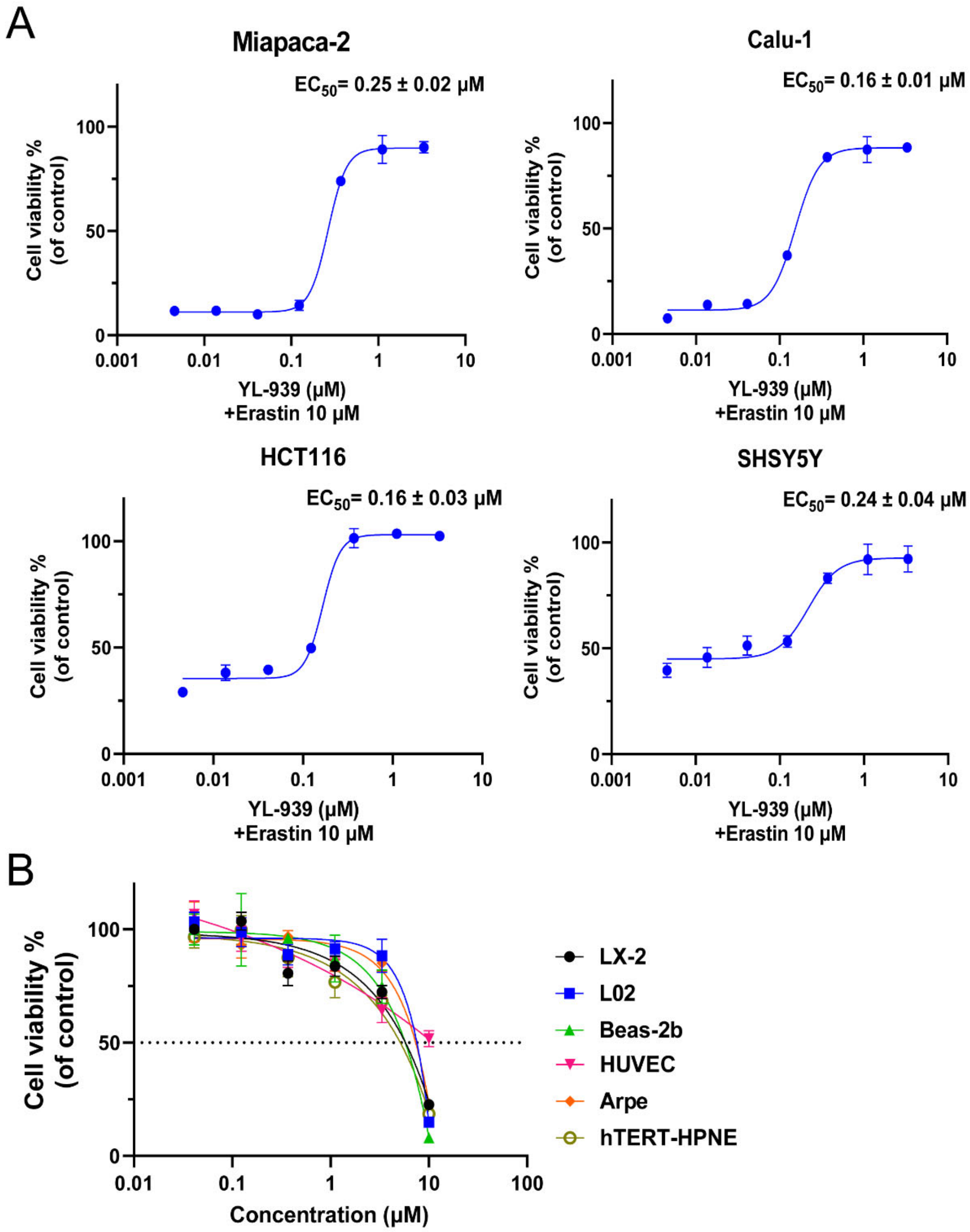

Supplementary Fig. 3: YL-939 inhibited ferroptosis in different cell lines and its cytotoxicity. (A) YL-939 exhibited very similar ferroptosis inhibitory effects in different cell lines. Data represent mean of two independent biological replicates. (B) YL-939 did not show obvious cytotoxicity against six normal cell lines at concentrations less than 3 μM. Data represent the mean ± SD of three biological replicates. Source data are provided as a Source Data file.

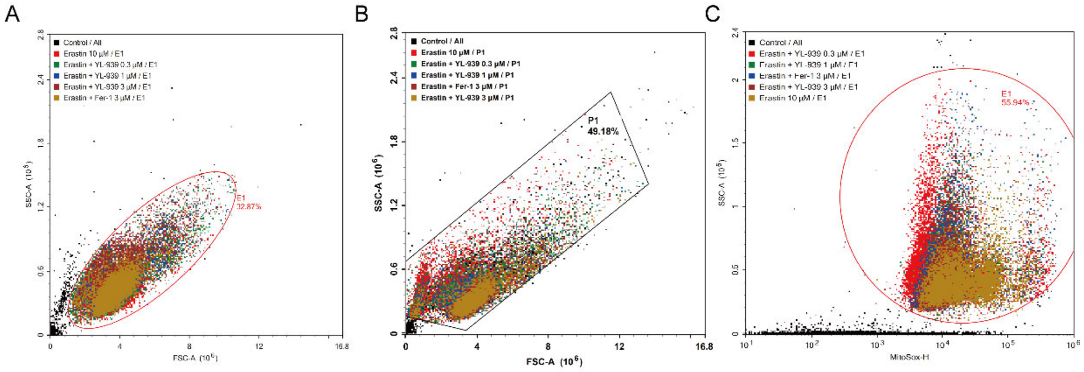

Supplementary Fig. 4: The gating strategy in flow cytometry. Cytosolic, lipid and mitochondrial ROS production assessed by flow cytometry using H2DCFDA (A), C11-BODIPY (B) and MitoSox (C).

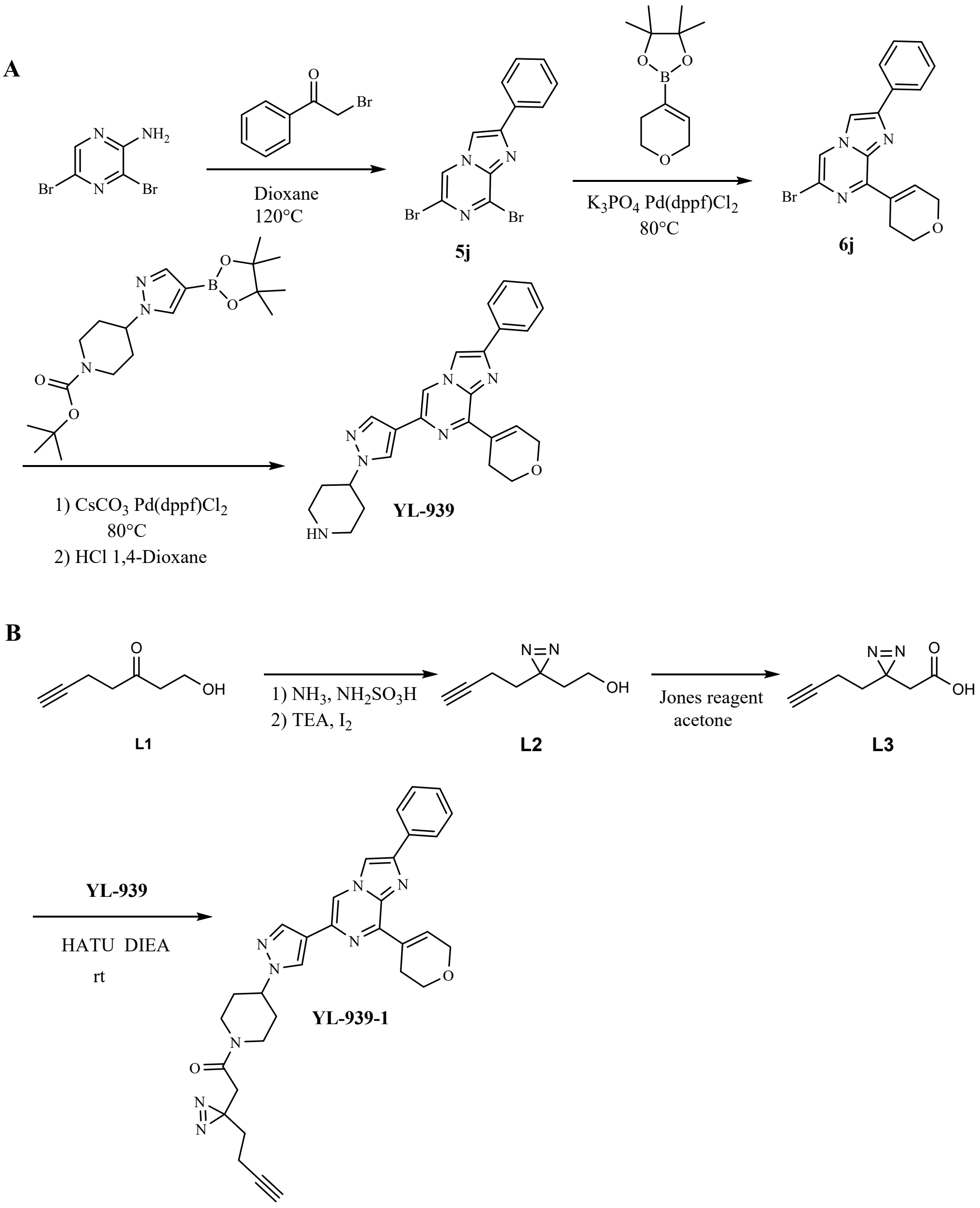

Supplementary Fig. 5: Synthesis of YL-939 and probe YL-939-1. (A) The synthetic route for YL-939. (B) The synthetic route for probe YL-939-1.

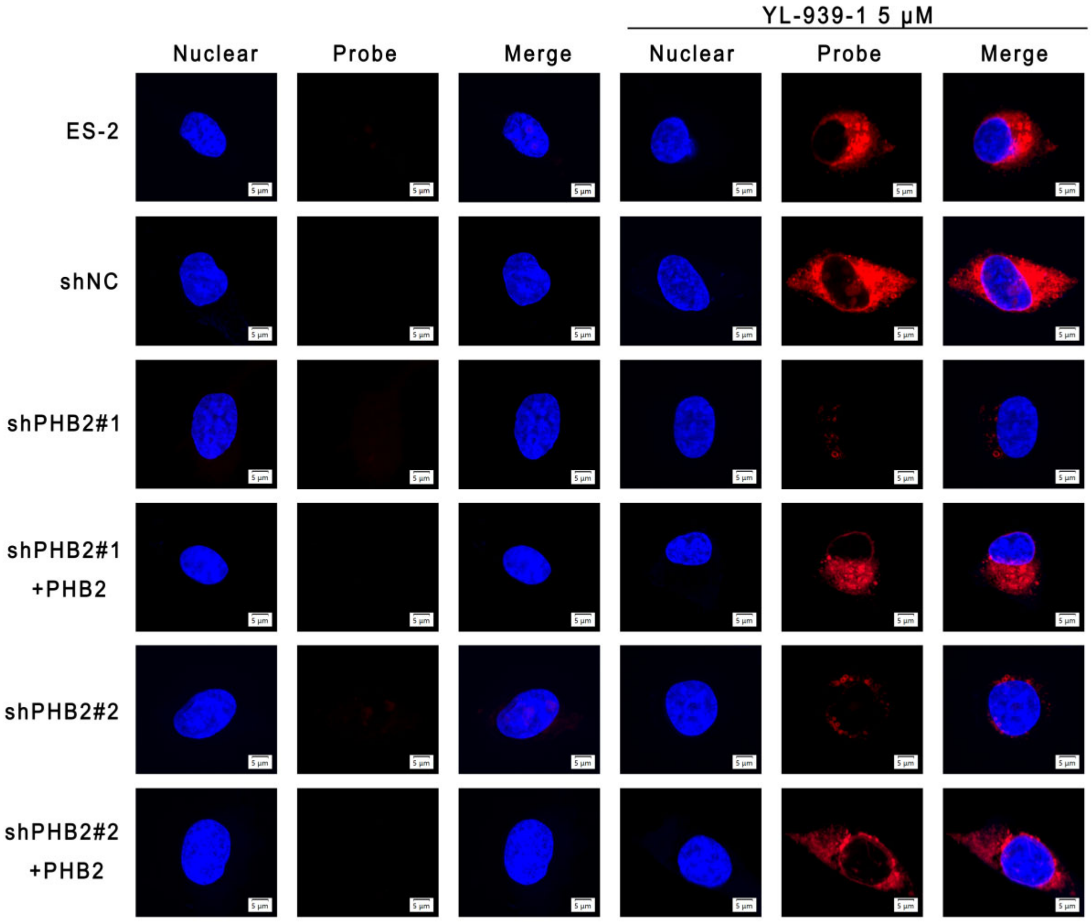

Supplementary Fig. 6: Knockout of PHB2 affected the labeling effect of the probe. Fluorescence imaging of fixed cells were performed at 3 hours after probe exposure to cells. Cells were stained with YL-939-1 probe (in red) and Dapi (in blue). Representative images of two biological replicates were shown. Scale Bars: 5 μm.

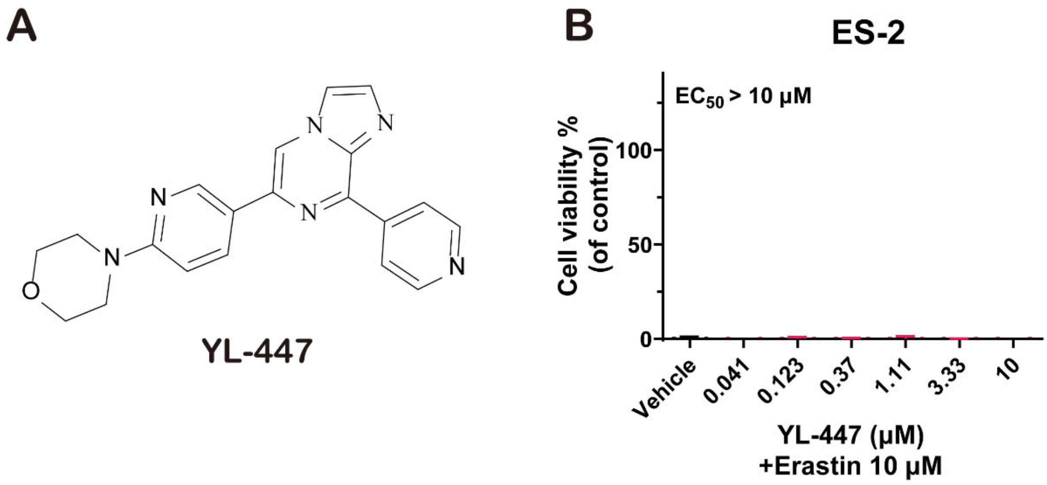

Supplementary Fig. 7: A negative control, YL-447. (A) The chemical structure of YL-447. (B) YL-447 could not protect ES-2 cells from erastin-induce ferroptosis. Data represent mean of two independent biological replicates. Source data are provided as a Source Data file.

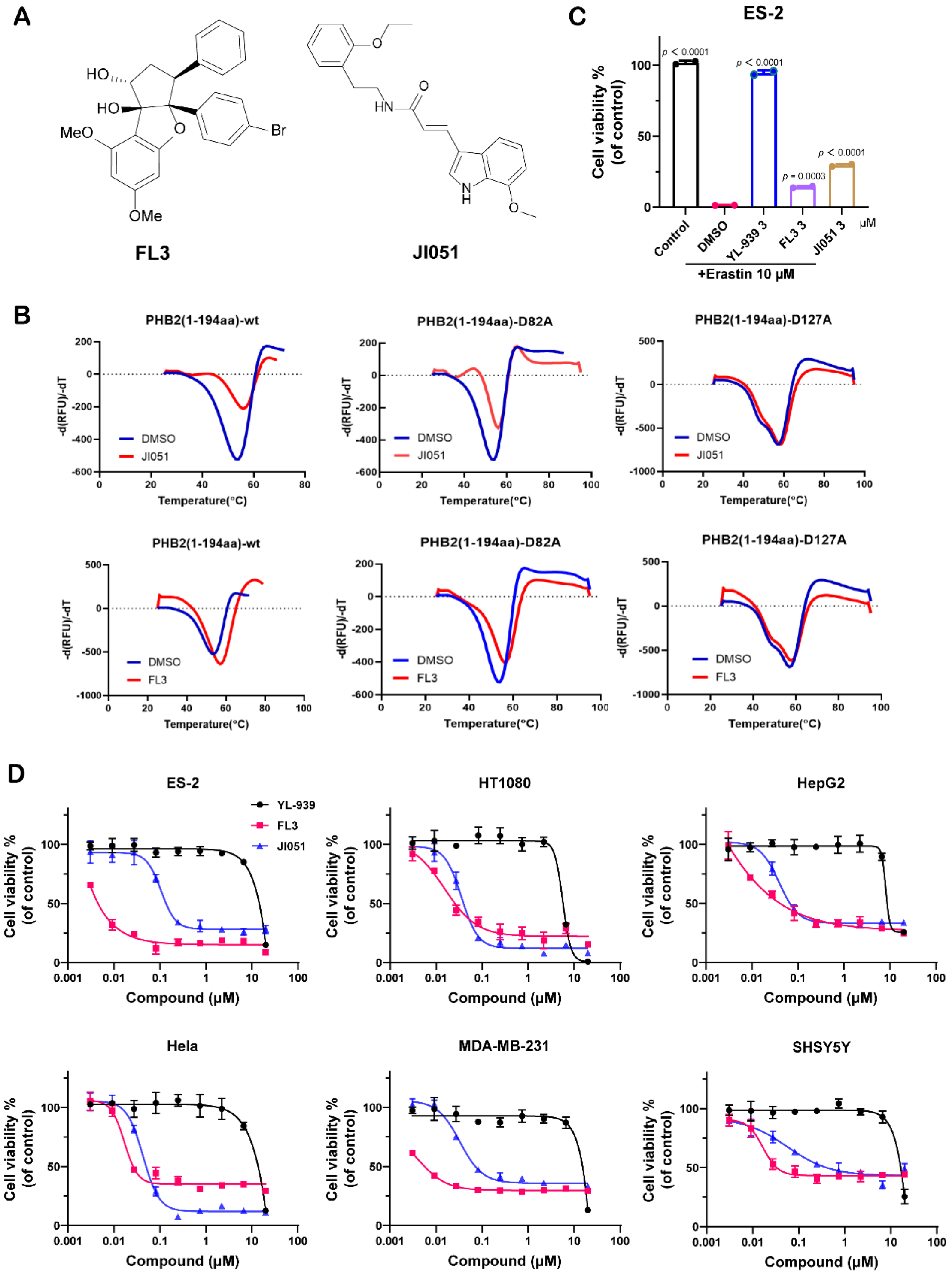

Supplementary Fig. 8: Inhibitory activity of PHB2 ligands against ferroptosis. (A) Chemical structures of FL3 and JI051. (B) DSF analysis of PHB2 ligands binding to the PHB2 wild type (wt) and mutants including PHB2$^{1\text{-}194\text{-}D82A}$ and PHB2$^{1\text{-}194\text{-}D127A}$ protein, respectively. Data were obtained from two biological replicates. (C) PHB2 ligands inhibited Erastin-induced ferroptosis in ES-2 cells. Data represent mean of two independent biological replicates. Statistical analyses were performed by One-way ANOVA with Dunnett's multiple comparisons test. Specific *p*-values are indicated in

the figure. (D) The cytotoxicity of PHB2 ligands measured by the MTT assay. Data represent the mean ± SD of four biological replicates. Source data are provided as a Source Data file.

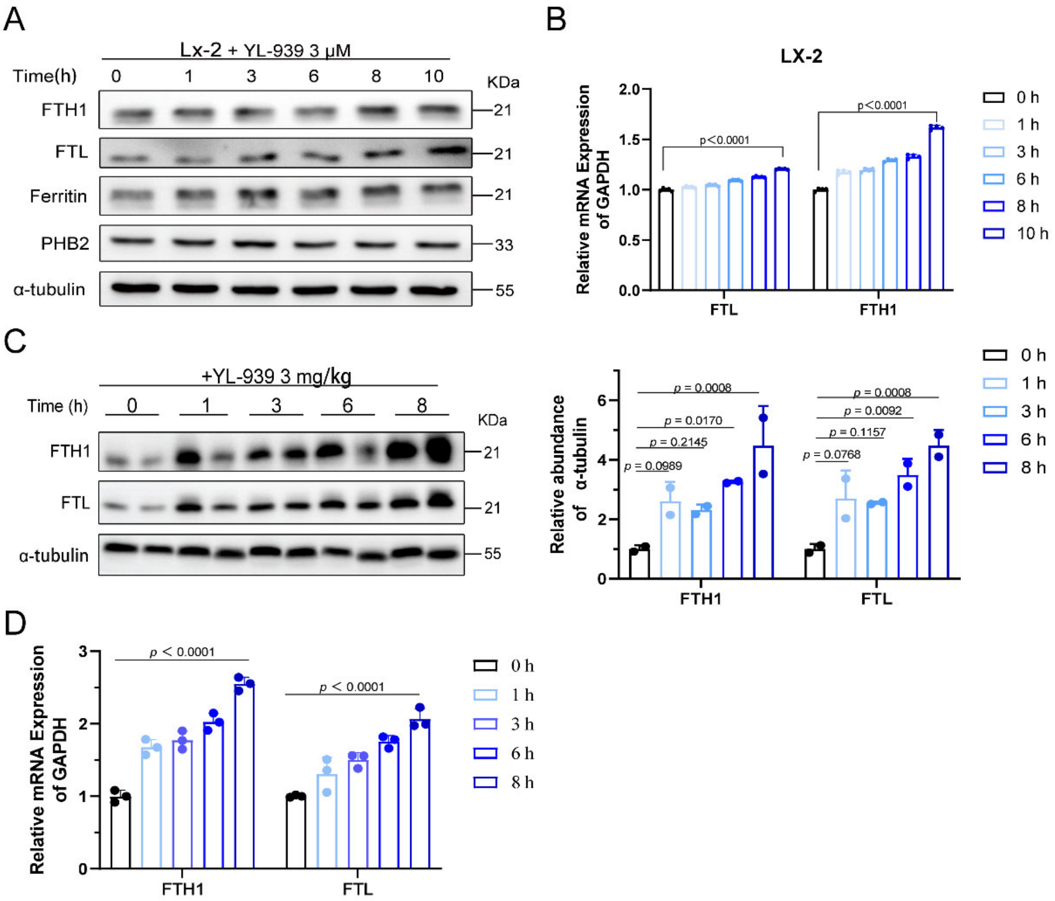

Supplementary Fig. 9: The effect on hepatic ferritin content over time after YL-939 administration. (A) Effects of YL-939 treatment on the expression of hepatic ferritin (FTH1 and FTL) protein over time in LX-2 cells. Blots shown are representative of two biological replicates. (B) YL-939 induced increased mRNA expression of hepatic ferritin over time in LX-2 cells. Data represent the mean ± SD of four biological replicates. Statistical analyses were performed by Two-way ANOVA with Dunnett's multiple comparisons test. Specific *p*-values are indicated in the figure. (C) YL-939 induced increased protein expression of hepatic ferritin (FTH and FTL) in mice ($n = 2$ mice). Blots shown are representative of three biological replicates. And the quantitation of protein level was showed. Statistical analyses were performed by Two-way ANOVA with Sidak's multiple comparisons test. Specific *p*-values are indicated in the figure. (D) YL-939 induced increased mRNA expression of hepatic ferritin (*FTH1* and *FTL*) in mice ($n = 3$ mice). Statistical analyses were performed by Two-way ANOVA with Dunnett's multiple comparisons test. Specific *p*-values are indicated in the figure. Source data are provided as a Source Data file.

Supplementary Table 1. Kinase inhibitory activity of YL-939 (10μM) against 401 kinases. The ATP concentration used was 10 μM.

Kinases	**%Ctrl@10 μM[a]**	Kinases	**%Ctrl@10 μM**
AAK1(h)	68	MARK3(h)	80
Abl(h)	102	MARK4(h)	111
Abl (H396P) (h)	91	MEKK2(h)	100
Abl (M351T)(h)	108	MEKK3(h)	108
Abl (Q252H) (h)	122	MELK(h)	91
Abl(T315I)(h)	119	Mer(h)	78
Abl(Y253F)(h)	123	Met(h)	54
ACK1(h)	85	Met(D1246H)(h)	82
ACTR2(h)	94	Met(D1246N)(h)	71
ALK(h)	106	Met(M1268T)(h)	77
ALK1(h)	94	Met(Y1248C)(h)	70
ALK2(h)	88	Met(Y1248D)(h)	62
ALK4(h)	117	Met(Y1248H)(h)	85
ALK6(h)	105	MINK(h)	95
Arg(h)	108	MKK3(h)	107
AMPKα1(h)	90	MKK6(h)	106
AMPKα2(h)	100	MLCK(h)	60
A-Raf(h)	83	MLK1(h)	86
ARK5(h)	93	MLK2(h)	82
ASK1(h)	110	MLK3(h)	111
Aurora-A(h)	111	Mnk2(h)	74
Aurora-B(h)	89	MOK(h)	88
Aurora-C(h)	101	MRCKα(h)	92
Axl(h)	84	MRCKβ(h)	84
BIKe(h)	115	MRCKγ(h)	95
Blk(h)	100	MSK1(h)	79
BMPR2(h)	99	MSK2(h)	90
Bmx(h)	87	MSSK1(h)	87
BRK(h)	109	MST1(h)	91
BrSK1(h)	91	MST2(h)	78
BrSK2(h)	101	MST3(h)	97
BTK(h)	83	MST4(h)	112
BTK(R28H)(h)	107	mTOR(h)	91
B-Raf(h)	84	mTOR/FKBP12(h)	88
B-Raf(V599E)(h)	90	MuSK(h)	88

CaMKI(h)	81	MYLK2(h)	52
CaMKIß(h)	88	MYO3B(h)	89
CaMKIγ(h)	108	NDR2(h)	87
CaMKIIα(h)	64	NEK1(h)	105
CaMKIIβ(h)	70	NEK2(h)	99
CaMKIIγ(h)	86	NEK4(h)	118
CaMKIδ(h)	90	NEK3(h)	102
CaMKIIδ(h)	68	NEK6(h)	97
CaMKIV(h)	77	NEK7(h)	89
CaMKK1(h)	91	NEK9(h)	99
CaMKK2(h)	83	NIM1(h)	85
Cdc7/cyclinB1(h)	104	NEK11(h)	86
CDK1/cyclinB(h)	92	NLK(h)	86
CDK2/cyclinA(h)	95	NUAK2(h)	72
CDK2/cyclinE(h)	85	p70S6K(h)	61
CDK3/cyclinE(h)	102	PAK1(h)	85
CDK4/cyclinD3(h)	80	PAK2(h)	90
CDK5/p25(h)	120	PAK4(h)	97
CDK5/p35(h)	100	PAK3(h)	70
CDK6/cyclinD3(h)	94	PAK5(h)	95
CDK7/cyclinH/MAT1(h)	92	PAK6(h)	65
CDK9/cyclin T1(h)	119	PAR-1Bα(h)	101
CDK12/cyclinK(h)	99	PEK(h)	99
CDK13/cyclinK(h)	93	PDGFRα(h)	76
CDK14/cyclinY(h)	111	PDGFRα(D842V)(h)	113
CDK16/cyclinY(h)	80	PDGFRα(V561D)(h)	101
CDK17/cyclinY(h)	93	PDGFRβ(h)	113
CDK18/cyclinY(h)	97	PDHK2(h)	87
CDKL1(h)	96	PDHK4(h)	109
CDKL2(h)	74	PDK1(h)	127
CDKL3(h)	82	PhKγ1(h)	85
CDKL4(h)	76	PhKγ2(h)	68
ChaK1(h)	92	Pim-1(h)	68
CHK1(h)	98	Pim-2(h)	84
CHK2(h)	100	Pim-3(h)	103
CHK2(I157T)(h)	87	PKA(h)	98
CHK2(R145W)(h)	98	PKAcβ(h)	94
CK1ε(h)	74	PKBα(h)	68

CK1γ1(h)	112	PKBβ(h)	87
CK1γ2(h)	90	PKBγ(h)	97
CK1γ3(h)	102	PKCα(h)	104
CK1δ(h)	111	PKCβI(h)	98
CK1(y)	71	PKCβII(h)	95
CK2(h)	97	PKCγ(h)	96
CK2α1(h)	103	PKCδ(h)	81
CK2α2(h)	98	PKCε(h)	95
CLIK1(h)	112	PKCη(h)	106
CLK2(h)	71	PKCι(h)	85
CLK3(h)	96	PKCμ(h)	77
cKit(h)	81	PKCθ(h)	93
cKit(D816V)(h)	108	PKCζ(h)	94
cKit(D816H)(h)	90	PKD2(h)	89
cKit(V560G)(h)	88	PKD3(h)	87
cKit(V654A)(h)	92	PKG1α(h)	90
CRIK(h)	101	PKG1β(h)	83
CSK(h)	105	PKR(h)	97
c-RAF(h)	76	Plk1(h)	84
cSRC(h)	78	Plk3(h)	103
DAPK1(h)	82	Plk4(h)	88
DAPK2(h)	138	PRAK(h)	144
DCAMKL2(h)	79	PRKG2(h)	97
DCAMKL3(h)	66	PRK1(h)	96
DDR1(h)	98	PRK2(h)	89
DDR2(h)	95	PrKX(h)	80
DMPK(h)	107	PRP4(h)	129
DRAK1(h)	60	PTK5(h)	75
DRAK2(h)	128	Pyk2(h)	108
DYRK1A(h)	83	Ret(h)	123
DYRK1B(h)	97	Ret (V804L)(h)	81
DYRK2(h)	93	Ret(V804M)(h)	102
DYRK3(h)	77	RIPK1(h)	106
eEF-2K(h)	93	RIPK2(h)	71
EGFR(h)	87	ROCK-I(h)	94
EGFR(L858R)(h)	86	ROCK-II(h)	89
EGFR(L861Q)(h)	61	Ron(h)	99
EGFR(T790M)(h)	138	Ros(h)	97

EGFR(T790M,L858R)(h)	91	Rse(h)	98
EphA1(h)	109	Rsk1(h)	94
EphA2(h)	82	Rsk2(h)	81
EphA3(h)	73	Rsk3(h)	96
EphA4(h)	89	Rsk4(h)	73
EphA5(h)	96	SAPK2a(h)	103
EphA7(h)	99	SAPK2a(T106M)(h)	105
EphA8(h)	76	SAPK2b(h)	84
EphB2(h)	81	SAPK3(h)	75
EphB1(h)	99	SAPK4(h)	100
EphB3(h)	93	SBK1(h)	106
EphB4(h)	97	SGK(h)	72
ErbB2(h)	72	SGK2(h)	96
ErbB4(h)	99	SGK3(h)	83
FAK(h)	112	SIK(h)	100
Fer(h)	86	SIK2(h)	94
Fes(h)	86	SIK3(h)	84
FGFR1(h)	115	SLK(h)	70
FGFR1(V561M)(h)	65	Snk(h)	112
FGFR2(h)	95	SNRK(h)	100
FGFR2(N549H)(h)	94	Src(1-530)(h)	85
FGFR3(h)	83	Src(T341M)(h)	100
FGFR4(h)	94	SRMS(h)	122
Fgr(h)	76	SRPK1(h)	107
Flt1(h)	65	SRPK2(h)	87
Flt3(D835Y)(h)	95	STK16(h)	100
Flt3(h)	86	STK25(h)	82
Flt4(h)	85	STK32A(h)	111
Fms(h)	122	STK32B(h)	105
Fms(Y969C)(h)	107	STK32C(h)	82
Fyn(h)	81	STK33(h)	105
GCK(h)	76	Syk(h)	62
GCN2(h)	67	TAF1L(h)	84
GRK1(h)	109	TAK1(h)	77
GRK2(h)	111	TAO1(h)	81
GRK3(h)	92	TAO2(h)	90
GRK5(h)	72	TAO3(h)	102
GRK6(h)	87	TBK1(h)	90

GSK3α(h)	119	Tec(h) activated	70
GSK3β(h)	96	TGFBR1(h)	101
Haspin(h)	100	TGFBR2(h)	97
Hck(h)	98	Tie2 (h)	81
Hck(h) activated	105	Tie2(R849W)(h)	122
HIPK1(h)	97	Tie2(Y897S)(h)	91
HIPK2(h)	90	TLK1(h)	71
HIPK3(h)	86	TLK2(h)	88
HIPK4(h)	106	TNIK(h)	56
HPK1(h)	88	TRB2(h)	59
HRI(h)	111	TrkA(h)	75
ICK(h)	92	TrkB(h)	81
IGF-1R(h)	82	TrkC(h)	64
IGF-1R(h), activated	95	TSSK1(h)	80
IKKα(h)	92	TSSK2(h)	83
IKKβ(h)	87	TSSK3(h)	87
IKKε(h)	113	TSSK4(h)	97
IR(h)	114	TTBK1(h)	103
IR(h), activated	91	TTBK2(h)	87
IRE1(h)	94	TTK(h)	102
IRR(h)	74	Txk(h)	73
IRAK1(h)	118	TYK2(h)	95
IRAK4(h)	96	ULK1(h)	65
Itk(h)	106	ULK2(h)	77
JAK1(h)	66	ULK3(h)	68
JAK2(h)	90	VRK1(h)	87
JAK3(h)	91	VRK2(h)	92
JNK1α1(h)	109	Wee1(h)	99
JNK2α2(h)	72	Wee1B(h)	92
JNK3(h)	65	WNK1(h)	85
KDR(h)	108	WNK2(h)	76
Lck(h)	122	WNK3(h)	87
Lck(h) activated	77	WNK4(h)	102
LIMK1(h)	86	Yes(h)	90
LIMK2(h)	85	ZAK(h)	85
LKB1(h)	87	ZAP-70(h)	97
LOK(h)	95	ZIPK(h)	88
Lyn(h)	95	ATM(h)	86

LRRK2(h)	103	ATR/ATRIP(h)	88
LTK(h)	108	DNA-PK(h)	94
MAK(h)	74	PI3 Kinase (p110/p85)(h)	73
MAPK1(h)	103	PI3 Kinase (p120)(h)	104
MAPK2(h)	88	PI3 Kinase (p110/p85)(h)	85
MAP4K3(h)	79	PI3 Kinase (p110(E542K)/p85)(h)	99
MAP4K4(h)	86	PI3 Kinase (p110(H1047R)/p85)(h)	99
MAP4K5(h)	76	PI3 Kinase (p110(E545K)/p85)(h)	95
MAPKAP-K2(h)	92	PI3 Kinase (p110/p65)(h)	100
MAPKAP-K3(h)	102	PI3KC2(h)	103
MEK1(h)	109	PI3KC2(h)	82
MEK2(h)	89	PIP4K2(h)	105
MARK1(h)	95	PIP5K1(h)	101
PIP5K1(h)	102		

[a]. The %Ctrl values represent the inhibitory activity of the compound against the kinases. The lower the value of %Ctrl, the stronger the binding ability of the test compound to the kinase.

Supplementary Table 2. Protein hits identified by LC-MS/MS with YL-939-1.

Protein IDs	Mol. weight [kDa]	Score	Intensity
Q9C019|TRI15	52.112	5.6954	2281000000
Q96RW7|HMCN1	613.38	18.593	1565600000
P21796|VDAC1	30.772	306.62	1366900000
P60709|ACTB	41.736	323.31	1316900000
Q96L93|KI16B	152.01	6.1599	1038500000
Q86SH2|ZAR1	45.872	6.723	885740000
Q8WXI2|CNKR2	117.53	6.9496	848050000
Q01130|SRSF2	25.476	5.769	797650000
P47914|RL29	17.752	11.625	769470000
Q99828|CIB1	21.703	5.9376	762470000
Q9NQC3|RTN4	129.93	131.64	696960000
P45880|VDAC2	31.566	121.26	665630000
Q9ULL4|PLXB3	206.84	5.5577	623260000
P08238|HS90B; Q58FF7|H90B3; Q58FF8|H90B2; Q58FF6|H90B4; Q58FG0|HS905	83.263	109.95	541890000
P0DML3|CSH2; P0DML2|CSH1; Q14406|CSHL	24.994	5.5598	476270000
O95359|TACC2	309.42	12.079	416080000
Q99623|PHB2	33.296	11.694	408410000
Q5VTE0|EF1A3; P68104|EF1A1; Q05639|EF1A2	50.184	22.549	403910000
Q9BQE3|TBA1C; Q13748|TBA3C; Q71U36| TBA1A; P68363|TBA1B; Q6PEY2|TBA3E; P68366|TBA4A; Q9NY65|TBA8; A6NHL2|TBAL3	49.895	56.47	391190000
P61313|RL15	24.146	9.1504	342940000
P04264|K2C1; CON__P04264; CON__ENSEMBL:ENSBTAP00000038253; CON__Q9R0H5; CON__Q6NXH9; CON__Q6IFZ6; Q7Z794|K2C1B; CON__Q7Z794	66.038	124.82	326130000
P00338|LDHA; Q6ZMR3|LDH6A; P07864|LDHC	36.688	43.246	315020000
P51172|SCNND	70.214	5.6492	285240000
O75396|SC22B	24.593	146.06	284030000
P07437|TBB5; P68371|TBB4B; P04350|TBB4A; Q9BVA1|TBB2B; Q13885|TBB2A; Q3ZCM7| TBB8; A6NNZ2|TBB8L; Q13509|TBB3; Q9H4B7| TBB1; CON__ENSEMBL:ENSBTAP00000025008	49.67	145.66	281500000
REV__ Q9BZF1|OSBL8	101.19	5.9653	277210000
P08670|VIME; P17661|DESM; Q16352|AINX;	53.651	71.525	252180000

P07196|NFL; P07197|NFM P12036|NFH			
P84098|RL19	23.466	42.471	251610000
P12236|ADT3; P12235|ADT1	32.866	19.161	214610000
Q8TCT9|HM13	41.488	42.549	206450000
Q12802|AKP13	307.55	5.6524	204980000
Q9Y587|AP4S1	17.005	6.4355	197410000
P36578|RL4	47.697	45.111	197250000
P35908|K22E; CON__P35908	65.432	38.27	189260000
P15880|RS2	31.324	22.333	186550000
P00749|UROK	48.507	6.7949	164900000
P62987|RL40; P62979|RS27A; P0CG47|UBB; P0CG48|UBC	14.728	54.911	161080000
Q9NZN4|EHD2	61.161	5.5897	158110000
REV__ O60513|B4GT4	40.041	6.03	156170000
P63261|ACTG	41.792	98.072	155310000
Q07020|RL18	21.634	20.187	144960000
P20929|NEBU	772.91	6.7972	144580000
Q9BW60|ELOV1	32.662	11.65	143850000
Q9H3N1|TMX1	31.791	12.21	140240000
Q9NS69|TOM22	15.521	114.38	136730000
Q8N7X1|RMXL3	114.94	6.2616	132860000
Q07065|CKAP4	66.022	117.62	129770000
P13645|K1C10; CON__P13645; CON__P02535-1	58.826	231.28	128670000
P10809|CH60	61.054	43.358	125110000
P11142|HSP7C; P54652|HSP72; P34931|HS71L; P0DMV9|HS71B; P0DMV8|HS71A	70.897	93.442	123920000
P04406|G3P	36.053	23.531	122990000
P62263|RS14	16.273	18.94	121420000
P14618|KPYM	57.936	36.32	118650000
P06748|NPM	32.575	6.7246	117320000
P07355|ANXA2; A6NMY6|AXA2L	38.604	44.961	115420000
P38646|GRP75	73.68	25.795	114880000
P62424|RL7A	29.995	12.051	111340000
P62280|RS11	18.431	24.559	109130000
P05141|ADT2; Q9H0C2|ADT4	32.852	6.5799	107690000
P62826|RAN	24.423	12.713	100880000
P62241|RS8	24.205	46.06	98782000
Q86UE4|LYRIC	63.836	33.416	96556000

Q15005|SPCS2	25.003	7.2824	92487000
Q9Y5M8|SRPRB	29.702	19.435	92181000
CON__P15497	30.276	12.784	91802000
P50914|RL14	23.432	7.1647	91582000
P46779|RL28	15.747	14.811	88630000
P08195|4F2	67.993	23.062	88418000
P60468|SC61B	9.9743	88.952	87617000
P26373|RL13	24.261	33.184	86216000
P04843|RPN1	68.569	35.617	85652000
P50990|TCPQ	59.62	18.79	85014000
P18621|RL17	21.397	7.5896	84343000
O43175|SERA	56.65	18.55	84044000
P13639|EF2	95.337	34.581	83292000
Q14315|FLNC; O75369|FLNB	291.02	64.172	81714000
P30101|PDIA3	56.782	16.827	79960000
P60059|SC61G	7.7412	7.1638	79820000
P35527|K1C9; CON__P35527	62.064	101.1	78965000
Q12912|LRMP	62.121	5.6699	78399000
P03956|MMP1	54.006	5.7851	75034000
P04075|ALDOA	39.42	41.703	74674000
Q15365|PCBP1	37.497	36.316	74205000
P35232|PHB	29.804	25.135	71022000
P52272|HNRPM	77.515	25.462	69052000
P23396|RS3	26.688	19.684	69027000
Q96N66|MBOA7	52.764	23.451	65757000
Q06830|PRDX1; P32119|PRDX2	22.11	13.807	64400000
Q9UQ35|SRRM2	299.61	44.659	64152000
Q00325|MPCP	40.094	7.7577	62861000
P61978|HNRPK	50.976	26.825	62588000
Q99541|PLIN2	48.075	13.527	61632000
Q92945|FUBP2	73.114	24.394	60716000
P62829|RL23	14.865	13.903	59935000
P05023|AT1A1; P13637|AT1A3; P50993|AT1A2; P20648|ATP4A	112.89	43.468	59051000
P11021|BIP	72.332	49.254	55630000
P16615|AT2A2; O14983|AT2A1; Q93084|AT2A3	114.76	28.684	55586000
P26038|MOES	67.819	34.044	49747000
P43243|MATR3	94.622	159.61	47879000

Q07666|KHDR1	48.227	12.605	45749000
P62942|FKB1A	11.951	6.0988	44385000
Q9NZJ7|MTCH1	41.544	5.7708	43746000
P63244|RACK1	35.076	12.556	43209000
P02656|APOC3	10.852	8.8482	42387000
CON__Q2UVX4	187.37	63.627	42214000
Q9UM47|NOTC3	243.63	5.785	41514000
Q16891|MIC60	83.677	24.781	40718000
P40939|ECHA	82.999	19.074	40534000
P02533|K1C14; CON__P02533; Q04695|K1C17; CON__Q04695; CON__Q9QWL7; P08779|K1C16; CON__P08779; P08727|K1C19; CON__P08727; CON__P19001; CON__Q6IFX2; P19012|K1C15; CON__P19012; CON__A2A4G1; CON__Q9Z2K1; CON__Q3ZAW8; Q14525|KT33B; CON__Q14525; Q15323|K1H1; CON__Q9UE12; CON__Q15323; CON__A2A5Y0; CON__ENSEMBL:ENSP00000377550; CON__P05784; CON__Q92764; P05783|K1C18; CON__P08730-1; Q14532|K1H2; CON__Q14532; O76014|KRT37; CON__A2AB72; Q92764|KRT35; CON__Q497I4; O76015|KRT38; CON__O76015; P13646|K1C13; Q7Z3Y7|K1C28; CON__Q148H6; O76013|KRT36; CON__O76013; CON__O76014; CON__REFSEQ:XP_986630; CON__Q7Z3Y7; Q2M2I5|K1C24; CON__Q2M2I5	51.561	43.697	40530000
P62258|1433E	29.174	12.591	40113000
P07195|LDHB	36.638	101.9	39517000
P51572|BAP31	27.991	13.317	39277000
P46782|RS5	22.876	69.187	38880000
P05556|ITB1	88.414	18.421	38725000
P07237|PDIA1	57.116	30.158	37114000
P07737|PROF1	15.054	19.245	36850000
Q5J8M3|EMC4	20.086	12.392	36012000
CON__ENSEMBL:ENSBTAP00000034412	22.336	12.965	35135000
Q14247|SRC8	61.585	25.465	34860000
Q9H3K2|GHITM	37.205	6.6074	34242000
P37802|TAGL2	22.391	31.155	32709000

Q9UBM7|DHCR7	54.489	17.77	32328000
P06733|ENOA; P13929|ENOB; P09104|ENOG	47.168	19.408	32125000
Q7Z4T9|CFA91	89.954	5.734	32091000
P61619|S61A1; Q9H9S3|S61A2	52.264	11.949	31823000
Q00839|HNRPU	90.583	18.631	30266000
P51148|RAB5C	23.482	6.4621	29348000
P18124|RL7	29.225	14.29	29136000
P40926|MDHM	35.503	6.5916	28014000
P22234|PUR6	47.079	14.273	27733000
Q96AE4|FUBP1	67.56	14.97	27521000
Q9Y3U8|RL36	12.254	6.7496	27477000
Q9Y5Z9|UBIA1	36.831	8.1968	27390000
P25398|RS12	14.515	183.16	26558000
P62910|RL32	15.86	12.108	26466000
P13667|PDIA4	72.932	6.5409	26463000
P51571|SSRD	18.998	6.5359	26121000
Q15046|SYK	68.047	7.2311	25726000
P78371|TCPB	57.488	5.8093	25054000
P0DP25|CALM3; P0DP24|CALM2; P0DP23|CALM1	16.837	6.5478	24397000
Q09666|AHNK	629.09	17.407	24363000
Q9Y3E0|GOT1B	15.425	6.8983	23875000
P31943|HNRH1; P55795|HNRH2	49.229	29.508	23531000
Q9NX14|NDUBB	17.316	5.7328	23434000
Q92841|DDX17; P17844|DDX5	80.272	18.927	21639000
P63104|1433Z	27.745	6.7147	21189000
Q9NP79|VTA1	33.879	6.4769	20988000
A6NCN2|KR87P; O43790|KRT86; CON__O43790; P78385|KRT83; CON__Q6NT21; CON__P78385; Q14533|KRT81 ;CON__Q14533	29.117	6.6885	20583000
P27824|CALX	67.567	14.457	19833000
O00299|CLIC1	26.922	6.4313	19698000
Q8N511|TM199	23.13	7.2636	19525000
Q14974|IMB1	97.169	6.3216	19061000
P51575|P2RX1	44.98	5.7654	18753000
Q02878|RL6	32.728	13.62	18752000
O43776|SYNC	62.942	5.7648	18550000
P48741|HSP77; P17066|HSP76	40.244	6.227	18536000
O75607|NPM3	19.343	6.6947	18329000

Q6Y1H2|HACD2	28.368	6.3762	17961000
Q96RQ3|MCCA	80.472	12.856	17608000
Q02543|RL18A	20.762	11.772	17485000
P18206|VINC	123.8	11.97	17169000
Q9UQ80|PA2G4	43.786	13.271	17092000
Q9NX76|CKLF6	20.419	5.6386	16390000
Q01844|EWS	68.477	6.4056	16290000
P21980|TGM2	77.328	32.287	15761000
Q8NHH9|ATLA2	66.228	6.1817	15607000
P53611|PGTB2	36.924	11.962	15414000
O75477|ERLN1; O94905|ERLN2	38.925	12.823	15110000
P54577|SYYC	59.143	17.276	15039000
P11387|TOP1	90.725	6.4636	14726000
P42167|LAP2B; P42166|LAP2A	50.67	17.991	14673000
Q9NX00|TM160	19.657	5.7603	14645000
P07814|SYEP	170.59	11.872	14641000
Q5JTH9|RRP12	143.7	12.498	14408000
Q01650|LAT1	55.01	5.7446	14191000
P35268|RL22	14.787	6.6637	13765000
O75533|SF3B1	145.83	11.992	13716000
Q9Y6N5|SQOR	49.96	6.297	13652000
P05120|PAI2	46.596	6.7934	13530000
Q08211|DHX9	140.96	6.4154	13468000
P21333|FLNA	280.74	12.585	13466000
O60664|PLIN3	47.074	18.463	13453000
Q86VK4|ZN410	52.113	5.5866	13282000
P02768|ALBU; CON__P02768-1	69.366	323.31	13218000
Q15637|SF01	68.329	39.14	12869000
Q9BQG0|MBB1A	148.85	6.2843	12840000
P49721|PSB2	22.836	12.978	12590000
O00231|PSD11	47.463	7.9843	12268000
Q12906|ILF3	95.337	5.7355	12185000
Q9Y4W6|AFG32	88.583	6.4009	12000000
P55265|DSRAD	136.06	5.9025	11925000
P29279|CTGF	38.091	11.451	11780000
O14828|SCAM3	38.287	11.724	11731000
P22314|UBA1	117.85	55.375	11725000
P47895|AL1A3	56.108	11.192	11667000

P06576|ATPB	56.559	5.9282	11662000
Q01469|FABP5	15.164	10.235	11570000
Q8WVM8|SCFD1	72.379	6.0211	11540000
Q15366|PCBP2; P57721|PCBP3	38.58	5.6403	11465000
P30519|HMOX2	36.032	6.8048	11110000
P25705|ATPA	59.75	11.975	10863000
Q99459|CDC5L	92.25	6.3228	10835000
Q16543|CDC37	44.468	6.6866	10789000
P27816|MAP4	121	5.8072	10788000
Q9Y241|HIG1A	10.143	12.618	10714000
P46776|RL27A	16.561	12.887	10474000
P30626|SORCN	21.676	6.5415	10279000
Q13428|TCOF	152.1	5.9272	10063000
Q8NAP8|ZBT8B	54.175	5.9991	10058000
Q6NVV1|R13P3; P40429|RL13A	12.134	5.7973	9844000
Q9Y2Z0|SGT1	41.024	8.9161	9623100
P58546|MTPN	12.895	6.4292	9496000
O43491|E41L2	112.59	5.9475	9377300
Q6PIU2|NCEH1	45.807	12.225	9357900
Q5SSJ5|HP1B3	61.206	6.6149	9325600
P60228|EIF3E	52.22	6.3113	9275400
Q9GZM5|YIPF3	38.247	7.2337	9119900
P11586|C1TC	101.56	5.6418	9098200
O95573|ACSL3	80.419	11.917	8953400
Q15029|U5S1	109.43	12.258	8914200
Q9BUF5|TBB6	49.857	6.3687	8884700
P16070|CD44	81.537	6.1813	8816600
P51610|HCFC1	208.73	17.328	8786800
Q04637|IF4G1	175.49	5.7292	8712700
P05165|PCCA	80.058	6.0973	8693300
Q9Y2Q3|GSTK1	25.497	6.0325	8691500
Q8N2K0|ABD12	45.096	5.9272	8515700
P18031|PTN1	49.966	6.5088	8423200
P26639|SYTC	83.434	5.9946	8330100
O95070|YIF1A	32.011	5.7097	8298000
P02545|LMNA	74.139	12.211	8271000
Q8TC12|RDH11	35.386	5.684	8251100
Q8WUM4|PDC6I	96.022	5.6685	8220900

P60900|PSA6	27.399	5.5843	8196600
P00387|NB5R3	34.234	6.1494	8162200
Q53GQ0|DHB12	34.324	6.6842	8106600
CON__P02070; CON__Q3SX09	15.954	6.5411	8092600
Q6EEV6|SUMO4; P61956|SUMO2; P55854|SUMO3	10.685	5.9985	8088100
P49448|DHE4; P00367|DHE3	61.433	5.8884	8084700
Q9C0A1|ZFHX2	274.17	5.5499	8079000
Q9Y262|EIF3L	66.726	5.7735	7922700
P51149|RAB7A	23.489	22.791	7802200
P04844|RPN2	69.283	11.62	7797500
P29692|EF1D	31.121	6.031	7759300
P30050|RL12	17.818	12.909	7756300
Q15006|EMC2	34.833	6.4999	7726000
Q15020|SART3	109.93	61.803	7684500
Q9Y6I9|TX264	34.188	6.7272	7646300
O94925|GLSK	73.46	6.2661	7607400
Q9Y3F4|STRAP	38.438	6.5404	7539600
P14625|ENPL	92.468	5.7096	7425600
P61106|RAB14	23.897	5.5921	7236800
O43399|TPD54	22.237	12.699	7182800
P18085|ARF4	20.511	5.7435	7090600
Q9NUU7|DD19A; Q9UMR2|DD19B	53.974	5.5908	7057900
P23284|PPIB	23.742	5.6366	7049600
Q53H12|AGK	47.137	7.5736	7048600
Q9NZI8|IF2B1	63.48	6.0948	6976200
P25205|MCM3	90.98	6.2329	6946200
P23526|SAHH	47.716	6.8327	6826300
Q9GZR7|DDX24	96.331	6.3223	6741300
P22626|ROA2	37.429	13.036	6739000
Q99832|TCPH	59.366	11.811	6659500
Q86UP2|KTN1	156.27	12.507	6541300
P04083|ANXA1	38.714	6.758	6342500
Q53HI1|UNC50	30.372	10.882	6340700
Q8NHW5|RLA0L; P05388|RLA0	34.364	5.8191	6238000
Q9Y6C9|MTCH2	33.331	102.54	6223900
P10599|THIO	11.737	5.5736	6188900
P46087|NOP2	89.301	6.0611	6178100
P61803|DAD1	12.497	6.1736	6172200

Q13435|SF3B2	100.23	5.8372	6107100
P34932|HSP74	94.33	5.6022	5995800
P49750|YLPM1	241.64	6.2291	5988500
Q9BZE4|NOG1	73.964	6.5669	5954800
P52597|HNRPF	45.671	12.328	5903600
Q9Y679|AUP1	53.028	8.4372	5789300
Q6ZXV5|TMTC3	104.01	6.5132	5782500
Q6P1Q9|MET2B	43.426	6.0175	5642500
Q00688|FKBP3	25.177	6.767	5536600
Q92968|PEX13	44.129	6.785	5531900
Q14684|RRP1B	84.427	5.9851	5498700
P26640|SYVC	140.47	6.3609	5457300
P09874|PARP1	113.08	6.5705	5372500
Q7KZF4|SND1	102	20.403	5352000
Q15758|AAAT	56.598	5.9103	5312500
Q9NSD9|SYFB	66.115	6.5402	5309600
P46013|KI67	358.69	5.9518	5257800
P07741|APT	19.608	7.3467	5215500
Q9Y277|VDAC3	30.658	5.7491	5164700
P39023|RL3	46.108	12.115	5091300
P55072|TERA	89.321	6.3039	4996800
Q14061|COX17	6.9151	6.4076	4980400
P53992|SC24C	118.32	5.7603	4972400
P00505|AATM	47.517	5.9345	4970700
Q14103|HNRPD	38.434	6.2918	4929600
P51114|FXR1	69.72	6.0705	4905600
O76003|GLRX3	37.432	6.0028	4890200
Q9NR30|DDX21	87.343	6.1183	4882000
P51153|RAB13	22.774	6.519	4840700
Q15084|PDIA6	48.121	5.8865	4836700
Q01085|TIAR	41.59	6.0734	4825100
O00567|NOP56	66.049	5.9362	4820000
Q04941|PLP2	16.691	5.9376	4736600
CON__Q3MHN2; P02748|CO9	61.998	5.7534	4725700
O96005|CLPT1	76.096	6.1901	4716400
P78344|IF4G2	102.36	7.3236	4714800
Q99613|EIF3C; B5ME19|EIFCL	105.34	6.676	4632200
P29401|TKT	67.877	6.6979	4605500

Q7Z2T5|TRM1L	81.746	9.1811	4561600
P56134|ATPK	10.918	6.8674	4556600
Q8IY81|SPB1	96.557	5.7048	4527200
P46777|RL5	34.362	6.6908	4479500
Q9BQ04|RBM4B; Q9BWF3|RBM4	40.149	13.292	4413900
Q9Y520|PRC2C	316.91	5.7009	4339900
Q86VR2|RETR3	51.396	7.1683	4246100
Q14318|FKBP8	44.561	6.4206	4126700
Q969Q0|RL36L; P83881|RL36A	12.469	5.8093	4081300
Q86TI2|DPP9	98.262	6.7078	4080500
P49748|ACADV	70.389	6.6258	4074500
P31948|STIP1	62.639	5.7054	4035900
O60493|SNX3	18.762	5.9824	4014500
Q96L92|SNX27	61.264	5.6156	3883200
Q9Y383|LC7L2	46.513	7.1392	3848000
P62913|RL11	20.252	6.2501	3806700
P61289|PSME3	29.506	6.0698	3763000
P60842|IF4A1; Q14240|IF4A2	46.153	6.5061	3724000
Q15417|CNN3	36.413	13.961	3704100
Q15149|PLEC	531.78	6.1665	3688900
P67809|YBOX1	35.924	6.6509	3636800
CON__P34955	46.103	5.7962	3614800
O95674|CDS2	51.417	6.9983	3607400
P32969|RL9	21.863	18.348	3531100
P35240|MERL	69.689	7.5601	3521700
Q9Y2R0|COA3	11.731	6.7612	3463200
Q14019|COTL1	15.945	5.5549	3406500
Q96P70|IPO9	115.96	7.1166	3397000
P08754|GNAI3	40.532	6.3835	3244600
CON__Q3ZBS7	54.099	5.5999	3183400
P53814|SMTN	99.058	6.4747	3152800
P36888|FLT3	112.9	5.561	3147600
P07900|HS90A; Q14568|HS902	84.659	5.7419	3113100
P51991|ROA3	39.594	5.742	3084900
Q9NT62|ATG3	35.864	6.0063	3061200
P36021|MOT8	59.511	7.443	3000900
P33176|KINH	109.68	5.8146	2991600
P06493|CDK1	34.095	6.5798	2957400

Q96KB5|TOPK	36.085	6.4141	2887800
O15523|DDX3Y; O00571|DDX3X	73.153	5.9041	2887100
CON__Q32PJ2	43.017	6.6042	2850200
Q2TAZ0|ATG2A	212.86	5.5663	2816700
Q96FW1|OTUB1	31.284	6.2049	2796000
P49588|SYAC	106.81	5.8968	2702200
O00116|ADAS	72.911	8.0358	2686700
Q13733|AT1A4	114.17	5.961	2669600
Q96TC7|RMD3	52.118	6.4292	2525800
Q9HCC0|MCCB	61.332	5.7063	2439700
Q8NG11|TSN14	30.69	5.7157	2378900
Q9H920|RN121	37.882	6.8648	2347000
O75410|TACC1	87.793	5.8093	2302900
P30566|PUR8	54.889	5.6419	2262300
Q05BV3|EMAL5	219.42	5.5598	2260500
Q9NX40|OCAD1	27.626	6.3637	2229500
O43707|ACTN4	104.85	6.2474	2146400
P40938|RFC3	40.556	5.8077	2145800
Q9NXW2|DJB12	41.818	6.877	2116000
P36871|PGM1	61.448	5.584	2028200
Q9NPC2|KCNK9	42.263	5.6585	1988900
Q15022|SUZ12	83.054	5.755	1937100
Q8WVX3|CD003	7.6035	6.8296	1924000
Q6NUQ4|TM214	77.15	6.0622	890340
Q16877|F264	54.039	5.6175	864530
P62937|PPIA	18.012	13.2	0
Q9NZM1|MYOF	234.71	12.687	0
Q9NQX3|GEPH	79.748	10.215	0
O75521|ECI2	43.585	8.3421	0
Q13283|G3BP1	52.164	7.9887	0
Q01518|CAP1	51.901	7.8453	0
Q15717|ELAV1; Q12926|ELAV2; P26378|ELAV4	36.091	7.8275	0
P23528|COF1	18.502	7.7853	0
P61088|UBE2N; Q5JXB2|UE2NL	17.138	7.7791	0
F5HB81|GB_HHV8P	93.983	7.6611	0
O15231|ZN185	73.525	7.5584	0
P28482|MK01	41.389	7.409	0
O15160|RPAC1	39.249	7.3706	0

P55060|XPO2	110.42	7.1631	0
P08621|RU17	51.556	6.8963	0
P50395|GDIB	50.663	6.8096	0
O43324|MCA3	19.81	6.767	0
Q9NQT5|EXOS3	29.572	6.7405	0
P14866|HNRPL	64.132	6.6839	0
P49023|PAXI	64.505	6.6539	0
Q6PHR2|ULK3	53.444	6.6156	0
P35579|MYH9	226.53	6.6068	0
Q71UM5|RS27L; P42677|RS27	9.4771	6.6006	0
P60174|TPIS	30.791	6.5779	0
O60437|PEPL	204.74	6.5774	0
Q96JJ7|TMX3	51.871	6.5759	0
P48739|PIPNB	31.54	6.5516	0
P26641|EF1G	50.118	6.5338	0
P10412|H14	21.865	6.5029	0
Q6P2Q9|PRP8	273.6	6.4825	0
Q9NRX5|SERC1	50.494	6.4326	0
P09601|HMOX1	32.818	6.3947	0
P49327|FAS	273.42	6.3404	0
Q15393|SF3B3	135.58	6.3009	0
Q9UM00|TMCO1	21.175	6.2732	0
P62917|RL8	28.024	6.2215	0
P27635|RL10	24.604	6.1992	0
Q9Y5F2|PCDBB	87.087	6.0779	0
P52564|MP2K6; P46734|MP2K3	37.492	6.0482	0
Q96HE7|ERO1A	54.392	6.0275	0
P62491|RB11A; Q15907|RB11B	24.393	5.9736	0
O75083|WDR1	66.193	5.9634	0
REV__ Q86YR7|MF2L2	126.99	5.9103	0
Q99816|TS101	43.944	5.8941	0
Q16831|UPP1	33.934	5.8941	0
Q32M45|ANO4	111.46	5.8595	0
P50991|TCPD	57.924	5.8532	0
Q5VT25|MRCKA	197.3	5.8371	0
Q9HCE3|ZN532	141.69	5.8111	0
P01023|A2MG; CON__ENSEMBL:ENSBTAP00000024146	163.29	5.7991	0

Q9UPP5|K1107	155.68	5.7881	0
Q6ZNG0|ZN620	48.502	5.7495	0
Q99700|ATX2	140.28	5.7253	0
O00273|DFFA	36.521	5.6756	0
P35713|SOX18	40.891	5.6706	0
Q7Z3I7|ZN572	61.238	5.6608	0
Q9BYT5|KRA22; Q9BYU5|KRA21; Q9BYR9|KRA24; P0C7H8|KRA23; CON__Q9BYR9	12.957	5.6536	0
P17858|PFKAL; Q01813|PFKAP	85.018	5.6511	0
Q5IJ48|CRUM2	134.26	5.6489	0
Q15643|TRIPB	227.58	5.6428	0
Q9NTJ3|SMC4	147.18	5.6371	0
P35900|K1C20;CON__P35900	48.486	5.6205	0
Q9BQ39|DDX50	82.564	5.587	0
Q5SVZ6|ZMYM1	128.72	5.5851	0
P22105|TENX	458.22	5.5809	0
Q16584|M3K11	92.687	5.5672	0
Q9H582|ZN644	149.56	5.5665	0
Q8NGL7|OR4P4	35.795	5.5663	0
Q96FI4|NEIL1	43.684	5.5642	0
Q5H9F3|BCORL	182.52	5.5633	0
Q9UMN6|KMT2B	293.51	5.5609	0
Q5SZL2|CE85L	91.807	5.5528	0

Supplementary Table 3. Proteins labeled by YL-939-l with molecular weight of about 30~34 KDa identified by LC-MS/MS.

Protein IDs	Protein names	Molecular weight (KDa)	Score	Intensity
P21796	VDAC1	30.77	306.62	1366900000
P45880	VDAC2	31.56	121.26	665630000
Q99623	PHB2	33.29	11.69	408410000
P12236	ADT3	32.86	19.16	214610000
P15880	RS2	31.32	22.33	186550000
Q9BW60	ELOV1	32.66	11.65	143850000
Q9H3N1	TMX1	31.79	12.21	140240000
P06748	NPM	32.57	6.72	117320000
P05141	ADT2	32.85	6.57	107690000
Q9NP79	VTA1	33.87	6.47	20988000
Q02878	RL6	32.72	13.62	18752000
O95070	YIF1A	32.01	5.70	8298000
P00387	NB5R3	34.23	6.14	8162200
Q53GQ0	DHB12	34.32	6.68	8106600
P29692	EF1D	31.12	6.03	7759300
Q15006	EMC2	34.83	6.49	7726000
Q9Y6I9	TX264	34.18	6.72	7646300
Q53HI1	UNC50	30.37	10.88	6340700
Q8NHW5	RLA0L	34.36	5.81	6238000
Q9Y6C9	MTCH2	33.33	102.54	6223900
Q9Y277	VDAC3	30.65	5.74	5164700
P46777	RL5	34.36	6.69	4479500
P06493	CDK1	34.09	6.57	2957400
Q96FW1	OTUB1	31.28	6.20	2796000
Q8NG11	TSN14	30.69	5.71	2378900
P60174	TPIS	30.79	6.57	-

P48739	PIPNB	31.54	6.55	-
P09601	HMOX1	32.81	6.39	-
Q16831	UPP1	33.93	5.89	-

Chemistry Methods

All chemical reagents and solvents were purchased from commercial sources and used without purification. Thin-layer chromatography (TLC) was carried out using silica gel plates coated with fluorescence F-254. Product spots were visualized under UV light (λ = 245 nm or 365 nm). Normal-phase silica gel chromatography was carried out using a Biotage Isolera One flash column chromatography system. ^{1}H NMR and ^{13}C NMR were recorded at 400 MHz, 101 MHz, respectively, using a Bruker AV-400 spectrometer in the deuterated solvent specified. Chemical shifts are reported as δ values (parts per million) relative to the residual nondeuterated solvent signal as an internal reference. Coupling constants (*J*) are reported in Hertz. The multiplicity was defined as singlet (s), doublet (d), triplet (t), broad (br) or multiplet (m). Highresolution mass spectra (HRMS) was measured by the micrOTOFQ II 10203 mass spectrometer with AP-ESI ion source and an Agilent 1200-G6410A mass spectrometer. All the final compounds were purified to > 95% purity, as determined by HPLC. HPLC analyses were performed on a Waters e2695 HPLC system with a Symmetry C18 reversed-phase column (4.6 mm × 250 mm, 5 μm).

Synthesis of compounds 1a-1j.

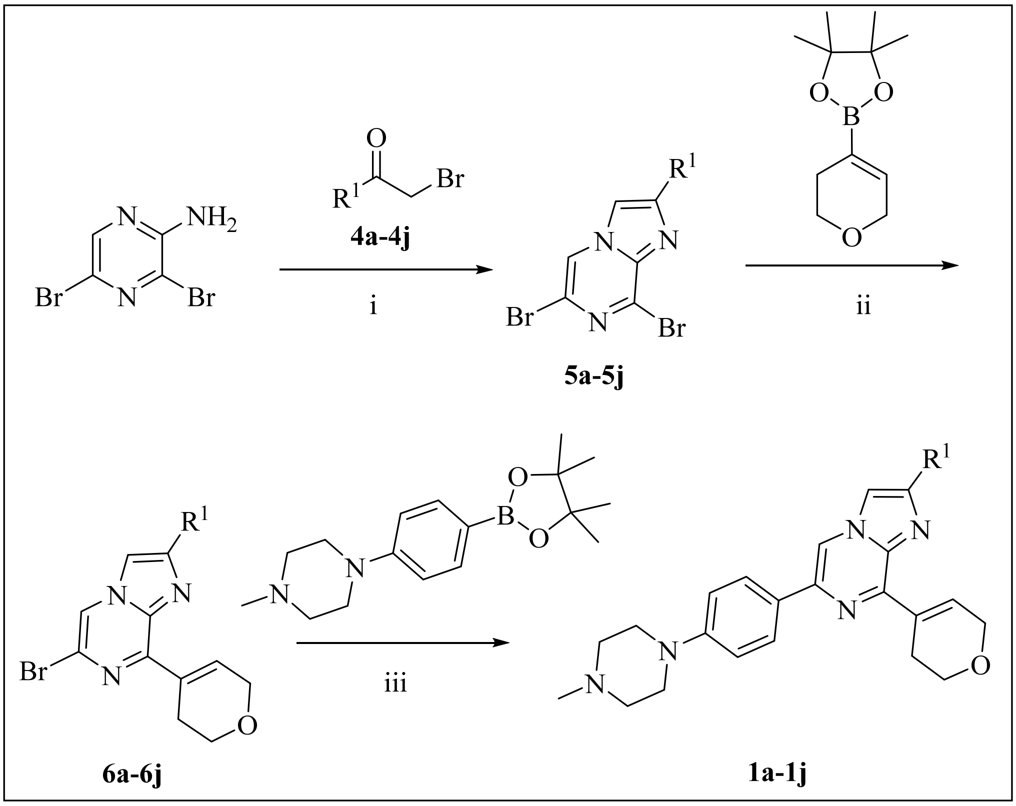

Reagents and conditions: (i) 1,4-Dioxane, 120°C, 8 h, 80-95%; (ii) Pd(dppf)Cl_2, K_3PO_4, 1,4-dioxane/H_2O (4/1), Ar, 80°C, 12 h, 30-60%, (iii) Pd(dppf)Cl_2, Cs_2CO_3, 1,4-dioxane/H_2O (4/1), Ar, 80°C, 12 h, 50-70%.

General procedure for the synthesis of intermediates 5a-5j. The 2-amino-3,5-

dibromopyrazine (10 mmol) and commercially available reagents **4a-4j** (10 mmol) were added to a flask in 1,4-dioxane (10 mL) at 120°C. The reaction mixture was stirred until a large amount of yellow solid was gradually separated out, and then the mixture was filtered, the solid was washed with 1,4-dioxane (3 mL × 2) and dried to obtain the **5a-5j** as yellow solid.

General procedure for the synthesis of intermediates 6a-6j. The intermediate **5a-5j** (5 mmol), commercially available reagent 3,6-dihydro-2*H*-pyran-4-boronic acid pinacol ester (4.5 mmol), Pd(dppf)Cl_2 (0.5 mmol), and K_3PO_4 (15 mmol) were added to a flask in 1,4-dioxane/H_2O (20 mL/5 mL) under an atmosphere of nitrogen, and stirred for 4 h at 80°C. After the reaction was complete as indicated by TLC, the reaction was cooled to room temperature, and the reaction mixture was washed with brine (25 mL), the aqueous phase was extracted with ethyl acetate (3 × 20 mL). The combined organic layer was dried over anhydrous Na_2SO_4 and filtered. The organic solution was concentrated in vacuo and the residue was purified by column chromatography to give intermediate **6a-6j** as white solid.

General procedure for the synthesis of compounds 1a-1j. The intermediate **6a-6j** (2 mmol), commercially available reagent 4-(4-methyl-1-piperazinyl) benzeneboronic acid pinacol ester (2 mmol), Pd(dppf)Cl_2 (0.2 mmol), and CS_2CO_3 (6 mmol) were added to a flask in 1,4-dioxane/H_2O (20 mL/5 mL) under an atmosphere of nitrogen, and stirred for 4 h at 80°C. After the reaction was complete as indicated by TLC, the reaction was cooled to room temperature, and the reaction mixture was washed with brine (25 mL), and the aqueous phase was extracted with ethyl acetate (3 × 20 mL). The combined organic layer was dried over anhydrous Na_2SO_4. After filtration, the organic solution was concentrated in vacuo and the residue was purified by column chromatography to give **1a-1j**.

Synthesis of compounds 2a-2j.

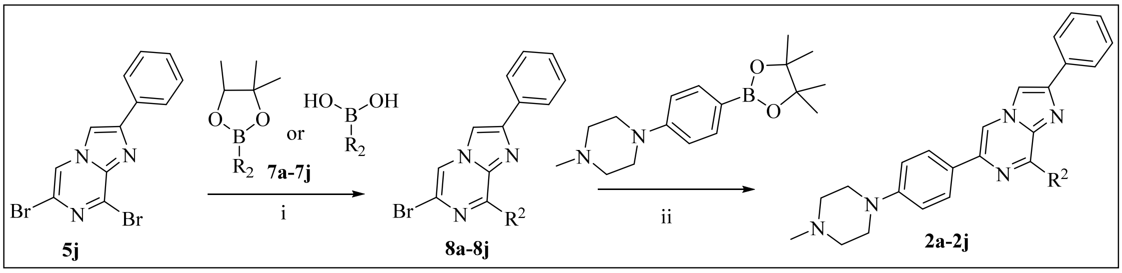

Reagents and conditions: (i) Pd(dppf)Cl_2, K_3PO_4, 1,4-dioxane/H_2O (4/1), Ar, 80°C, 12 h, 30-60%, (ii) Pd(dppf)Cl_2, Cs_2CO_3, 1,4-dioxane/H_2O (4/1), Ar, 80°C, 12 h, 50-70%.

General procedure for the synthesis of intermediates 8a-8j. The intermediate **5j** (5 mmol), commercially available reagents **7a-7j** (4.5 mmol), Pd(dppf)Cl_2 (0.5 mmol),

and K_3PO_4 (15 mmol) were added to a flask in 1,4-dioxane/H_2O (20 mL/5 mL) under an atmosphere of nitrogen, and stirred for 4 h at 80°C. After the reaction was complete as indicated by TLC, the reaction was cooled to room temperature, and the reaction mixture was washed with brine (25 mL), the aqueous phase was extracted with ethyl acetate (3 × 20 mL). The organic layer was dried over anhydrous Na_2SO_4. After filtration, the combined organic solution was concentrated in vacuo and the residue was purified by column chromatography to give intermediate **8a-8j** as white solid.

General procedure for the synthesis of compounds 2a-2j. The intermediates **8a-8j** (2 mmol), commercially available reagent 4-(4-methyl-1-piperazinyl) benzeneboronic acid pinacol ester (2 mmol), Pd(dppf)Cl_2 (0.2 mmol), and CS_2CO_3 (6 mmol) were added to a flask in 1,4-dioxane/H_2O (20 mL/5 mL) under an atmosphere of nitrogen, and stirred for 4 h at 80°C. After the reaction was complete as indicated by TLC, the reaction was cooled to room temperature, and the reaction mixture was washed with brine (25 mL), and the aqueous phase was extracted with ethyl acetate (3 × 20 mL). The combined organic layer was dried over anhydrous Na_2SO_4. After filtration, the organic solution was concentrated in vacuo and the residue was purified by column chromatography to give **2a-2j**.

Synthesis of compounds 3a-3f and YL-939.

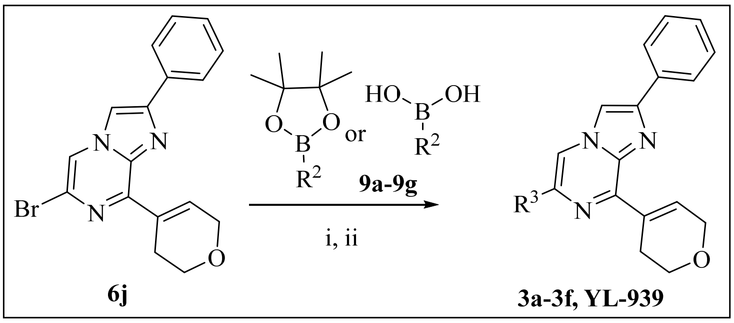

Reagents and conditions: (i) Pd(dppf)Cl_2, Cs_2CO_3, 1,4-dioxane/H_2O (4/1), Ar, 80°C, 12 h, 50-70%, (ii) HCl (4 M in 1,4-dioxane).

General procedure for the synthesis of compounds 3a-3f. The intermediate **6j** (2 mmol), commercially available reagents **9a-9f** (2 mmol), Pd(dppf)Cl_2 (0.2 mmol), and CS_2CO_3 (6 mmol) were added to a flask in 1,4-dioxane/H_2O (20 mL/5 mL) under an atmosphere of nitrogen, and stirred for 4 h at 80°C. After the reaction was complete as indicated by TLC, the reaction was cooled to room temperature, and the reaction mixture was washed with brine (25 mL), the aqueous phase was extracted with ethyl acetate (3 × 20 mL). The organic layer was dried over anhydrous Na_2SO_4. After filtration, the combined organic solution was concentrated in vacuo and the residue was purified by column chromatography to give **3a-3f**.

General procedure for the synthesis of compound YL-939. The intermediate **6j** (2 mmol), commercially available reagent tert-Butyl 4-[4-(4,4,5,5-tetramethyl-1,3,2-dioxaborolan-2-yl)-1*H*-pyrazol-1-yl]piperidine-1-carboxylate (**9g**) (2 mmol), Pd(dppf)Cl_2 (0.2 mmol), and CS_2CO_3 (6 mmol) were added to a flask in 1,4-dioxane/H_2O (20 mL/5 mL) under an atmosphere of nitrogen, and stirred for 4 h at 80°C. After the reaction was complete as indicated by TLC, the reaction was cooled to room temperature, and the reaction mixture was washed with brine (25 mL), the aqueous phase was extracted with ethyl acetate (3 × 25 mL). The organic layer was dried over anhydrous Na_2SO_4. After filtration, the organic solution was concentrated in vacuo and the residue was purified by column chromatography. The intermediate dissolved with HCl (4 M in 1,4-dioxane), After the reaction was complete as indicated by TLC, the reaction was adjusted to PH ≈ 9. And then, the mixture was washed with brine (25 mL), the aqueous phase was extracted with dichloromethane (3 × 20 mL), the combined organic layer was dried over Na_2SO_4. After filtration, the combined organic solution was concentrated in vacuo and the residue was purified by column chromatography to give compound 8-(3,6-dihydro-2*H*-pyran-4-yl)-2-phenyl-6-(1-(piperidin-4-yl)-1*H*-pyrazol-4-yl)imidazo[1,2-*a*]pyrazine (YL-939). White solid, 41% yield. ^{1}H NMR (400 MHz, DMSO-d_6) δ 8.76 (s, 1H), 8.50 (s, 1H), 8.39 (s, 1H), 8.28 (s, 1H), 8.07 (d, *J* = 7.2 Hz, 2H), 8.00 (s, 1H), 7.49 (t, *J* = 7.6 Hz, 2H), 7.38 (t, *J* = 7.3 Hz, 1H), 4.47 (d, *J* = 2.4 Hz, 2H), 4.32 (m, 1H), 3.92 (t, *J* = 5.4 Hz, 2H), 3.13 (d, *J* = 12.5 Hz, 2H), 2.82 (s, 2H), 2.69 (t, *J* = 11.3 Hz, 2H), 2.03 (dd, *J* = 12.2, 2.2 Hz, 2H), 1.88 (qd, *J* = 12.9, 3.9 Hz, 2H).^{13}C NMR (101 MHz, DMSO-d_6) δ 146.60, 145.73, 137.76, 136.30, 136.10, 133.51, 132.85, 132.26, 129.27, 128.78, 126.41, 126.30, 119.99, 112.91, 110.95, 65.57, 64.40, 59.42, 45.32, 33.61, 25.37. HRMS *m/z* (ESI) calcd for $C_{25}H_{26}N_6O$ $[M+H]^+$ 427.2241 found: 427.2239.

Synthesis of probe YL-939-1.

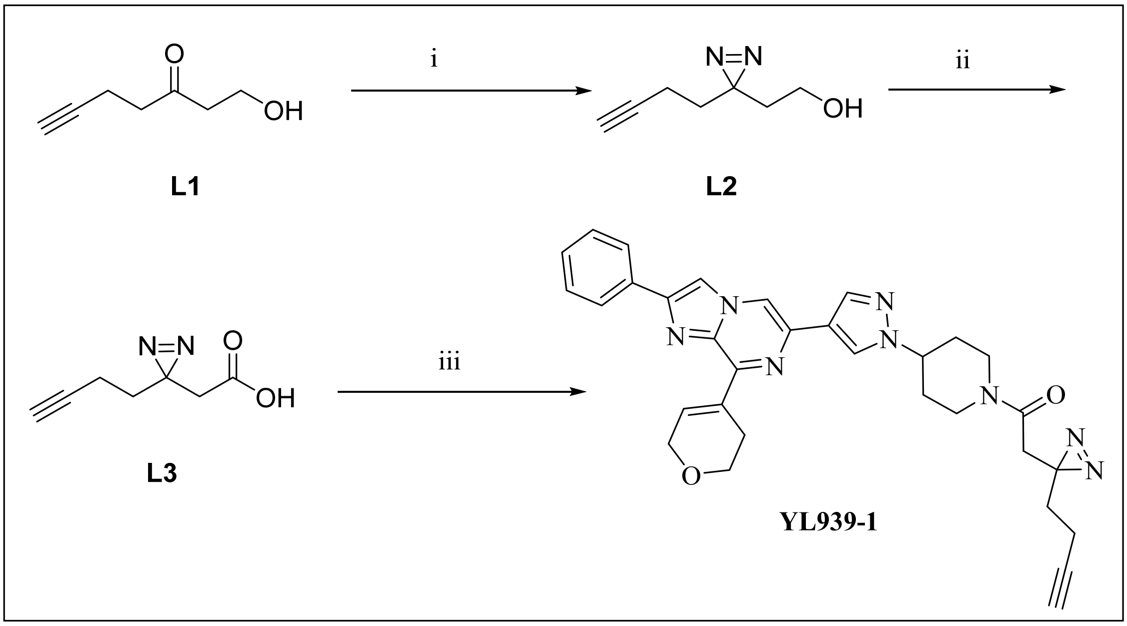

Reagents and conditions: (i) (1) NH_3, NH_2SO_3H (2) TEA, I_2, (ii) Jones reagent, (iii) HATU, DIEA, YL-939, DCM.

Step 1: Added NH_3 (7 M in MeOH; 35.71 mmol) to a three-neck flask containing commercially available 1-hydroxyhept-6-yn-3-one (L1) (2.38 mmol) at -10°C in argon atmosphere, and stirred for 4.5 h at -10°C. Afterwards, added hydroxylamine-O-sulfonic acid (8.84 mmol) which dissolved in anhydrous methanol and the reaction mixture was stirred at -10°C for 1 h. Then, the reaction mixture was stirred at room temperature for 16 h. Removal of NH_3 by blowing argon into the bottle. The reaction mixture was filtered with diatomite and washed with methanol. Then extracted solution was placed in 0°C with Et_3N (17.61 mmol), a solution of I_2 (3.09 mmol) in anhydrous MeOH (5 mL) was added dropwise at 0 °C and the mixture was stirred at 0°C for 1 h. The mixture was washed with brine (25 mL), the aqueous phase was extracted with Et_2O (3 × 25 mL), the combined organic layer was dried over Na_2SO_4, filtered and the solvent was removed under reduced pressure. The residue was purified by flash column chromatography on silica to afford 2-(3-(but-3-yn-1-yl)-3*H*-diazirin-3-yl)ethan-1-ol (L2) as pale yellow oil, 23 % yield, ^1H NMR (400 MHz, $CDCl_3$) δ 3.50 (dd, J = 11.3, 5.9 Hz, 2H), 2.05 (td, J = 7.4, 2.5 Hz, 2H), 2.00 (t, J = 2.6 Hz, 1H), 1.71 (dt, J = 11.2, 6.8 Hz, 4H), 1.43 (t, J = 5.0 Hz, 1H).

Step 2: Jones reagent (2 M CrO_3 in H_2SO_4, 2.60 mmol) was added dropwise to an acetone solution of L2 (0.47mmol) at 0°C. The reaction mixture was stirred at room temperature for 1 h, and then quenched with isopropanol (5 mL) and filtered with diatomite and washed with acetone (3 × 20 mL). The combined organic layer was dried over Na_2SO_4, filtered and the solvent was removed under reduced pressure. The residue was purified by flash column chromatography on silica to give 2-(3-(but-3-yn-1-yl)-

3*H*-diazirin-3-yl)acetic acid (L3) as pale yellow oil, 88 % yield, ^{1}H NMR (400 MHz, $CDCl_3$) δ 2.41 (d, *J* = 2.0 Hz, 2H), 2.08 (m, 2H), 2.02 (dd, *J* = 4.8, 2.4 Hz, 1H), 1.81 (td, *J* = 7.3, 1.9 Hz, 2H).

Step 3: YL-939 (0.38 mmol), L3 (0.38 mmol), HATU (0.46 mmol), and DIEA (1.14 mmol) were added to a flask in Dichloromethane (10 mL). The reaction was stirred for 2 h at room temperature and detected by TLC. After completed, the mixture was washed with brine (25 mL), the aqueous phase was extracted with dichloromethane (3 × 25 mL), the combined organic layer was dried over Na_2SO_4, filtered and the solvent was removed under reduced pressure. The residue was purified by flash column chromatography on silica to afford 2-(3-(but-3-yn-1-yl)-3*H*-diazirin-3-yl)-1-(4-(4- (8-(3,6-dihydro-2*H*-pyran-4-yl)-2-phenylimidazo[1,2-*a*]pyrazin-6-yl)-1H-pyrazol-1-yl)piperidin-1-yl)ethan-1-one (YL-939-1). White solid, 32% yield. ^{1}H NMR (400 MHz, DMSO-*d*$_6$) δ 8.76 (s, 1H), 8.49 (s, 1H), 8.38 (s, 1H), 8.31 (s, 1H), 8.07 (d, *J* = 7.4 Hz, 2H), 8.01 (s, 1H), 7.49 (t, *J* = 7.6 Hz, 2H), 7.39 (t, *J* = 7.3 Hz, 1H), 4.52 (m, 1H), 4.47 (d, *J* = 2.0 Hz, 2H), 3.92 (t, *J* = 5.3 Hz, 2H), 3.82 (d, *J* = 13.3 Hz, 1H), 3.19 (t, *J* = 12.1 Hz, 1H), 2.85 (m, 1H), 2.82 (s, 2H), 2.74 (d, *J* = 11.4 Hz, 1H), 2.68 (d, *J* = 6.9 Hz, 1H), 2.63 (d, *J* = 7.7 Hz, 2H), 2.05 (td, *J* = 7.4, 2.4 Hz, 4H), 1.96 (ddd, *J* = 15.6, 12.3, 4.0 Hz, 1H), 1.80 (m, 1H), 1.70 (td, *J* = 7.3, 4.1 Hz, 2H). ^{13}C NMR (101 MHz, DMSO-*d*$_6$) δ 166.69, 146.62, 145.75, 137.77, 136.46, 136.13, 133.50, 132.76, 132.25, 129.27, 128.78, 126.67, 126.42, 120.19, 112.98, 110.98, 83.71, 72.25, 65.58, 64.40, 58.55, 44.63, 38.70, 37.74, 32.94, 32.31, 25.38, 13.04. HRMS *m/z* (ESI) calcd for $C_{32}H_{32}N_8O_2$ $[M+H]^+$ 561.2721 found: 561.2717.

.

Characterization of compounds

8-(3,6-dihydro-2*H*-pyran-4-yl)-2-(4-methoxyphenyl)-6-(4-(4-methylpiperazin-1-yl)phenyl)imidazo[1,2-*a*]pyrazine (Cpd-015). Yellow solid, 47% yield. ^{1}H NMR (400 MHz, $CDCl_3$) δ 8.40 (d, *J* = 0.9 Hz, 1H), 8.14 (s, 1H), 7.91 (d, *J* = 7.8 Hz, 2H), 7.86 (d, *J* = 7.9 Hz, 2H), 7.75 (s, 1H), 6.98 (t, *J* = 7.0 Hz, 4H), 4.55 (s, 2H), 4.02 (t, *J* = 5.1 Hz, 2H), 3.85 (d, *J* = 0.8 Hz, 3H), 3.28 (d, *J* = 4.3 Hz, 4H), 2.96 (s, 2H), 2.60 (m, 4H), 2.37 (s, 3H). ^{13}C NMR (101 MHz, $CDCl_3$) δ 159.92, 151.11, 146.70, 146.42, 138.20, 137.95, 135.43, 132.74, 127.74, 127.57, 126.79, 126.01, 115.79, 114.19, 111.49, 108.46, 65.98, 64.93, 55.34, 54.98, 48.64, 46.20, 25.45. HRMS *m/z* (ESI) calcd for $C_{29}H_{31}N_5O_2$ $[M+H]^+$ 482.2551 found:482.2551.

2-(4-chlorophenyl)-8-(3,6-dihydro-2*H*-pyran-4-yl)-6-(4-(4-methylpiperazin-1-yl)phenyl)imidazo[1,2-*a*]pyrazine (**1a**). Yellow solid, 53% yield. ^{1}H NMR (400 MHz, $CDCl_3$) δ 8.41 (s, 1H), 8.23 (s, 1H), 7.95 (s, 1H), 7.93 (s, 1H), 7.91 (d, *J* = 2.4 Hz, 2H), 7.89 (s, 1H), 7.43 (d, *J* = 8.5 Hz, 2H), 7.02 (d, *J* = 8.8 Hz, 2H), 4.58 (d, *J* = 2.6 Hz, 2H), 4.04 (t, *J* = 5.4 Hz, 2H), 3.31 (m, 4H), 2.98 (s, 2H), 2.61 (m, 4H), 2.37 (s, 3H). ^{13}C

NMR (101 MHz, $CDCl_3$) δ 151.52, 147.20, 145.34, 138.37, 138.30, 135.73, 134.18, 132.95, 132.72, 131.82, 128.98, 127.48, 126.87, 115.78, 111.42, 109.29, 65.95, 64.88, 54.96, 48.59, 46.13, 24.87. HRMS *m/z* (ESI) calcd for $C_{28}H_{28}ClN_5O$ $[M+H]^+$ 486.2055 found: 486.2056.

8-(3,6-dihydro-2*H*-pyran-4-yl)-2-(4-fluorophenyl)-6-(4-(4-methylpiperazin-1-yl)phenyl)imidazo[1,2-*a*]pyrazine (**1b**). Yellow solid, 42% yield. ^1H NMR (400 MHz, $CDCl_3$) δ 8.41 (s, 1H), 8.24 (s, 1H), 7.98 (m, 2H), 7.89 (m, 3H), 7.15 (m, 2H), 7.03 (d, *J* = 8.9 Hz, 2H), 4.58 (d, *J* = 2.7 Hz, 2H), 4.04 (t, *J* = 5.5 Hz, 2H), 3.31 (m, 4H), 2.98 (d, *J* = 1.6 Hz, 2H), 2.62 (m, 4H), 2.38 (s, 3H). ^{13}C NMR (101 MHz, $CDCl_3$) δ 162.97(d, *J* = 249.5 Hz), 151.48, 147.01, 145.54, 138.22(d, *J* = 2.0 Hz), 135.63, 132.71, 129.51(d, *J* = 3.0 Hz), 128.00, 127.92, 127.50, 126.81, 115.75(d, *J* = 21.2 Hz), 115.74, 111.43, 108.93, 65.94, 64.88, 54.98, 48.61, 46.17, 25.42. HRMS *m/z* (ESI) calcd for $C_{28}H_{28}FN_5O$ $[M+H]^+$ 470.2351 found: 470.2353.

4-(8-(3,6-dihydro-2*H*-pyran-4-yl)-6-(4-(4-methylpiperazin-1-yl)phenyl)imidazo[1,2-*a*]pyrazin-2-yl)phenol (**1c**). Yellow solid, 48% yield. ^1H NMR (400 MHz, DMSO-d_6) δ 9.71 (s, 1H), 8.84 (s, 1H), 8.39 (s, 1H), 8.29 (s, 1H), 7.88 (dd, *J* = 11.4, 8.7 Hz, 4H), 7.02 (d, *J* = 8.7 Hz, 2H), 6.88 (d, *J* = 8.4 Hz, 2H), 4.44 (s, 2H), 3.91 (t, *J* = 5.1 Hz, 2H), 3.20 (s, 4H), 2.81 (s, 2H), 2.47 (s, 4H), 2.23 (s, 3H). ^{13}C NMR (101 MHz, DMSO-d_6) δ 158.31, 151.37, 146.39, 145.57, 137.72, 137.02, 135.83, 132.46, 127.88, 127.09, 126.81, 124.55, 116.09, 115.52, 113.14, 109.66, 65.56, 64.41, 54.91, 48.04, 46.14, 25.51. HRMS *m/z* (ESI) calcd for $C_{28}H_{29}N_5O_2$ $[M+H]^+$ 468.2394 found: 468.2398.

8-(3,6-dihydro-2*H*-pyran-4-yl)-6-(4-(4-methylpiperazin-1-yl)phenyl)-2-(p-tolyl)imidazo[1,2-*a*]pyrazine (**1d**). White solid, 51% yield. ^1H NMR (400 MHz, $CDCl_3$) δ 8.43 (s, 1H), 8.23 (s, 1H), 7.89 (m, 5H), 7.28 (m, 1H), 7.25 (m, 1H), 7.02 (d, *J* = 8.4 Hz, 2H), 4.58 (d, *J* = 2.1 Hz, 2H), 4.04 (t, *J* = 5.3 Hz, 2H), 3.30 (m, 4H), 2.98 (s, 2H), 2.61 (m, 4H), 2.41 (s, 3H), 2.37 (s, 3H). ^{13}C NMR (101 MHz, $CDCl_3$) δ 151.37, 146.79, 146.51, 138.28, 138.15, 137.90, 135.52, 132.69, 130.48, 129.46, 127.66, 126.76, 126.12, 115.71, 111.45, 108.95, 65.96, 64.90, 54.97, 48.61, 46.16, 25.43, 21.41. HRMS *m/z* (ESI) calcd for $C_{29}H_{31}N_5O$ $[M+H]^+$ 466.2601 found: 466.2602.

2-([1,1'-biphenyl]-4-yl)-8-(3,6-dihydro-2*H*-pyran-4-yl)-6-(4-(4-methylpiperazin-1-yl)phenyl)imidazo[1,2-*a*]pyrazine (**1e**). Yellow solid, 49% yield. ^1H NMR (400 MHz, $CDCl_3$) δ 8.47 (s, 1H), 8.26 (s, 1H), 8.08 (d, *J* = 8.3 Hz, 2H), 7.96 (s, 1H), 7.91 (d, *J* = 8.8 Hz, 2H), 7.70 (d, *J* = 8.3 Hz, 2H), 7.66 (d, *J* = 7.3 Hz, 2H), 7.47 (t, *J* = 7.6 Hz, 2H), 7.37 (t, *J* = 7.3 Hz, 1H), 7.03 (d, *J* = 8.8 Hz, 2H), 4.59 (d, *J* = 2.5 Hz, 2H), 4.05 (t, *J* = 5.4 Hz, 2H), 3.31 (m, 4H), 3.00 (s, 2H), 2.61 (m, 4H), 2.38 (s, 3H).^{13}C NMR (101 MHz,

$CDCl_3$) δ 151.45, 147.07, 146.13, 141.10, 140.67, 138.34, 138.18, 135.69, 132.74, 132.27, 128.86, 127.62, 127.45, 127.01, 126.84, 126.65, 115.78, 111.48, 109.33, 65.99, 64.91, 54.97, 48.61, 46.14, 25.45. HRMS *m/z* (ESI) calcd for $C_{34}H_{33}N_5O$ $[M+H]^+$ 528.2758 found:528.2756.

8-(3,6-dihydro-2*H*-pyran-4-yl)-6-(4-(4-methylpiperazin-1-yl)phenyl)-2-(naphthalen-2-yl)imidazo[1,2-*a*]pyrazine (**1f**). Yellow solid, 47% yield. ^1H NMR (400 MHz, $CDCl_3$) δ 8.49 (d, *J* = 3.6 Hz, 2H), 8.23 (s, 1H), 8.07 (d, *J* = 8.5 Hz, 1H), 8.00 (s, 1H), 7.92 (m, 4H), 7.86 (d, *J* = 7.9 Hz, 1H), 7.50 (m, 2H), 7.02 (d, *J* = 8.5 Hz, 2H), 4.61 (s, 2H), 4.05 (t, *J* = 5.3 Hz, 2H), 3.30 (m, 4H), 3.00 (s, 2H), 2.60 (m, 4H), 2.37 (s, 3H). ^{13}C NMR (101 MHz, $CDCl_3$) δ 151.41, 146.94, 146.27, 138.29, 138.03, 135.65, 133.61, 133.39, 132.72, 130.61, 128.38, 127.81, 127.54, 126.78, 126.39, 126.19, 125.05, 124.19, 115.70, 111.41, 109.67, 66.01, 64.90, 54.97, 48.59, 46.16, 25.44. HRMS *m/z* (ESI) calcd for $C_{33}H_{31}N_5O$ $[M+H]^+$ 502.2601 found: 502.2587.

8-(3,6-dihydro-2*H*-pyran-4-yl)-2-(3-methoxyphenyl)-6-(4-(4-methylpiperazin-1-yl)phenyl)imidazo[1,2-*a*]pyrazine (**1g**). Yellow solid, 43% yield. ^1H NMR (400 MHz, $CDCl_3$) δ 8.43 (s, 1H), 8.23 (s, 1H), 7.90 (t, *J* = 4.4 Hz, 3H), 7.57 (dd, *J* = 10.2, 5.0 Hz, 2H), 7.37 (t, *J* = 7.9 Hz, 1H), 7.02 (d, *J* = 8.9 Hz, 2H), 6.92 (dd, *J* = 8.2, 1.8 Hz, 1H), 4.57 (d, *J* = 2.6 Hz, 2H), 4.04 (t, *J* = 5.4 Hz, 2H), 3.91 (s, 3H), 3.31 (m, 4H), 2.98 (d, *J* = 1.4 Hz, 2H), 2.61 (m, 4H), 2.38 (s, 3H). ^{13}C NMR (101 MHz, $CDCl_3$) δ 160.03, 151.44, 147.05, 146.22, 138.16, 138.09, 135.66, 134.70, 132.67, 129.80, 127.57, 126.80, 118.76, 115.73, 113.75, 111.98, 111.45, 109.52, 65.96, 64.89, 55.39, 54.98, 48.62, 46.17, 25.43. HRMS *m/z* (ESI) calcd for $C_{33}H_{31}N_5O$ $[M+H]^+$ 482.2551 found: 482.2552.

8-(3,6-dihydro-2*H*-pyran-4-yl)-6-(4-(4-methylpiperazin-1-yl)phenyl)-2-(3-nitrophenyl)imidazo[1,2-*a*]pyrazine (**1h**). Yellow solid, 52% yield. ^1H NMR (400 MHz, $CDCl_3$) δ 8.80 (m, 1H), 8.43 (s, 1H), 8.37 (d, *J* = 7.8 Hz, 1H), 8.26 (s, 1H), 8.21 (dd, *J* = 8.2, 1.3 Hz, 1H), 8.04 (s, 1H), 7.91 (d, *J* = 8.8 Hz, 2H), 7.64 (t, *J* = 8.0 Hz, 1H), 7.04 (d, *J* = 8.9 Hz, 2H), 4.60 (d, *J* = 2.6 Hz, 2H), 4.05 (t, *J* = 5.4 Hz, 2H), 3.32 (m, 4H), 2.99 (s, 2H), 2.62 (m, 4H), 2.38 (s, 3H). ^{13}C NMR (101 MHz, $CDCl_3$) δ 151.60, 148.76, 147.49, 143.91, 138.68, 138.38, 136.09, 135.17, 132.67, 132.01, 129.75, 127.17, 126.90, 122.88, 120.93, 115.74, 111.39, 110.10, 65.98, 64.85, 54.93, 48.50, 46.11, 25.41. HRMS *m/z* (ESI) calcd for $C_{28}H_{28}N_6O_3$ $[M+H]^+$ 497.2296 found:497.2297.

8-(3,6-dihydro-2*H*-pyran-4-yl)-2-(2-methoxyphenyl)-6-(4-(4-methylpiperazin-1-yl)phenyl)imidazo[1,2-*a*]pyrazine (**1i**). Yellow solid, 42% yield. ^1H NMR (400 MHz, $CDCl_3$) δ 8.48 (d, *J* = 7.9 Hz, 2H), 8.26 (s, 2H), 7.91 (d, *J* = 8.3 Hz, 2H), 7.34 (t, *J* =

7.8 Hz, 1H), 7.13 (t, J = 7.5 Hz, 1H), 7.02 (d, J = 8.5 Hz, 3H), 4.59 (d, J = 1.8 Hz, 2H), 4.04 (t, J = 5.4 Hz, 2H), 4.02 (s, 3H), 3.31 (m, 4H), 2.99 (s, 2H), 2.63 (m, 4H), 2.39 (s, 3H). ^{13}C NMR (101 MHz, $CDCl_3$) δ 157.01, 151.34, 146.75, 142.09, 137.61, 137.06, 135.42, 132.80, 129.15, 129.11, 127.92, 126.77, 121.93, 121.04, 115.80, 113.62, 111.59, 110.89, 66.00, 64.93, 55.43, 55.02, 48.70, 46.18, 25.45. HRMS *m/z* (ESI) calcd for $C_{33}H_{31}N_5O$ $[M+H]^+$ 482.2551 found: 482.2554.

8-(3,6-dihydro-2*H*-pyran-4-yl)-6-(4-(4-methylpiperazin-1-yl)phenyl)-2-phenylimidazo[1,2-*a*]pyrazine (**1j**). Yellow solid, 58% yield. ^{1}H NMR (400 MHz, DMSO-d_6) δ 8.93 (s, 1H), 8.51 (s, 1H), 8.43 (s, 1H), 8.07 (d, J = 7.5 Hz, 2H), 7.95 (d, J = 8.7 Hz, 2H), 7.50 (t, J = 7.6 Hz, 2H), 7.39 (t, J = 7.3 Hz, 1H), 7.07 (d, J = 8.9 Hz, 2H), 4.48 (d, J = 2.0 Hz, 2H), 3.94 (t, J = 5.4 Hz, 2H), 3.23 (m, 4H), 2.85 (s, 2H), 2.48 (d, J = 4.9 Hz, 4H), 2.24 (s, 3H). ^{13}C NMR (101 MHz, $CDCl_3$) δ 151.42, 147.06, 146.45, 138.26, 138.10, 135.66, 133.28, 132.69, 128.79, 128.42, 127.64, 126.82, 126.26, 115.77, 111.50, 109.28, 65.97, 64.90, 54.97, 48.60, 46.14, 25.43. HRMS *m/z* (ESI) calcd for $C_{28}H_{29}N_5O$ $[M+H]^+$ 452.2445 found:452.2440.

4-(6-(4-(4-methylpiperazin-1-yl)phenyl)-2-phenylimidazo[1,2-*a*]pyrazin-8-yl)pyridin-2-amine (**2a**). Yellow solid, 54% yield. ^{1}H NMR (400 MHz, DMSO-d_6) δ 9.10 (s, 1H), 8.61 (s, 1H), 8.16 (dd, J = 6.0, 4.7 Hz, 3H), 8.10 (s, 1H), 8.02 (d, J = 8.8 Hz, 2H), 7.95 (dd, J = 5.4, 1.3 Hz, 1H), 7.53 (t, J = 7.6 Hz, 2H), 7.42 (t, J = 7.3 Hz, 1H), 7.09 (d, J = 8.9 Hz, 2H), 6.20 (s, 2H), 3.25 (m, 4H), 2.48 (m, 4H), 2.24 (s, 3H). ^{13}C NMR (101 MHz, DMSO-d_6) δ 160.71, 151.30, 148.37, 146.73, 144.83, 144.38, 138.45, 137.88, 133.40, 129.33, 129.06, 127.08, 126.88, 126.70, 115.66, 115.06, 111.79, 111.63, 108.61, 54.48, 47.55, 45.50. HRMS *m/z* (ESI) calcd for $C_{28}H_{27}N_7$ $[M+H]^+$ 462.2401 found:462.2402.

5-(6-(4-(4-methylpiperazin-1-yl)phenyl)-2-phenylimidazo[1,2-*a*]pyrazin-8-yl)picolinonitrile (**2b**). Yellow solid, 39% yield. ^{1}H NMR (400 MHz, DMSO-d_6) δ 10.23 (d, J = 1.3 Hz, 1H), 9.54 (dd, J = 8.2, 2.1 Hz, 1H), 9.18 (s, 1H), 8.66 (s, 1H), 8.31 (d, J = 8.6 Hz, 1H), 8.14 (d, J = 7.3 Hz, 2H), 8.04 (d, J = 8.8 Hz, 2H), 7.54 (t, J = 7.4 Hz, 2H), 7.43 (t, J = 7.5 Hz, 1H), 7.10 (d, J = 8.9 Hz, 2H), 3.26 (m, 4H), 2.47 (s, 4H), 2.24 (s, 3H). ^{13}C NMR (101 MHz, $CDCl_3$) δ 151.85, 151.70, 147.81, 142.43, 138.97, 138.16, 137.39, 134.55, 133.78, 132.50, 129.05, 128.92, 127.85, 126.82, 126.32, 126.27, 117.53, 115.58, 113.16, 109.80, 77.38, 77.06, 76.75, 48.31, 46.12, 24.88. HRMS *m/z* (ESI) calcd for $C_{27}H_{25}N_7$ $[M+H]^+$ 472.2244 found:472.2239.

8-(6-ethoxypyridin-3-yl)-6-(4-(4-methylpiperazin-1-yl)phenyl)-2-phenylimidazo[1,2-*a*]pyrazine (**2c**). Yellow solid, 41% yield. ^{1}H NMR (400 MHz, DMSO-d_6) δ 9.90 (d, J

= 2.3 Hz, 1H), 9.15 (dd, *J* = 8.8, 2.4 Hz, 1H), 9.02 (s, 1H), 8.59 (s, 1H), 8.12 (d, *J* = 7.4 Hz, 2H), 8.02 (d, *J* = 8.8 Hz, 2H), 7.53 (t, *J* = 7.6 Hz, 2H), 7.41 (t, *J* = 7.4 Hz, 1H), 7.08 (m, 2H), 4.46 (q, *J* = 7.0 Hz, 2H), 3.25 (m, 4H), 2.47 (m, 4H), 2.24 (s, 3H), 1.40 (t, *J* = 7.0 Hz, 3H). ^{13}C NMR (101 MHz, $CDCl_3$) δ 164.93, 151.46, 149.52, 147.05, 145.41, 139.44, 138.74, 138.26, 133.16, 128.76, 128.51, 127.44, 126.90, 126.34, 125.76, 115.69, 111.75, 110.60, 109.60, 62.13, 48.57, 46.16, 24.88, 14.74. HRMS *m/z* (ESI) calcd for $C_{30}H_{30}N_6O$ $[M+H]^+$ 491.2554 found:491.2551.

8-(3-fluoropyridin-4-yl)-6-(4-(4-methylpiperazin-1-yl)phenyl)-2-phenylimidazo[1,2-*a*]pyrazine (**2d**). Yellow solid, 43% yield. ^{1}H NMR (400 MHz, DMSO-d_6) δ 9.17 (s, 1H), 8.85 (d, *J* = 2.0 Hz, 1H), 8.70 (d, *J* = 4.8 Hz, 1H), 8.65 (s, 1H), 8.18 (m, 1H), 8.03 (d, *J* = 7.3 Hz, 2H), 7.95 (d, *J* = 8.8 Hz, 2H), 7.49 (t, *J* = 7.5 Hz, 2H), 7.39 (t, *J* = 7.3 Hz, 1H), 7.08 (d, *J* = 8.9 Hz, 2H), 3.24 (m, 4H), 2.47 (m, 4H), 2.24 (s, 3H). ^{13}C NMR (101 MHz, $CDCl_3$) δ 157.17(d, *J* = 265.6 Hz), 151.66, 148.15, 145.58 (d, *J* = 3.0 Hz), 143.94 (d, *J* = 2.0 Hz), 139.71, 139.53, 139.46, 138.53, 132.82, 131.54 (d, *J* = 9.1 Hz), 128.85, 127.04, 126.69, 126.48, 125.67, 115.70, 113.32, 110.04, 77.39, 77.07, 76.75, 54.94, 48.46, 46.17. HRMS *m/z* (ESI) calcd for $C_{28}H_{25}FN_6$ $[M+H]^+$ 465.2197 found:465.2188.

8-(2,6-dimethylpyridin-4-yl)-6-(4-(4-methylpiperazin-1-yl)phenyl)-2-phenylimidazo[1,2-*a*]pyrazine (**2e**). Yellow solid, 49% yield. ^{1}H NMR (400 MHz, $CDCl_3$) δ 8.54 (s, 2H), 8.40 (s, 1H), 8.07 (dd, *J* = 10.3, 3.2 Hz, 3H), 7.97 (d, *J* = 8.8 Hz, 2H), 7.51 (t, *J* = 7.5 Hz, 2H), 7.41 (t, *J* = 7.4 Hz, 1H), 7.07 (d, *J* = 8.9 Hz, 2H), 3.33 (m, 4H), 2.72 (s, 6H), 2.62 (m, 4H), 2.38 (s, 3H). ^{13}C NMR (101 MHz, $CDCl_3$) δ 157.91, 151.58, 147.46, 145.45, 143.92, 138.91, 138.68, 132.95, 128.86, 128.76, 127.07, 126.98, 126.38, 120.09, 115.71, 113.08, 109.68, 54.96, 48.51, 46.16, 24.85. HRMS *m/z* (ESI) calcd for $C_{30}H_{30}N_6$ $[M+H]^+$ 475.2605 found: 475.2605.

.

6-(4-(4-methylpiperazin-1-yl)phenyl)-2-phenyl-8-(pyrimidin-5-yl)imidazo[1,2-*a*]pyrazine (**2f**). Yellow solid, 43% yield. ^{1}H NMR (400 MHz, $CDCl_3$) δ 10.33 (s, 2H), 9.36 (s, 1H), 8.39 (s, 1H), 8.08 (d, *J* = 1.3 Hz, 1H), 8.05 (d, *J* = 4.4 Hz, 2H), 7.95 (d, *J* = 8.8 Hz, 2H), 7.50 (t, *J* = 7.5 Hz, 2H), 7.41 (t, *J* = 7.3 Hz, 1H), 7.05 (d, *J* = 8.9 Hz, 2H), 3.33 (m, 4H), 2.62 (m, 4H), 2.38 (s, 3H). ^{13}C NMR (101 MHz, $CDCl_3$) δ 159.04, 157.51, 151.63, 147.79, 142.30, 139.03, 138.01, 132.60, 129.83, 128.93, 128.89, 126.81, 126.43, 126.32, 115.57, 112.89, 109.80, 54.95, 48.38, 46.23. HRMS *m/z* (ESI) calcd for $C_{27}H_{25}N_7$ $[M+H]^+$ 448.2244 found:448.2234.

8-(2-methoxypyrimidin-5-yl)-6-(4-(4-methylpiperazin-1-yl)phenyl)-2-phenylimidazo[1,2-*a*]pyrazine (**2g**). Yellow solid, 43% yield. ^{1}H NMR (400 MHz,

DMSO-d_6) δ 10.05 (s, 2H), 9.07 (s, 1H), 8.62 (s, 1H), 8.12 (d, J = 7.3 Hz, 2H), 8.03 (d, J = 8.8 Hz, 2H), 7.53 (t, J = 7.6 Hz, 2H), 7.42 (t, J = 7.3 Hz, 1H), 7.08 (d, J = 9.0 Hz, 2H), 4.07 (s, 3H), 3.25 (m, 4H), 2.47 (s, 4H), 2.24 (s, 3H). ^{13}C NMR (101 MHz, $CDCl_3$) δ 165.95, 160.46, 151.54, 147.39, 142.81, 138.84, 137.75, 132.79, 128.82, 128.73, 126.89, 126.82, 126.27, 124.07, 115.62, 112.21, 109.73, 55.28, 54.93, 48.42, 46.13. HRMS *m/z* (ESI) calcd for $C_{28}H_{27}N_7O$ [M+H]$^+$ 478.2350 found:478.2351.

6-(4-(4-methylpiperazin-1-yl)phenyl)-2,8-diphenylimidazo[1,2-a]pyrazine (**2h**). Yellow solid, 43% yield. ^{1}H NMR (400 MHz, $CDCl_3$) δ 9.02 (m, 2H), 8.31 (s, 1H), 8.07 (m, 2H), 7.97 (t, J = 4.4 Hz, 3H), 7.59 (m, 2H), 7.53 (m, 1H), 7.48 (t, J = 7.5 Hz, 2H), 7.38 (t, J = 7.4 Hz, 1H), 7.04 (d, J = 8.9 Hz, 2H), 3.31 (m, 4H), 2.61 (m, 4H), 2.38 (s, 3H). ^{13}C NMR (101 MHz, $CDCl_3$) δ 151.45, 147.66, 147.10, 138.81, 138.79, 136.40, 133.33, 130.26, 129.88, 128.78, 128.48, 128.27, 127.62, 126.98, 126.39, 115.73, 112.14, 109.64, 55.01, 48.61, 46.20. HRMS *m/z* (ESI) calcd for $C_{29}H_{27}N_5$ [M+H]$^+$ 446.2339 found:446.2339.

8-(1-methyl-1,2,3,6-tetrahydropyridin-4-yl)-6-(4-(4-methylpiperazin-1-yl)phenyl)-2-phenylimidazo[1,2-*a*]pyrazine (**2i**). Yellow solid, 50% yield. ^{1}H NMR (400 MHz, $CDCl_3$) δ 8.40 (s, 1H), 8.19 (s, 1H), 8.00 (d, J = 7.2 Hz, 2H), 7.88 (t, J = 4.2 Hz, 3H), 7.45 (t, J = 7.5 Hz, 2H), 7.35 (t, J = 7.3 Hz, 1H), 7.01 (d, J = 8.8 Hz, 2H), 3.40 (d, J = 2.6 Hz, 2H), 3.29 (m, 4H), 3.04 (s, 2H), 2.77 (t, J = 5.7 Hz, 2H), 2.60 (m, 4H), 2.48 (s, 3H), 2.37 (s, 3H). ^{13}C NMR (101 MHz, $CDCl_3$) δ 151.39, 147.75, 146.30, 138.34, 138.07, 134.71, 133.41, 133.25, 128.75, 128.32, 127.78, 126.83, 126.27, 115.76, 111.34, 109.25, 55.43, 55.00, 52.41, 48.67, 46.18, 45.85, 26.38. HRMS *m/z* (ESI) calcd for $C_{29}H_{32}N_6$ [M+H]$^+$ 465.2761 found:465.2763.

6-(4-(4-methylpiperazin-1-yl)phenyl)-2-phenyl-8-(1,2,3,6-tetrahydropyridin-4-yl)imidazo[1,2-*a*]pyrazine (**2j**). Yellow solid, 41% yield. ^{1}H NMR (400 MHz, DMSO-d_6) δ 8.90 (s, 1H), 8.48 (s, 1H), 8.38 (s, 1H), 8.05 (d, J = 7.3 Hz, 2H), 7.92 (d, J = 8.8 Hz, 2H), 7.49 (t, J = 7.6 Hz, 2H), 7.38 (t, J = 7.3 Hz, 1H), 7.04 (d, J = 8.9 Hz, 2H), 3.71 (s, 2H), 3.21 (m, 4H), 3.10 (s, 2H), 2.81 (s, 2H), 2.46 (m, 4H), 2.22 (s, 3H). ^{13}C NMR (101 MHz, DMSO-d_6) δ 151.47, 146.72, 145.71, 137.88, 137.22, 135.52, 133.60, 133.58, 129.30, 128.76, 126.92, 126.84, 126.39, 115.50, 113.16, 113.13, 54.98, 48.09, 46.25, 45.00, 42.62, 25.09. HRMS *m/z* (ESI) calcd for $C_{28}H_{30}N_6$ [M+H]$^+$451.2605 found:451.2602.

8-(3,6-dihydro-2*H*-pyran-4-yl)-6-(1,5-dimethyl-1*H*-pyrazol-4-yl)-2-phenylimidazo[1,2-*a*]pyrazine (**3a**). Yellow solid, 40% yield. ^{1}H NMR (400 MHz, $CDCl_3$) δ 8.48 (s, 1H), 8.07 (s, 1H), 8.01 (m, 2H), 7.92 (s, 1H), 7.74 (s, 1H), 7.47 (t, J

= 7.5 Hz, 2H), 7.37 (t, J = 7.4 Hz, 1H), 4.58 (m, 2H), 4.03 (t, J = 5.5 Hz, 2H), 3.87 (s, 3H), 2.90 (d, J = 1.7 Hz, 2H), 2.65 (s, 3H). ^{13}C NMR (101 MHz, $CDCl_3$) δ 147.35, 146.48, 137.81, 137.34, 136.33, 135.94, 134.30, 133.21, 132.53, 128.80, 128.47, 126.26, 116.69, 112.26, 109.01, 65.97, 64.82, 36.45, 25.60, 11.06. HRMS *m/z* (ESI) calcd for $C_{22}H_{21}N_5O$ $[M+H]^+$372.1819 found:372.1808.

8-(3,6-dihydro-2*H*-pyran-4-yl)-6-(1-methyl-1*H*-pyrazol-5-yl)-2-phenylimidazo[1,2-*a*]pyrazine (**3b**). Yellow solid, 56% yield. ^{1}H NMR (400 MHz, DMSO-d_6) δ 8.90 (s, 1H), 8.59 (s, 1H), 8.50 (s, 1H), 8.08 (d, J = 7.2 Hz, 2H), 7.51 (q, J = 6.8 Hz, 3H), 7.40 (t, J = 7.3 Hz, 1H), 6.70 (d, J = 1.9 Hz, 1H), 4.48 (d, J = 2.5 Hz, 2H), 4.13 (s, 3H), 3.92 (t, J = 5.4 Hz, 2H), 2.77 (s, 2H). ^{13}C NMR (101 MHz, DMSO-d_6) δ 146.32, 138.95, 138.24, 137.52, 137.08, 133.22, 132.19, 130.32, 129.37, 129.05, 126.53, 117.60, 111.51, 106.08, 73.98, 65.61, 64.36, 25.41. HRMS *m/z* (ESI) calcd for $C_{21}H_{19}N_5O$ $[M+H]^+$358.1662 found:358.1659.

8-(3,6-dihydro-2*H*-pyran-4-yl)-2-phenyl-6-(1H-pyrazol-4-yl)imidazo[1,2-*a*]pyrazine (**3c**). Yellow solid, 37% yield. ^{1}H NMR (400 MHz, DMSO-d_6) δ 13.07 (s, 1H), 8.78 (s, 1H), 8.49 (s, 1H), 8.39 (s, 1H), 8.26 (s, 1H), 8.06 (d, J = 7.2 Hz, 2H), 7.49 (t, J = 7.6 Hz, 2H), 7.38 (t, J = 7.3 Hz, 1H), 4.46 (d, J = 2.3 Hz, 2H), 3.92 (t, J = 5.3 Hz, 2H), 2.82 (s, 2H). ^{13}C NMR (101 MHz, DMSO-d_6) δ 146.63, 145.71, 137.80, 136.14, 136.08, 133.53, 133.07, 132.29, 129.27, 128.77, 126.41, 119.85, 113.02, 110.92, 65.58, 64.43, 25.38. HRMS *m/z* (ESI) calcd for $C_{20}H_{17}N_5O$ $[M+H]^+$344.1506 found:344.1501.

8-(3,6-dihydro-2*H*-pyran-4-yl)-6-(1-methyl-1*H*-pyrazol-3-yl)-2-phenylimidazo[1,2-*a*]pyrazine (**3d**). White solid, 45% yield. ^{1}H NMR (400 MHz, $CDCl_3$) δ 8.58 (s, 1H), 8.42 (m, 1H), 8.02 (m, 2H), 7.91 (s, 1H), 7.45 (m, 3H), 7.37 (m, 1H), 6.89 (d, J = 2.2 Hz, 1H), 4.58 (dd, J = 5.3, 2.6 Hz, 2H), 4.04 (t, J = 5.5 Hz, 2H), 3.98 (s, 3H), 2.97 (d, J = 1.6 Hz, 2H). ^{13}C NMR (101 MHz, $CDCl_3$) δ 149.95, 147.34, 146.68, 138.61, 135.58, 133.40, 133.19, 132.50, 131.45, 128.77, 128.47, 126.31, 112.41, 109.33, 104.79, 65.96, 64.88, 39.17, 25.36. HRMS *m/z* (ESI) calcd for $C_{21}H_{19}N_5O$ $[M+H]^+$358.1662 found:358.1660.

8-(3,6-dihydro-2*H*-pyran-4-yl)-6-(1-methyl-1*H*-imidazol-5-yl)-2-phenylimidazo[1,2-*a*]pyrazine (**3e**). Yellow solid, 49% yield. ^{1}H NMR (400 MHz, DMSO-d_6) δ 8.79 (s, 1H), 8.56 (s, 1H), 8.48 (s, 1H), 8.07 (d, J = 7.2 Hz, 2H), 7.77 (s, 1H), 7.50 (t, J = 7.6 Hz, 2H), 7.40 (t, J = 7.4 Hz, 1H), 7.34 (s, 1H), 4.47 (d, J = 2.5 Hz, 2H), 3.92 (d, J = 5.5 Hz, 2H), 3.90 (s, 3H), 2.75 (s, 2H). ^{13}C NMR (101 MHz, $CDCl_3$) δ 147.40, 147.09, 140.53, 140.51, 137.83, 136.82, 132.90, 132.21, 131.04, 128.80, 128.52, 126.36, 114.37, 109.37, 65.98, 64.72, 33.99, 25.60. HRMS *m/z* (ESI) calcd for $C_{21}H_{19}N_5O$

$[M+H]^+$358.1662 found:358.1665.

8-(3,6-dihydro-2*H*-pyran-4-yl)-6-(1*H*-indol-4-yl)-2-phenylimidazo[1,2-*a*]pyrazine (**3f**). White solid, 44% yield. ^{1}H NMR (400 MHz, $CDCl_3$) δ 8.49 (m, 1H), 8.39 (d, *J* = 5.4 Hz, 2H), 8.04 (m, 2H), 7.97 (s, 1H), 7.58 (m, 1H), 7.48 (t, *J* = 7.7 Hz, 3H), 7.38 (ddd, *J* = 6.5, 3.9, 1.1 Hz, 1H), 7.34 (t, *J* = 2.8 Hz, 1H), 7.07 (m, 1H), 4.60 (dd, *J* = 5.3, 2.6 Hz, 2H), 4.05 (t, *J* = 5.5 Hz, 2H), 3.02 (d, *J* = 1.6 Hz, 2H). ^{13}C NMR (101 MHz, DMSO-d_6) δ 146.31, 145.73, 138.76, 137.85, 137.26, 136.16, 133.58, 132.55, 129.35, 128.98, 128.81, 126.57, 126.39, 125.54, 121.52, 118.91, 116.38, 112.36, 111.45, 101.75, 65.60, 64.41, 25.69. HRMS *m/z* (ESI) calcd for $C_{25}H_{20}N_4O$ $[M+H]^+$393.1701 found:393.1708.

^{1}H NMR Spectra, ^{13}C NMR Spectra of all compounds.

^{1}H NMR Spectra of Cpd-015

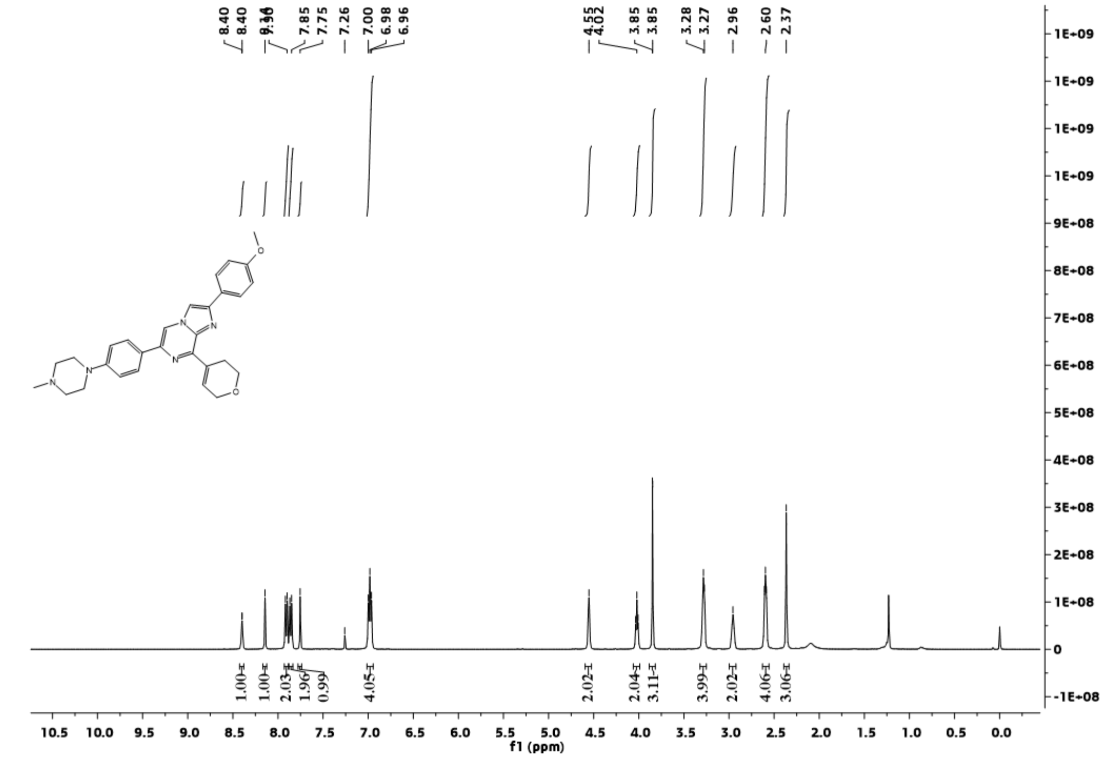

^{13}C NMR Spectra of Cpd-015

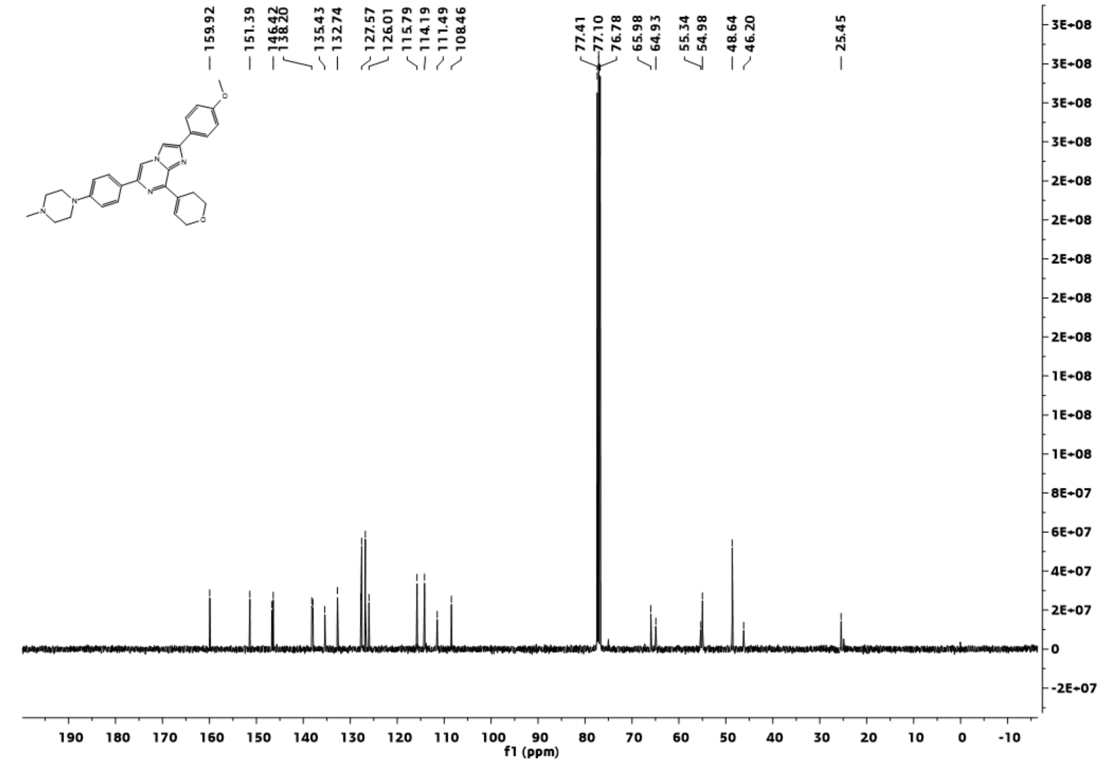

^{1}H NMR Spectra of **1a**

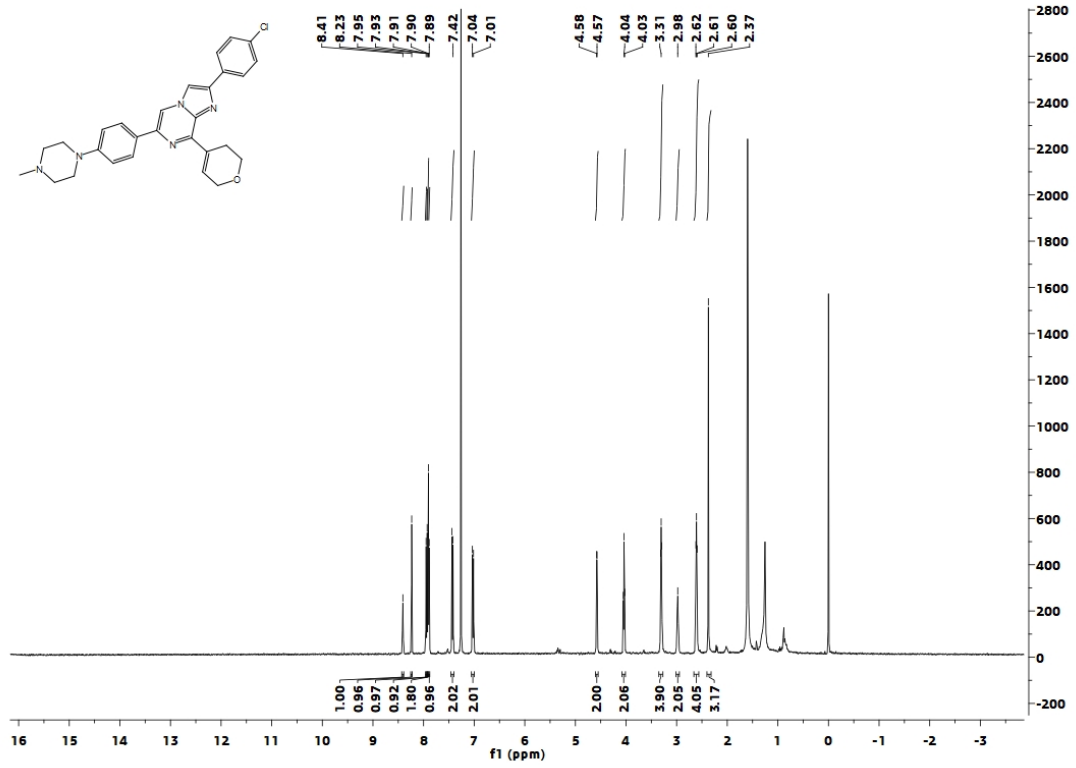

^{13}C NMR Spectra of **1a**

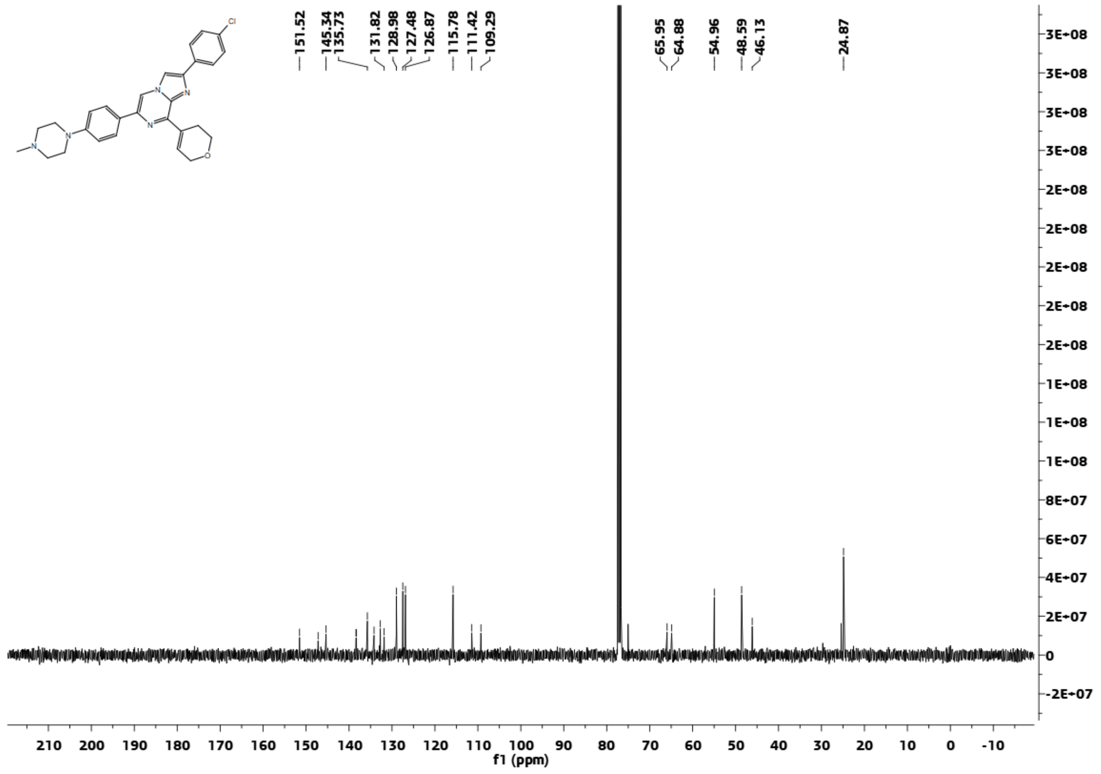

^{1}H NMR Spectra of **1b**

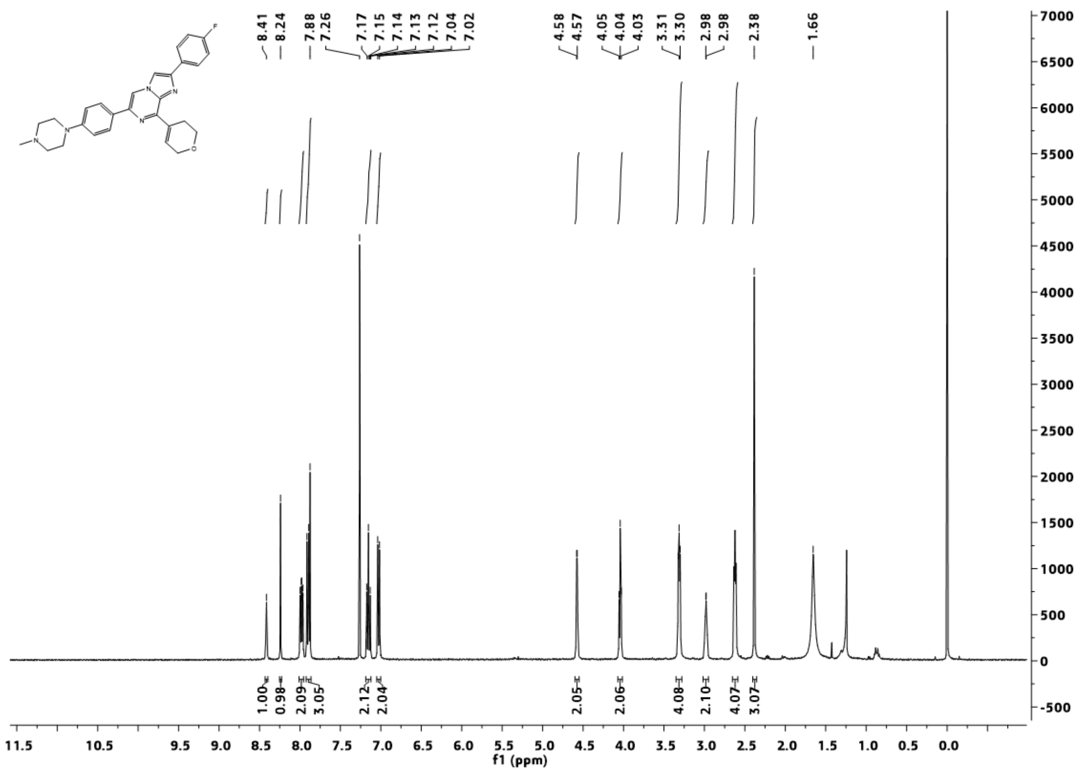

^{13}C NMR Spectra of **1b**

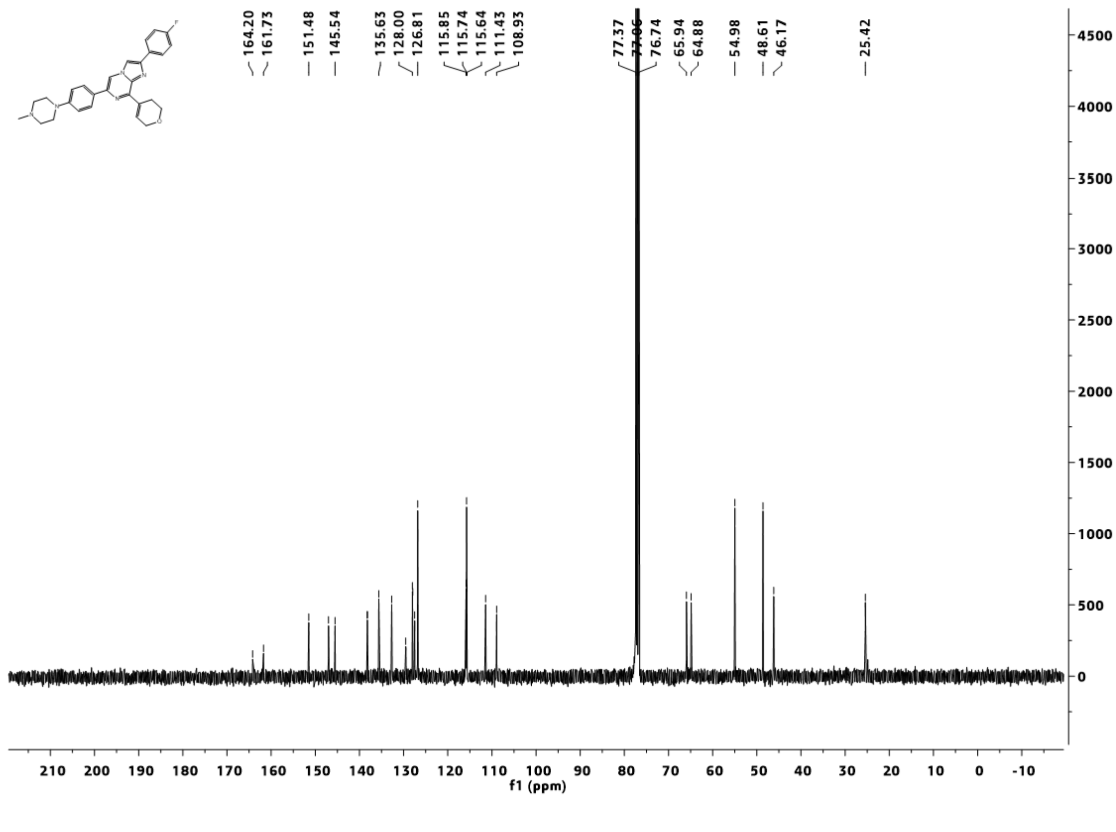

^{1}H NMR Spectra of **1c**

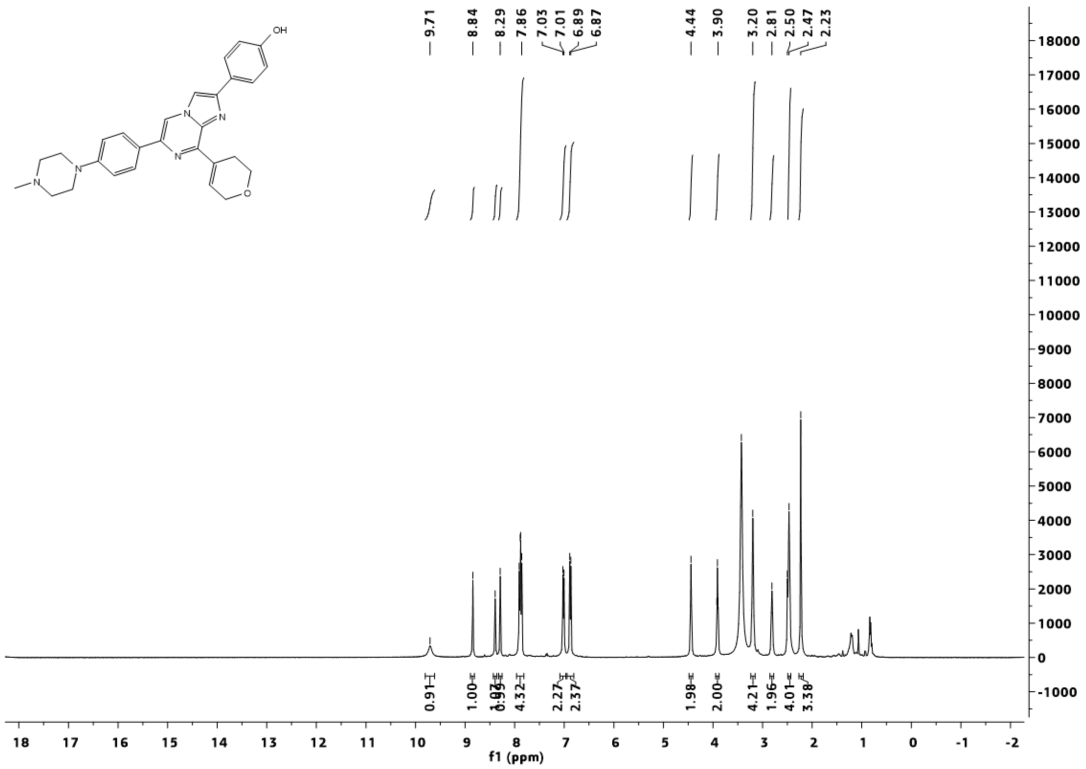

^{13}C NMR Spectra of **1c**

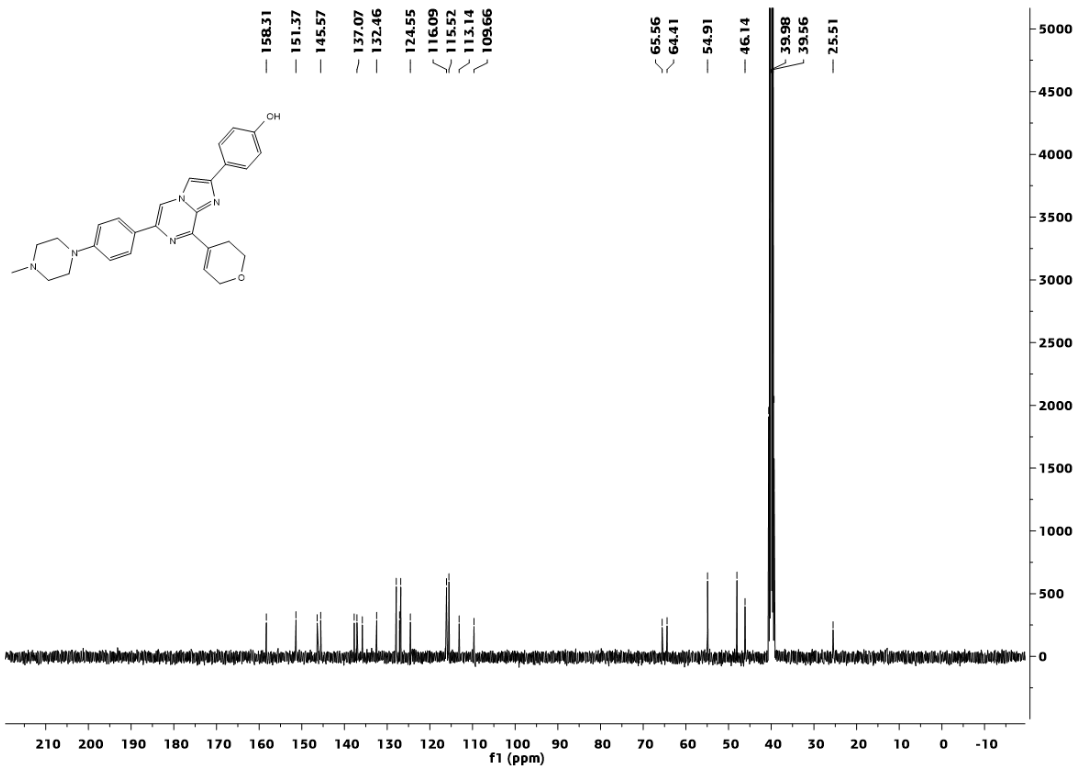

^{1}H NMR Spectra of **1d**

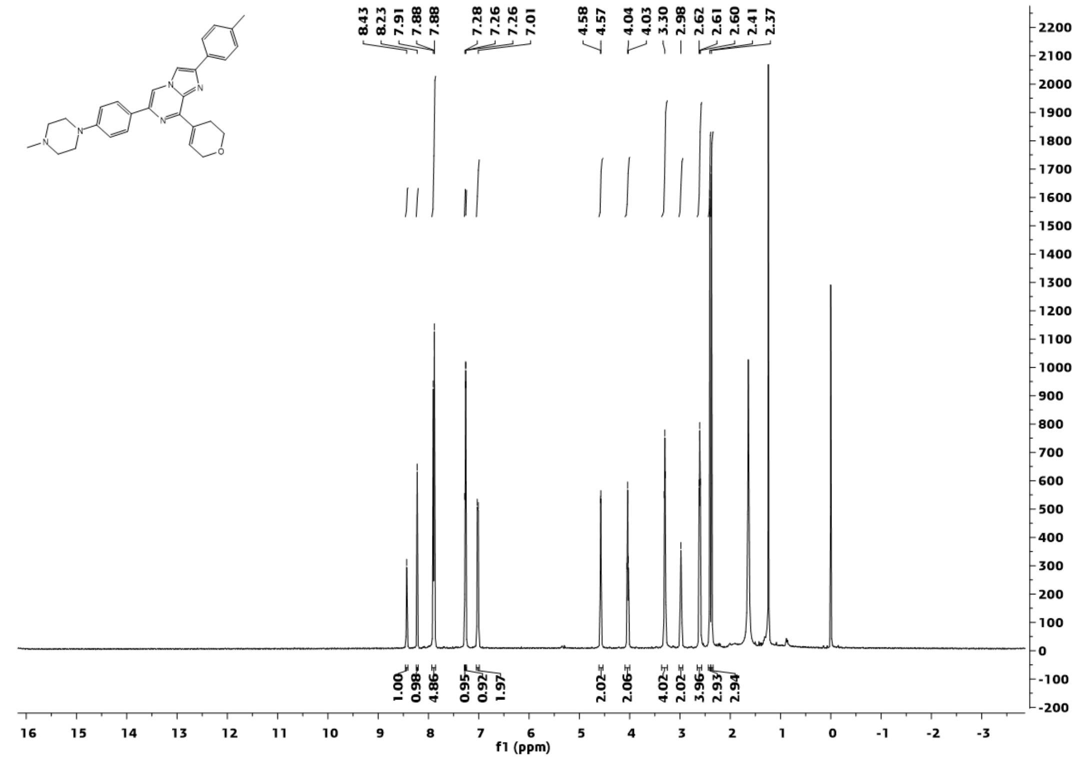

^{13}C NMR Spectra of **1d**

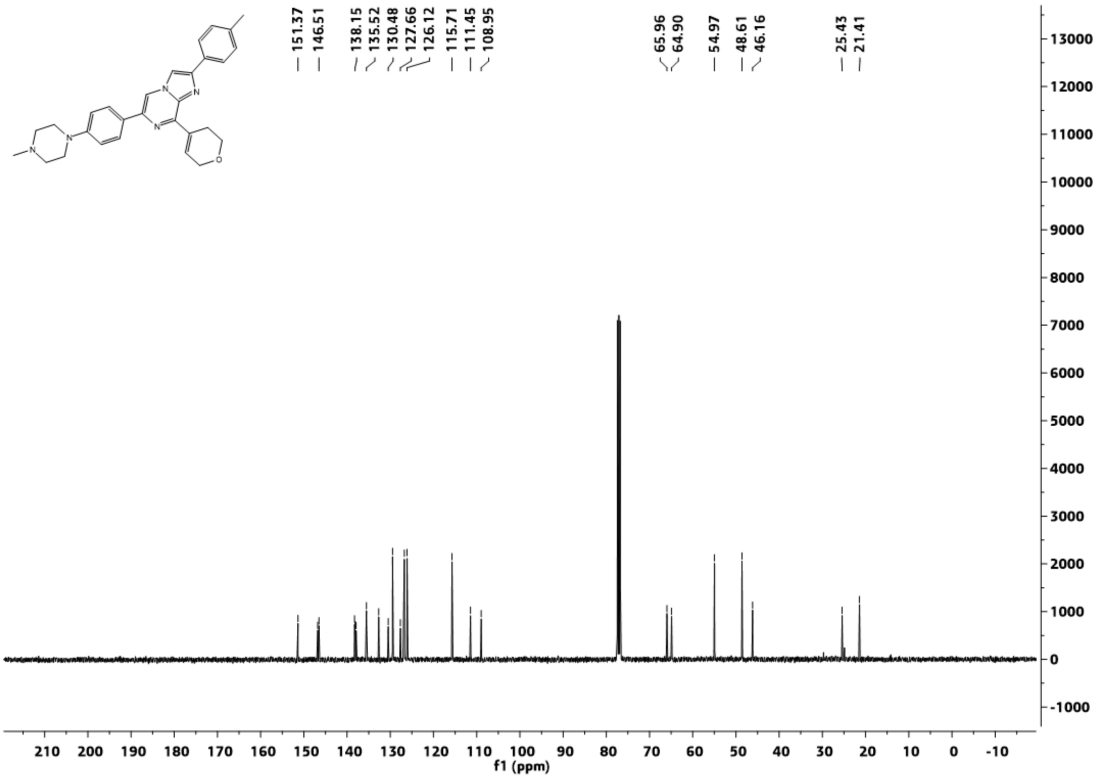

^{1}H NMR Spectra of **1e**

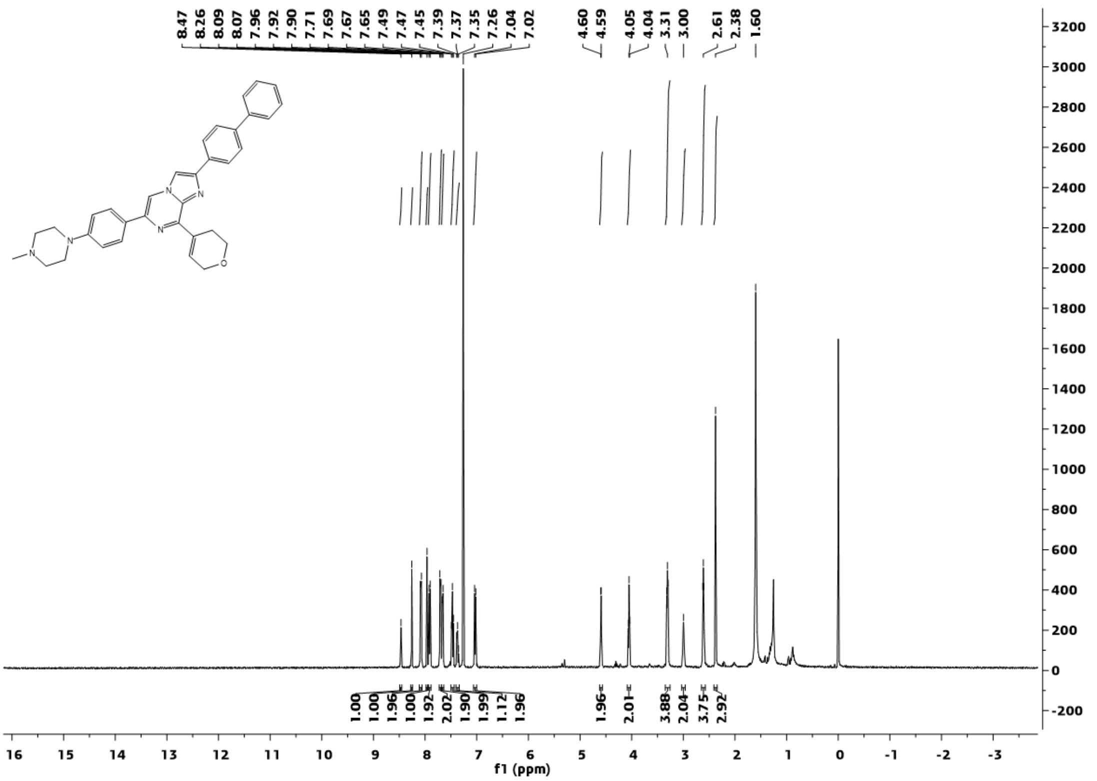

^{13}C NMR Spectra of **1e**

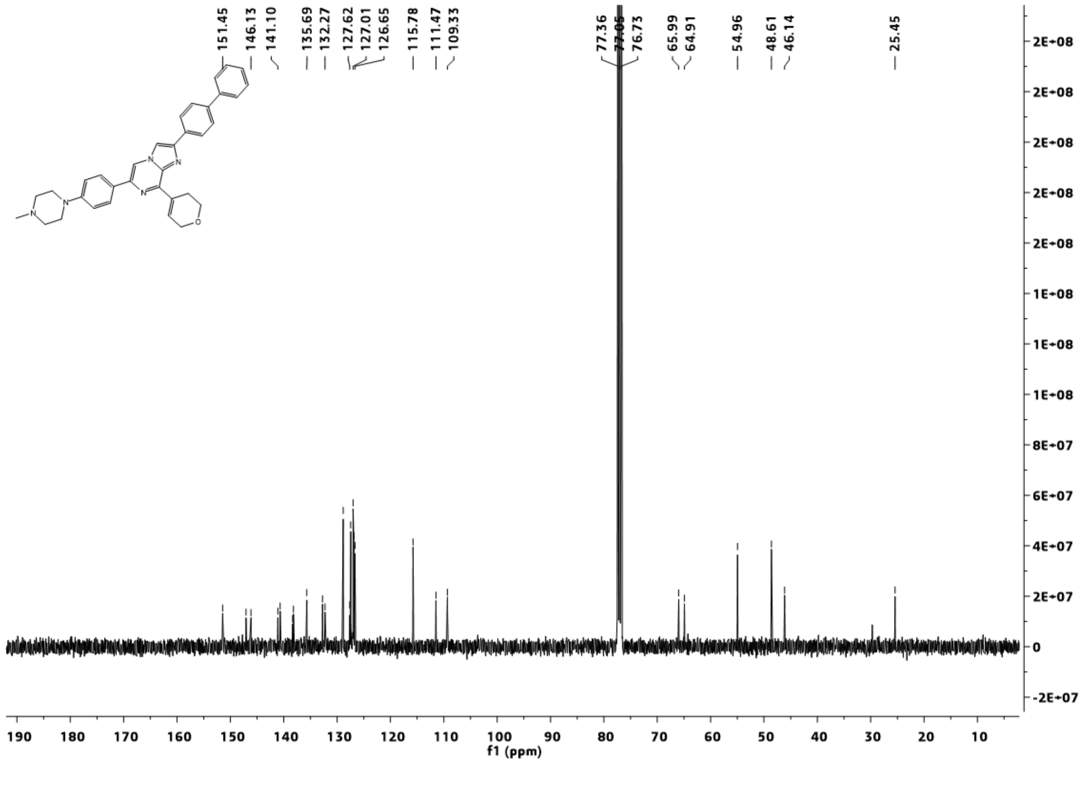

^{1}H NMR Spectra of **1f**

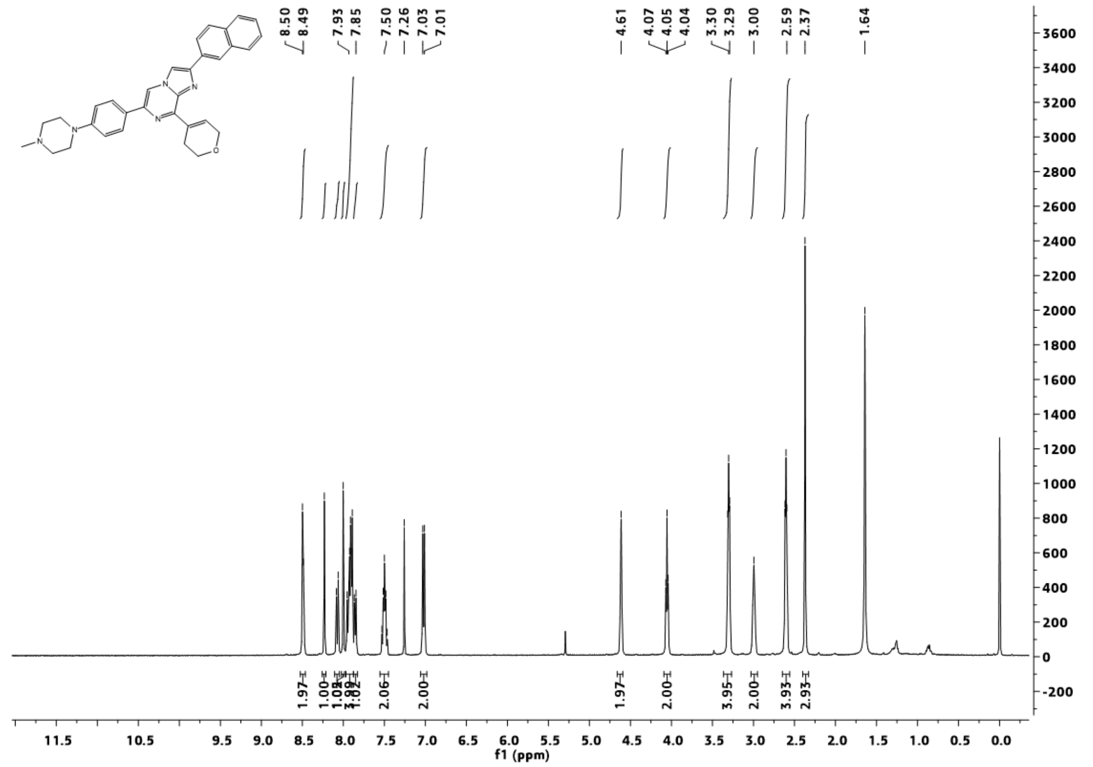

^{13}C NMR Spectra of **1f**

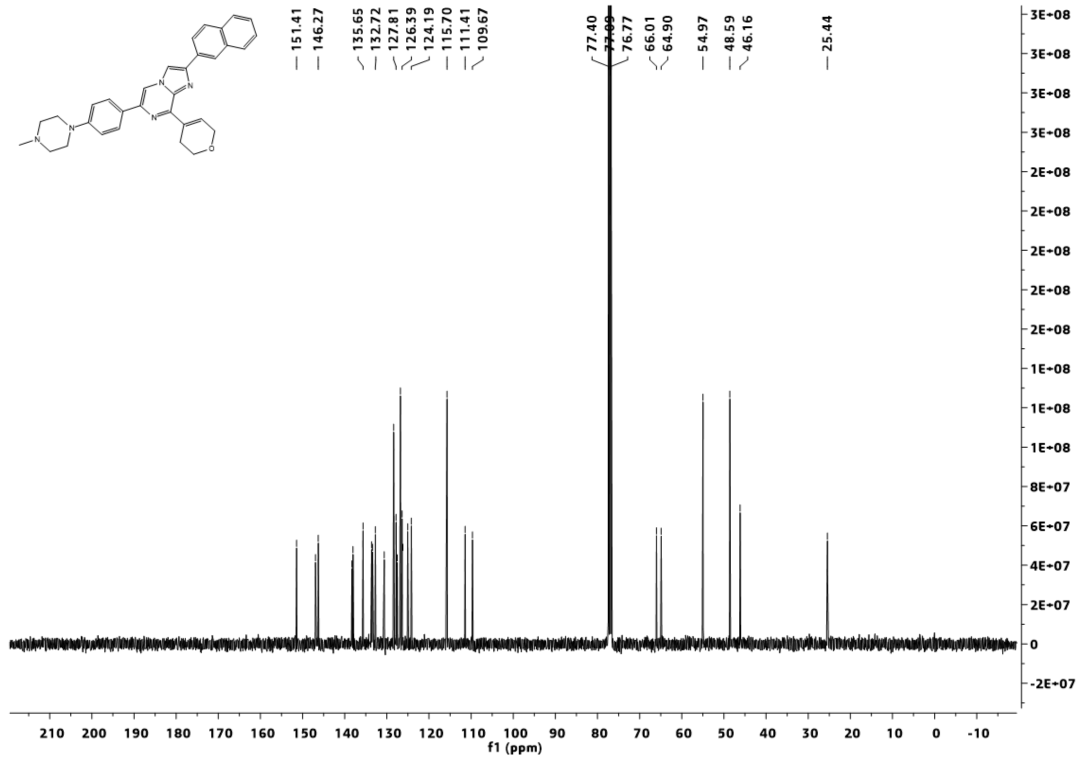

^{1}H NMR Spectra of **1g**

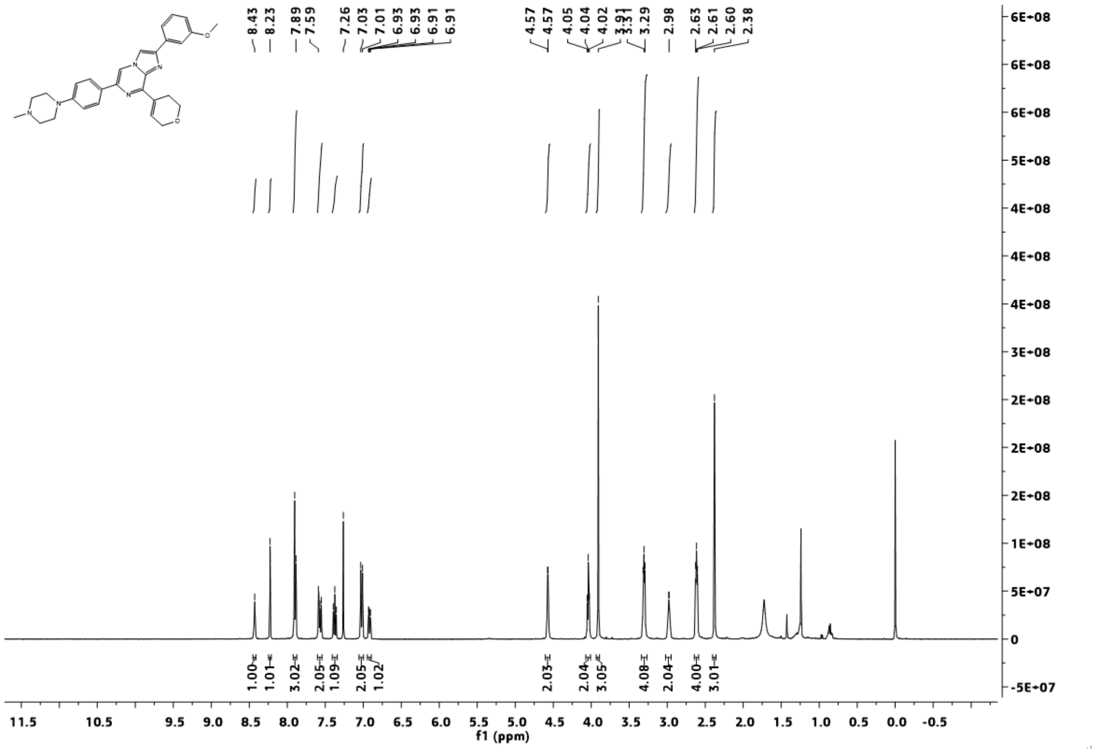

^{13}C NMR Spectra of **1g**

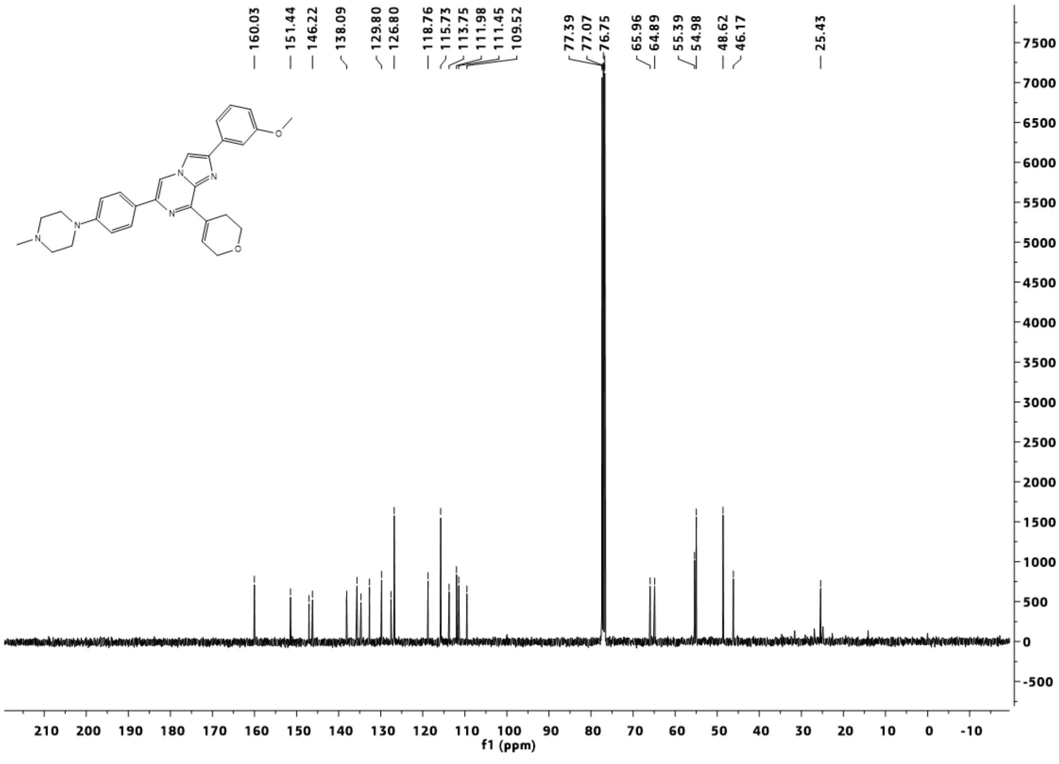

^{1}H NMR Spectra of **1h**

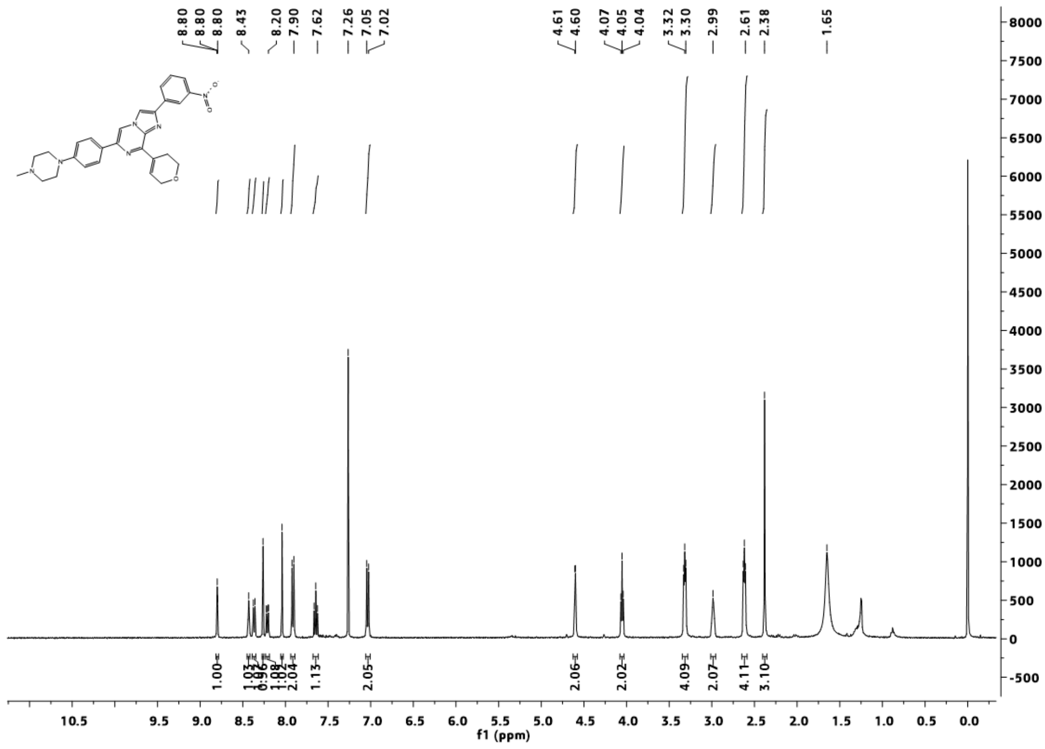

^{13}C NMR Spectra of **1h**

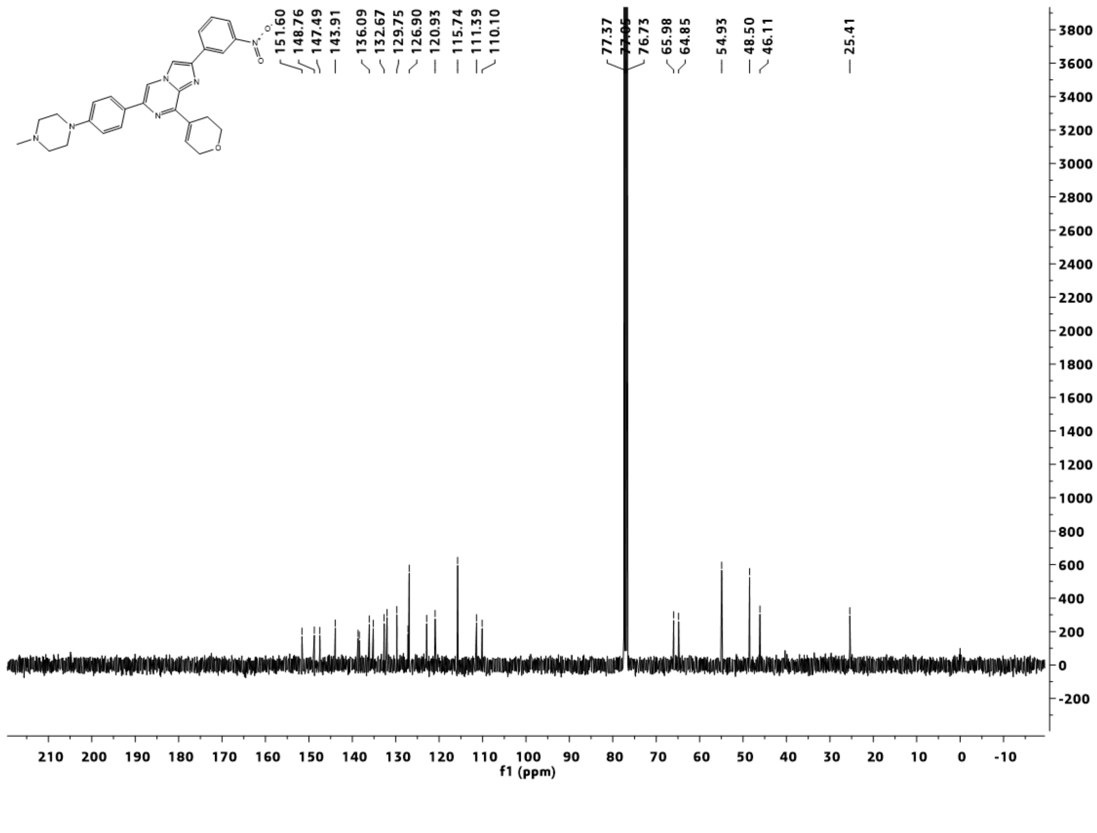

^{1}H NMR Spectra of **1i**

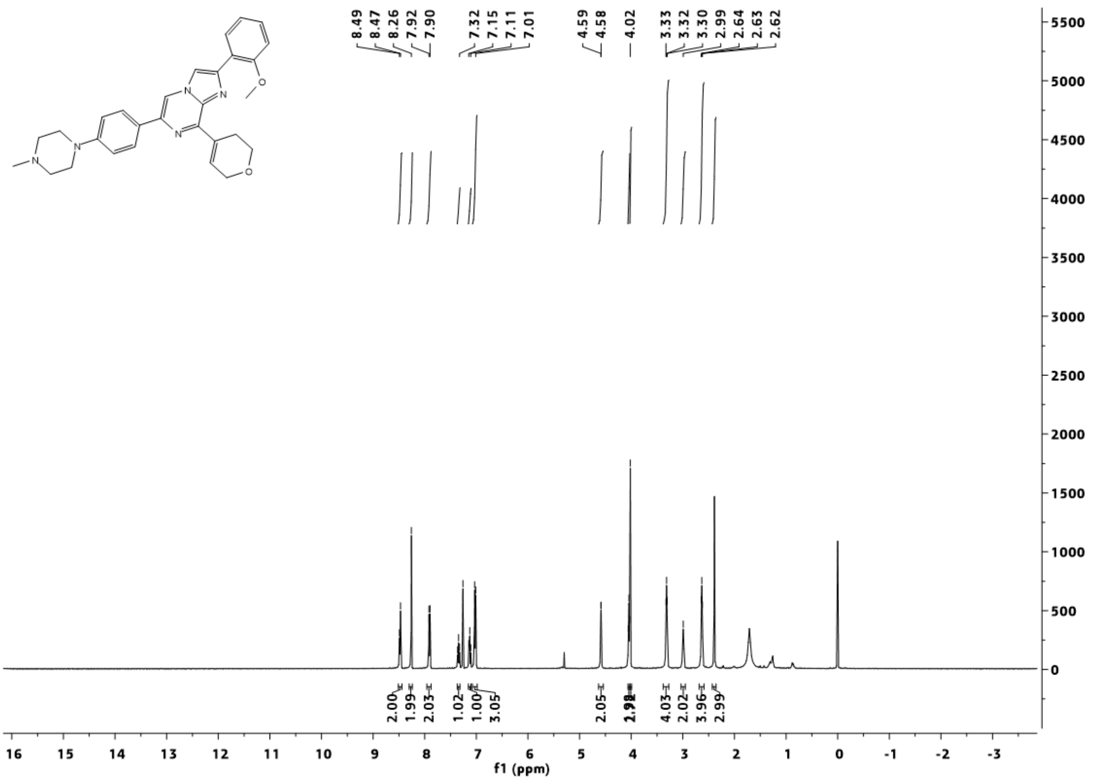

^{13}C NMR Spectra of **1i**

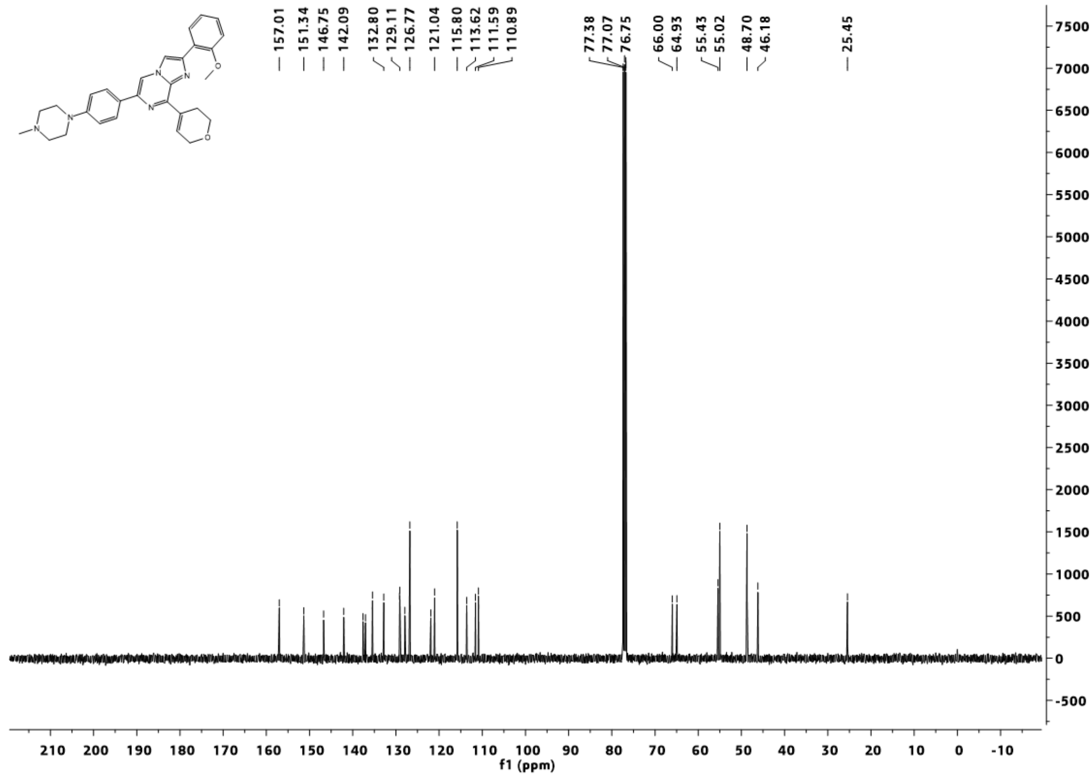

^{1}H NMR Spectra of **1j**

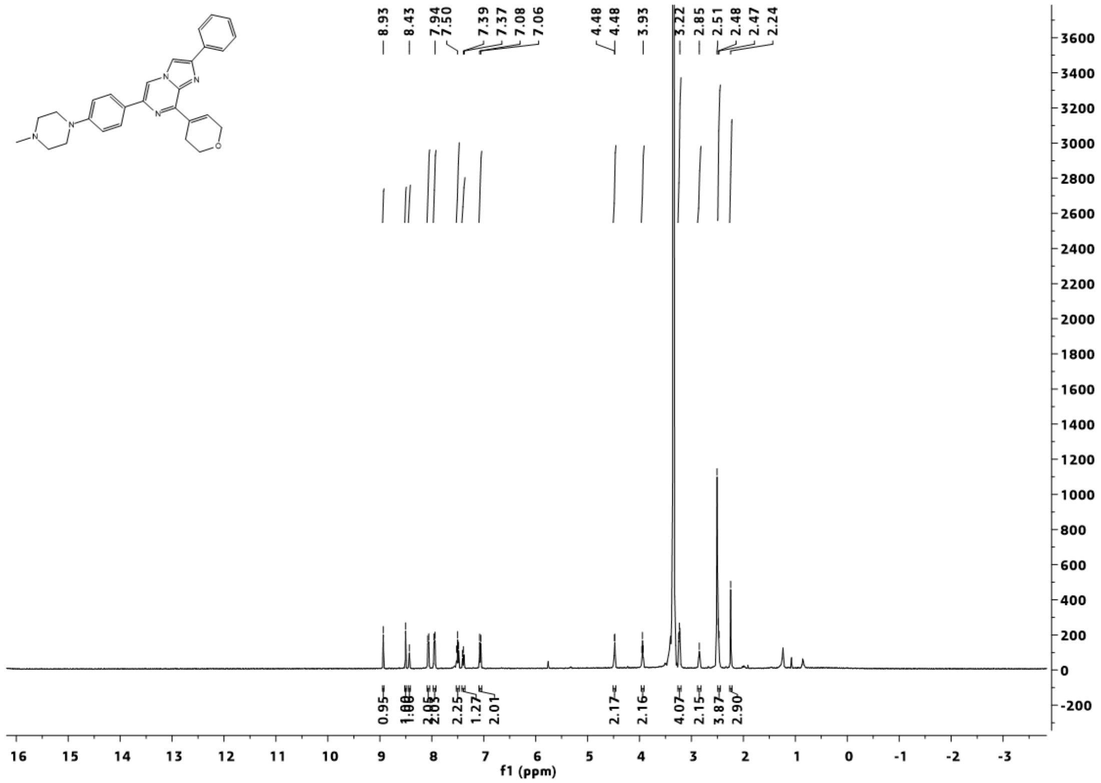

^{13}C NMR Spectra of **1j**

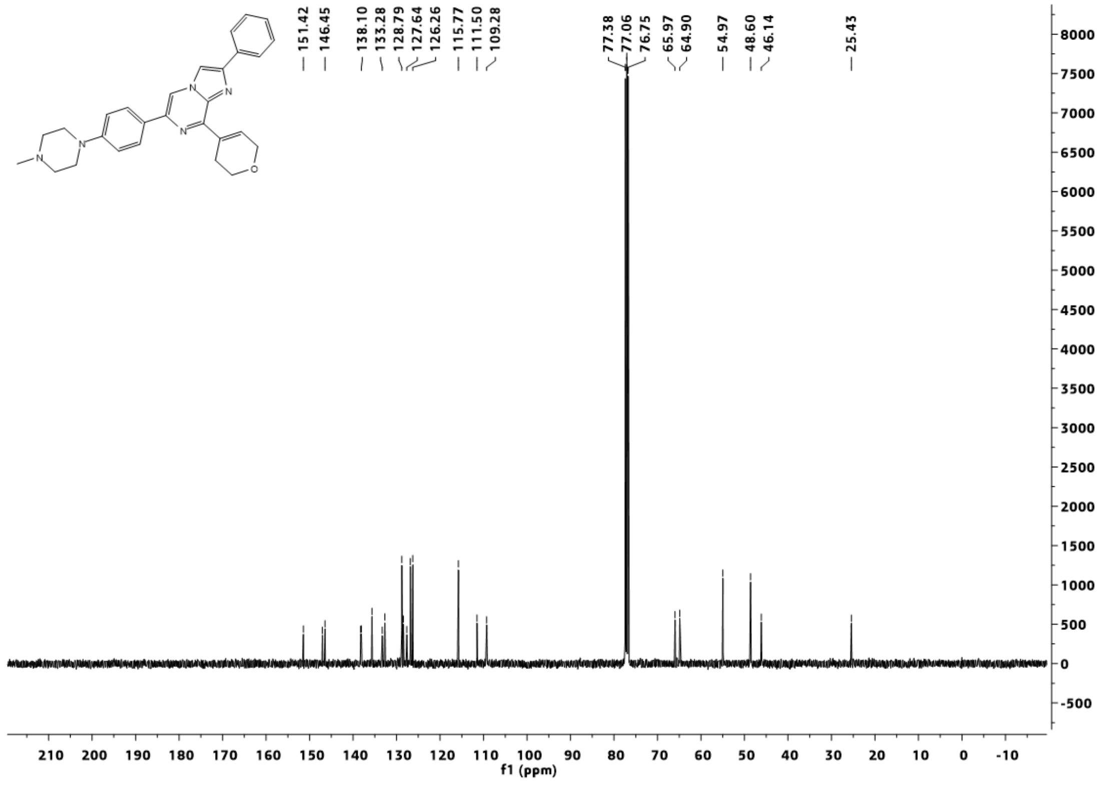

^{1}H NMR Spectra of **2a**

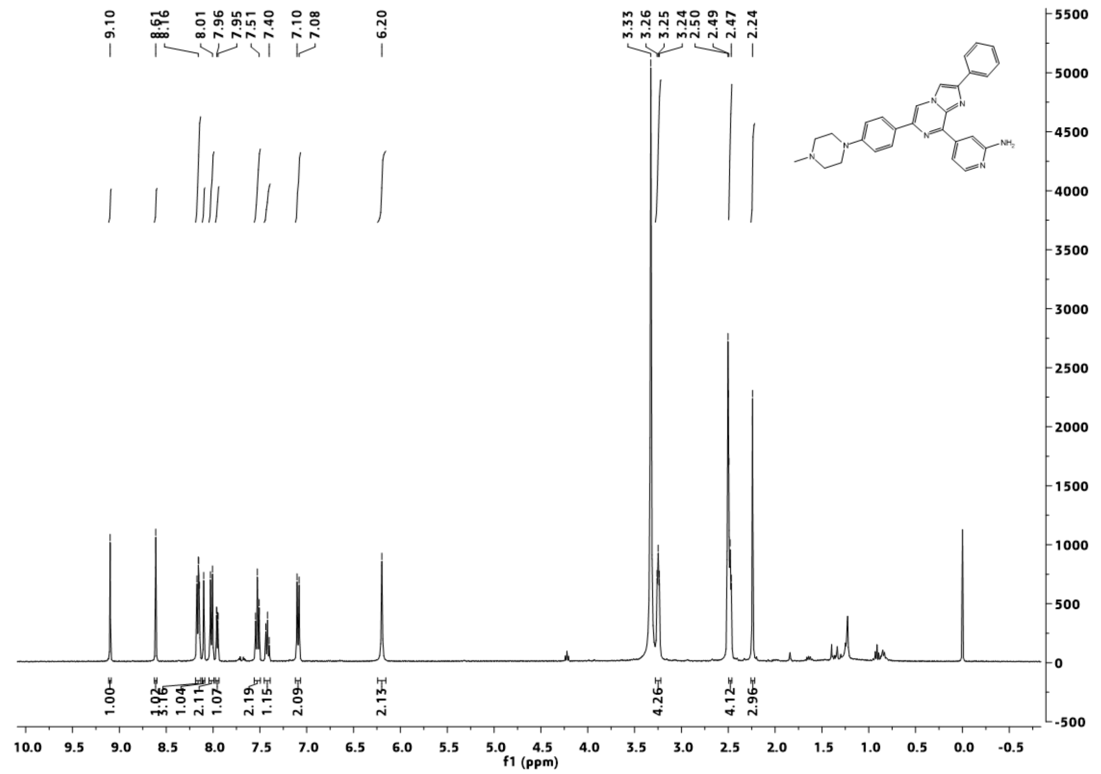

^{13}C NMR Spectra of **2a**

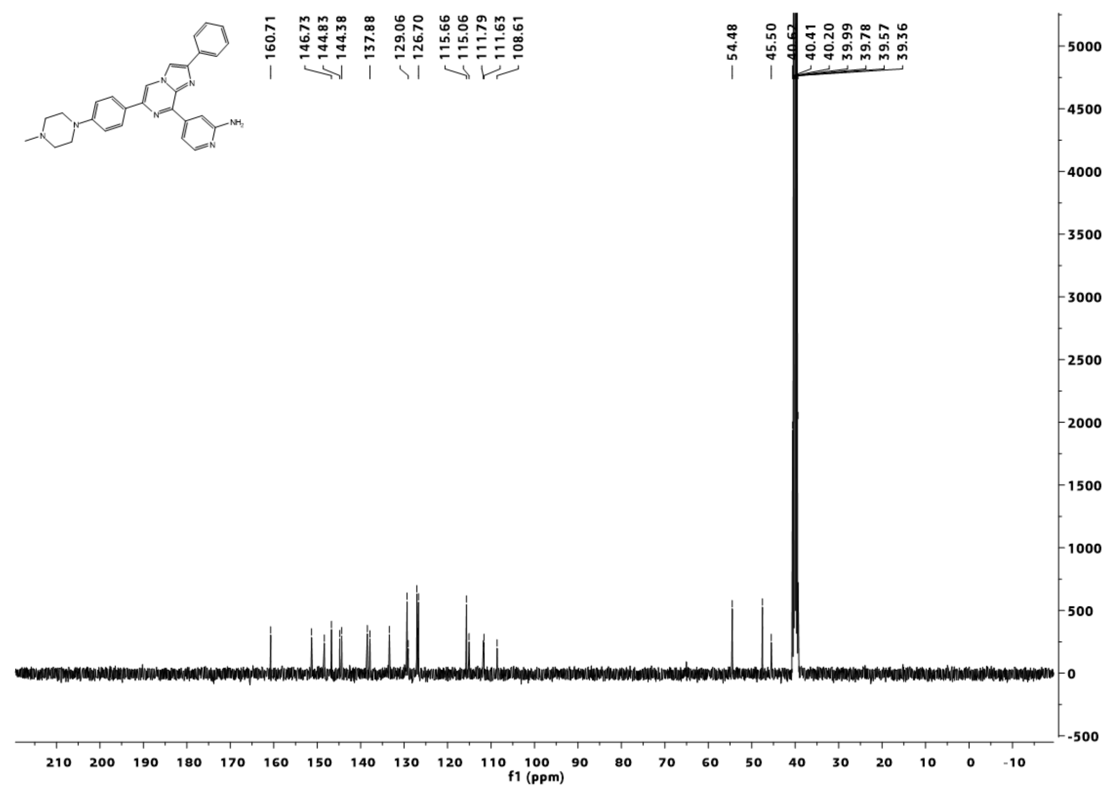

^{1}H NMR Spectra of **2b**

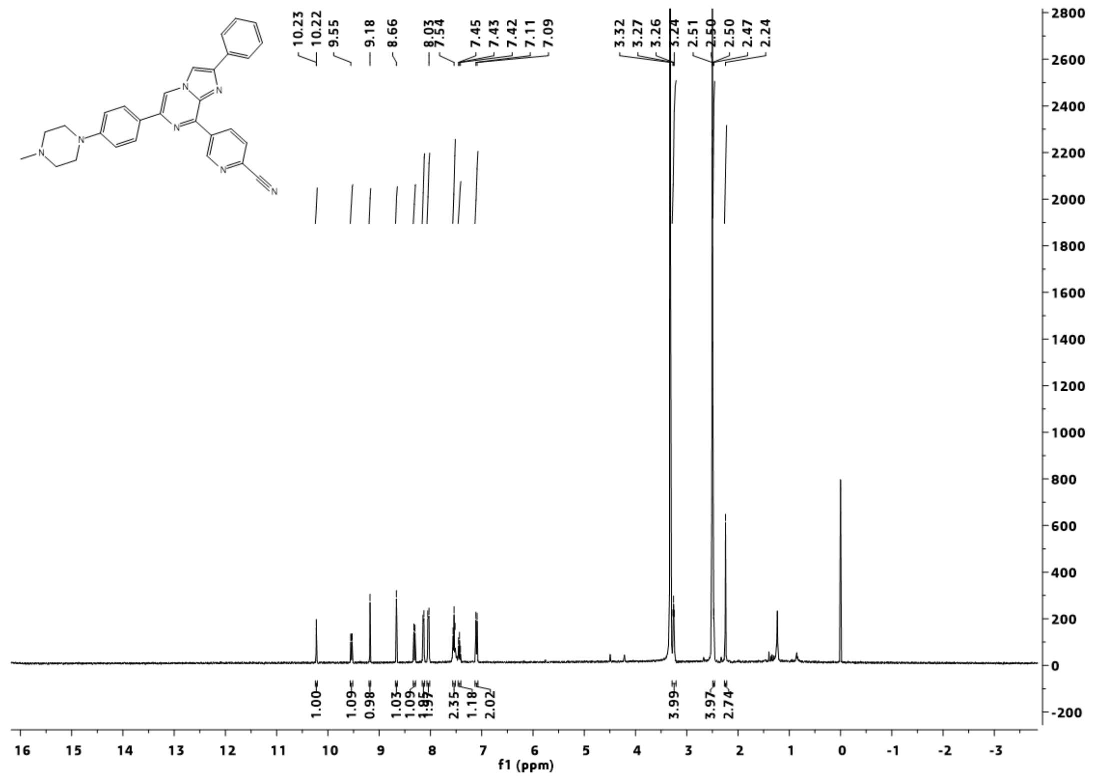

^{13}C NMR Spectra of **2b**

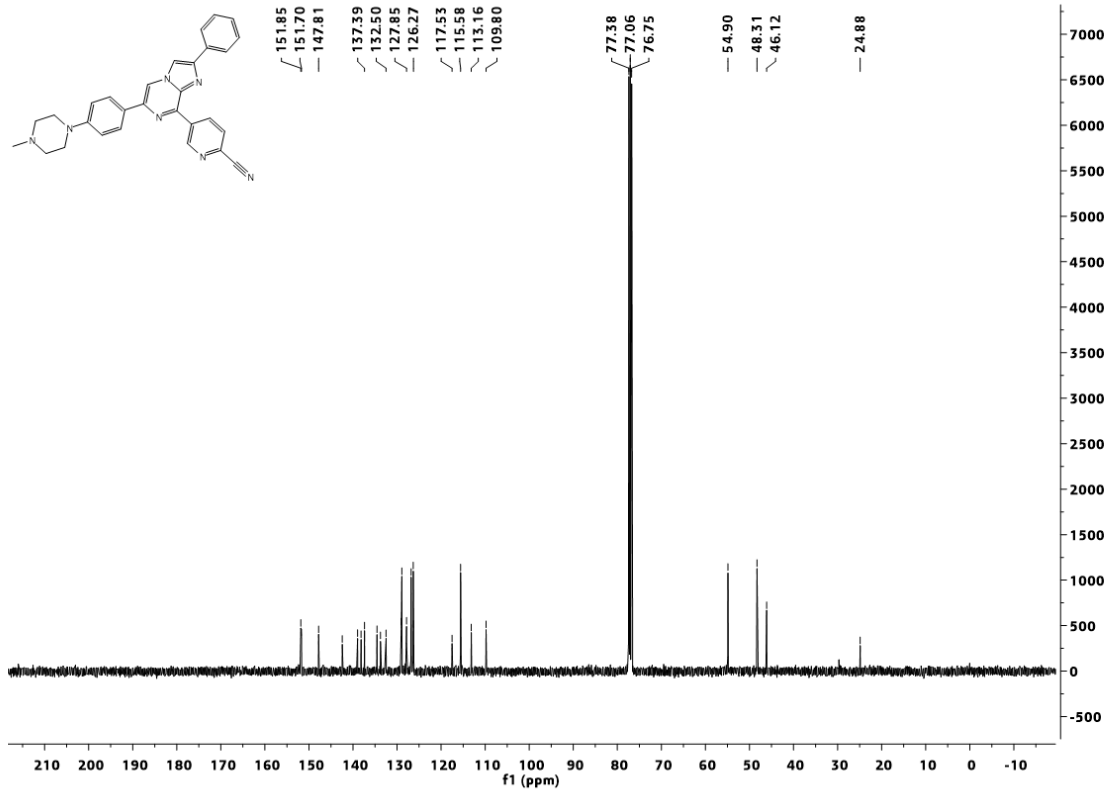

^{1}H NMR Spectra of **2c**

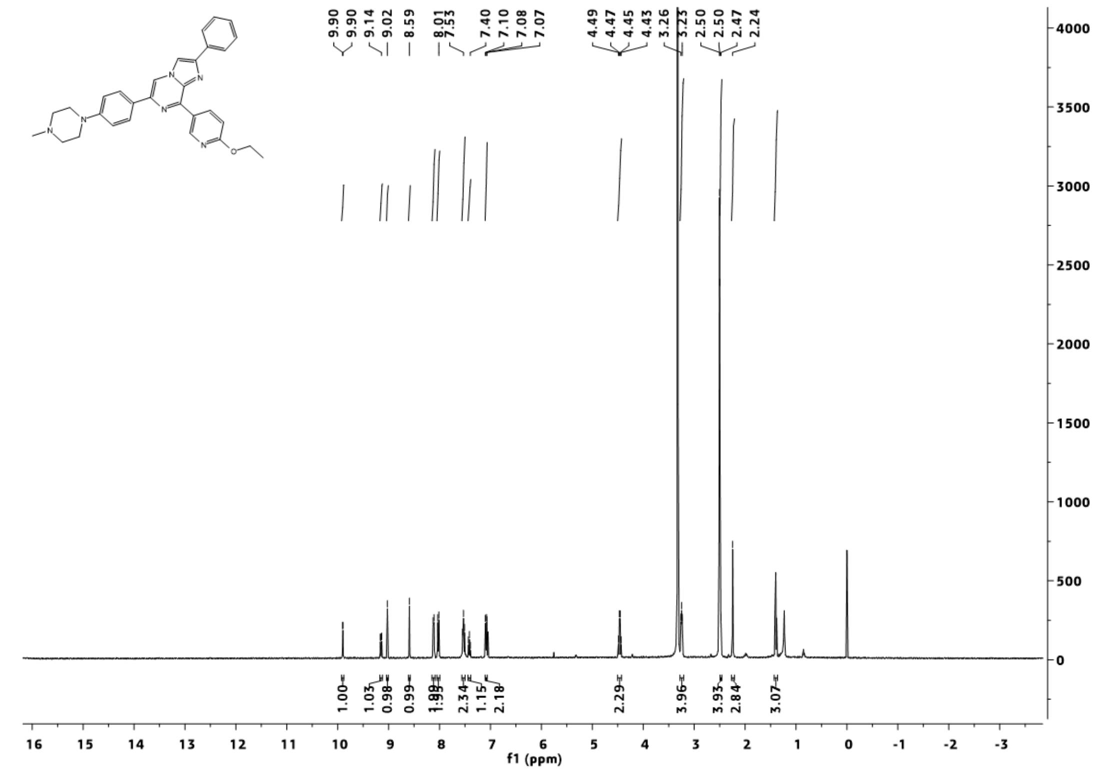

^{13}C NMR Spectra of **2c**

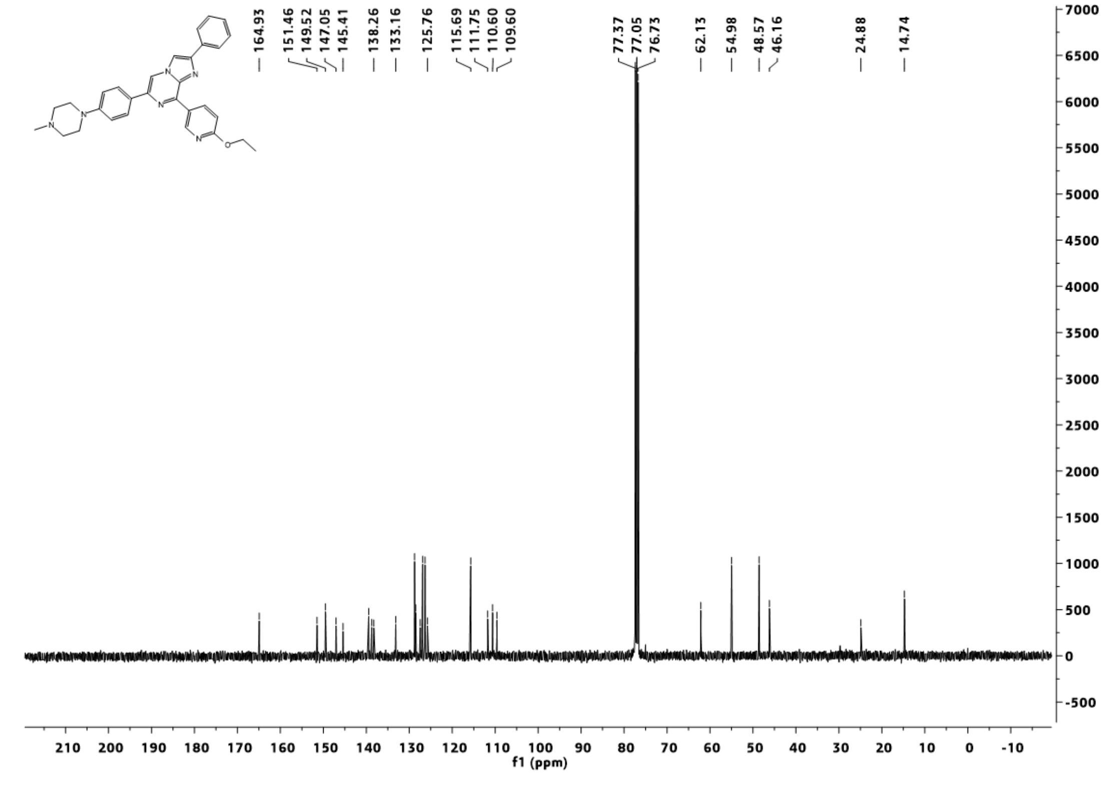

[1] H NMR Spectra of **2d**

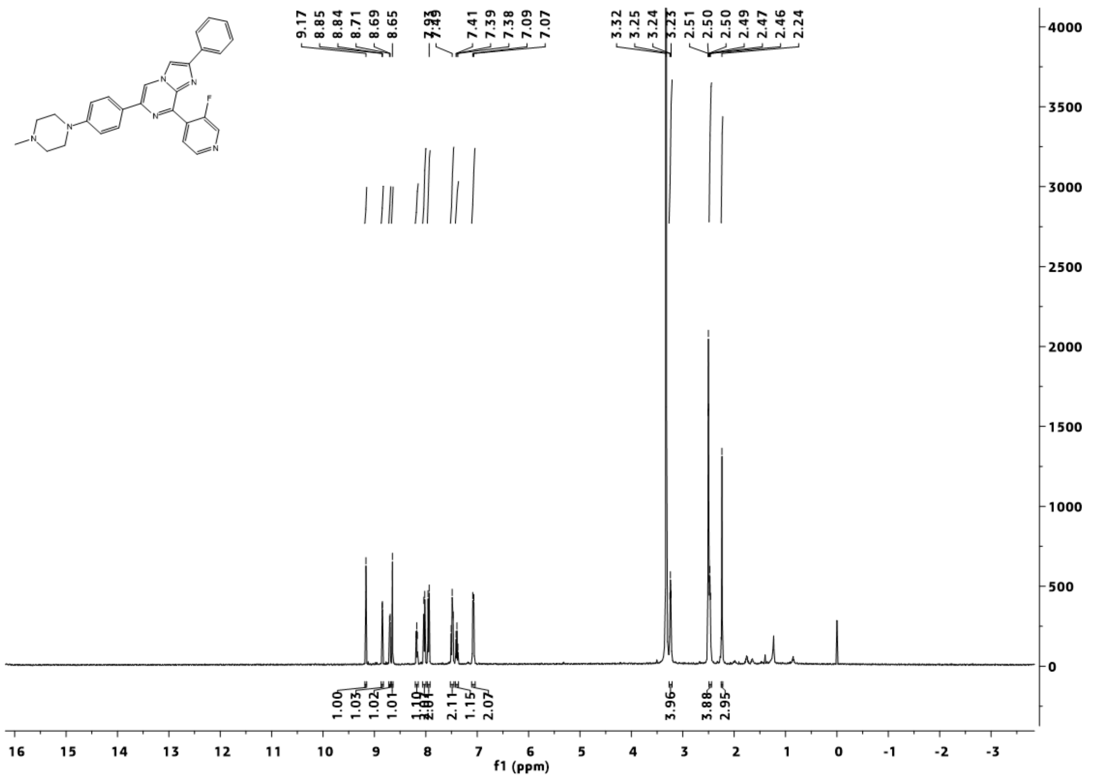

[13] C NMR Spectra of **2d**

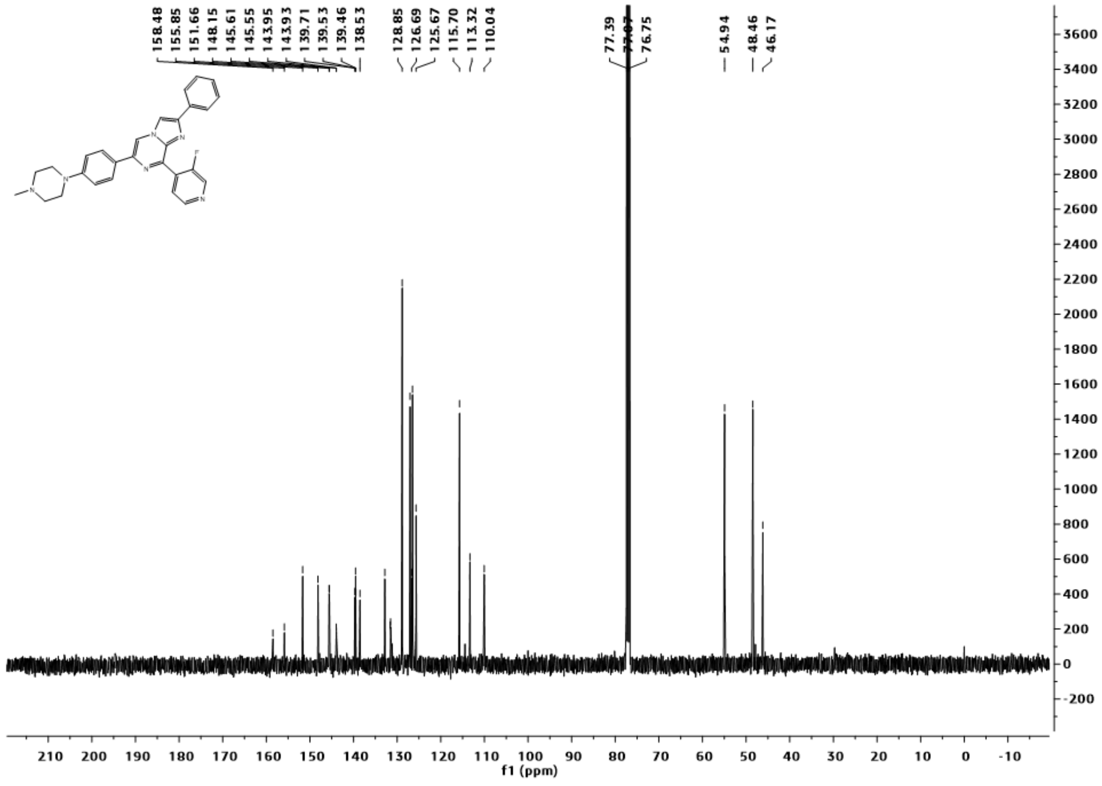

^{1}H NMR Spectra of **2e**

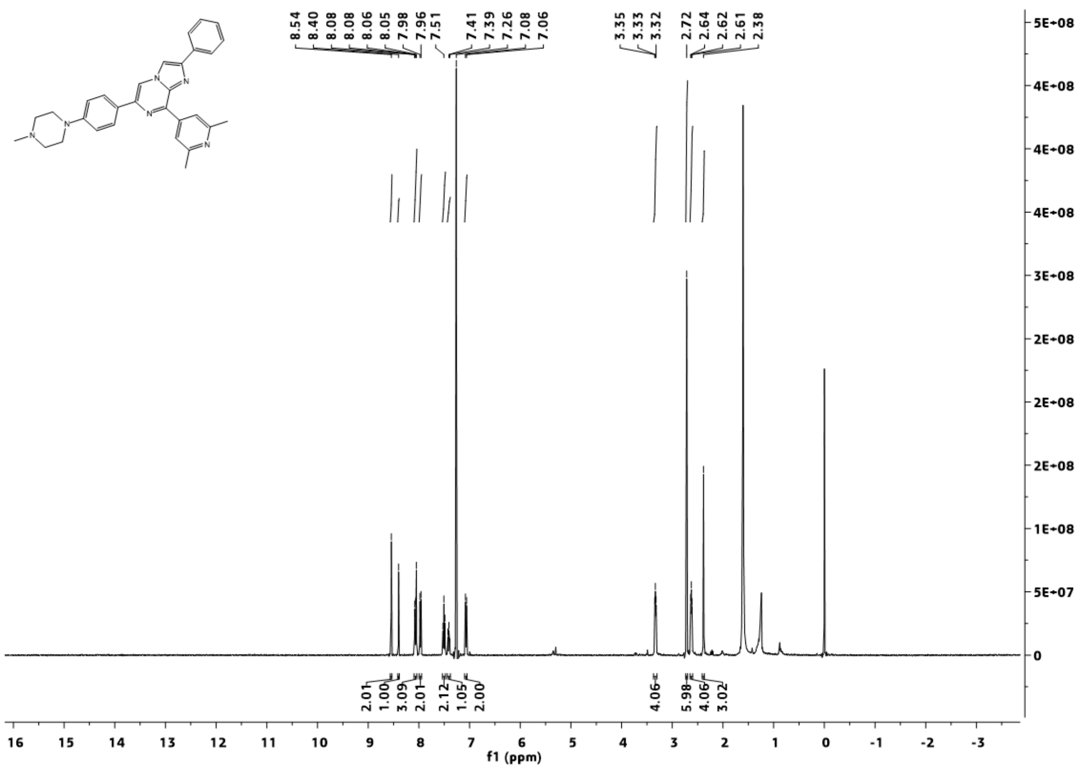

^{13}C NMR Spectra of **2e**

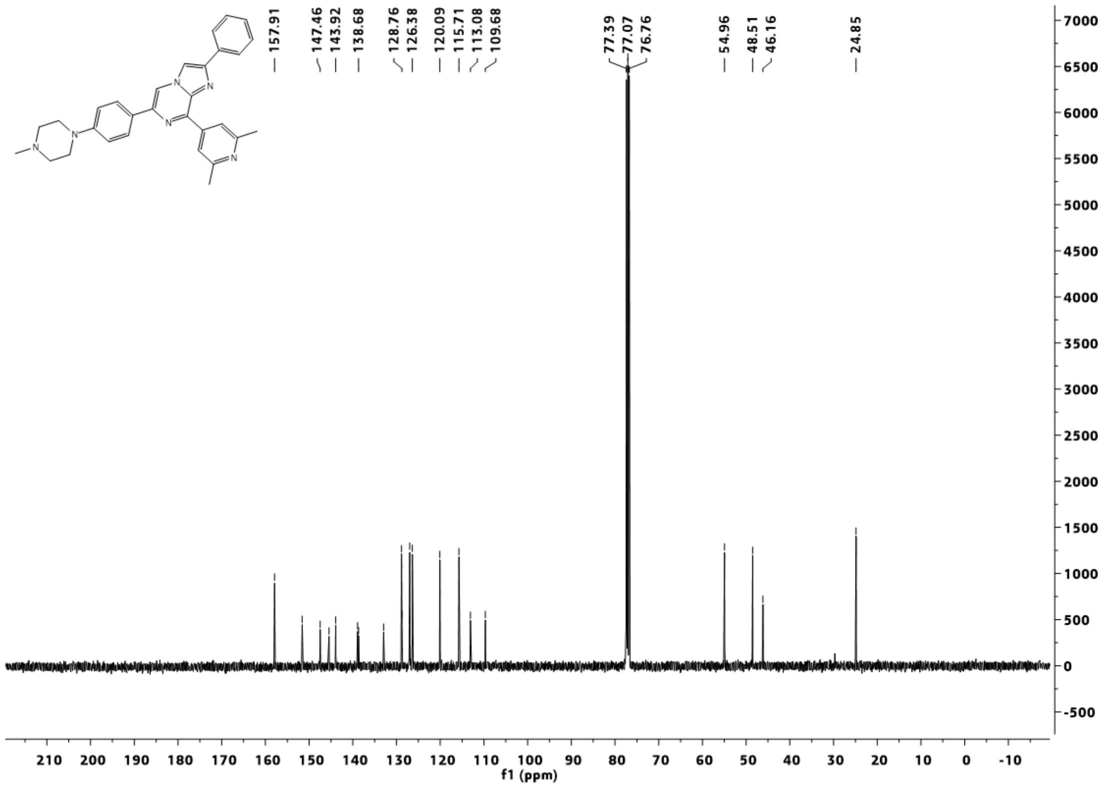

^{1}H NMR Spectra of **2f**

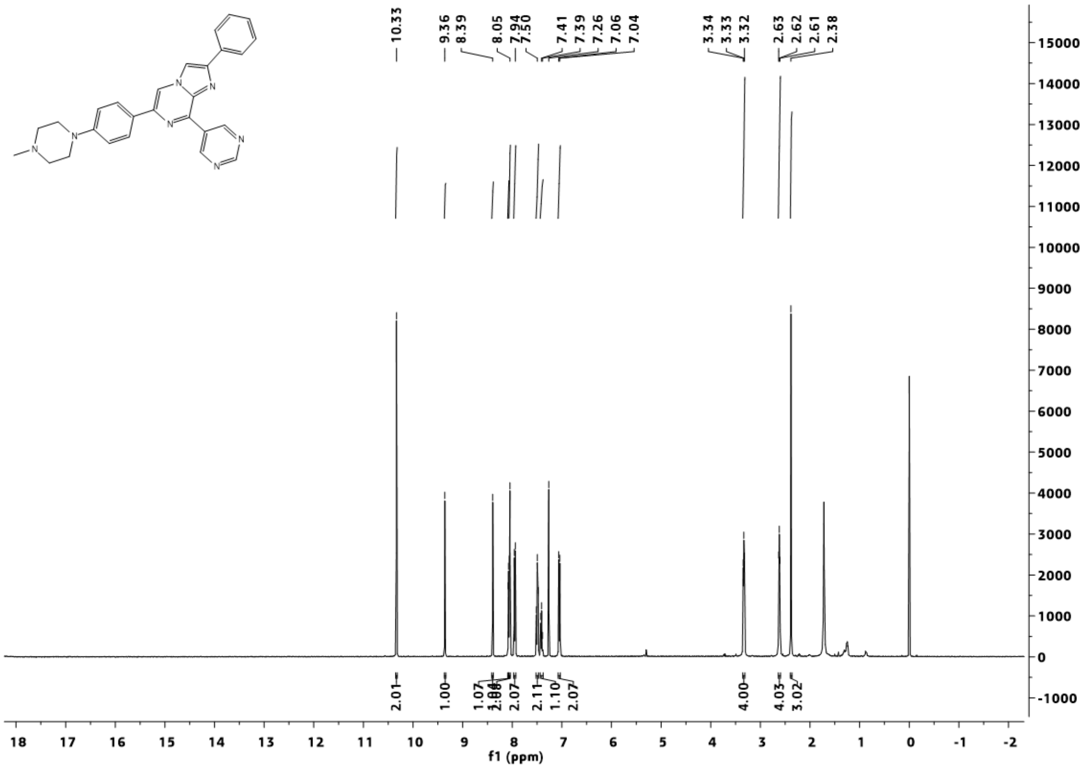

^{13}C NMR Spectra of **2f**

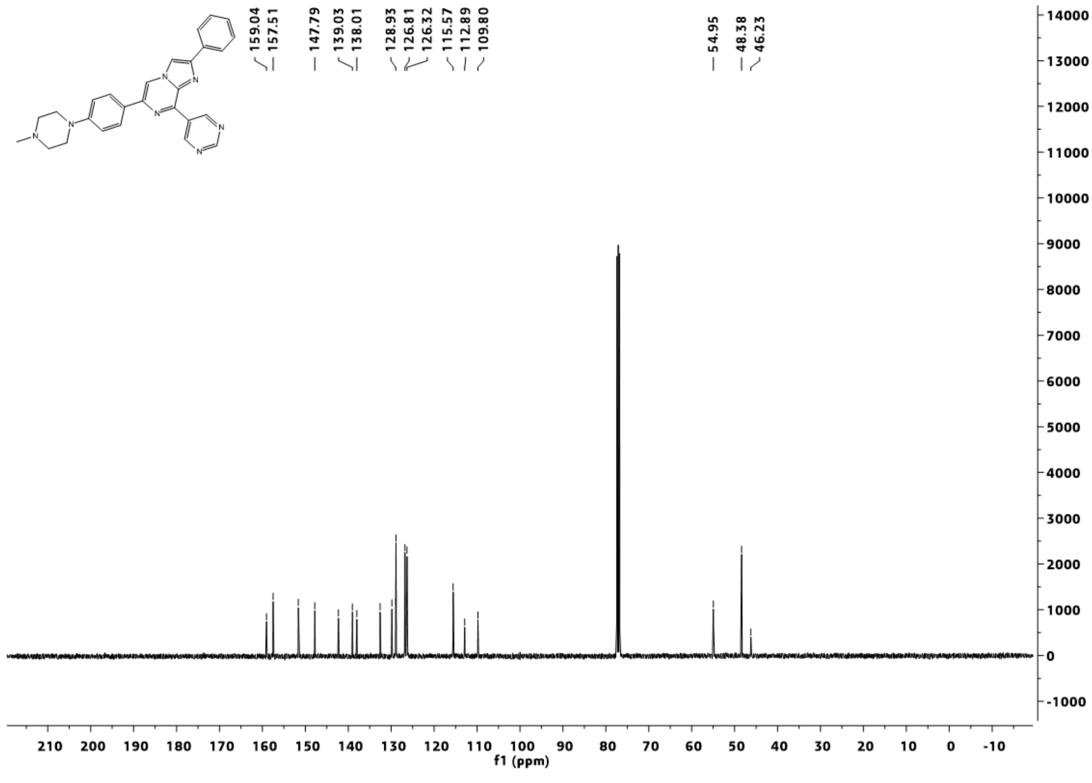

^{1}H NMR Spectra of **2g**

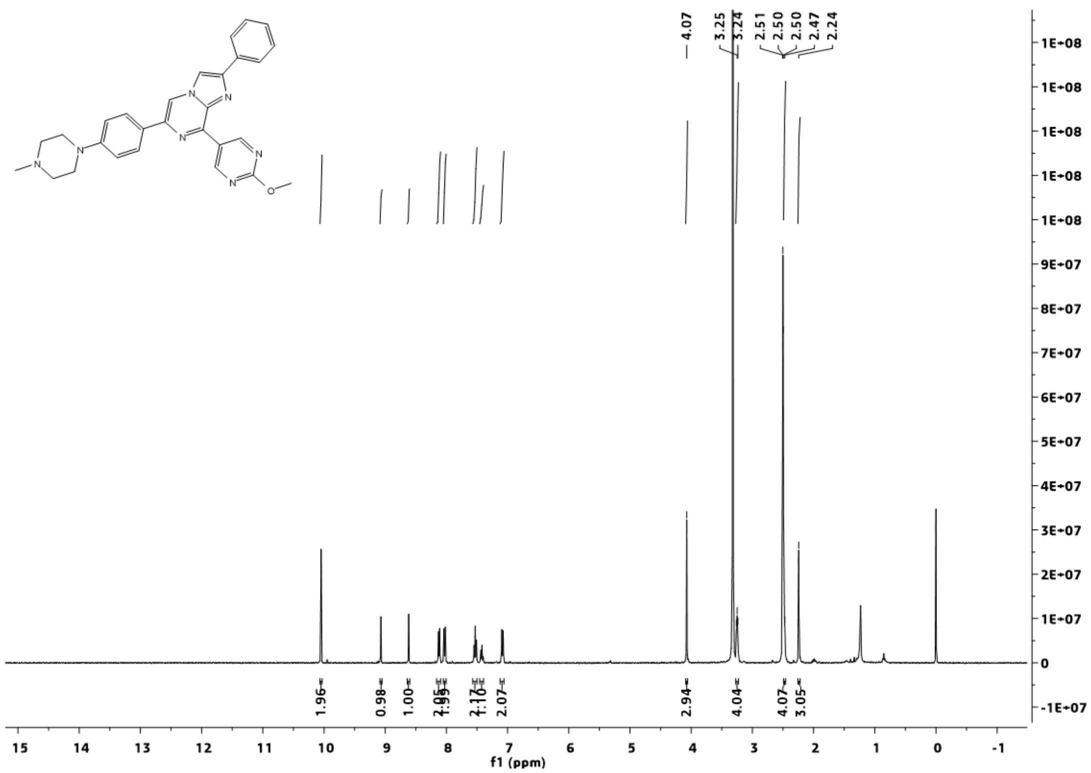

^{13}C NMR Spectra of **2g**

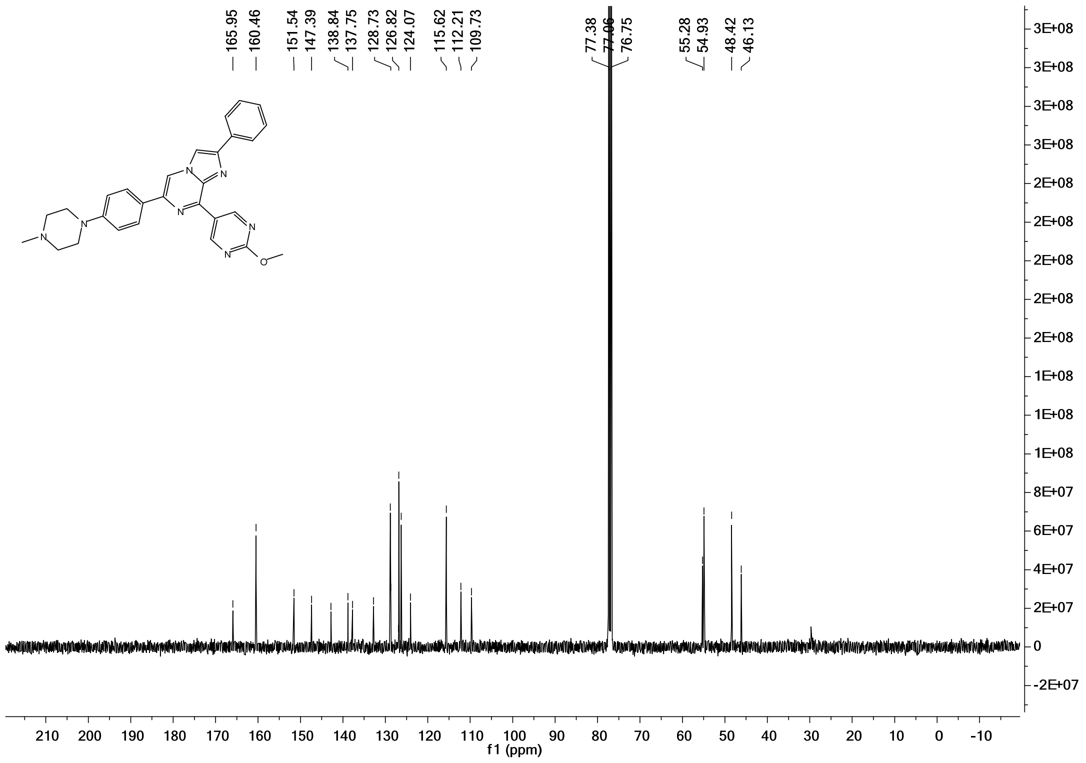

^{1}H NMR Spectra of **2h**

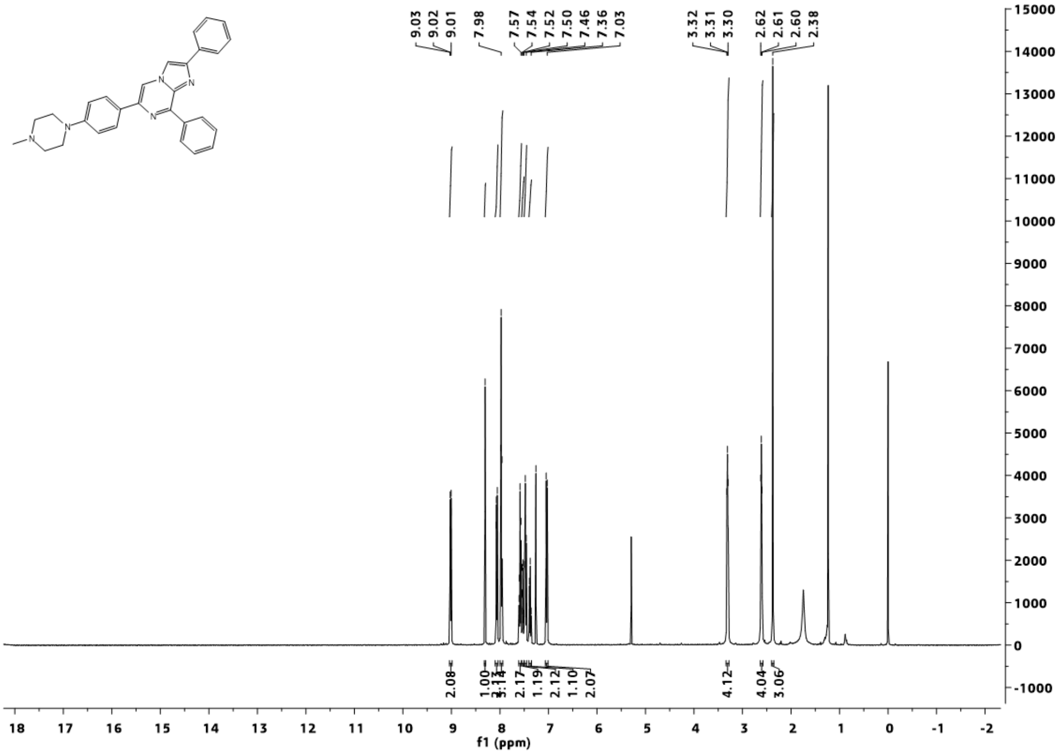

^{13}C NMR Spectra of **2h**

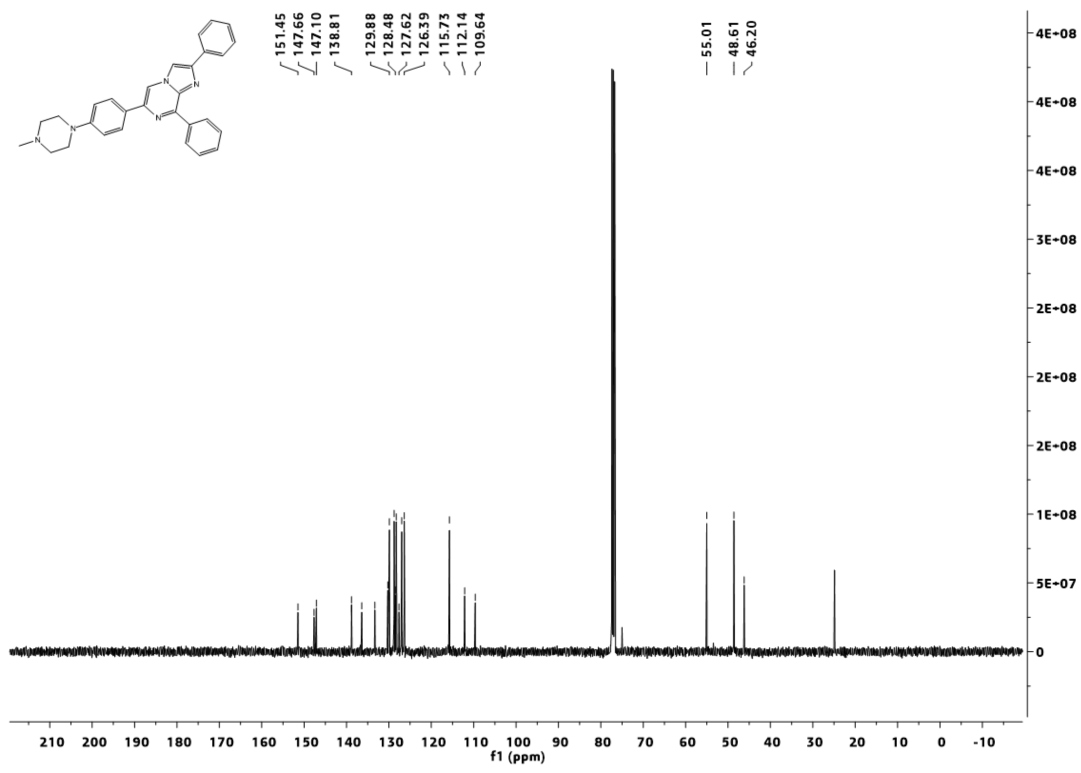

^{1}H NMR Spectra of **2i**

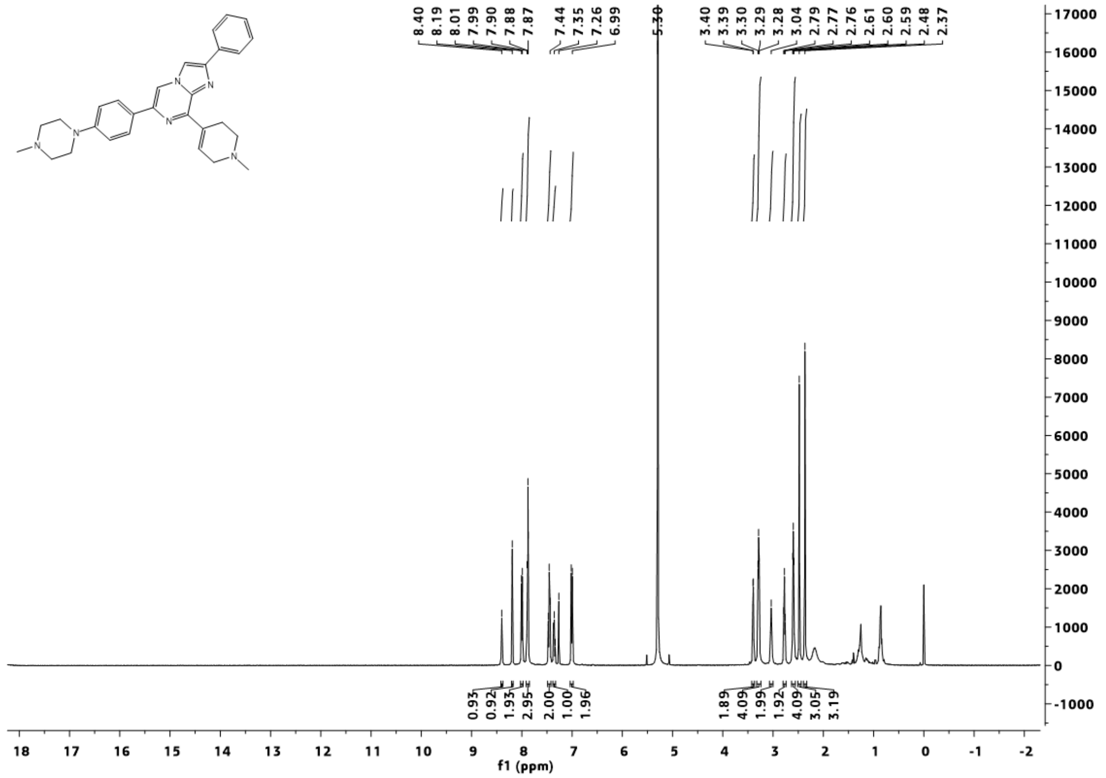

^{13}C NMR Spectra of **2i**

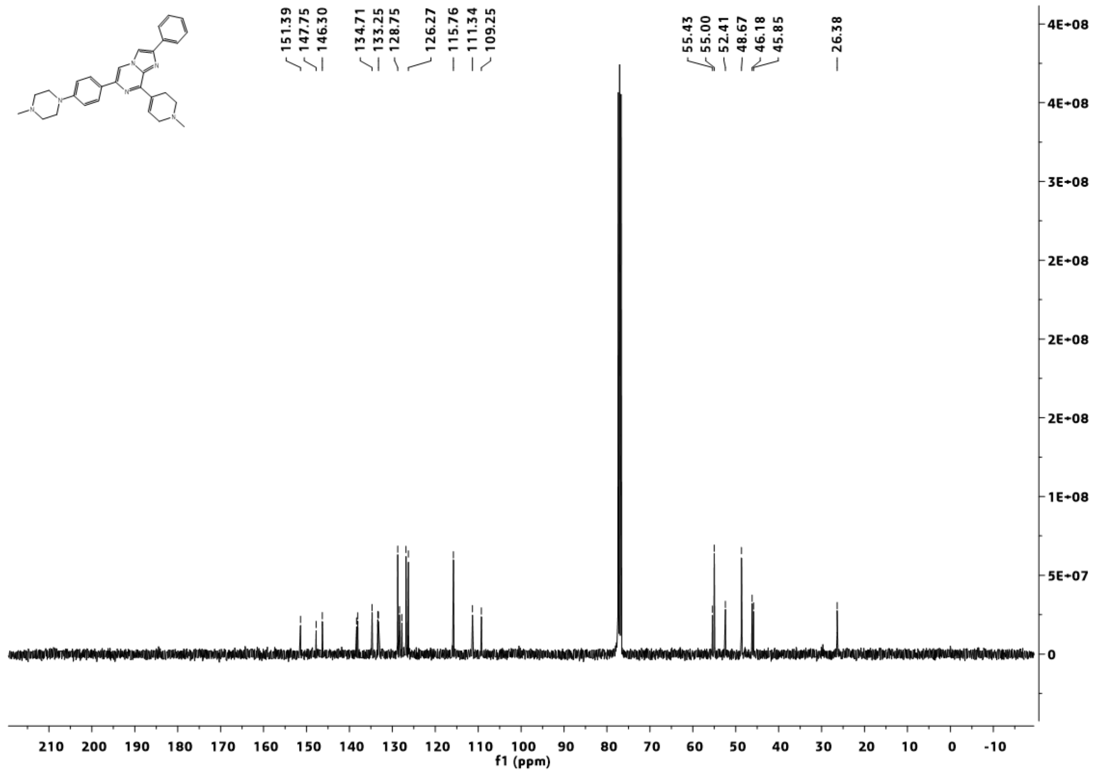

^{1}H NMR Spectra of **2j**

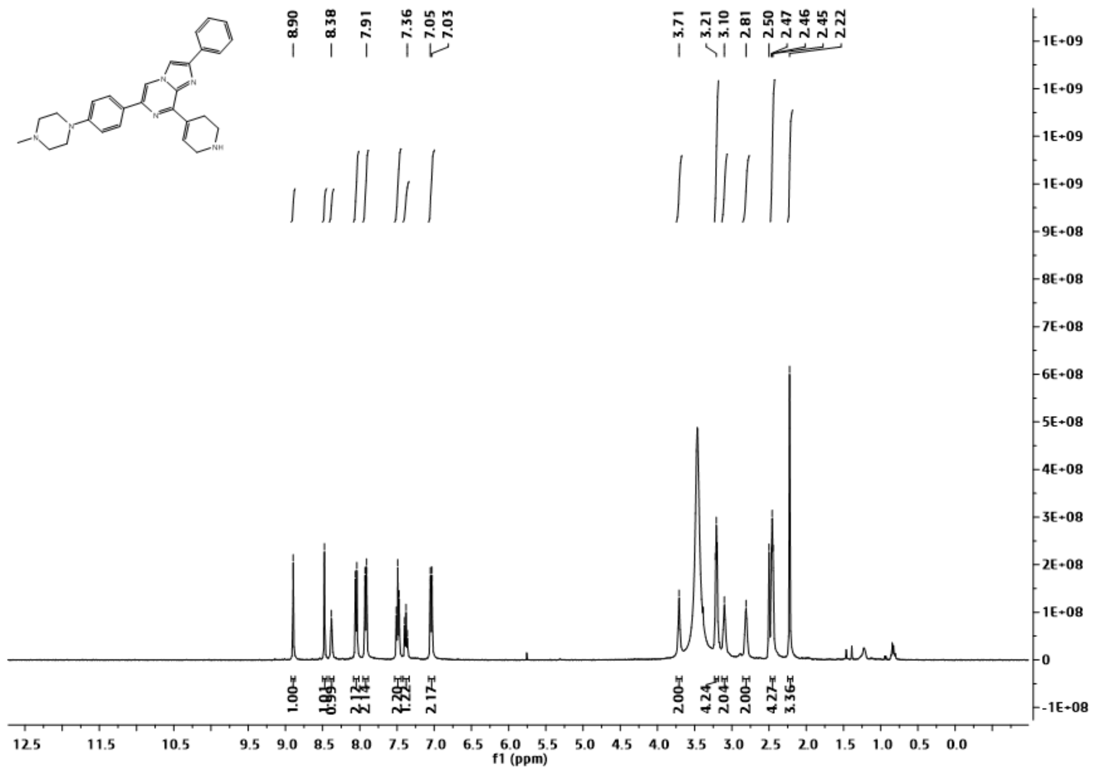

^{13}C NMR Spectra of **2j**

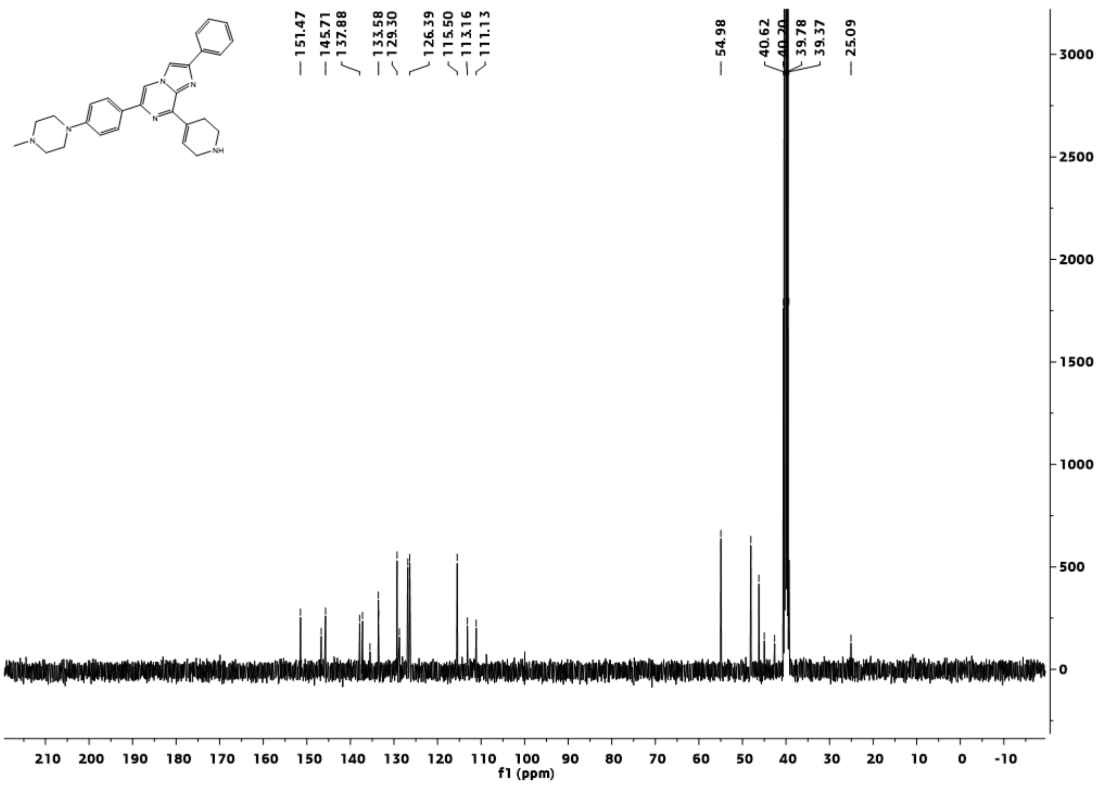

^{1}H NMR Spectra of **3a**

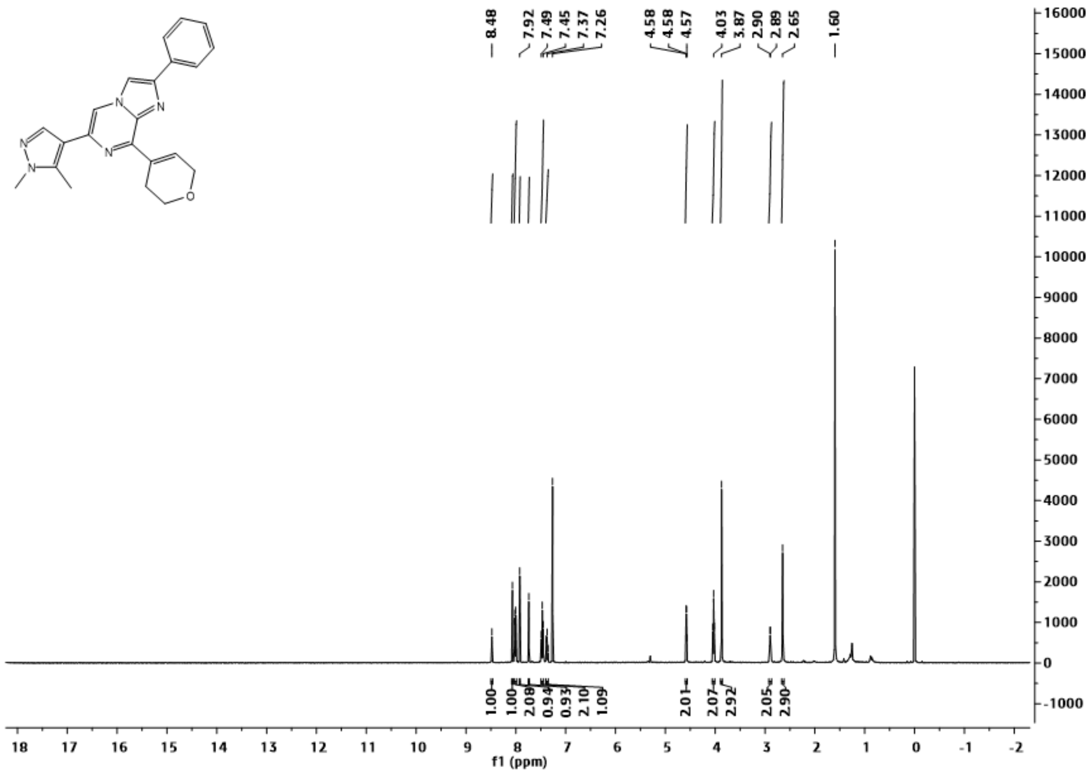

^{13}C NMR Spectra of **3a**

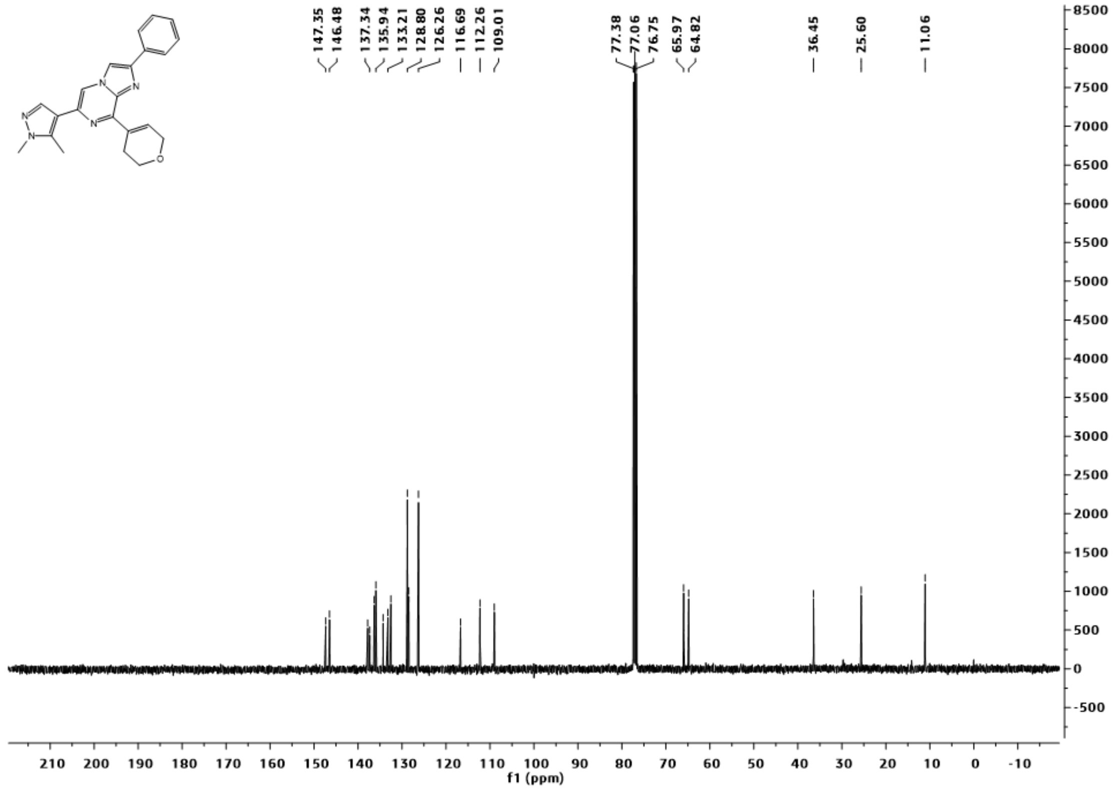

^{1}H NMR Spectra of **3b**

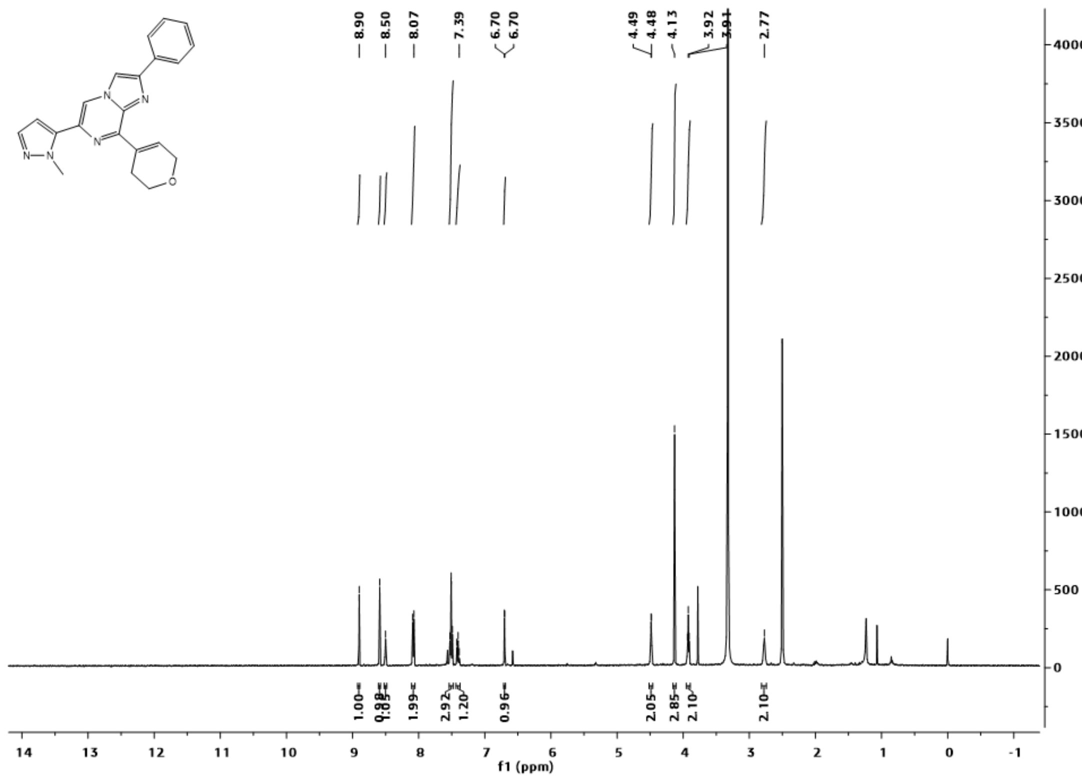

^{13}C NMR Spectra of **3b**

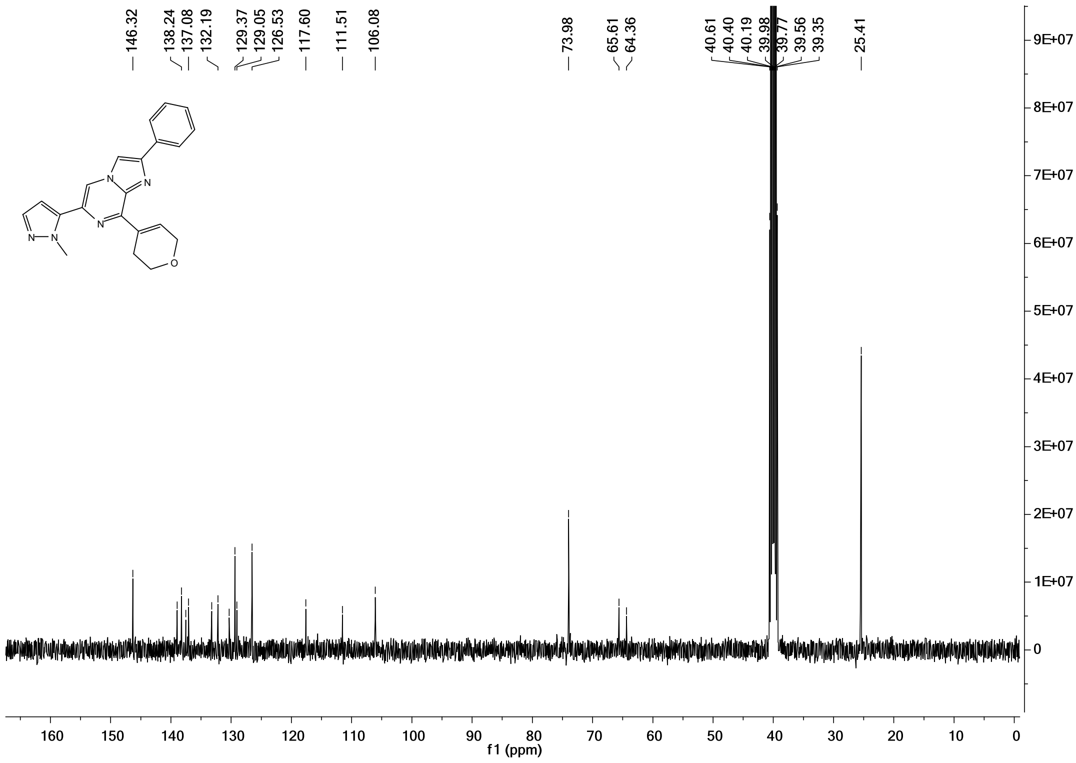

^{1}H NMR Spectra of **3c**

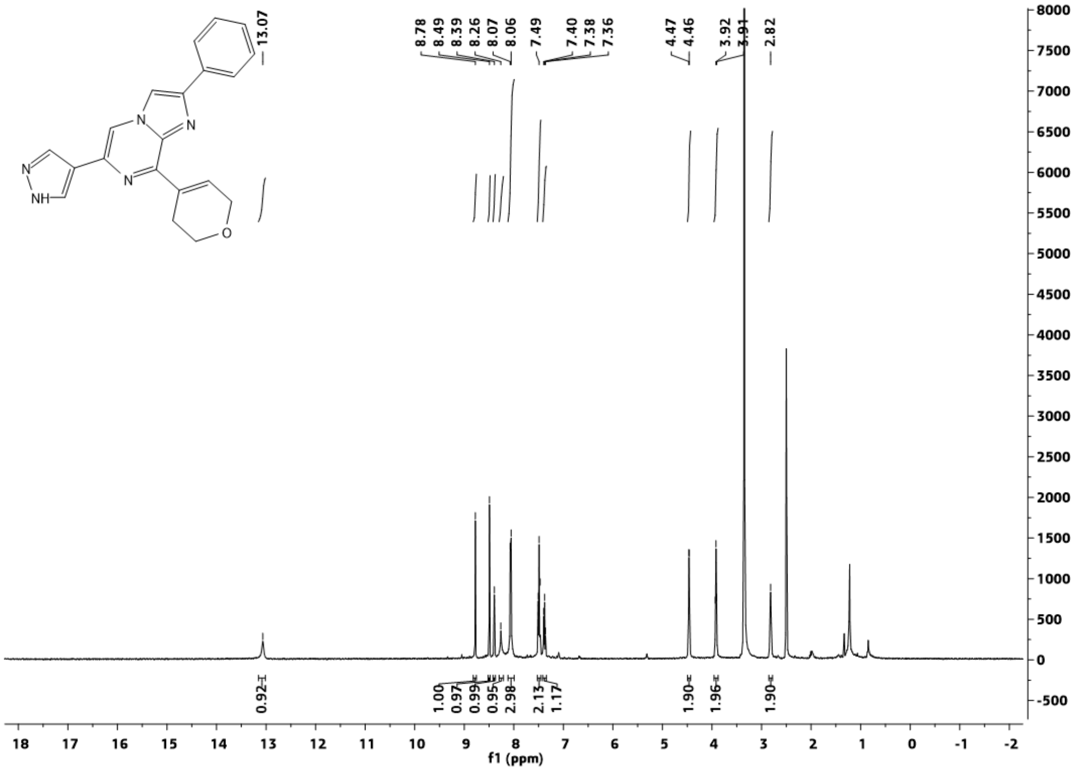

^{13}C NMR Spectra of **3c**

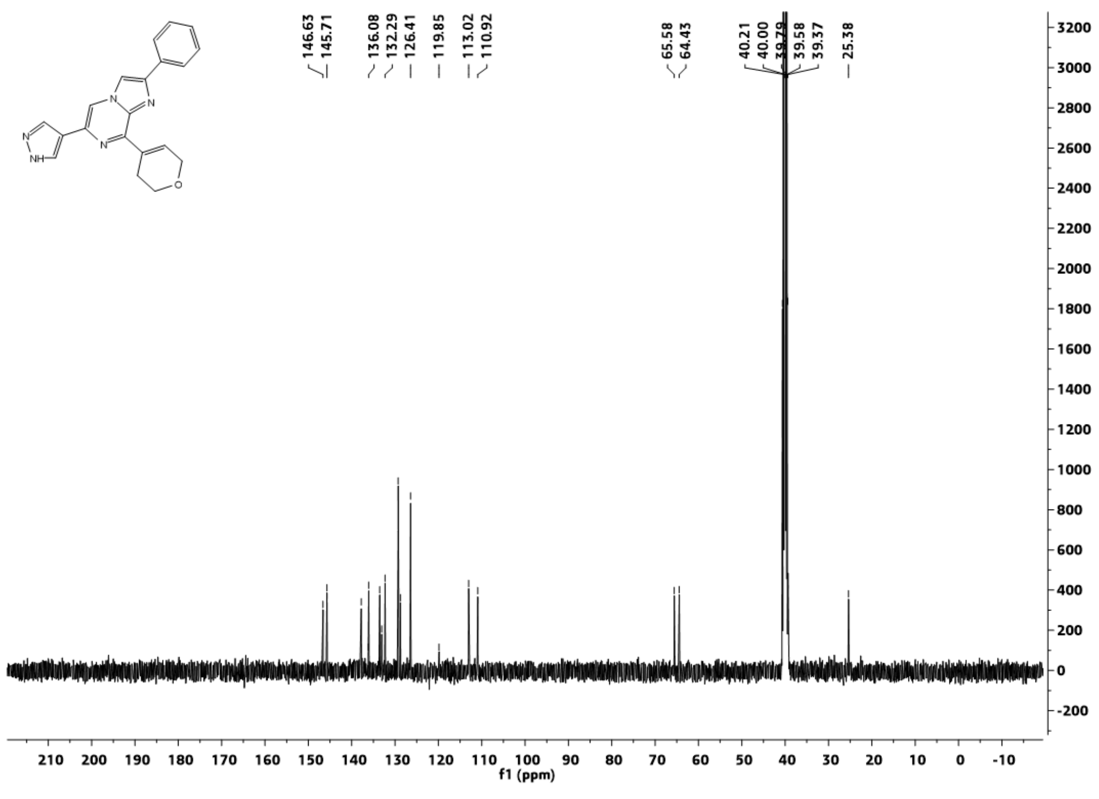

^{1}H NMR Spectra of **3d**

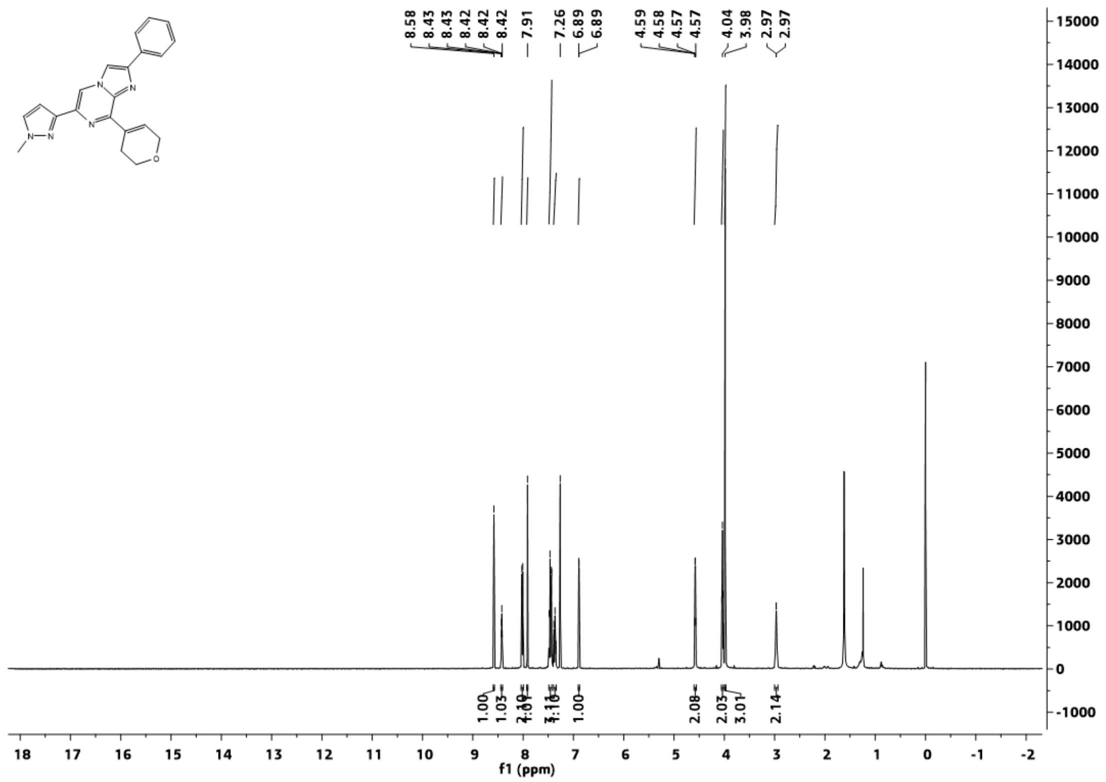

^{13}C NMR Spectra of **3d**

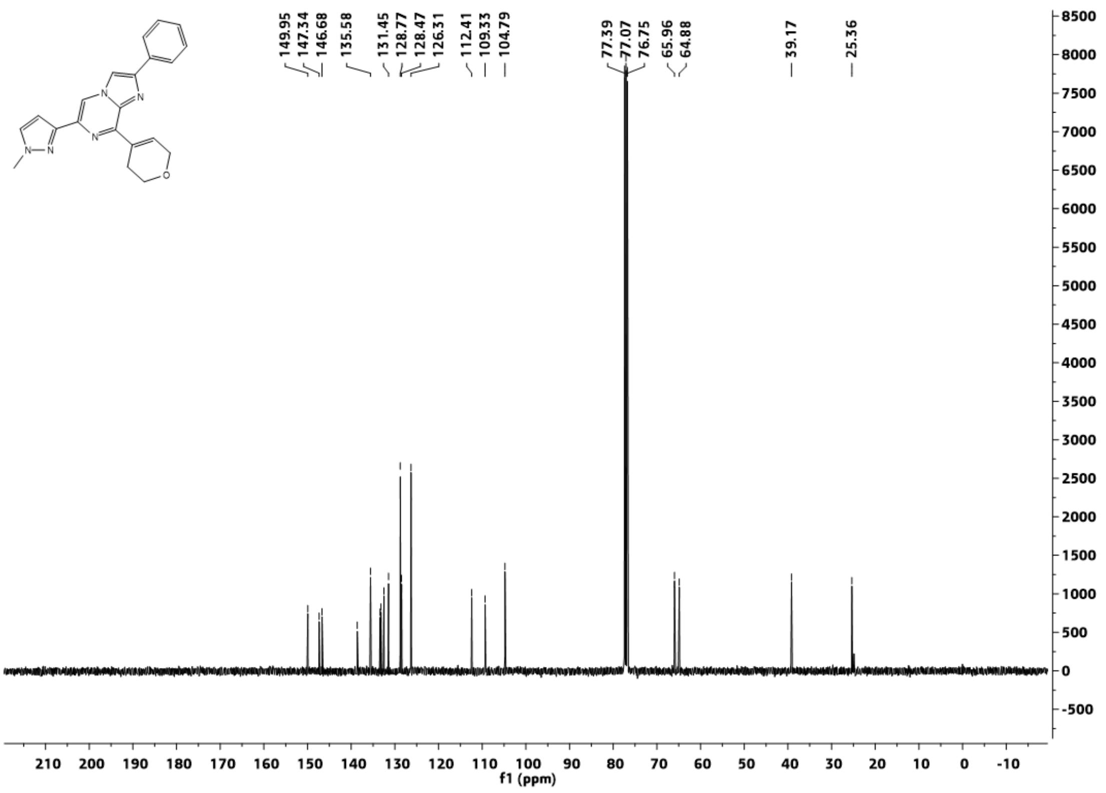

^{1}H NMR Spectra of **3e**

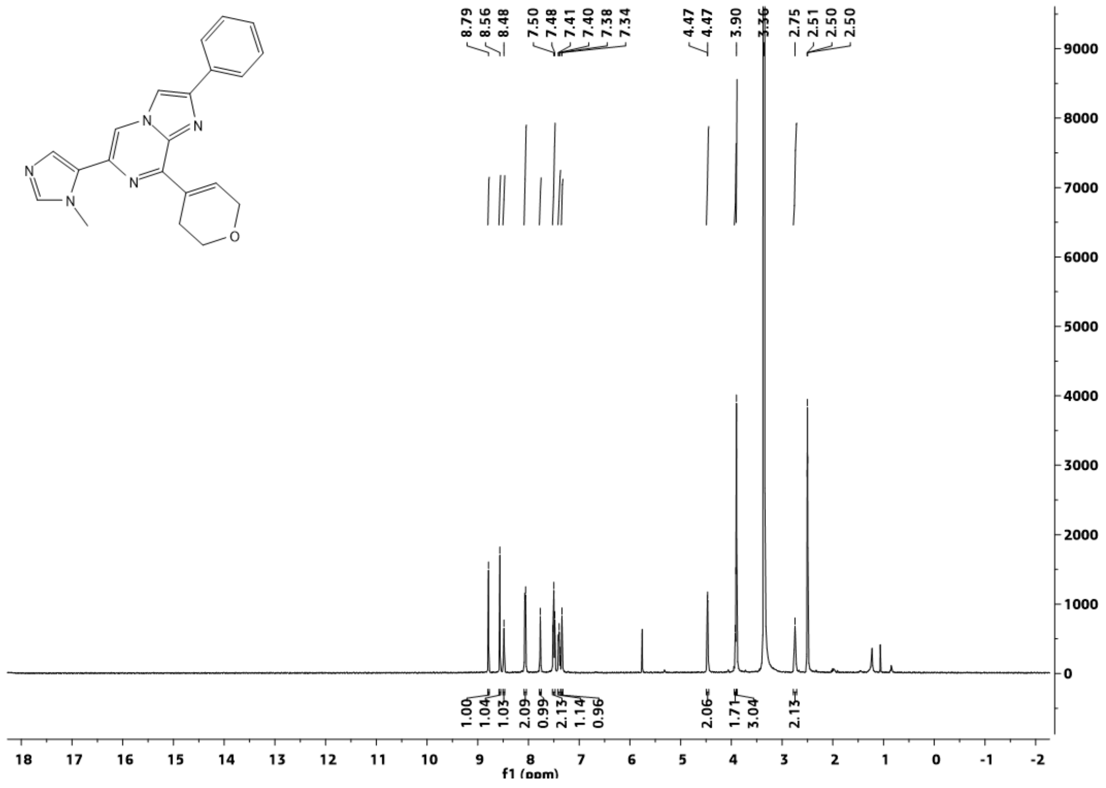

^{13}C NMR Spectra of **3e**

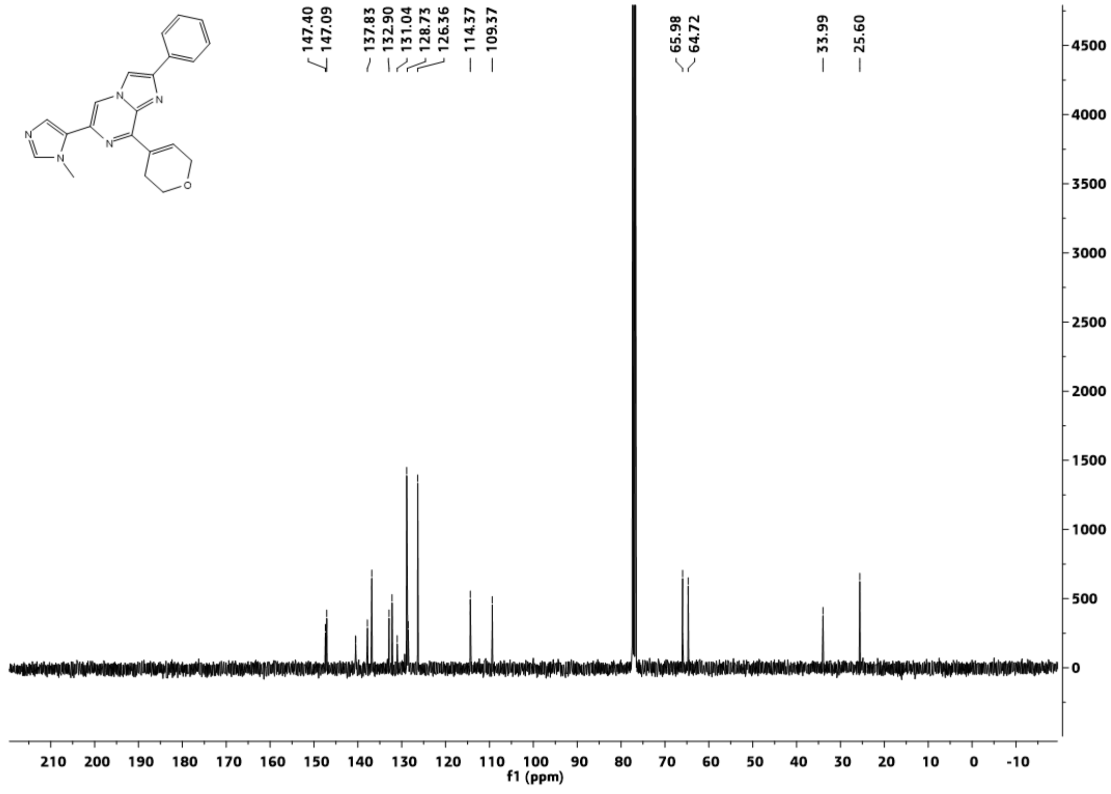

^{1}H NMR Spectra of **3f**

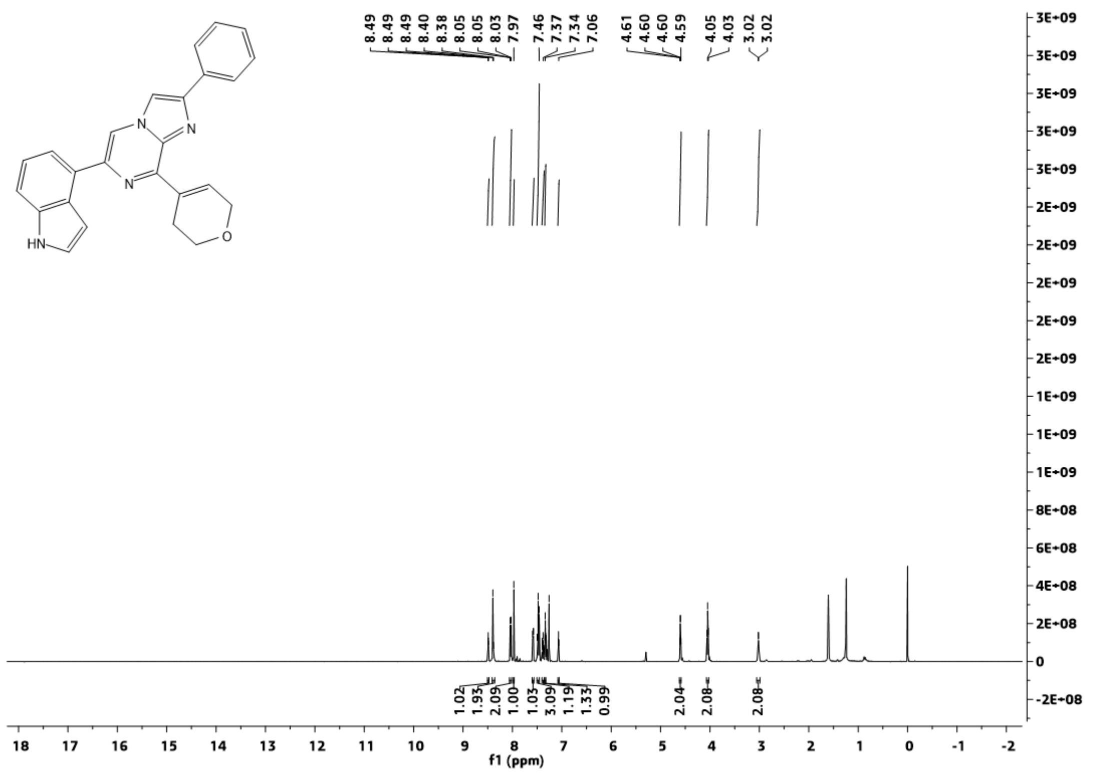

^{13}C NMR Spectra of **3f**

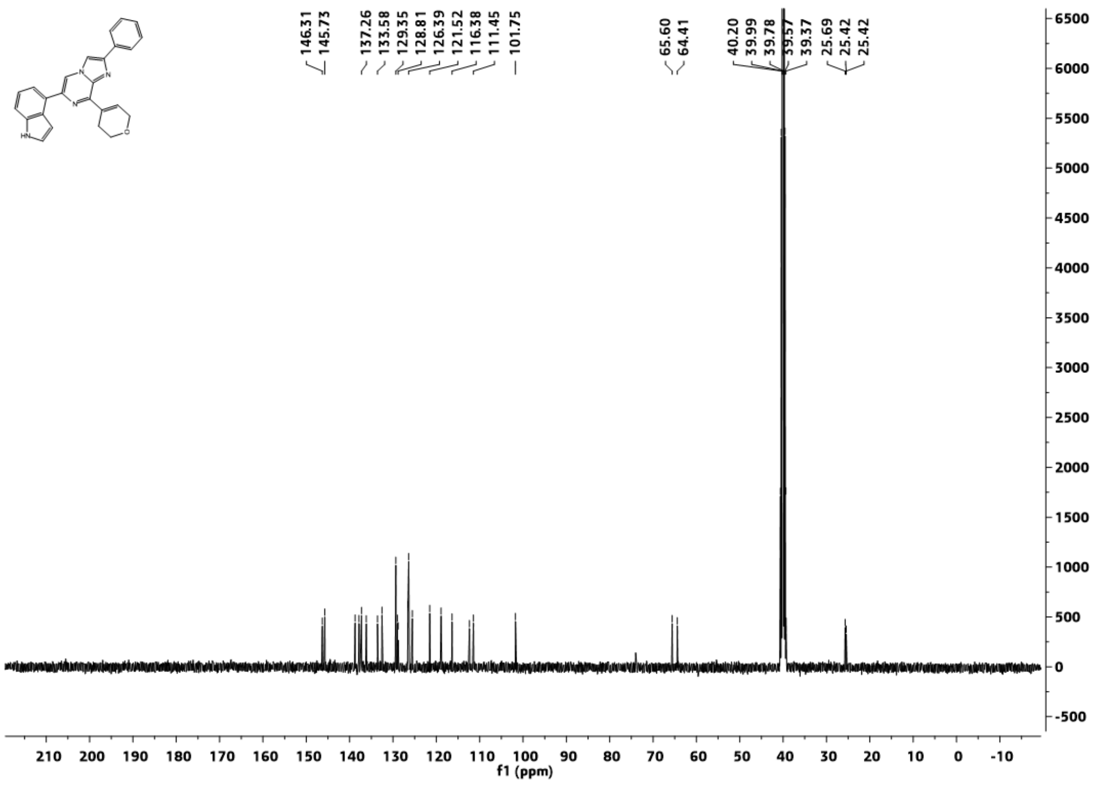

^{1}H NMR Spectra of YL-939

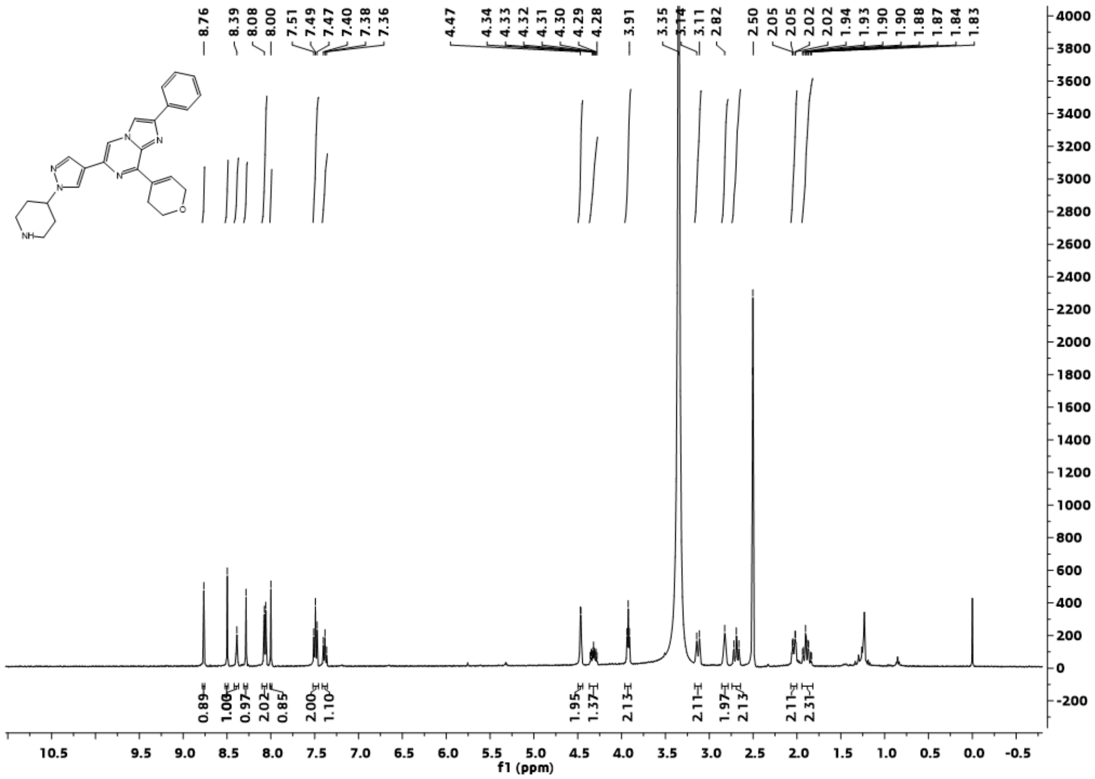

^{13}C NMR Spectra of YL-939

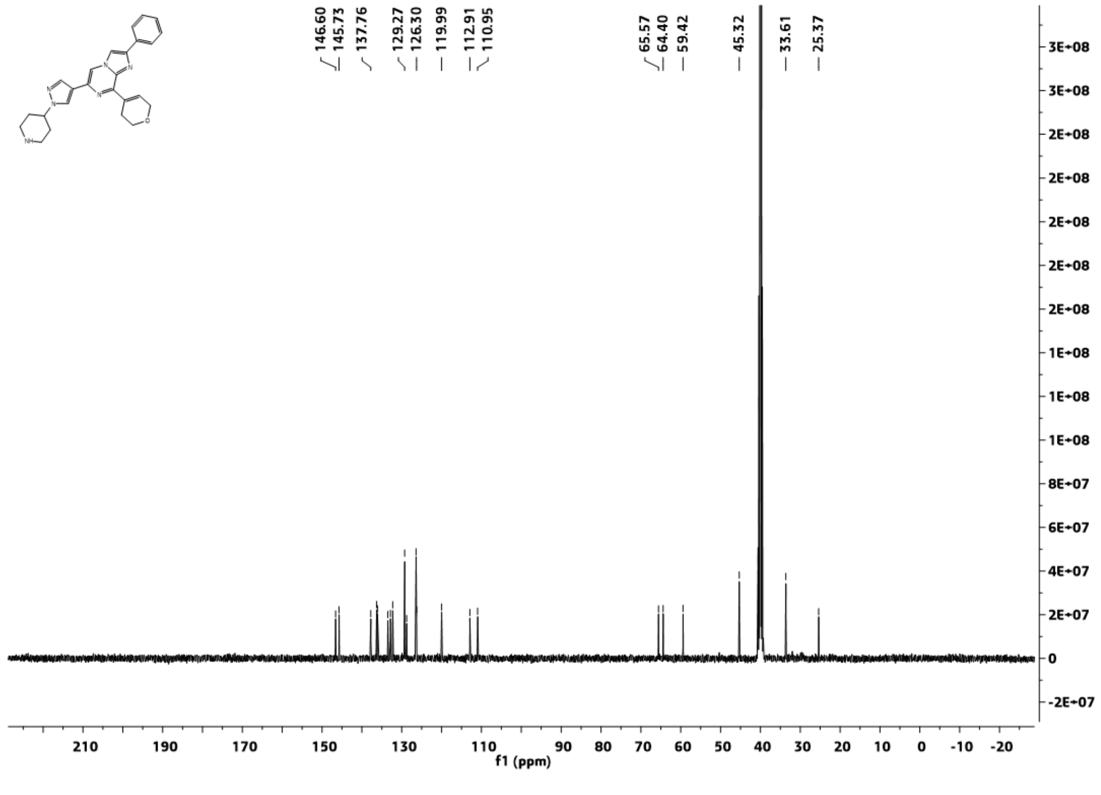

^{1}H NMR Spectra of YL-939-1

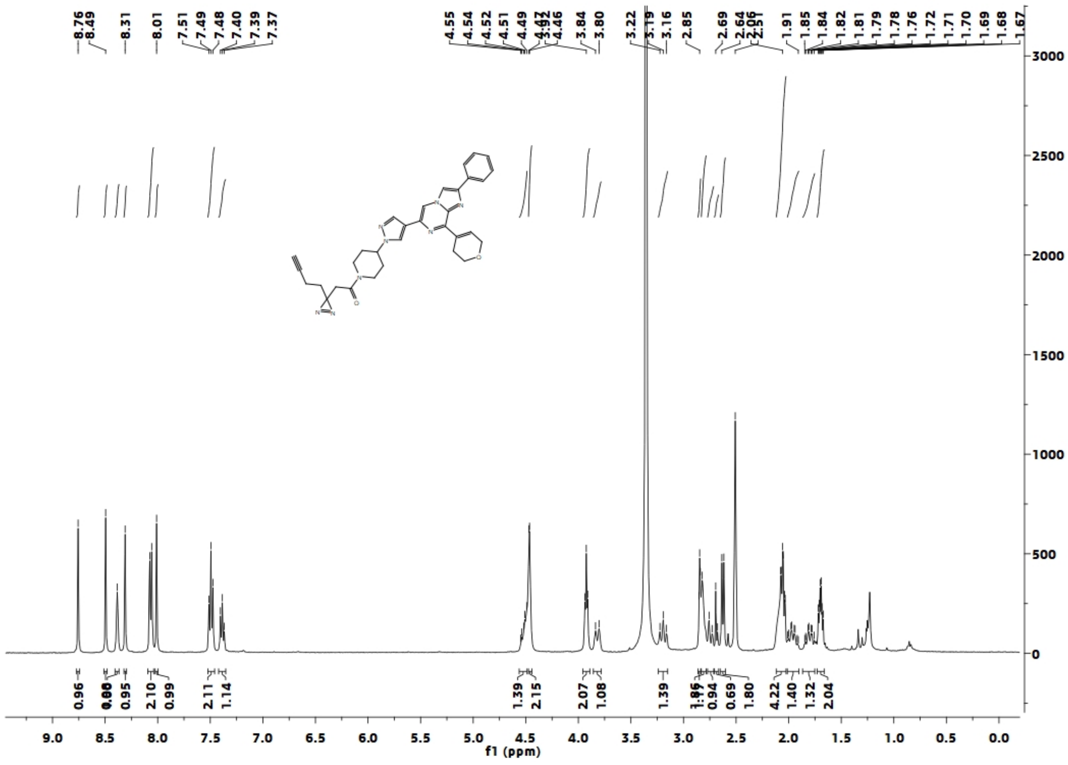

^{13}C NMR Spectra of YL-939-1

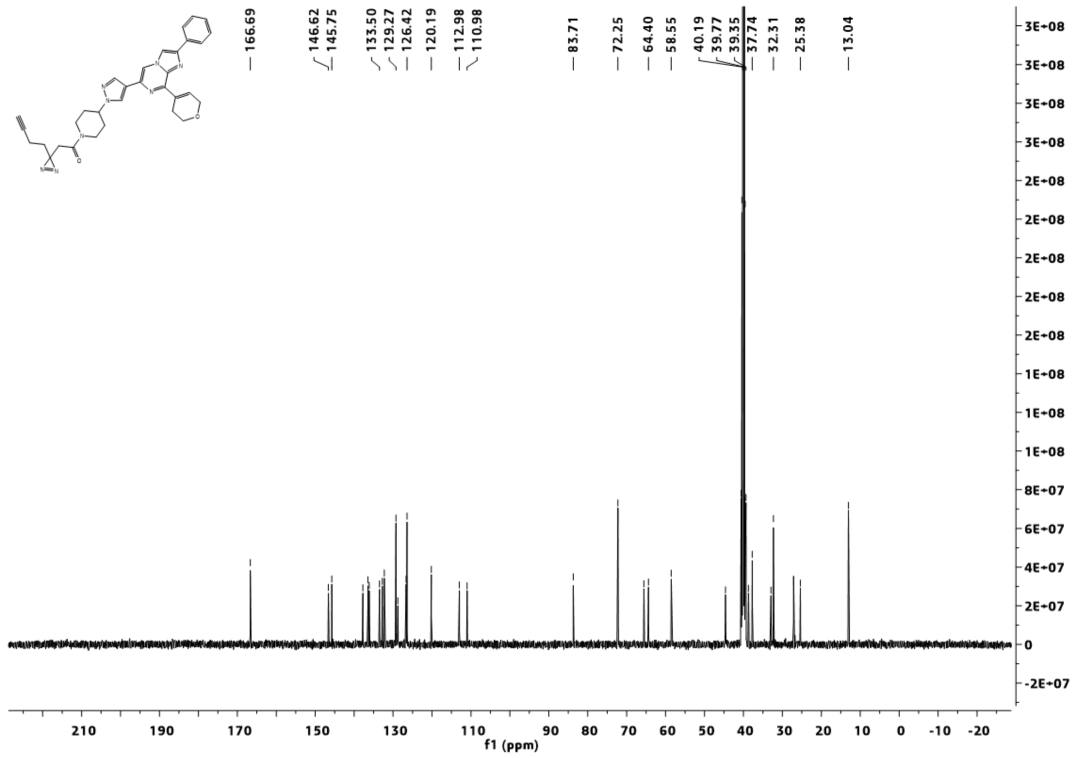

HRMS of all compounds.

Cpd-015

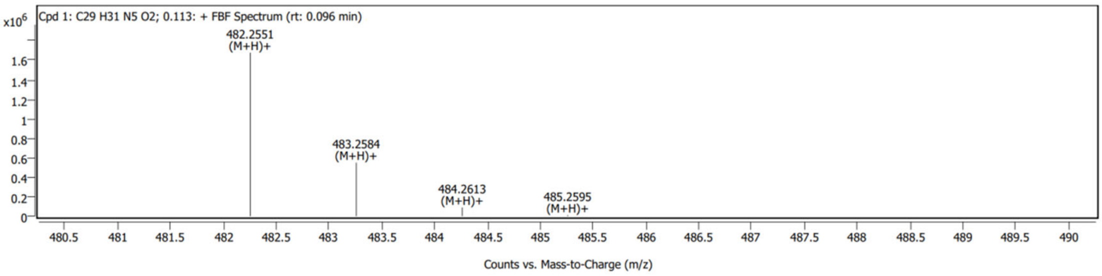

Spectrum Peaks

m/z	m/z (Calc)	Diff (ppm)	Abund	Height %	Height % (Calc)	Ion Species	Z
482.2551	482.2551	0.10	1678719	100.00	100.00	(M+H)+	1
483.2584	483.2581	0.70	549764	32.75	33.64	(M+H)+	1
484.2613	484.2610	0.68	90189	5.37	5.90	(M+H)+	1
485.2595	485.2638	-8.89	11981	0.71	0.72	(M+H)+	1

1a

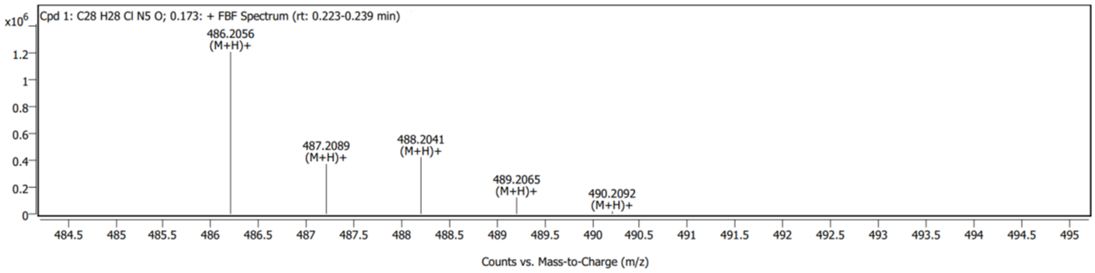

Spectrum Peaks

m/z	m/z (Calc)	Diff (ppm)	Abund	Height %	Height % (Calc)	Ion Species	Z
486.2056	486.2055	0.21	1205143	100.00	100.00	(M+H)+	1
487.2089	487.2085	0.75	371961	30.86	32.48	(M+H)+	1
488.2041	488.2038	0.46	422450	35.05	37.31	(M+H)+	1
489.2065	489.2061	0.90	123231	10.23	10.98	(M+H)+	1
490.2092	490.2088	0.84	19847	1.65	1.75	(M+H)+	1

1b

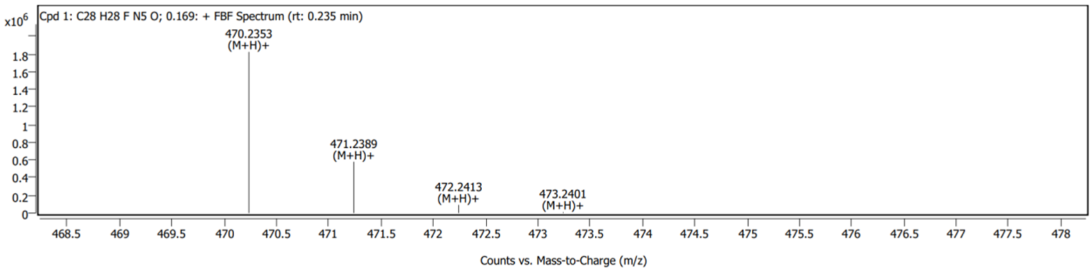

Spectrum Peaks

m/z	m/z (Calc)	Diff (ppm)	Abund	Height %	Height % (Calc)	Ion Species	Z
470.2353	470.2351	0.52	1823095	100.00	100.00	(M+H)+	1
471.2389	471.2381	1.62	580293	31.83	32.48	(M+H)+	1
472.2413	472.2410	0.54	92179	5.06	5.31	(M+H)+	1
473.2401	473.2439	-7.99	10842	0.59	0.58	(M+H)+	1

1c

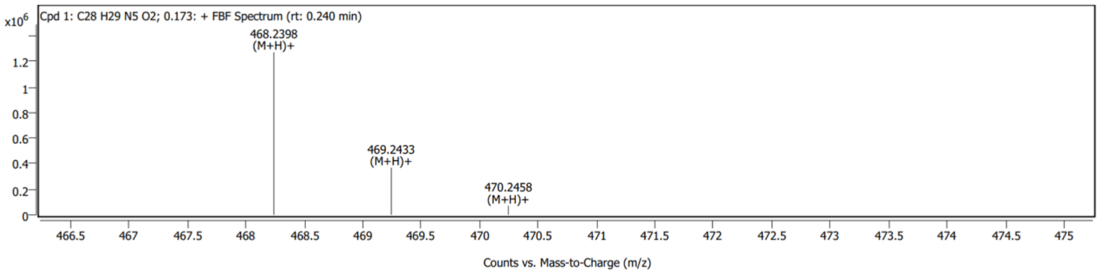

Spectrum Peaks

m/z	m/z (Calc)	Diff (ppm)	Abund	Height %	Height % (Calc)	Ion Species	Z
468.2398	468.2394	0.92	1273241	100.00	100.00	(M+H)+	1
469.2433	469.2424	1.88	368784	28.96	32.53	(M+H)+	1
470.2458	470.2453	0.96	71781	5.64	5.54	(M+H)+	1

1d

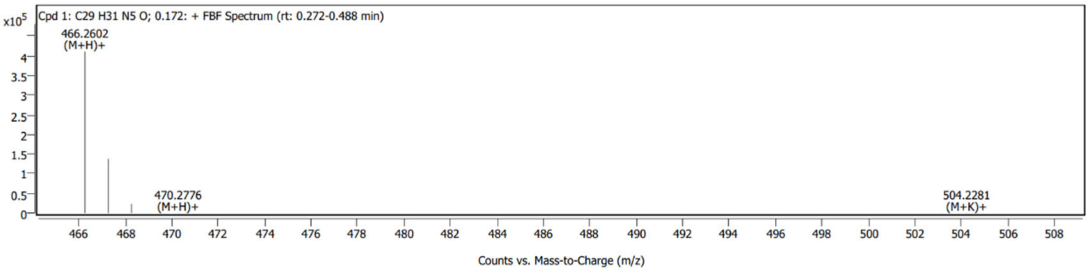

Spectrum Peaks

m/z	m/z (Calc)	Diff (ppm)	Abund	Height %	Height % (Calc)	Ion Species	Z
466.2602	466.2601	0.11	410646	100.00	100.00	(M+H)+	1
467.2633	467.2632	0.36	138173	33.65	33.60	(M+H)+	1
468.2656	468.2661	-1.08	23341	5.68	5.68	(M+H)+	1
470.2776	470.2719	12.20	207	0.05	0.06	(M+H)+	1
504.2281	504.2160	24.02	410	100.00	100.00	(M+K)+	1

1e

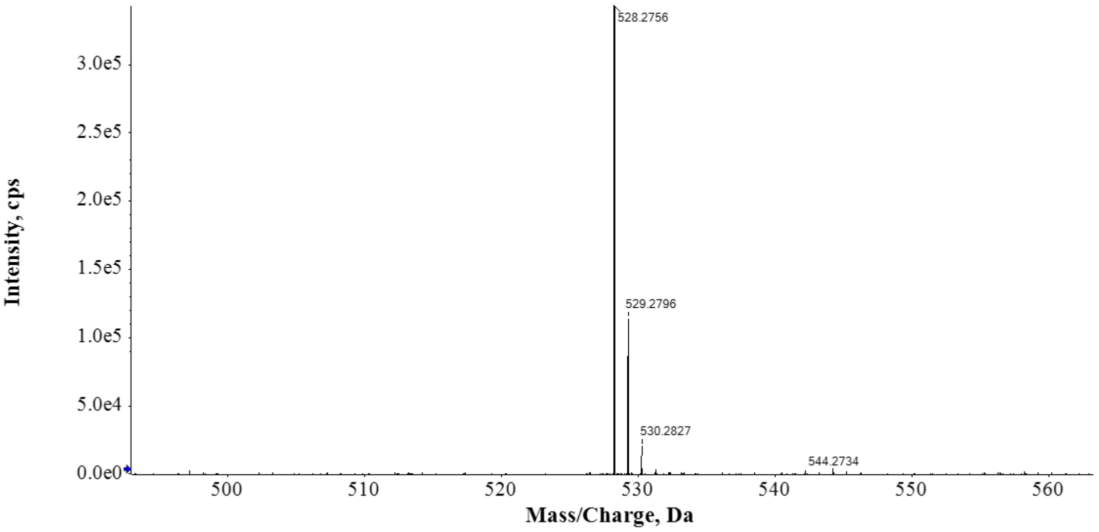

1f

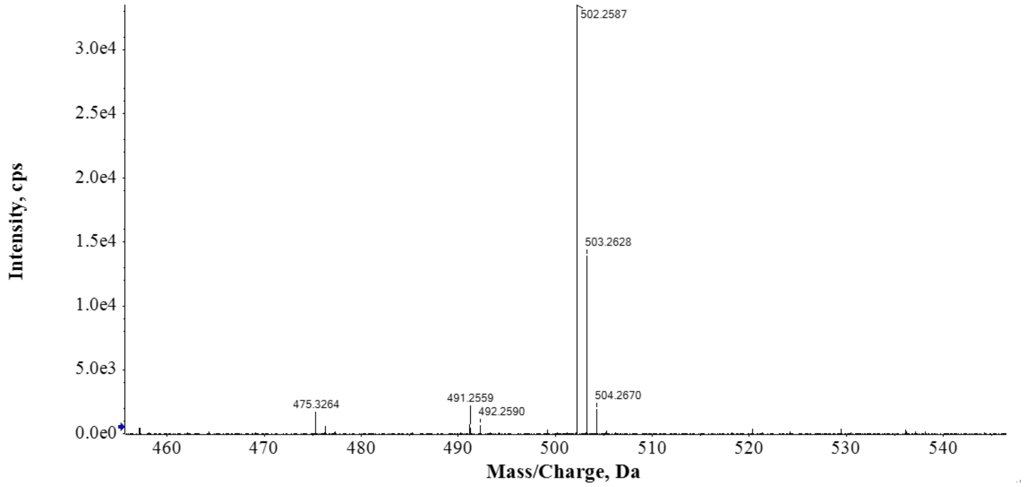

502.2587
503.2628
475.3264
491.2559
492.2590
504.2670
3.0e4
2.5e4
2.0e4
1.5e4
1.0e4
5.0e3
0.0e0
Intensity, cps
460
470
480
490
500
510
520
530
540
Mass/Charge, Da

1g

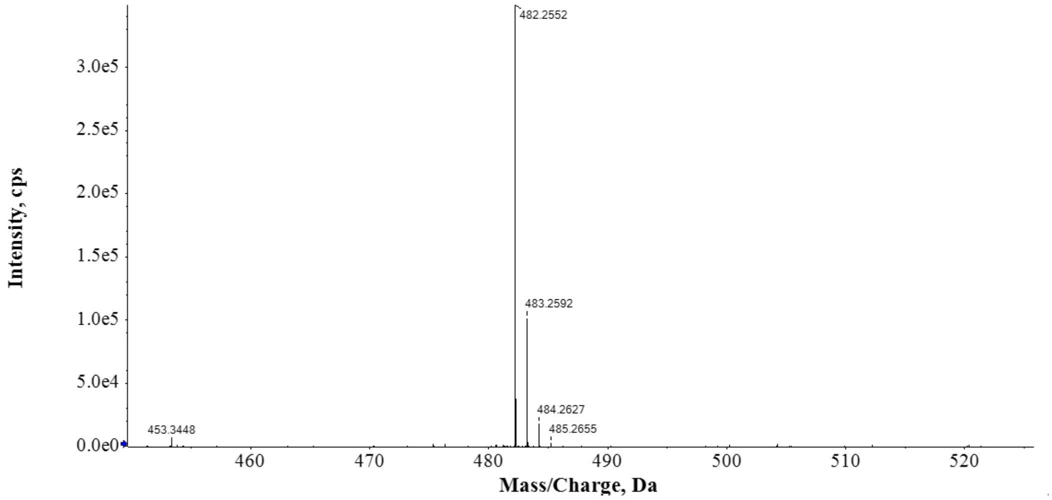

482.2552
483.2592
484.2627
485.2655
453.3448
3.0e5
2.5e5
2.0e5
1.5e5
1.0e5
5.0e4
0.0e0
Intensity, cps
460
470
480
490
500
510
520
Mass/Charge, Da

1h

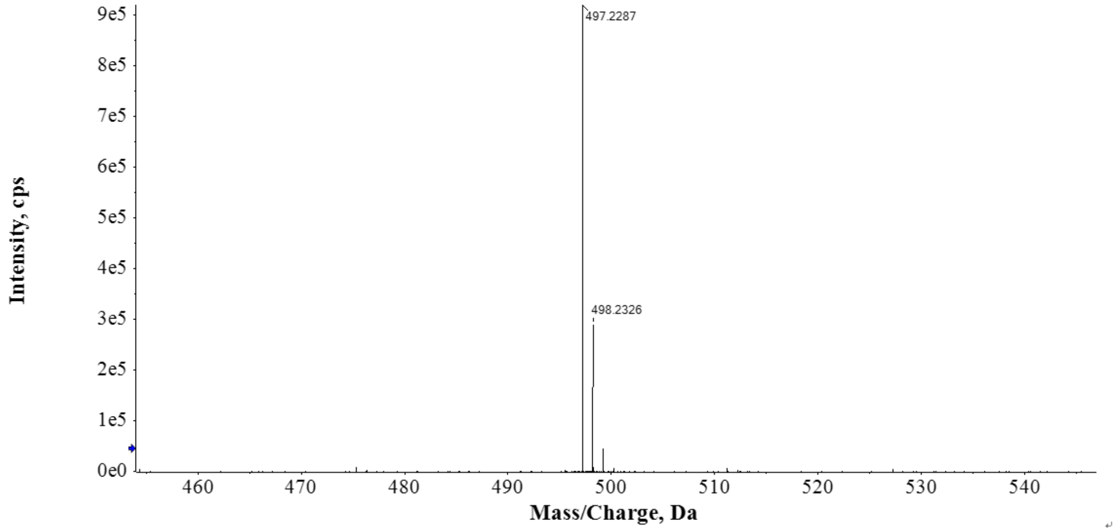

497.2287
498.2326
9e5
8e5
7e5
6e5
5e5
4e5
3e5
2e5
1e5
0e0
Intensity, cps
460
470
480
490
500
510
520
530
540
Mass/Charge, Da

1i

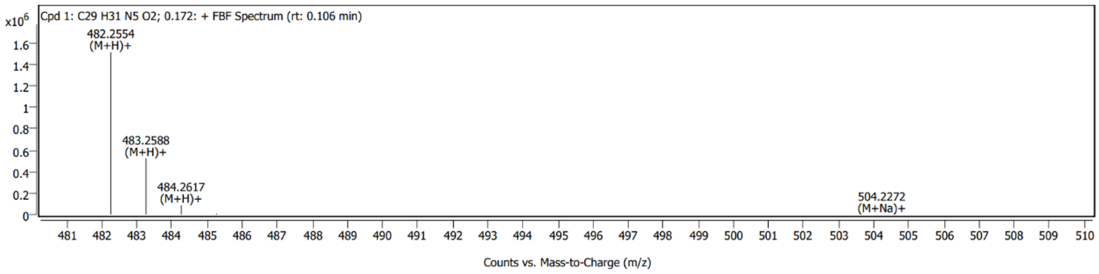

Spectrum Peaks

m/z	m/z (Calc)	Diff (ppm)	Abund	Height %	Height % (Calc)	Ion Species	Z
482.2554	482.2551	0.78	1516446	100.00	100.00	(M+H)+	1
483.2588	483.2581	1.46	524905	34.61	33.64	(M+H)+	1
484.2617	484.2610	1.43	86580	5.71	5.90	(M+H)+	1
485.2635	485.2638	-0.60	9263	0.61	0.72	(M+H)+	1
504.2272	504.2370	-19.48	1672	100.00	100.00	(M+Na)+	1

1j

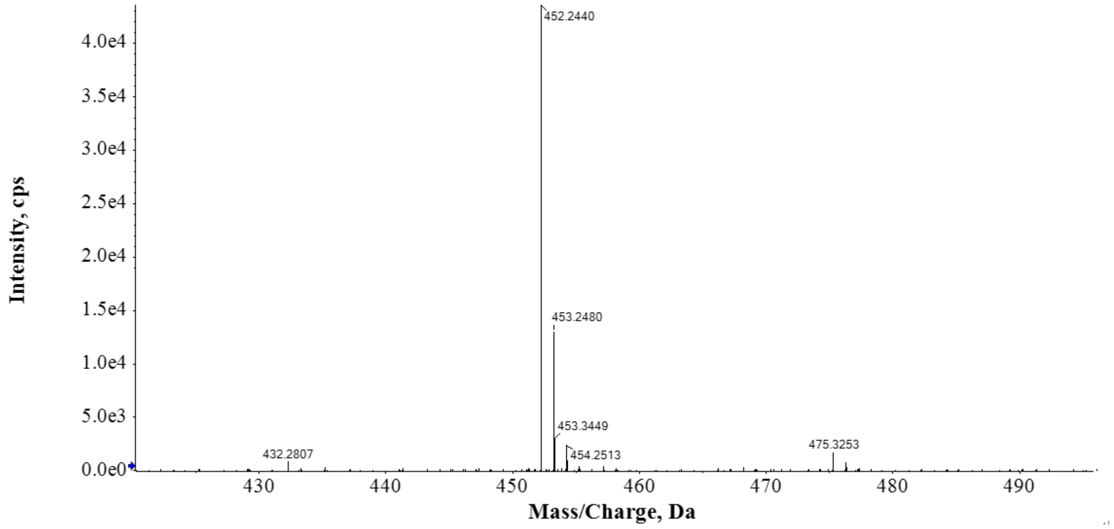

2a

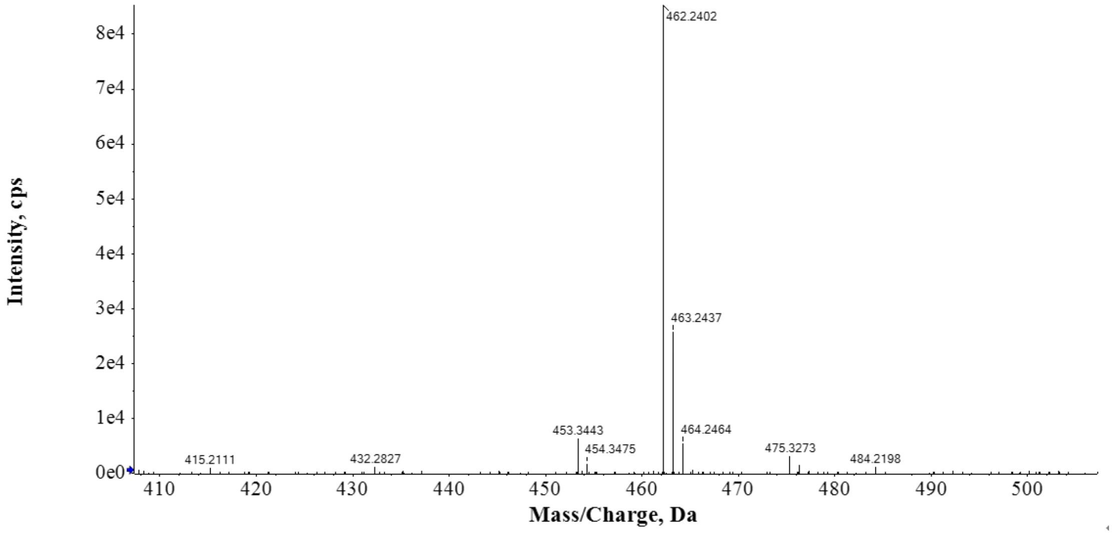

2b

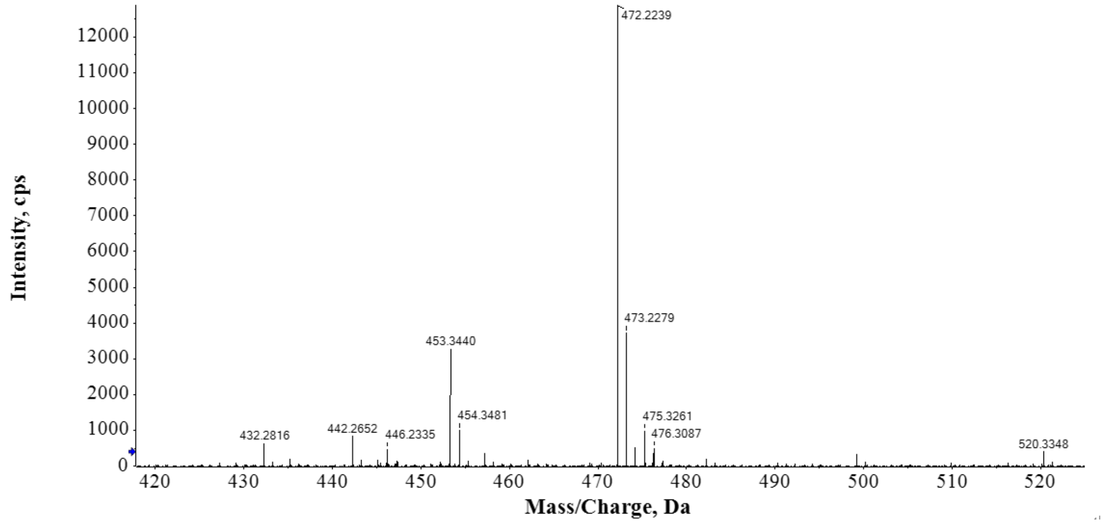

472.2239
473.2279
453.3440
454.3481
475.3261
476.3087
432.2816
442.2652
446.2335
520.3348
12000
11000
10000
9000
8000
7000
6000
5000
4000
3000
2000
1000
0
420
430
440
450
460
470
480
490
500
510
520
Intensity, cps
Mass/Charge, Da

2c

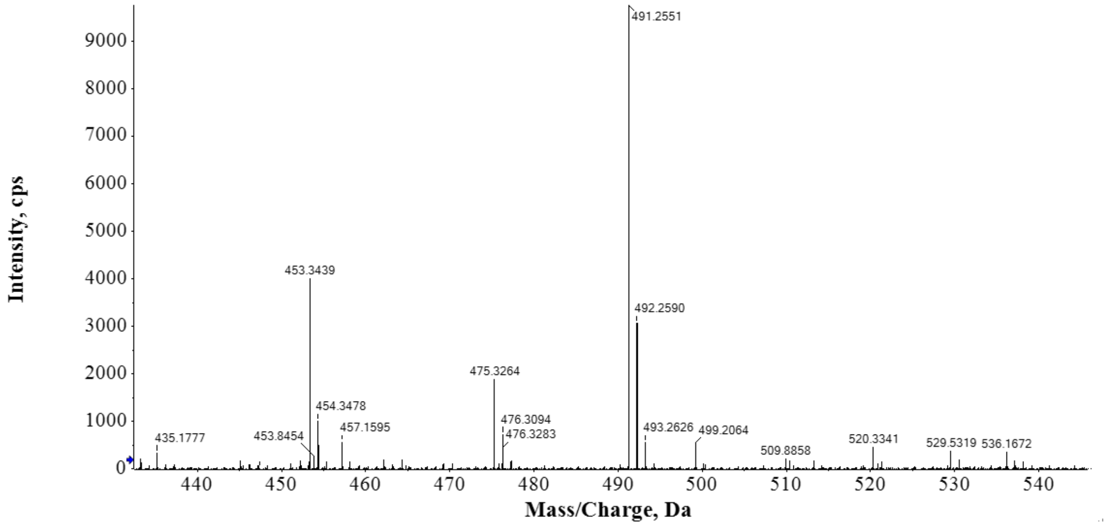

491.2551
492.2590
453.3439
475.3264
454.3478
457.1595
453.8454
435.1777
476.3094
476.3283
493.2626
499.2064
509.8858
520.3341
529.5319
536.1672
9000
8000
7000
6000
5000
4000
3000
2000
1000
0
440
450
460
470
480
490
500
510
520
530
540
Intensity, cps
Mass/Charge, Da

2d

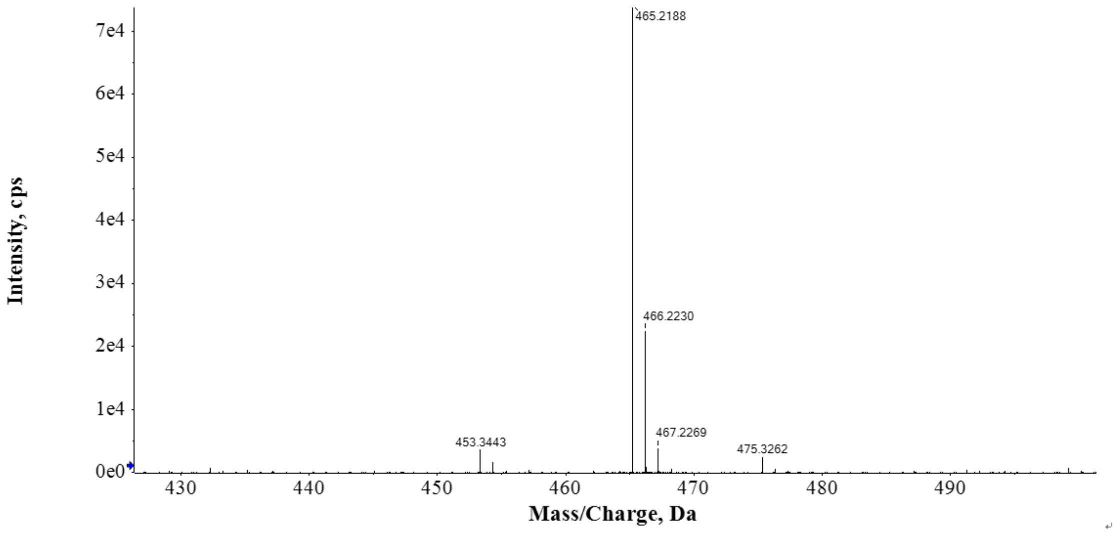

465.2188
466.2230
467.2269
453.3443
475.3262
7e4
6e4
5e4
4e4
3e4
2e4
1e4
0e0
430
440
450
460
470
480
490
Intensity, cps
Mass/Charge, Da

2e

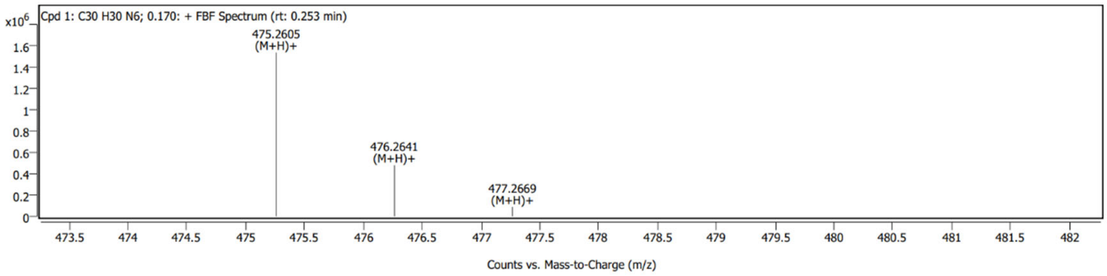

Spectrum Peaks

m/z	m/z (Calc)	Diff (ppm)	Abund	Height %	Height % (Calc)	Ion Species	Z
475.2605	475.2605	0.11	1538419	100.00	100.00	(M+H)+	1
476.2641	476.2635	1.31	480267	31.22	35.00	(M+H)+	1
477.2669	477.2664	0.91	89152	5.80	5.94	(M+H)+	1

2f

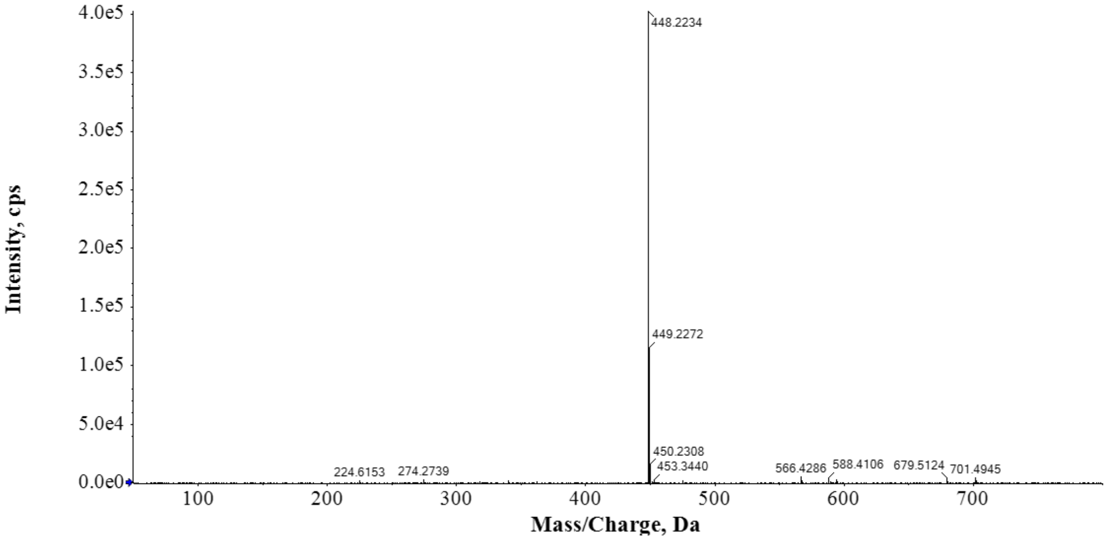

2g

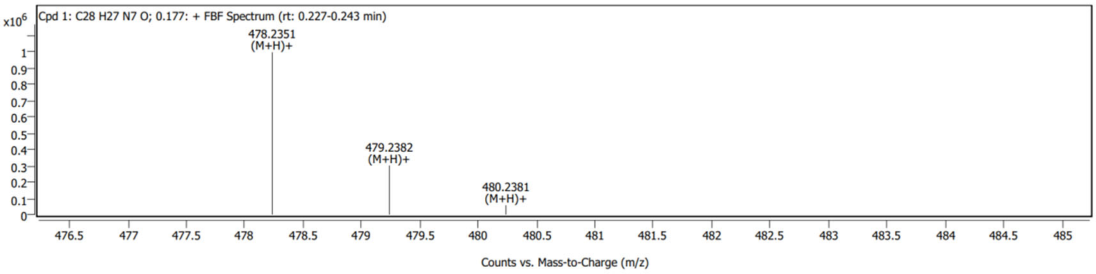

Spectrum Peaks

m/z	m/z (Calc)	Diff (ppm)	Abund	Height %	Height % (Calc)	Ion Species	Z
478.2351	478.2350	0.17	996295	100.00	100.00	(M+H)+	1
479.2382	479.2379	0.65	301563	30.27	33.20	(M+H)+	1
480.2381	480.2407	-5.33	56971	5.72	5.55	(M+H)+	1

2h

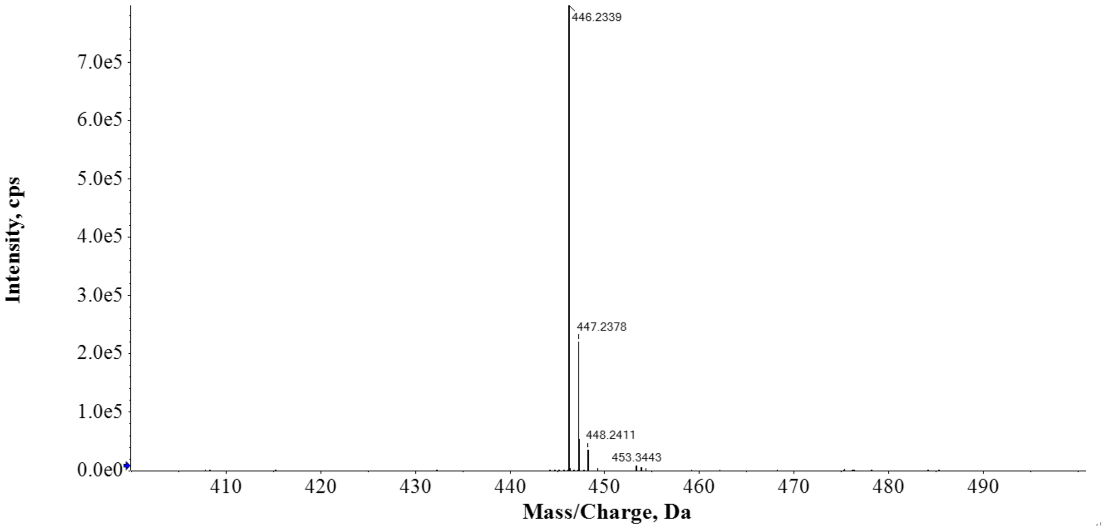

2i

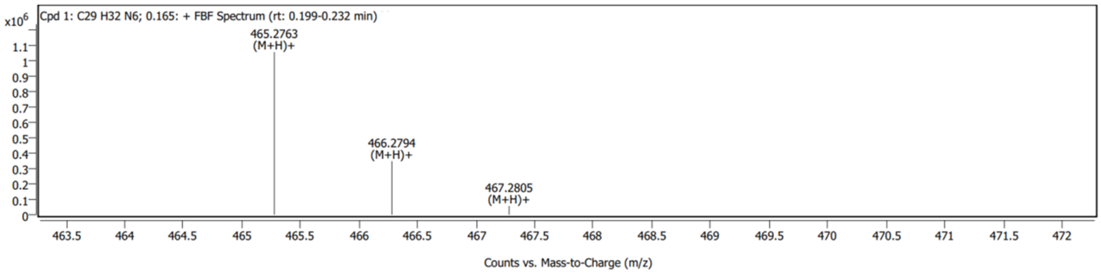

Spectrum Peaks

m/z	m/z (Calc)	Diff (ppm)	Abund	Height %	Height % (Calc)	Ion Species	Z
465.2763	465.2761	0.41	1053022	100.00	100.00	(M+H)+	1
466.2794	466.2791	0.59	347156	32.97	33.94	(M+H)+	1
467.2805	467.2821	-3.46	56699	5.38	5.58	(M+H)+	1

2j

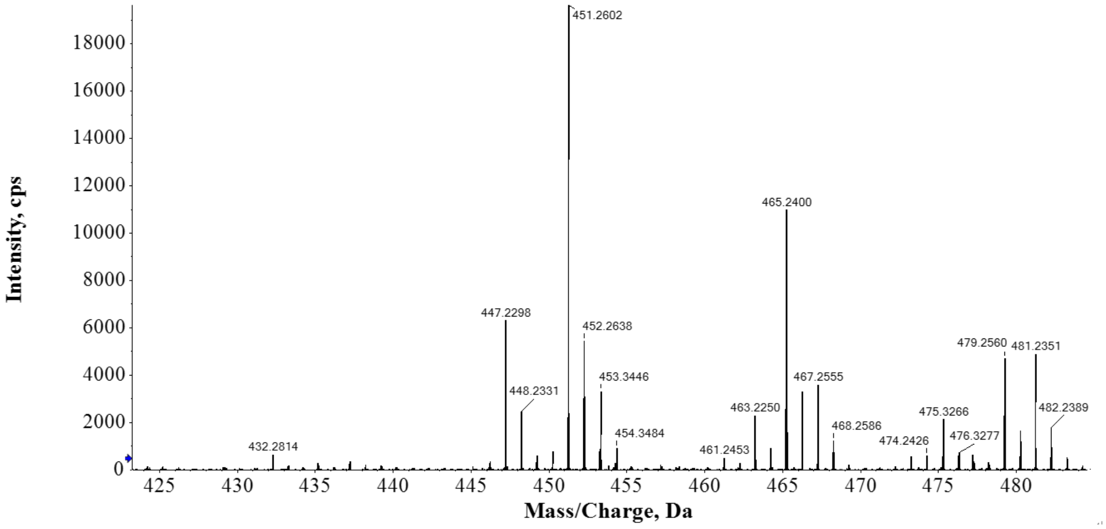

3a

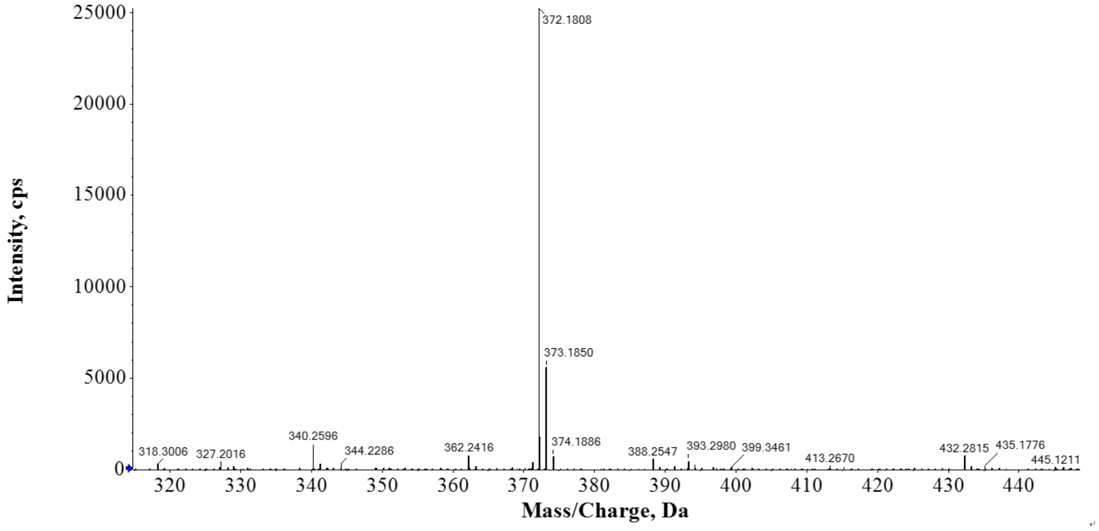
372.1808
373.1850
374.1886
318.3006
327.2016
340.2596
344.2286
362.2416
388.2547
393.2980
399.3461
413.2670
432.2815
435.1776
445.1211
Intensity, cps
Mass/Charge, Da

3b

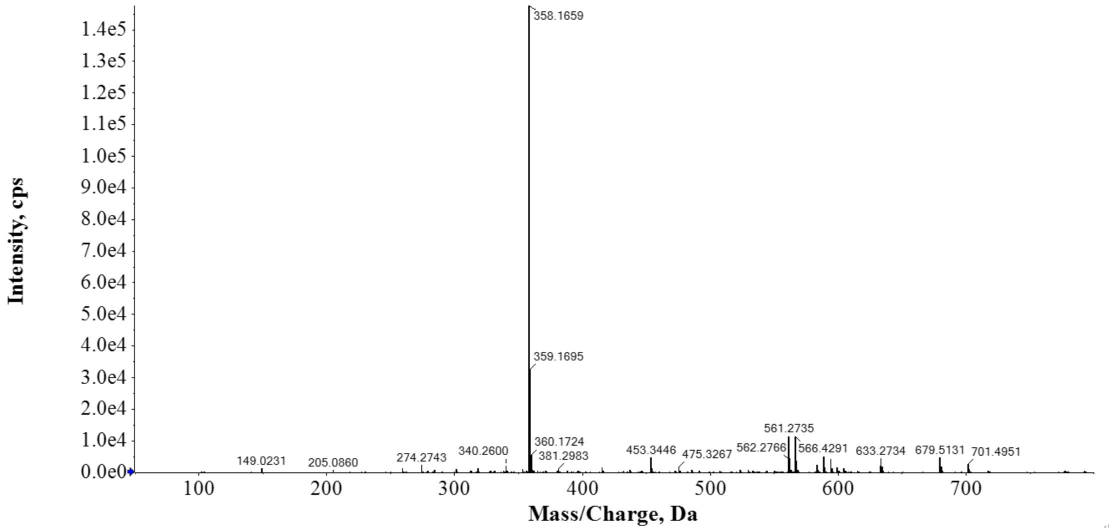
358.1659
359.1695
360.1724
381.2983
149.0231
205.0860
274.2743
340.2600
453.3446
475.3267
562.2766
561.2735
566.4291
633.2734
679.5131
701.4951
Intensity, cps
Mass/Charge, Da

3c

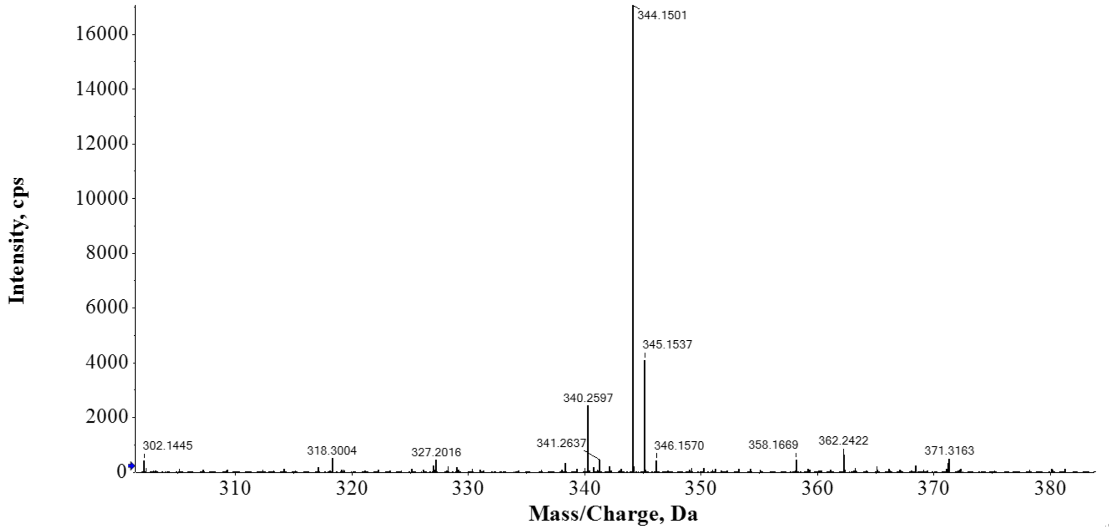
344.1501
345.1537
340.2597
341.2637
346.1570
302.1445
318.3004
327.2016
358.1669
362.2422
371.3163
Intensity, cps
Mass/Charge, Da

3d

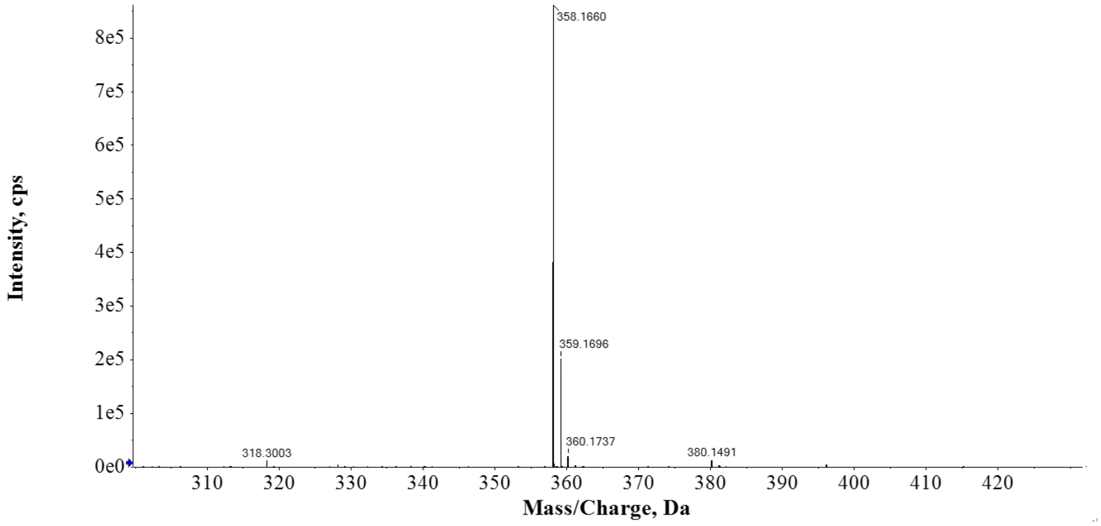

3e

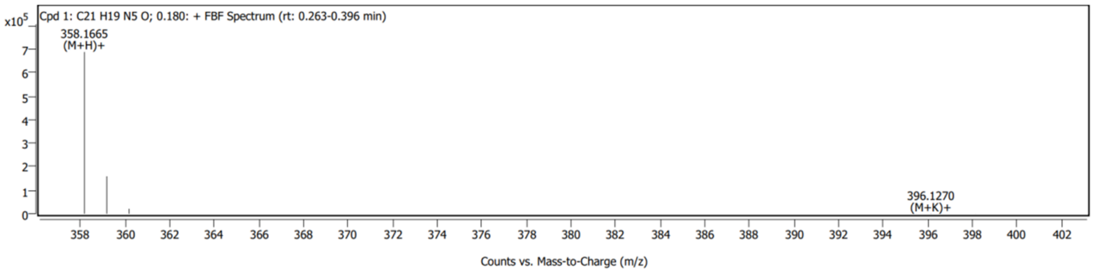

Spectrum Peaks

m/z	m/z (Calc)	Diff (ppm)	Abund	Height %	Height % (Calc)	Ion Species	Z
358.1665	358.1662	0.77	689243	100.00	100.00	(M+H)+	1
359.1696	359.1692	1.36	159425	23.13	24.81	(M+H)+	1
360.1725	360.1719	1.45	21248	3.08	3.16	(M+H)+	1
396.1270	396.1221	12.22	769	100.00	100.00	(M+K)+	1
398.1293	398.1225	17.08	32	4.21	10.37	(M+K)+	1

3f

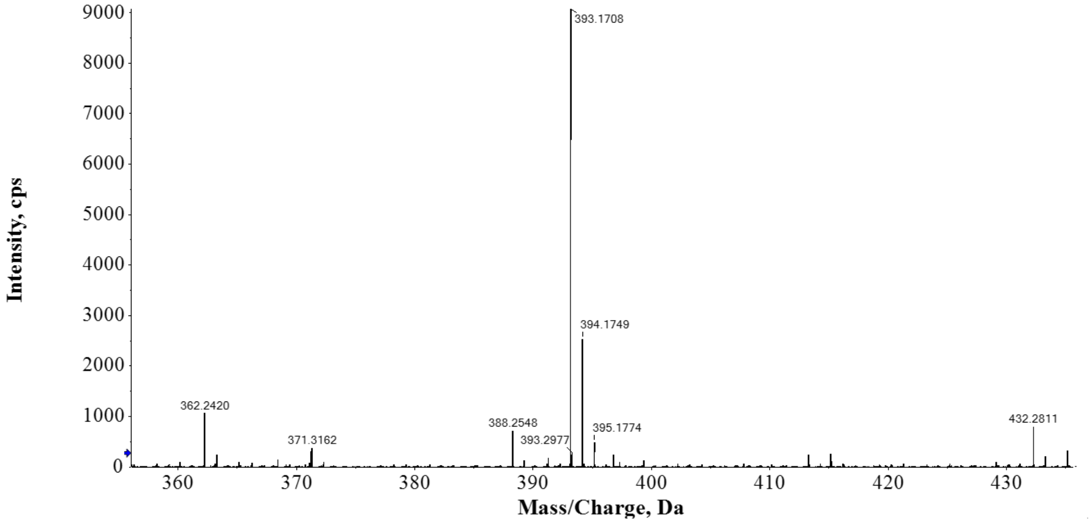

YL-939

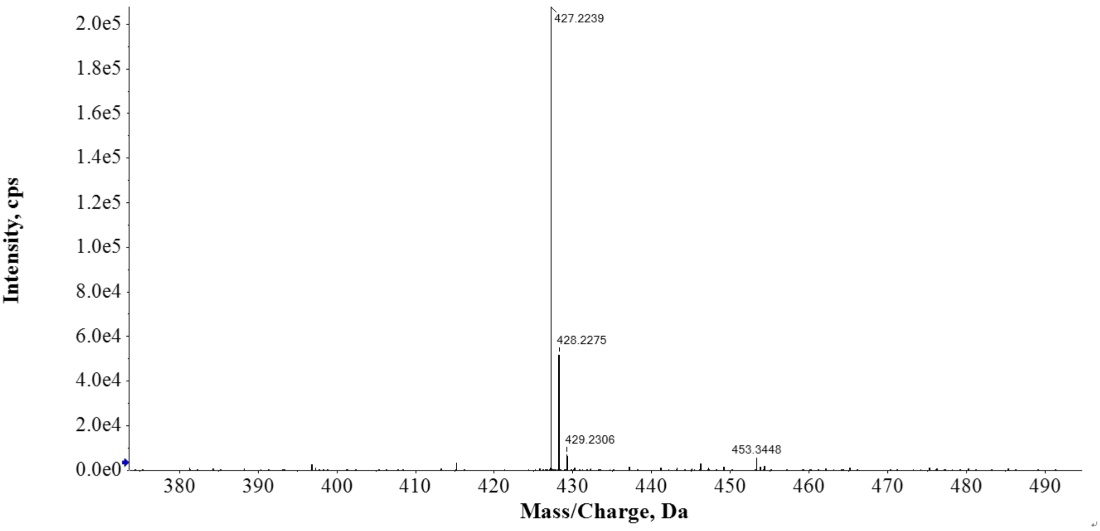

YL-939-1

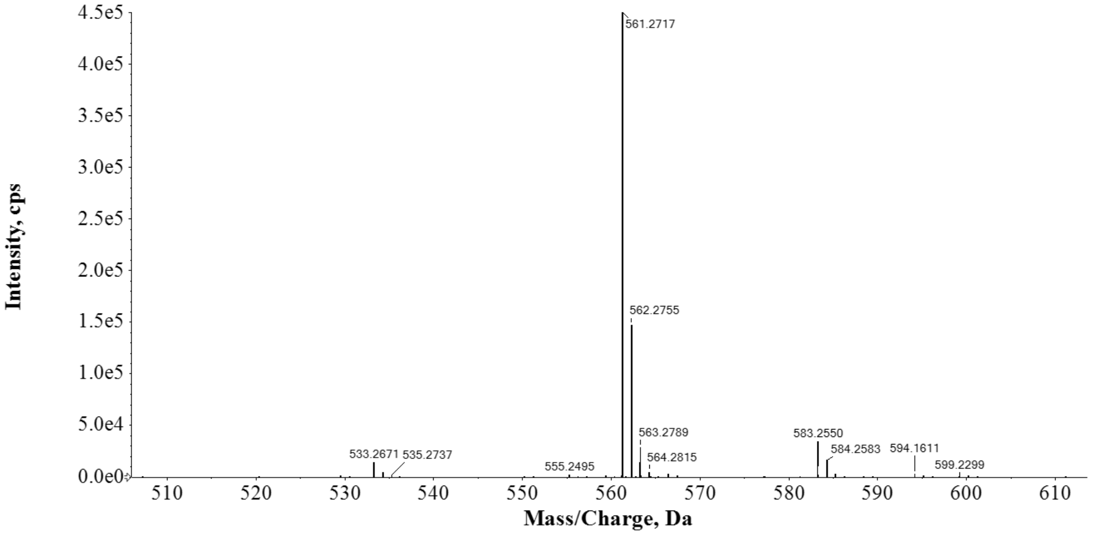

HPLC spectra of YL-939

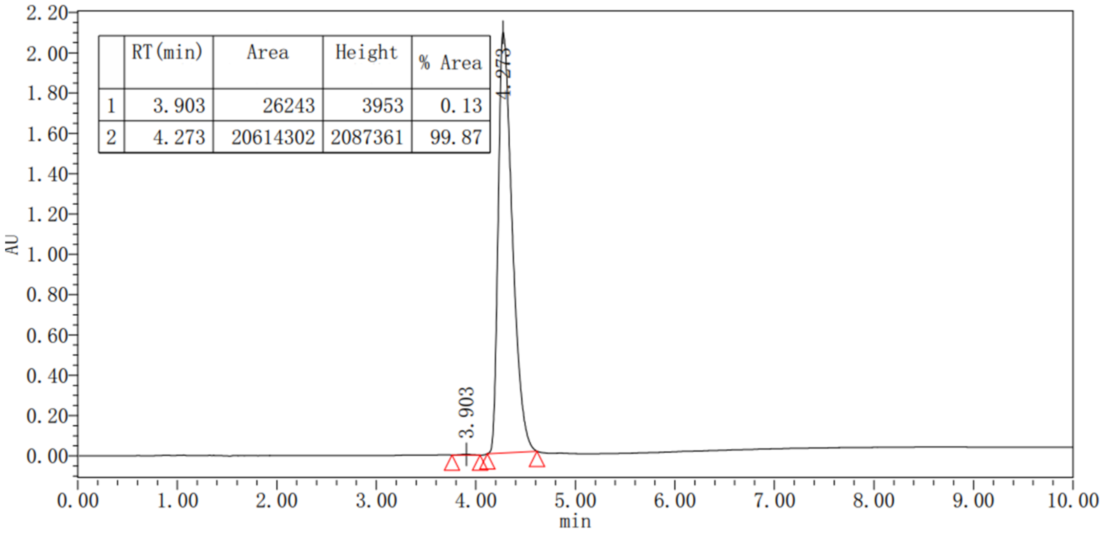

Supplement: Supplementary file 1 — Supplementary Information [file 41467_2022_35294_MOESM1_ESM.pdf]
