# Supplementary material for: Non-classical ferroptosis inhibition by a small molecule targeting PHB2
Source: Nat Commun. 2022 Dec 3;13:7473. doi: 10.1038/s41467-022-35294-2 (PMC9719519; doi:10.1038/s41467-022-35294-2)
Supplement: Supplementary file 2 — Reporting Summary [file 41467_2022_35294_MOESM2_ESM.pdf]

nature portfolio

Corresponding author(s): Shengyong Yang
Last updated by author(s): Nov 21, 2022

Reporting Summary

Nature Portfolio wishes to improve the reproducibility of the work that we publish. This form provides structure for consistency and transparency in reporting. For further information on Nature Portfolio policies, see our Editorial Policies and the Editorial Policy Checklist.

Statistics

For all statistical analyses, confirm that the following items are present in the figure legend, table legend, main text, or Methods section.

n/a	Confirmed	
☐	☒	The exact sample size (*n*) for each experimental group/condition, given as a discrete number and unit of measurement
☐	☒	A statement on whether measurements were taken from distinct samples or whether the same sample was measured repeatedly
☐	☒	The statistical test(s) used AND whether they are one- or two-sided<br>*Only common tests should be described solely by name; describe more complex techniques in the Methods section.*
☒	☐	A description of all covariates tested
☒	☐	A description of any assumptions or corrections, such as tests of normality and adjustment for multiple comparisons
☐	☒	A full description of the statistical parameters including central tendency (e.g. means) or other basic estimates (e.g. regression coefficient) AND variation (e.g. standard deviation) or associated estimates of uncertainty (e.g. confidence intervals)
☐	☒	For null hypothesis testing, the test statistic (e.g. *F*, *t*, *r*) with confidence intervals, effect sizes, degrees of freedom and *P* value noted<br>*Give P values as exact values whenever suitable.*
☒	☐	For Bayesian analysis, information on the choice of priors and Markov chain Monte Carlo settings
☒	☐	For hierarchical and complex designs, identification of the appropriate level for tests and full reporting of outcomes
☒	☐	Estimates of effect sizes (e.g. Cohen's *d*, Pearson's *r*), indicating how they were calculated

Our web collection on statistics for biologists contains articles on many of the points above.

Software and code

Policy information about availability of computer code

Data collection	CLARIOstar (v5.61); Bio-Rad CFX Maestro (v4.1.2433.1219); Agilent NovoSampler Pro; Thermo QE Plus; Bruker Spectrometer (AV-400); Agilent Mass Spectrometer (1200-G6410A); Bruker Mass Spectrometer (micrOTOFQ II 10203); Transmission electron microscopy (JEM-1400PLUS); Multi-functional fluorescence analyzer(Typhoon FLA 9500); Zeiss LSM 880; Pannoramic MIDI II scanner (3DHISTECH)
Data analysis	GraphPad Prism (v 8.30); CaseViewer2.4; Microsoft Excel (v2016); Olympus Image Viewer (v3.2.21633); NovoExpress (v1.4.1)

For manuscripts utilizing custom algorithms or software that are central to the research but not yet described in published literature, software must be made available to editors and reviewers. We strongly encourage code deposition in a community repository (e.g. GitHub). See the Nature Portfolio guidelines for submitting code & software for further information.

Data

Policy information about availability of data

All manuscripts must include a data availability statement. This statement should provide the following information, where applicable:

- Accession codes, unique identifiers, or web links for publicly available datasets
- A description of any restrictions on data availability
- For clinical datasets or third party data, please ensure that the statement adheres to our policy

The protein information used in this study are available in the UniProt human protein database (release 2016_07, 70630 sequences) under https://www.uniprot.org/.

All other data that support the conclusions are available from this published article, supplementary information files, source data file or corresponding authors on reasonable request.
Source data are provided with this paper.

Human research participants

Policy information about studies involving human research participants and Sex and Gender in Research.

Reporting on sex and gender	Not Applicable
Population characteristics	Not Applicable
Recruitment	Not Applicable
Ethics oversight	Not Applicable

Note that full information on the approval of the study protocol must also be provided in the manuscript.

Field-specific reporting

Please select the one below that is the best fit for your research. If you are not sure, read the appropriate sections before making your selection.

☒ Life sciences ☐ Behavioural & social sciences ☐ Ecological, evolutionary & environmental sciences

For a reference copy of the document with all sections, see nature.com/documents/nr-reporting-summary-flat.pdf

Life sciences study design

All studies must disclose on these points even when the disclosure is negative.

Sample size	Sample size estimation was not relevant for this study, as it does not report on a statistical evaluation of effects between two or more groups. Sample-size calculations were not done. For the animal study , the numbers of animals in each group meet the requirement for statistical analysis (at least 3 for each group), which is sufficient given the excellent technical reproducibility. For experiments other than animal studies, at least three samples were selected to meet the requirements for statistical analysis.
Data exclusions	No data has been excluded from the analyses presented in this manuscript.
Replication	To ensure reproducibility of experiment findings, each assay was performed at least two times to confirm the results.<br>EC50 and IC50 measurements were carried out with at least two biological replicates for each data point and these data were used to calculate mean values.<br>DSF assays were carried out with three biological replicates for each data point and these data were used to calculate mean values.<br>SPR assay and LC-MS/MS were performed in two biological replicates, but one result was showed.<br>Cytotoxicity and RT-qPCR were carried out with three biological replicates.<br>ROS assays and western blot assays were performed in at least two biological replicates.<br>Transmission electron microscope, fluorescence imaging experiments were performed twice, and the representative images were shown.<br>In animal studies, multiple mice were included in each group.
Randomization	For all in vivo experiments, sex- and age-matched mice were randomized into different experimental groups. Mice were randomly assigned to vehicle and treatment groups. For in vitro experiments, cells were randomized into different control/treatment groups. The tests were also randomly selected from all samples. The pictures were representatively shown.
Blinding	For the measurement of quantitative values, such as serum AST and ALT, serum and liver MDA, mRNA and protein expression of some key gene, data acquiring does not involve subjective judgments, therefore no blinding procedures were applied to the experimentalists involved. For the in vitro and in vivo experiments, blinding was not possible because researchers were involved in induction/treatment procedures. For the histological examinations, qualified pathologists were blinded to group allocation to ensure the assessment was unbiased.

Reporting for specific materials, systems and methods

We require information from authors about some types of materials, experimental systems and methods used in many studies. Here, indicate whether each material, system or method listed is relevant to your study. If you are not sure if a list item applies to your research, read the appropriate section before selecting a response.

Materials & experimental systems

n/a	Involved in the study
	☒ Antibodies
	☒ Eukaryotic cell lines
☒	☐ Palaeontology and archaeology
	☒ Animals and other organisms
☒	☐ Clinical data
☒	☐ Dual use research of concern

Methods

n/a	Involved in the study
☒	☐ ChIP-seq
	☒ Flow cytometry
☒	☐ MRI-based neuroimaging

Antibodies

Antibodies used

(1)PHB2 (E1Z5A) Rabbit mAb (#14085, Cell Signaling Technology, 1:1000 dilution);
(2)VDAC1 recombinant rabbit monoclonal antibody (ET1601-20, HUABIO, 1:1000 dilution);
(3)VDAC2 Antibody (#9412, Cell Signaling Technology, 1:1000 dilution);
(4)PHB1 Antibody (#2426, Cell Signaling Technology, 1:1000 dilution);
(5)α-tubulin (T0926, Sigma-Aldrich, 1:1000 dilution);
(6)β-actin (66009-1-lg, Proteintech, 1:1000 dilution);
(7)Anti-xCT Antibody (SLC7A11) (ab175186, abcam, 1:1000 dilution);
(8)Anti-Glutathione Peroxidase 4 antibody (ab125066, abcam, 1:1000 dilution);
(9)CD71 Polyclonal antibody (TFR1) (10084-2-AP, Proteintech, 1:1000 dilution);
(10)Ferroportin/SLC40A1 Antibody (NBP1-21502, Novusbio, 1:1000 dilution);
(11)DMT1 Polyclonal antibody (20507-1-AP, Proteintech, 1:1000 dilution);
(12)Anti-Ferritin Antibody (ab75973, abcam, 1:1000 dilution);
(13)FTH1 (D1D4) Rabbit mAb(#4393, Cell Signaling Technology, 1:1000 dilution);
(14)Ferritin Light Chain Rabbit mAb (A11241, ABclonal, 1:1000 dilution);
(15)NCOA4 (E8H8Z) Rabbit mAb (#66849, Cell Signaling Technolog)y, 1:1000 dilution);
(16)HRP-conjugated Affinipure Goat Anti-Rabbit IgG(H+L) (Proteintech, SA00001-2, 1:5000 dilution)
(17) HRP-conjugated Affinipure Goat Anti-Mouse IgG(H+L) (Proteintech, SA00001-1, 1:5000 dilution)

Validation

All antibodies were commercially available, and validated by manufacturers and/or citations. Manufacturer websites containing their validation data and/or citations, are listed below:
(1)PHB2 (E1Z5A) Rabbit mAb (#14085, Cell Signaling Technology, 1:1000 dilution): website (https://www.cellsignal.com/products/primary-antibodies/phb2-e1z5a-rabbit-mab/14085?site-search-type=Products&N=4294956287&Ntt=phb2&fromPage=plp&_requestid=2752006).
(2)VDAC1 recombinant rabbit monoclonal antibody (ET1601-20, HUABIO, 1:1000 dilution): website (http://www.huabio.cn/product/VDAC1-antibody-ET1601-20) and citations (PMID:35110683, etc).
(3)VDAC2 Antibody (#9412, Cell Signaling Technology, 1:1000 dilution): website (https://www.cellsignal.cn/products/primary-antibodies/vdac2-antibody/9412?site-search-type=Products&N=4294956287&Ntt=9412&fromPage=plp&_requestid=2733982) and citations (PMID: 34751412, etc).
(4)PHB1 Antibody (#2426, Cell Signaling Technology, 1:1000 dilution): website (https://www.cellsignal.cn/products/primary-antibodies/phb1-antibody/2426?site-search-type=Products&N=4294956287&Ntt=phb1+%28%232426&fromPage=plp&_requestid=2734292).
(5)α-tubulin (T0926, Sigma-Aldrich, 1:1000 dilution): website (https://www.sigmaaldrich.cn/CN/zh/product/sigma/t9026) and citations (PMID: 17494697, etc).
(6)β-actin (66009-1-lg, Proteintech, 1:1000 dilution): website (https://www.ptglab.com/products/Pan-Actin-Antibody-66009-1-Ig.htm) and citations (PMID: 32581380, etc).
(7)Anti-xCT Antibody (SLC7A11) (ab175186, abcam, 1:1000 dilution): website (https://www.abcam.cn/xct-antibody-epr82902-ab175186.html) and citations (PMID: 33365082, etc).
(8)Anti-Glutathione Peroxidase 4 antibody (ab125066, abcam, 1:1000 dilution): website (https://www.abcam.cn/glutathione-peroxidase-4-antibody-epncir144-ab125066.html) and citations (PMID:33431801, etc).
(9)CD71 Polyclonal antibody (TFR1) (10084-2-AP, Proteintech, 1:1000 dilution): website (https://www.ptgcn.com/products/TFRC-Antibody-10084-2-AP.htm) and citations (PMID: 34012073, etc).
(10)Ferroportin/SLC40A1 Antibody(NBP1-21502, Novusbio, 1:1000 dilution): website (https://www.novusbio.com/products/ferroportin-slc40a1-antibody_nbp1-21502) and citations (PMID: 35860023, etc).
(11)DMT1 Polyclonal antibody (20507-1-AP, Proteintech, 1:1000 dilution): website (https://www.ptglab.com/Products/SLC11A2-Antibody-20507-1-AP.html) and citations (PMID: 33529321, etc).
(12)Anti-Ferritin Antibody (ab75973, abcam, 1:1000 dilution): website (https://www.abcam.cn/ferritin-antibody-epr3004y-ab75973.html) and citations (PMID: 32892384, etc).
(13)FTH1 (D1D4) Rabbit mAb (#4393, Cell Signaling Technology, 1:1000 dilution): website (https://www.cellsignal.cn/products/primary-antibodies/fth1-d1d4-rabbit-mab/4393?site-search-type=Products&N=4294956287&Ntt=fth1+%28%234393&fromPage=plp&_requestid=2736037) and citations (PMID: 36137413, etc).
(14)Ferritin Light Chain Rabbit mAb (A11241, ABclonal, 1:1000 dilution): website (https://abclonal.com.cn/catalog/A11241) and citations (PMID: 35675856, etc).
(15)NCOA4 (E8H8Z) Rabbit mAb (#66849, Cell Signaling Technology, 1:1000 dilution): website (https://www.cellsignal.com/products/primary-antibodies/ncoa4-e8h8z-rabbit-mab/66849?site-search-type=Products&N=4294956287&Ntt=ncoa4+%28%2366849&fromPage=plp&_requestid=2754990).

Eukaryotic cell lines

Policy information about cell lines and Sex and Gender in Research

Cell line source(s)	ES-2 cell line was purchased from the National Collection of Authenticated Cell Cultures (No. TCHu111). HT1080, HT-29, A549, THP-1, L02, LX-2, Beas-2b, HUVEC, Arpe and hTERT-HPNE cell lines were purchased from the American Type Culture Collection (ATCC).
Authentication	All the cell lines were commercially available and have not been authenticated after receiving them.
Mycoplasma contamination	Mycoplasma testing confirmed negative at regular intervals.
Commonly misidentified lines (See ICLAC register)	No commonly misidentified cell lines were used.

Animals and other research organisms

Policy information about studies involving animals; ARRIVE guidelines recommended for reporting animal research, and Sex and Gender in Research

Laboratory animals	C57BL/6 mice were purchased from Chengdu Dashuo Laboratory Animal CO., Ltd. C57BL/6 male mice (age: 4-5 weeks) were used to evaluate the protective effect of YL-939 on APAP-induced liver injury. The mice were kept in cages with individual ventilation under 65% humidity and an ambient temperature of 21–23°C and a 12h–12h day–night cycle for housing and husbandry.
Wild animals	The study did not involve wild animals.
Reporting on sex	The effect of gender on the establishment of APAP hepatotoxicity model was assessed, and the results showed that female mice were resistant to the hepatotoxic effects of APAP as reported. Therefore, male mice were subsequently used to evaluate the protective effect of YL-939 against ferroptosis in vivo.<br>The sex of the animals used for each experiments were reported in the figure legends and methods.
Field-collected samples	The study did not involve samples collected from the field.
Ethics oversight	All procedures related to animal handling, care and treatment in efficacy studies were performed according to the guidelines approved by the Institute Animal Care and Use Committee (IACUC) of West China Hospital, Sichuan University (20211063A).

Note that full information on the approval of the study protocol must also be provided in the manuscript.

Flow Cytometry

Plots

Confirm that:

☒ The axis labels state the marker and fluorochrome used (e.g. CD4-FITC).

☒ The axis scales are clearly visible. Include numbers along axes only for bottom left plot of group (a 'group' is an analysis of identical markers).

☒ All plots are contour plots with outliers or pseudocolor plots.

☒ A numerical value for number of cells or percentage (with statistics) is provided.

Methodology

Sample preparation	The cells were respectively incubated with HBSS containing 5 µM of BODIPY-C11, H2DCFDA and MitoSox dye and the cells were returned to the cell culture incubator for 10 min. Cells were harvested and washed two times with HBSS followed by resuspending in 500 µL of HBSS.
Instrument	Agilent NovoSampler Pro
Software	NovoExpress (v1.4.1)
Cell population abundance	10,000 cells were analyzed for each sample.
Gating strategy	The starting cell population gating by Forward Scatter and Side Scatter was used to make sure doublet exclusion. Only single cell was used for analysis.

☒ Tick this box to confirm that a figure exemplifying the gating strategy is provided in the Supplementary Information.
